# Supplementary material for: Structure-Based Discovery of Receptor Activator of Nuclear Factor-κB Ligand (RANKL)-Induced Osteoclastogenesis Inhibitors
Source: Int J Mol Sci. 2023 Jul 10;24(14):11290. doi: 10.3390/ijms241411290 (PMC10379842; doi:10.3390/ijms241411290)
Supplement: Supplementary file 1 [file ijms-24-11290-s001.zip › SupportingFile_1_Figures-Tables.pdf]

Supplementary Figures and Tables

Figure S1…………………………………………………………………………………… S2
Figure S2…………………………………………………………………………………… S3
Figure S3...………………………………………………………………………………….. S4
Figure S4…………………………………………………………………………………….. S5
Table S1…..………………………………………………………………………………… S6
Table S2……..……………………………………………………………………………… S7
Table S3……….…………………………………………………………………..……… S8
Table S4…………………………………………………………………..……… S8–S11

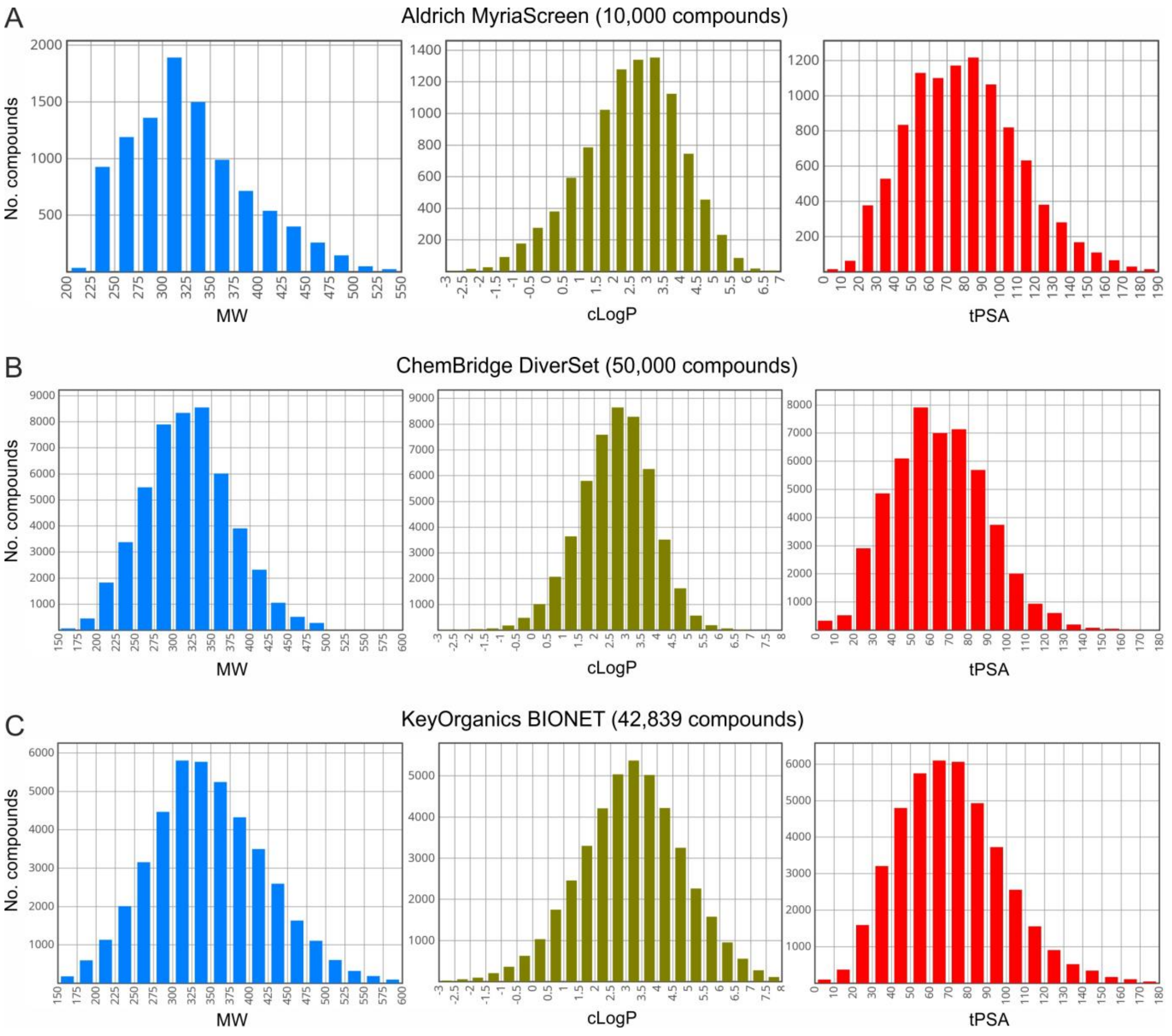

Figure S1. Distribution of key chemical properties of the compounds comprising MyriaScreen library from Aldrich (A), DiverSet compounds from ChemBridge (B) and Screening BIONET collection from KeyOrganics (C). Molecular weight (MW), log partition coefficient between *n*-octanol/water (cLogP) and topological polar surface area (tPSA)[1] were calculated using DataWarrior v5.5 (https://openmolecules.org/index.html).[2]

---

[1] Peter Ertl, Bernhard Rohde, and Paul Selzer. Fast Calculation of Molecular Polar Surface Area as a Sum of Fragment-Based Contributions and Its Application to the Prediction of Drug Transport Properties. J. Med. Chem. 2000, 43, 20, 3714–3717. doi 10.1021/jm000942e

[2] Thomas Sander, Joel Freyss, Modest von Korff, Christian Rufener. DataWarrior: An Open-Source Program For Chemistry Aware Data Visualization And Analysis. J Chem Inf Model 2015, 55, 460-473, doi 10.1021/ci500588j

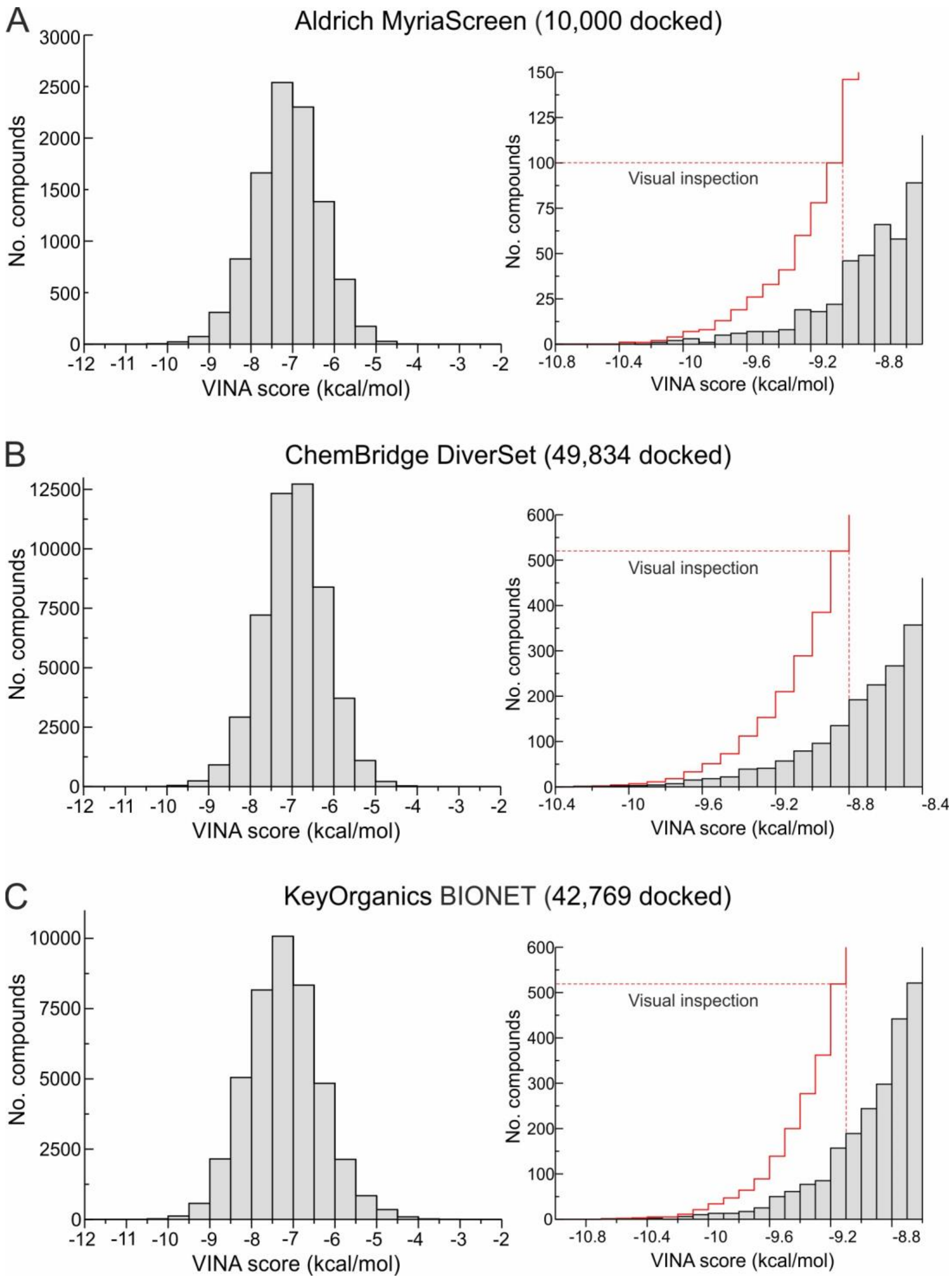

Figure S2. Distribution of VINA scores for the 3 libraries employed in screening the interface of human RANKL dimer (A–C are as in **Figure S1**). The number of docked compounds in parentheses is the final number of compounds converted successfully into 3D coordinates, excluding macrocycles, or inorganic compounds. Panels on the right display the score cutoff, and the cumulative histogram (red line) indicates the number of docked compounds that were visually investigated for appropriate complementarity and intermolecular interactions.

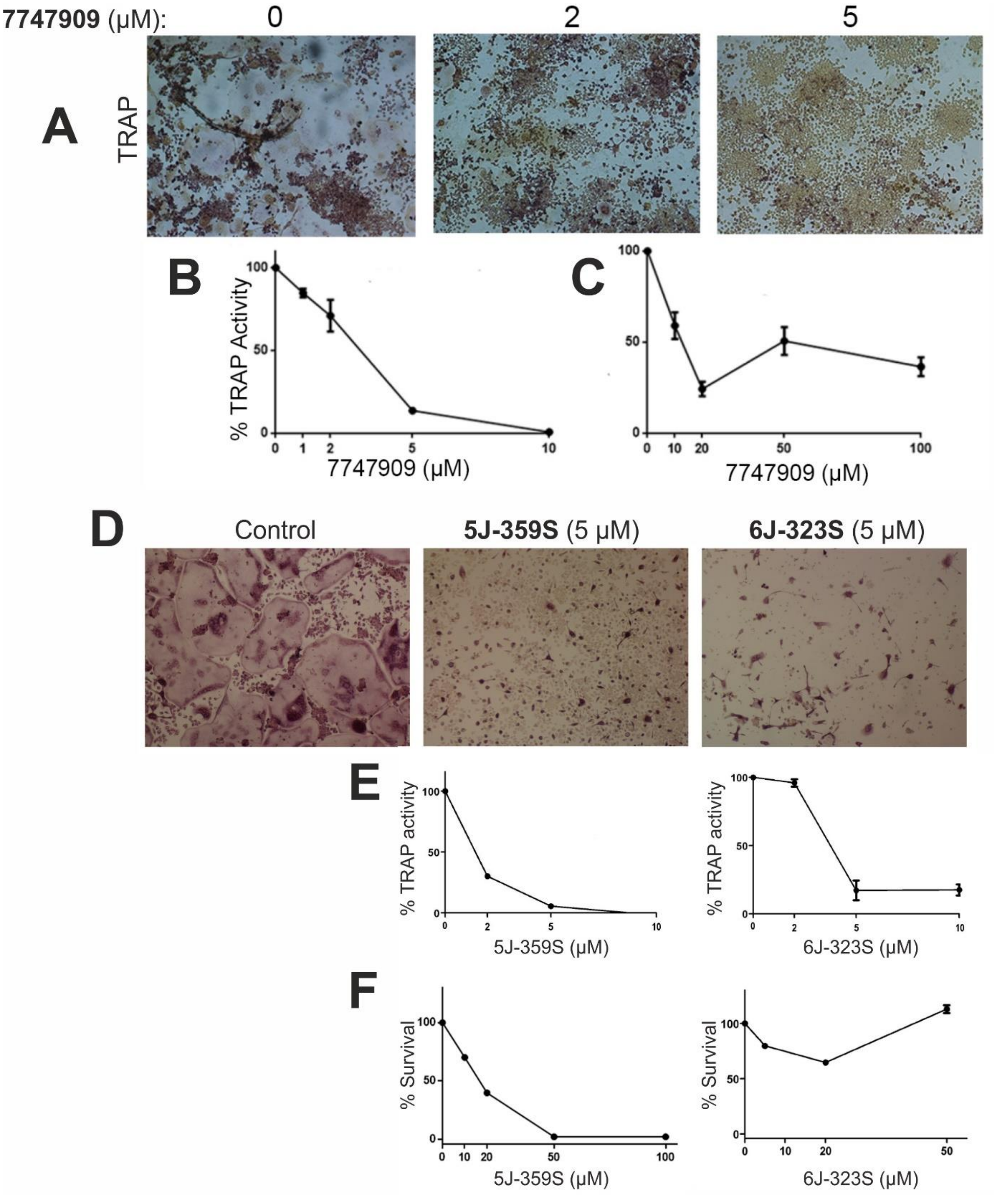

Figure S3. (A) TRAP activity assay for hit compound **7747909** at 0, 2 and 5 µM. (B) Plot of TRAP activity (%) for calculation of the IC_{50}. (C) Plot of cell survival (%) for calculation of the LC_{50}. (D) TRAP activity assays for hit compounds **5J-549S** and **6J-323S** at 5 µM. (E–F) Plots for the calculation of IC_{50} and LC_{50}, respectively.

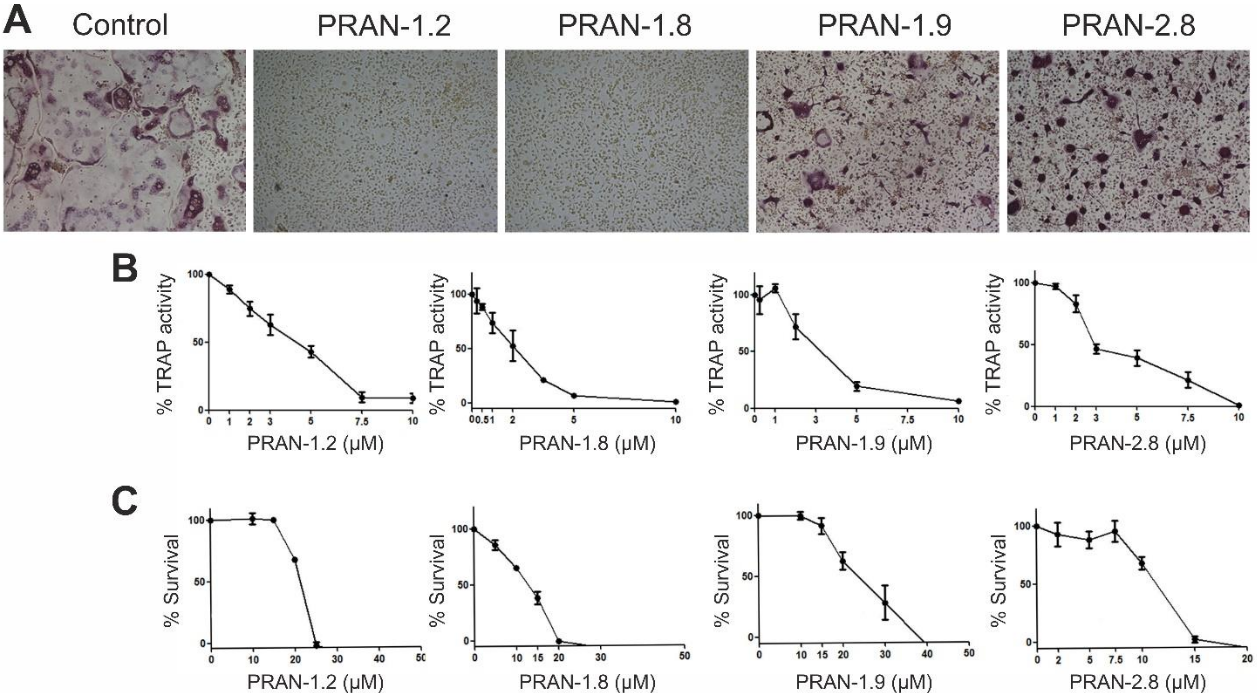

Figure S4. (A) TRAP activity assay for the 4 synthetic PRAN hits 5 μM. (B) Plots of TRAP activity (%) for calculation of the IC_{50}. (C) Plots of cell survival (%) for calculation of the LC_{50}.

Table S1. Identification codes and canonical SMILES representations of the 30 compounds selected for the first round of screening. Supplier IDs are colored as in **Table 1** and information from the Zinc15 database can be retrieved at: https://zinc15.docking.org/substances/*"ZINC ID"*

Supplier ID	ZINC ID	Canonical SMILES
R774383	ZINC000000639552	N#Cc1c2CCCc2c(n2c1nc1c2cccc1)Nc1cccc2c1cccc2
R818984	ZINC000019924571	O=C(c1ccccc1)c1cc(C(=O)c2cccc3ccccc23)n2cnc3ccccc3c12
R872172	ZINC000019833832	N#Cc1ccn2c(c1)c(cc2C(=O)c1ccc(cc1)c1ccccc1)C(=O)c1ccccc1
ST042026	ZINC000100787281	N#CC1=C(C)/C(=C\c2ccc(o2)c2ccc3c(c2)COC3=O)/C(=O)N(C1=O)CCc1ccccc1
ST041788	ZINC000015240419	CN1CCC2C(C1)c1cc(C)ccc1N2C(=O)CSc1nnnn1c1ccc2c(c1)OCO2
R679445	ZINC000017729590	N#Cc1c(NC2=NC(=Nc3sc4c(c3C#N)CCCC4)c3c2cccc3)sc2c1CCCC2
R897698	ZINC000012524283	[O-][N+](=O)c1cccc(c1)S(=O)(=O)N1CN(c2c1nc1ccccc1n2)c1ccc2c(c1)cccc2
R460974	ZINC000006156981	COc1ccc(C2CC(=O)C3=C(C2)Nc2ccccc2NC3c2cccc(OCc3ccccc3)c2)cc1
ST002674	ZINC000013475755	Brc1ccc(cc1)C1OC2(C3C1C(=O)N(C3=O)Cc1ccccc1)C(=O)c1c(C2=O)cccc1
ST018363	ZINC000017951225	O=C1CC(C)(C)CC2=C1C(c1ccc(o1)c1cccc(c1)[N+](=O)[O-])N(c1c(N2)cccc1) C(=O)C(F)(F)F
7685088	ZINC000013131857	CCc1ccc(cc1)C1Nc2nc(nn2C(C1)c1ccccc1C)N
7266825	ZINC000006225510	CCc1ccc(cc1)c1nc2ccccc2c(c1)C(=O)Nc1nnn[nH]1
5641450	ZINC000100504092	O=c1cccc2n1CC1CN(CC2C1)Cc1ccc2c3c1ccc1c3c(cc2)ccc1
7715520	ZINC000016294212	Fc1ccc(cc1)NC(=O)C1=C(C)NC2=C(C1c1cccc(c1)C(F)(F)F)C(=O)CCC2
7626463	ZINC000001140605	O=C(N1CCOCC1)COc1ccc(cc1)NS(=O)(=O)c1ccc2c(c1)Cc1c2cccc1
5187026	ZINC000012375757	O=C(c1ccc2c(c1)C(=O)N(C2=O)c1ccccc1)Nc1cccc(c1)C(=O)/C=C/c1ccccc1
5579819	ZINC000001188357	Cc1cccc(c1)NC(=O)c1cnn(c1NC(=O)c1ccccc1Br)c1ccccc1
**6747072**	ZINC000001167590	O=S(=O)(c1ccccc1)NC1c2cccc3c2c(C1Sc1nnnn1c1ccccc1)ccc3
5569062	ZINC000001216188	Cc1cccc(c1)NC(=O)c1ccc2c(c1)C(=O)N(C2=O)c1cccc(c1)C(=O)Nc1cccc(c1)C
**7756003**	ZINC000001114674	Cc1ccc(-c2nnc(N3CCN(C(=O)CSc4nc(C)cc(C)n4)CC3)c3ccccc23)cc1
**8P-504S**	ZINC000005739168	Cc1ccc(cc1)S(=O)(=O)N1CCN(CC1)c1cc(C)c2c(n1)c(C)ccc2
1T-0267	ZINC000012954249	Cc1ccc(c(c1)CSC1=NC(c2ccccc2F)C2=C(N1)c1ccccc1C2=O)C
6X-0309	ZINC000012961347	N#C/C(=C\c1cccc2c1cccc2)/c1scc(n1)CCN1C(=O)c2c(C1=O)cccc2
12R-0285	ZINC000012961042	Fc1cccc(c1)CSC1=NC(c2ccc3c(c2)OCO3)C2=C(N1)c1ccccc1C2=O
7H-063	ZINC000001396884	Clc1ccc(cc1)CSc1nn2c(n1)ncc1c2CC2N(C1=O)CCc1c2cccc1
8W-0823	ZINC000008873613	O=C1N(C)C(=O)C(=Cc2c(OCc3cccc(c3)C(F)(F)F)ccc3c2cccc3)C(=O)N1C
**5J-319S**	ZINC000001391858	Cc1cc2n(cnc2c(c1C)NS(=O)(=O)c1cccc(c1)C(F)(F)F)Cc1ccccc1C
11T-0208	ZINC000008762551	N#Cc1c(ccn2c1nc(n2)c1onc(c1C)c1c(F)cccc1Cl)c1ccc2c(c1)OCO2
7H-056	ZINC000008855436	O=C1CC2N(C(=O)/C/1=C/Nc1cccc(c1)Oc1ncc(cc1Cl)C(F)(F)F)CCc1c2cccc1
8L-940	ZINC000012956999	O=C(Nc1cccc2c1C(=O)N(C2=O)CCNc1ncc(cc1Cl)C(F)(F)F)Nc1cccc(c1)C(F)(F)F

Hit compounds are marked with bold IDs.

Table S2. Identification codes and canonical SMILES representations of the 20 hit-analogues that were employed in a second round of screening. Supplier IDs are colored as in **Table 2** and information from Zinc15 can be found at: https://zinc15.docking.org/substances/*"ZINC ID"*

	Supplier ID	ZINC ID	Canonical SMILES
a	7757551	ZINC000001114894	Cc1ccc(cc1)c1nnc(c2c1cccc2)N1CCN(CC1)C(=O)CSc1nc2c(o1)cccc2
	**7775352**	ZINC000009191255	Cc1ccc(-c2nnc(N3CCN(C(=O)COc4ccc([N+](=O)[O-])cc4)CC3)c3ccccc23)cc1
	**7775390**	ZINC000009424347	Cc1ccc(-c2nnc(N3CCN(C(=O)c4ccc([N+](=O)[O-])cc4)CC3)c3ccccc23)cc1
	7771348	ZINC000002849096	COc1ccccc1C(=O)N1CCN(c2nnc(-c3ccc(C)cc3)c3ccccc23)CC1
	**7774021**	ZINC000002391952	Cc1ccc(-c2nnc(N3CCN(C(=O)c4ccc(Cl)cc4)CC3)c3ccccc23)cc1
	**7753688**	ZINC000001114282	Cc1ccc(-c2nnc(N3CCN(C(=O)C4CCCCC4)CC3)c3ccccc23)cc1
	**7747909**	ZINC000004196835	Cc1ccc(-c2nnc(N3CCN(c4ccccn4)CC3)c3ccccc23)cc1
b	7553178	ZINC000002836916	Cc1ccc(S(=O)(=O)NC2c3cccc4cccc(c34)C2Sc2nnnn2-c2ccccc2)cc1
c	4J-400S	ZINC000001388569	Cc1cccc(Cn2cnc3c(NS(=O)(=O)c4cccc(C(F)(F)F)c4)c(C)c(C)cc32)c1
	4J-327S	ZINC000001388506	Cc1ccc(Cn2cnc3c(NS(=O)(=O)c4cccc(C(F)(F)F)c4)c(C)c(C)cc32)cc1
	5J-364S	ZINC000001391893	COc1ccc(Cn2cnc3c(NS(=O)(=O)c4cccc(C(F)(F)F)c4)c(C)c(C)cc32)cc1
	6J-330S	ZINC000003133395	COc1ccc(Cn2c(C)nc3c(NS(=O)(=O)c4cccc(C(F)(F)F)c4)c(C)c(C)cc32)cc1
	5J-351S	ZINC000001391883	Cc1ccccc1Cn1cnc2c(NS(=O)(=O)c3ccc(C(C)(C)C)cc3)c(C)c(C)cc21
	**6J-323S**	ZINC000003133393	Cc1cccc(Cn2c(C)nc3c(NS(=O)(=O)c4ccc(C(C)(C)C)cc4)c(C)c(C)cc32)c1
	5J-305S	ZINC000001391846	COc1ccc(S(=O)(=O)Nc2c(C)c(C)cc3c2ncn3Cc2ccccc2C)cc1
	**5J-359S**	ZINC000001391890	COc1ccc(Cn2cnc3c(NS(=O)(=O)c4ccc(OC)cc4)c(C)c(C)cc32)cc1
	5J-345S	ZINC000001391877	Cc1ccccc1Cn1cnc2c(NS(=O)(=O)c3ccc(Cl)c(Cl)c3)c(C)c(C)cc21
d	8P-505S	ZINC000001402689	Cc1cc(N2CCN(S(=O)(=O)c3ccc(F)cc3)CC2)nc2c(C)cccc12
	8P-517S	ZINC000001402699	Cc1cc(N2CCN(S(=O)(=O)c3ccc(Br)cc3)CC2)nc2c(C)cccc12
	8P-515S	ZINC000001402698	Cc1cc(N2CCN(S(=O)(=O)Cc3ccccc3)CC2)nc2c(C)cccc12
	7958467	ZINC000006174335	COc1ccc(OC)c2c(C)cc(N3CCN(S(=O)(=O)c4ccc(C)cc4)CC3)nc12

Analogues of the hit-compound: (a) **7756003**; (b) **6747072**; (c) **5J-319S**; (d) **8P-504S**

Table S3. Comparable analysis of 3 hit compounds in differentiation of primary BM cells and RAW264.7 cells towards osteoclast formation and cytotoxicity. The efficacy of the compounds was evaluated for activity in the TRAP assay (IC_{50}) and toxicity in both BMMs and RAW264.7 cells (LC_{50}).

	BMMs cells		RAW264.7 cells	
**Compound**	**TRAP activity IC_{50} (μM)**	**Toxicity LC_{50} (μM)**	**TRAP activity IC_{50} (μM)**	**Toxicity LC_{50} (μM)**
**5J-319S**	1.11 ± 0.35	41.5 ± 0.1	2.28 ± 0.60	43.5 ± 1.3
**8P-504S**	3.53 ± 0.35	107 ± 2.0	3.48 ± 0.34	>100
**6747072**	2.90 ± 0.97	>200	2.90 ± 1.00	>200

Table S4 (contd. p. S8–S10). Characterization and purity information for the 30 synthetic PRAN compounds with their chemical structures shown inset (left). Yields are reported for the last step of amine coupling with the corresponding sulfonyl chloride (**Scheme 5**), whereas ^{1}H and ^{13}C NMR chemical shifts are reported in ppm (*δ*) relative to residual proton peaks in deuterated solvents.

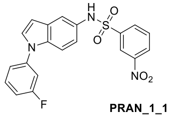
PRAN_1_1

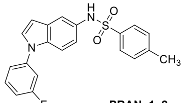
PRAN_1_2

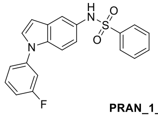
PRAN_1_3

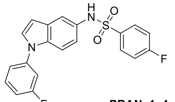
PRAN_1_4

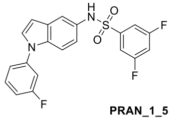
PRAN_1_5

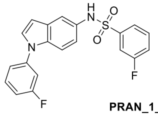
PRAN_1_6

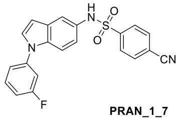
PRAN_1_7

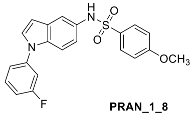
PRAN_1_8

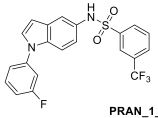
PRAN_1_9

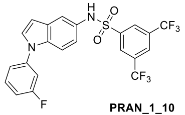
PRAN_1_10

Compound ID	Yield (%)	^{1}H NMR (500 MHz, $CDCl_3$)	^{13}C NMR (126 MHz, $CDCl_3$)	Chemical formula $[M+H]^+$	Calc. mass $[M+H]^+$	Found mass $[M+H]^+$	HPLC purity (%)
PRAN_1_1	96.7	8.64 (s, 1H), 8.37 (d, J = 7.7 Hz, 1H), 8.03 (d, J = 7.4 Hz, 1H), 7.63 (t, J = 7.9 Hz, 1H), 7.52 – 7.40 (m, 3H), 7.34 (s, 1H), 7.26 (d, J = 7.7 Hz, 1H), 7.18 (d, J = 9.3 Hz, 1H), 7.09 (br s, 2H), 6.94 (d, J = 8.6 Hz, 1H), 6.61 (s, 1H).	164.32, 148.30, 141.46, 140.86, 134.45, 133.08, 131.18, 131.11, 130.37, 130.01, 129.28, 128.27, 127.35, 122.71, 119.78, 117.33, 113.95, 113.78, 111.75, 111.56, 111.37, 104.47.	$C_{20}H_{15}FN_3O_4S^+$	412.08	411.60	97.5
PRAN_1_2	93.0	7.65 (d, J = 7.3 Hz, 2H), 7.49 (d, J = 6.7 Hz, 1H), 7.43 (s, 2H), 7.33 (s, 1H), 7.27 (d, J = 7.4 Hz, 1H), 7.22 (d, J = 7.4 Hz, 3H), 7.08 (t, J = 5.9 Hz, 1H), 6.94 (d, J = 8.1 Hz, 1H), 6.67 (s, 1H), 6.62 (s, 1H), 2.39 (s, 3H).	164.34, 143.67, 141.09, 141.02, 136.40, 134.11, 131.13, 131.06, 129.67, 129.51, 128.85, 127.52, 119.69, 116.76, 113.74, 113.57, 111.64, 111.45, 111.05, 104.50, 21.67.	$C_{21}H_{18}FN_2O_2S^+$	381.11	380.75	99.8
PRAN_1_3	80.6	7.77 (d, J = 7.6 Hz, 2H), 7.50 (dd, J = 21.7, 7.0 Hz, 2H), 7.42 (s, 4H), 7.32 (s, 1H), 7.26 (d, J = 8.8 Hz, 1H), 7.19 (d, J = 9.4 Hz, 1H), 7.07 (t, J = 7.4 Hz, 1H), 6.93 (d, J = 8.6 Hz, 1H), 6.73 (s, 1H), 6.61 (s, 1H).	162.37, 141.06, 139.32, 134.19, 132.90, 131.14, 131.07, 129.95, 129.31, 129.05, 128.91, 127.49, 119.80, 116.97, 113.77, 113.60, 111.66, 111.48, 111.07, 104.49.	$C_{20}H_{16}FN_2O_2S^+$	367.09	366.65	99.4
PRAN_1_4	100	7.75 (dd, J = 8.5, 5.1 Hz, 2H), 7.54 – 7.39 (m, 4H), 7.35 (s, 1H), 7.28 (t, J = 4.1 Hz, 1H), 7.20 (d, J = 9.6 Hz, 1H), 7.10 (t, J = 8.1 Hz, 2H), 6.91 (d, J = 8.7 Hz, 1H), 6.64 (s, 1H), 6.54 (s, 1H).	166.29, 164.35, 164.26, 140.97, 135.28, 134.28, 131.17, 131.09, 130.27, 130.19, 129.05, 119.83, 117.10, 116.39, 116.21, 113.85, 113.69, 111.71, 111.58, 111.17, 104.48.	$C_{20}H_{15}F_2N_2O_2S^+$	385.08	384.60	99.7
PRAN_1_5	99.0	7.53 – 7.41 (m, 3H), 7.37 (d, J = 3.0 Hz, 1H), 7.33 – 7.24 (m, 3H), 7.22 (d, J = 9.5 Hz, 1H), 7.10 (t, J = 8.0 Hz, 1H), 6.99 (t, J = 8.3 Hz, 1H), 6.92 (d, J = 8.5 Hz, 1H), 6.66 (d, J = 2.8 Hz, 1H), 6.52 (s, 1H).	162.08, 131.19, 131.12, 129.28, 128.30, 119.87, 117.45, 113.97, 113.81, 111.83, 111.64, 111.33, 111.24, 111.18, 111.07, 111.02, 108.78, 108.58, 108.38, 104.51.	$C_{20}H_{14}F_3N_2O_2S^+$	403.07	402.60	99.8
PRAN_1_6	88.0	7.56 (d, J = 7.3 Hz, 1H), 7.52 – 7.37 (m, 5H), 7.34 (s, 1H), 7.27 (d, J= 7.8 Hz, 1H), 7.25 – 7.16 (m, 2H), 7.08 (t, J = 7.8 Hz, 1H), 6.97 –6.80 (m, 2H), 6.63 (s, 1H).	164.34, 163.40, 162.37, 161.40, 141.28, 140.92, 134.32, 131.16, 131.08, 130.86, 130.80, 129.98, 129.07, 128.84, 123.30, 120.23, 120.06, 119.78, 117.13, 115.01, 114.81, 113.84, 113.68, 111.72, 111.53, 111.18, 104.50.	$C_{20}H_{15}F_2N_2O_2S^+$	385.08	384.80	99.5
PRAN_1_7	93.0	7.87 (d, J = 8.2 Hz, 2H), 7.71 (d, J = 8.2 Hz, 2H), 7.49 (dd, J = 14.6, 7.9 Hz, 1H), 7.47 – 7.39 (m, 2H), 7.35 (d, J = 3.1 Hz, 1H), 7.30 – 7.22 (m, 1H), 7.23 – 7.11 (m, 2H), 7.09 (t, J = 8.2 Hz, 1H), 6.97 – 6.82 (m, 1H), 6.62 (s, 1H).	164.30, 162.33, 143.36, 140.82, 134.37, 132.84, 131.19, 129.95, 129.26, 128.34, 128.10, 119.75, 117.44, 117.21, 116.53, 113.94, 113.78, 111.69, 111.50, 111.31, 104.45.	$C_{21}H_{15}FN_3O_2S^+$	392.09	391.65	99.4
PRAN_1_8	100	7.74 (d, J = 8.8 Hz, 2H), 7.45 (t, J = 7.1 Hz, 1H), 7.41 (d, J = 8.8 Hz, 1H), 7.38 (s, 1H), 7.30 (s, 1H), 7.25 (d, J = 7.9 Hz, 1H), 7.20 – 7.11 (m, 2H), 7.09 – 7.02 (m, 1H), 6.97 (d, J = 8.8 Hz, 1H), 6.87 (d, J = 8.6 Hz, 2H), 6.60 (s, 1H), 3.80 (s, 3H).	164.27, 163.00, 162.30, 141.05, 133.94, 131.09, 130.87, 129.90, 129.74, 129.62, 128.74, 119.49, 116.43, 114.18, 113.45, 111.51, 111.32, 110.98, 104.47, 55.60.	$C_{21}H_{18}FN_2O_3S^+$	397.10	396.70	99.6
PRAN_1_9	95.8	8.04 (s, 1H), 7.90 (d, J = 7.5 Hz, 1H), 7.79 (d, J = 7.4 Hz, 1H), 7.57 (t, J = 7.6 Hz, 1H), 7.53 – 7.39 (m, 3H), 7.35 (s, 1H), 7.27 (d, J = 8.3 Hz, 1H), 7.19 (d, J = 9.3 Hz, 1H), 7.09 (t, J = 7.3 Hz, 1H), 6.91 (d, J = 8.4 Hz, 1H), 6.77 (s, 1H), 6.63 (s, 1H).	158.78, 126.72, 124.75, 102.85, 96.83, 93.47, 93.09, 92.38, 92.16, 91.88, 91.56, 90.89, 86.97, 82.33, 82.17, 79.87, 76.28, 76.12, 74.12, 73.93, 73.62, 66.85.	$C_{21}H_{15}F_4N_2O_2S^+$	435.08	434.90	98.6
PRAN_1_10	88.0	8.15 (s, 2H), 8.03 (s, 1H), 7.54 – 7.41 (m, 3H), 7.37 (s, 1H), 7.30 – 7.24 (m, 1H), 7.19 (d, J = 8.8 Hz, 1H), 7.10 (t, J = 7.2 Hz, 1H), 6.88 (d, J = 5.5 Hz, 1H), 6.79 (s, 1H), 6.64 (s, 1H).	164.37, 141.98, 140.87, 140.78, 138.24, 134.75, 132.92, 132.65, 131.23, 131.16, 130.12, 129.52, 127.82, 126.40, 120.12, 119.92, 118.07, 114.08, 113.92, 111.87, 111.68, 111.46, 104.48.	$C_{22}H_{14}F_7N_2O_2S^+$	503.07	502.65	98.1

	Compound ID	Yield (%)	^{1}H NMR (500 MHz, $CDCl_3$)	^{13}C NMR (126 MHz, $CDCl_3$)	Chemical formula $[M+H]^+$	Calc. mass $[M+H]^+$	Found mass $[M+H]^+$	HPLC purity (%)
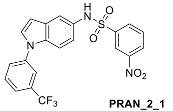 PRAN_2_1	PRAN_2_1	99.8	8.63 (s, 1H), 8.37 (d, J = 7.8 Hz, 1H), 8.02 (d, J = 7.5 Hz, 1H), 7.70 (s, 1H), 7.67 – 7.58 (m, 4H), 7.43 (s, 1H), 7.41 – 7.32 (m, 2H), 6.99 – 6.85 (m, 2H), 6.64 (s, 1H).	148.34, 141.40, 139.95, 134.55, 133.08, 132.68, 132.42, 130.61, 130.41, 130.10, 129.26, 128.38, 127.54, 127.41, 123.63, 122.74, 121.20, 119.98, 117.48, 111.15, 104.84.	$C_{21}H_{15}F_3N_3O_4S^+$	462.07	461.70	99.6
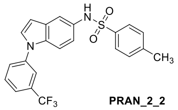 PRAN_2_2	PRAN_2_2	99.8	7.73 – 7.56 (m, 6H), 7.43 (s, 1H), 7.39 – 7.30 (m, 2H), 7.20 (d, J = 7.8 Hz, 2H), 6.95 (d, J = 8.2 Hz, 1H), 6.87 (d, J = 9.5 Hz, 1H), 6.63 (s, 1H), 2.36 (s, 3H).	143.70, 140.15, 136.38, 134.12, 132.60, 132.34, 130.54, 130.00, 129.69, 128.75, 127.52, 127.38, 123.36, 121.02, 119.77, 116.72, 110.78, 104.86, 21.65.	$C_{22}H_{18}F_3N_2O_2S^+$	431.10	430.65	99.4
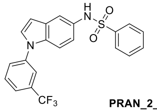 PRAN_2_3	PRAN_2_3	92.8	7.78 (d, J = 7.9 Hz, 2H), 7.70 (s, 1H), 7.63 (t, J = 6.3 Hz, 3H), 7.51 (t, J = 7.3 Hz, 1H), 7.42 (dd, J = 14.1, 6.2 Hz, 3H), 7.36 (d, J = 8.7 Hz,1H), 7.33 (d, J = 2.9 Hz, 1H), 6.99 (s, 1H), 6.95 (d, J = 8.7 Hz, 1H), 6.62 (s, 1H).	140.10, 139.26, 134.15, 132.92, 130.53, 129.99, 129.55, 129.06, 128.79, 127.48, 127.39, 123.39, 121.00, 119.83, 116.86, 110.78, 104.84.	$C_{21}H_{16}F_3N_2O_2S^+$	417.09	416.65	99.9
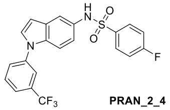 PRAN_2_4	PRAN_2_4	90.0	7.79 (dd, J = 7.9, 5.1 Hz, 2H), 7.71 (s, 1H), 7.63 (d, J = 7.3 Hz, 3H), 7.45 (s, 1H), 7.36 (dd, J = 16.4, 5.8 Hz, 2H), 7.08 (dd, J = 15.2, 6.3 Hz, 3H), 6.96 (d, J = 8.6 Hz, 1H), 6.64 (s, 1H).	166.28, 164.25, 140.05, 135.20, 134.23, 132.60, 132.34, 130.56, 130.28, 130.21, 130.01, 129.32, 128.91, 127.41, 123.44, 121.03, 119.82, 116.93, 116.40, 116.22, 110.88, 104.83.	$C_{21}H_{15}F_4N_2O_2S^+$	435.08	434.60	99.5
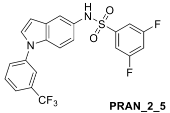 PRAN_2_5	PRAN_2_5	81.0	7.78 – 7.56 (m, 4H), 7.48 (s, 1H), 7.44 – 7.30 (m, 4H), 7.08 (s, 1H), 6.98 (d, J = 7.4 Hz, 2H), 6.68 (s, 1H).	163.96, 161.80, 142.50, 140.00, 134.47, 132.63, 132.36, 130.57, 130.07, 129.11, 128.59, 127.50, 123.55, 121.12, 119.85, 117.26, 111.25, 111.03, 108.80, 108.60, 108.40, 104.86.	$C_{21}H_{14}F_5N_2O_2S^+$	453.07	452.85	99.8
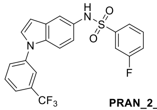 PRAN_2_6	PRAN_2_6	93.0	7.71 (s, 1H), 7.64 (t, J = 5.8 Hz, 3H), 7.59 (d, J = 7.8 Hz, 1H), 7.51 (d, J = 8.0 Hz, 1H), 7.46 (s, 1H), 7.43 – 7.36 (m, 2H), 7.34 (d, J = 3.1 Hz, 1H), 7.21 (t, J = 8.1 Hz, 1H), 7.16 (s, 1H), 6.97 (d, J = 8.6 Hz, 1H), 6.64 (d, J = 3.0 Hz, 1H).	163.38, 161.38, 141.20, 140.04, 134.29, 132.61, 132.32, 130.89, 130.83, 130.55, 130.02, 129.08, 128.93, 127.43, 123.43, 123.30, 121.04, 120.24, 120.08, 119.81, 117.01, 115.00, 114.81, 110.90, 104.84.	$C_{21}H_{15}F_4N_2O_2S^+$	435.08	434.65	99.6
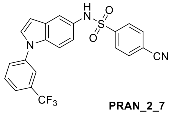 PRAN_2_7	PRAN_2_7	77.1	7.89 (d, J = 8.1 Hz, 2H), 7.71 (d, J = 7.7 Hz, 3H), 7.66 (br s, 3H), 7.44 (s, 1H), 7.39 (dd, J = 9.6, 6.0 Hz, 2H), 7.28 (d, J = 3.5 Hz, 1H), 6.95 (d, J = 8.7 Hz, 1H), 6.65 (d, J = 2.8 Hz, 1H).	143.33, 139.88, 134.38, 132.86, 130.59, 130.00, 129.17, 128.55, 128.10, 127.43, 123.58, 121.06, 119.79, 117.44, 117.17, 116.52, 111.04, 104.78.	$C_{22}H_{15}F_3N_3O_2S^+$	442.08	440.65	99.7
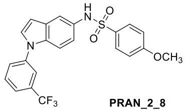 PRAN_2_8	PRAN_2_8	83.4	7.74 – 7.56 (m, 6H), 7.42 (s, 1H), 7.35 (dd, J = 17.5, 5.8 Hz, 2H), 6.93 (d, J = 8.4 Hz, 1H), 6.87 (d, J = 8.7 Hz, 2H), 6.66 – 6.58 (m, 2H), 3.81 (s, 3H).	163.09, 140.15, 134.15, 130.97, 130.54, 130.00, 129.78, 129.63, 128.77, 127.40, 123.38, 121.04, 119.90, 116.86, 114.22, 110.78, 104.86, 55.68.	$C_{22}H_{18}F_3N_2O_3S^+$	447.10	446.65	99.9
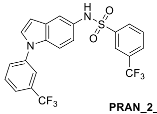 PRAN_2_9	PRAN_2_9	90.6	8.04 (s, 1H), 7.93 (d, J = 7.8 Hz, 1H), 7.78 (d, J = 7.7 Hz, 1H), 7.70 (s, 1H), 7.64 (s, 4H), 7.56 (t, J = 7.8 Hz, 1H), 7.44 (s, 1H), 7.36 (dd, J = 13.9, 5.7 Hz, 2H), 7.04 (s, 1H), 6.93 (d, J = 8.7 Hz, 1H), 6.65 (s, 1H).	140.41, 140.02, 134.44, 132.37, 131.95, 131.46, 130.71, 130.58, 130.07, 129.84, 129.55, 129.09, 128.76, 127.46, 124.59, 123.52, 121.09, 120.03, 117.44, 110.98, 104.83.	$C_{22}H_{15}F_6N_2O_2S^+$	485.08	484.65	99.8
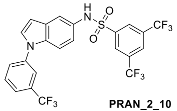 PRAN_2_10	PRAN_2_10	85.7	8.13 (s, 2H), 8.02 (s, 1H), 7.71 (s, 1H), 7.65 (s, 3H), 7.46 – 7.34 (m, 3H), 6.87 (d, J = 8.1 Hz, 1H), 6.82 (s, 1H), 6.67 (d, J = 2.6 Hz, 1H).	141.94, 139.94, 134.79, 133.23, 132.95, 132.74, 132.68, 132.48, 130.65, 130.19, 129.46, 127.92, 127.81, 127.57, 126.44, 123.75, 121.25, 120.30, 118.17, 111.21, 104.84.	$C_{23}H_{14}F_9N_2O_2S^+$	553.06	552.45	99.8

	Compound ID	Yield (%)	^{1}H NMR (500 MHz, $CDCl_3$)	^{13}C NMR (126 MHz, $CDCl_3$)	Chemical formula $[M+H]^+$	Calc. mass $[M+H]^+$	Found mass $[M+H]^+$	HPLC purity (%)
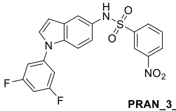 PRAN_3_1	PRAN_3_1	27.8	8.63 (s, 1H), 8.36 (d, J = 8.1 Hz, 1H), 8.03 (d, J = 7.7 Hz, 1H), 7.63 (t, J = 8.0 Hz, 1H), 7.50 – 7.38 (m, 2H), 7.29 (t, J = 12.7 Hz, 1H), 7.05 (s, 1H), 7.00 (d, J = 5.9 Hz, 2H), 6.96 (d, J = 8.7 Hz, 1H), 6.81 (t, J = 8.7 Hz, 1H), 6.62 (d, J = 2.9 Hz, 1H).	164.71, 162.61, 148.32, 141.52, 141.31, 134.17, 133.06, 130.44, 129.10, 128.62, 127.44, 122.72, 119.98, 117.38, 111.36, 107.40, 107.24, 105.20, 102.51, 102.31, 102.16.	$C_{20}H_{14}F_2N_3O_4S^+$	430.07	429.65	95.0
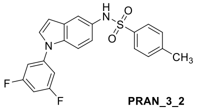 PRAN_3_2	PRAN_3_2	53.0	7.68 (d, J = 8.0 Hz, 2H), 7.49 – 7.39 (m, 2H), 7.28 (d, J = 3.0 Hz, 1H), 7.21 (d, J = 7.9 Hz, 2H), 7.08 (s, 1H), 6.99 (dd, J = 14.1, 8.0 Hz,3H), 6.80 (t, J = 8.7 Hz, 1H), 6.62 (d, J = 2.8 Hz, 1H), 2.37 (s, 3H).	164.67, 162.69, 143.70, 141.74, 136.31, 133.68, 130.17, 130.03, 129.68, 128.43, 127.50, 119.67, 116.51, 110.96, 107.23, 107.01, 105.22, 102.18, 101.98, 101.78, 21.63.	$C_{21}H_{17}F_2N_2O_2S^+$	399.10	398.60	97.6
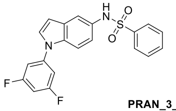 PRAN_3_3	PRAN_3_3	46.0	7.80 (d, J = 7.6 Hz, 2H), 7.52 (t, J = 7.3 Hz, 1H), 7.43 (d, J = 8.3 Hz, 4H), 7.29 (d, J = 2.9 Hz, 1H), 7.10 – 6.93 (m, 4H), 6.81 (t, J = 8.7 Hz, 1H), 6.62 (d, J = 2.6 Hz, 1H).	164.60, 162.61, 141.65, 139.33, 133.83, 132.91, 130.21, 129.84, 129.06, 128.52, 127.49, 119.85, 116.79, 111.00, 107.29, 107.07, 105.24, 102.24, 102.04, 101.84.	$C_{20}H_{15}F_2N_2O_2S^+$	385.08	384.60	97.5
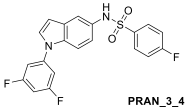 PRAN_3_4	PRAN_3_4	52.2	7.83 – 7.74 (m, 2H), 7.45 (d, J = 9.7 Hz, 2H), 7.30 (t, J = 9.4 Hz, 1H),7.09 (t, J = 8.4 Hz, 2H), 7.04 – 6.98 (m, 3H), 6.96 (d, J = 8.7 Hz, 1H),6.81 (t, J = 8.6 Hz, 1H), 6.63 (s, 1H).	166.31, 164.62, 164.29, 162.64, 141.69, 135.29, 133.95, 130.21, 129.57, 128.68, 119.92, 116.97, 116.41, 116.23, 111.12, 107.31, 107.14, 105.23, 102.36, 102.15, 101.95.	$C_{20}H_{14}F_3N_2O_2S^+$	403.07	402.60	97.2
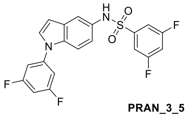 PRAN_3_5	PRAN_3_5	48.0	7.51 – 7.37 (m, 2H), 7.29 (t, J = 14.3 Hz, 3H), 7.02 (d, J = 6.3 Hz, 2H), 6.95 (d, J = 8.6 Hz, 2H), 6.86 (s, 1H), 6.82 (t, J = 8.5 Hz, 1H), 6.64 (s, 1H).	164.62, 163.75, 162.69, 161.72, 142.52, 141.59, 134.18, 130.36, 128.90, 119.99, 117.37, 111.28, 111.01, 108.84, 108.64, 108.44, 107.48, 107.26, 105.23, 102.49, 102.29, 102.08.	$C_{20}H_{13}F_4N_2O_2S^+$	421.06	420.60	97.4
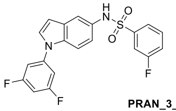 PRAN_3_6	PRAN_3_6	38.5	7.55 (d, J = 7.5 Hz, 1H), 7.50 – 7.38 (m, 4H), 7.30 (d, J = 2.5 Hz, 1H), 7.22 (t, J = 7.0 Hz, 1H), 7.01 (d, J = 5.6 Hz, 2H), 6.94 (d, J = 8.3Hz, 1H), 6.81 (s, 2H), 6.63 (s, 1H).	164.76, 164.64, 163.45, 162.77, 162.66, 161.45, 141.40, 134.08, 130.89, 130.83, 130.27, 129.27, 128.75, 123.32, 120.27, 120.10, 120.00, 117.19, 115.02, 114.83, 111.16, 107.43, 107.20, 105.25, 102.30, 102.20, 102.00.	$C_{20}H_{14}F_3N_2O_2S^+$	403.07	402.75	97.2
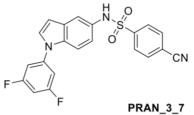 PRAN_3_7	PRAN_3_7	38.0	7.86 (d, J = 8.1 Hz, 2H), 7.70 (d, J = 8.1 Hz, 2H), 7.45 (d, J = 8.7 Hz, 1H), 7.41 (s, 1H), 7.32 (d, J = 2.8 Hz, 1H), 7.12 (s, 1H), 7.00 (d, J = 5.6 Hz, 2H), 6.93 (d, J = 8.6 Hz, 1H), 6.81 (t, J = 8.5 Hz, 1H), 6.63 (s, 1H).	164.63, 162.59, 143.33, 141.48, 134.08, 132.88, 130.21, 128.94, 128.73, 128.10, 119.91, 117.42, 117.23, 116.61, 111.29, 107.35, 107.18, 105.17, 102.50, 102.30, 102.10.	$C_{21}H_{14}F_2N_3O_2S^+$	410.08	410.15	97.6
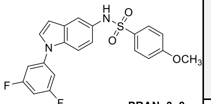 PRAN_3_8	PRAN_3_8	50.2	7.70 (d, J = 8.6 Hz, 2H), 7.52 – 7.37 (m, 2H), 7.33 – 7.27 (m, 1H), 7.04 (d, J = 6.5 Hz, 2H), 6.96 (d, J = 8.7 Hz, 1H), 6.89 (d, J = 8.6 Hz, 2H), 6.82 (t, J = 8.7 Hz, 1H), 6.64 (d, J = 2.6 Hz, 2H), 3.84 (s, 3H).	164.75, 163.13, 133.81, 131.08, 131.02, 130.23, 130.03, 129.64, 128.52, 119.94, 116.83, 114.24, 111.01, 107.33, 107.11, 105.26, 102.29, 102.08, 101.88, 55.69.	$C_{21}H_{17}F_2N_2O_3S^+$	415.09	414.60	97.7
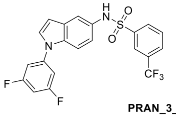 PRAN_3_9	PRAN_3_9	38.0	8.00 (s, 1H), 7.87 (d, J = 7.7 Hz, 1H), 7.79 (d, J = 7.6 Hz, 1H), 7.56 (t, J = 7.7 Hz, 1H), 7.46 (d, J = 8.7 Hz, 1H), 7.41 (s, 1H), 7.31 (s, 1H), 7.02 (d, J = 6.0 Hz, 2H), 6.89 (d, J = 8.6 Hz, 1H), 6.82 (t, J = 8.7 Hz, 1H), 6.64 (s, 1H), 6.52 (s, 1H).	164.64, 154.11, 153.61, 135.38, 134.20, 133.42, 130.71, 130.28, 129.82, 129.58, 128.91, 124.61, 120.28, 117.71, 111.24, 107.48, 107.25, 105.22, 102.51, 102.31, 102.11.	$C_{21}H_{14}F_5N_2O_2S^+$	453.07	452.60	98.4
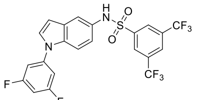 PRAN_3_10	PRAN_3_10	41.3	8.14 (s, 2H), 8.02 (s, 1H), 7.47 (d, J = 8.7 Hz, 1H), 7.42 (s, 1H), 7.33(s, 1H), 7.01 (d, J = 6.0 Hz, 2H), 6.90 (d, J = 7.3 Hz, 2H), 6.82 (t, J =8.6 Hz, 1H), 6.65 (s, 1H).	164.75, 162.65, 141.91, 141.52, 134.41, 133.21, 132.96, 132.69, 132.41, 130.37, 129.16, 128.28, 127.79, 126.46, 123.60, 121.42, 120.31, 118.05, 111.42, 107.54, 107.31, 105.19, 102.75, 102.41, 102.21.	$C_{22}H_{13}F_8N_2O_2S^+$	521.06	520.50	96.8

LCMS and NMR spectra are provided in Supporting File 2 (PDF, 120 pages)
