# Supplementary material for: Structure-Based Discovery of Receptor Activator of Nuclear Factor-κB Ligand (RANKL)-Induced Osteoclastogenesis Inhibitors
Source: Int J Mol Sci. 2023 Jul 10;24(14):11290. doi: 10.3390/ijms241411290 (PMC10379842; doi:10.3390/ijms241411290)
Supplement: Supplementary file 1 [file ijms-24-11290-s001.zip › SupportingFile_2_Spectra.pdf]

# ==== Shimadzu LCMSsolution Analysis Report ====

## Sample Name : PRAN-1.1

Method

Column: Purospher RP-8
Mobile Phase A: $H_2O$ + 0.9% acetic acid
Mobile Phase B: ACN
% Pump B Concetrate: 75.0
Flow (ml/min): 0.6000

Detector A:SPD-20A
UV_1.Wavelength: 225
UV_2.Wavelength: 254
LC Program

| Time | Unit | Command | Value |
|---|---|---|---|
| 20.00 | Controller | Stop | |

MS Chromatogram

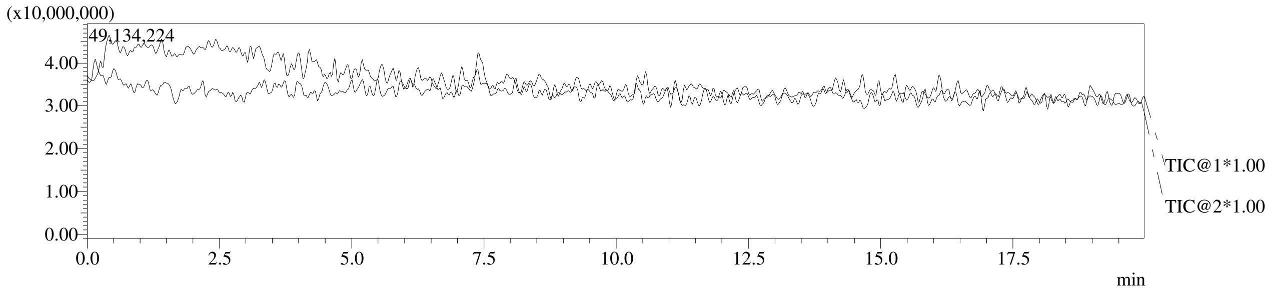


## <LC-UV Chromatogram>

Chromatogram

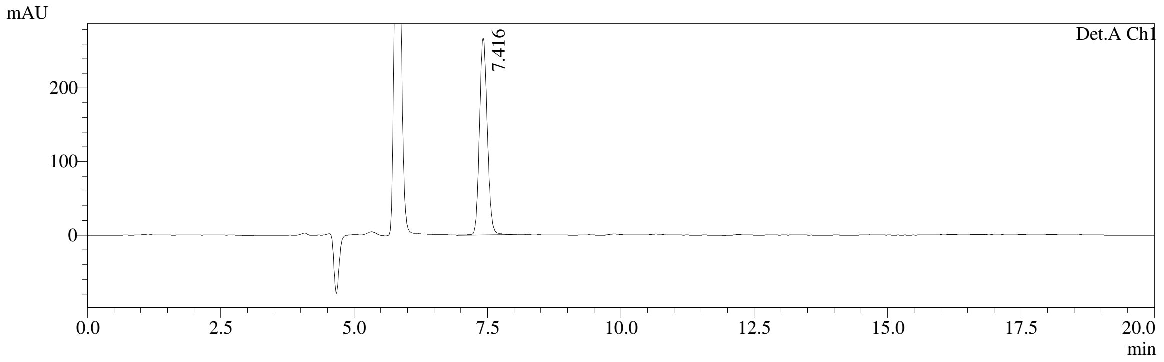

## Sample Name : PRAN-1.1

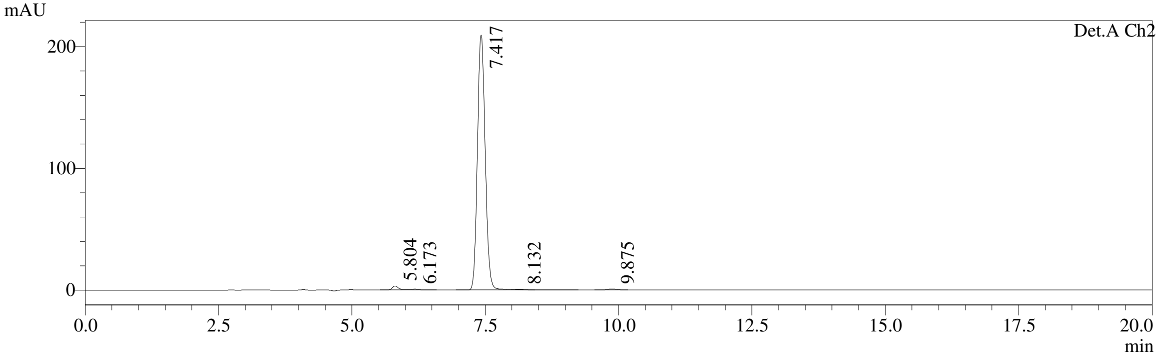


1 Det.A Ch1 / 225nm
2 Det.A Ch2 / 254nm

PeakTable

Detector A Ch2 254nm

| Peak# | Ret. Time | Area | Height | Area % | Height % |
|---|---|---|---|---|---|
| 1 | 5.804 | 28149 | 3248 | 1.288 | 1.511 |
| 2 | 6.173 | 7967 | 820 | 0.365 | 0.381 |
| 3 | 7.417 | 2131293 | 209437 | 97.531 | 97.399 |
| 4 | 8.132 | 5740 | 535 | 0.263 | 0.249 |
| 5 | 9.875 | 12098 | 991 | 0.554 | 0.461 |
| Total | | 2185247 | 215031 | 100.000 | 100.000 |

MS Spectrum Graph

#:1 Ret.Time:Averaged 7.193-7.605(Scan#:665-703)
BG Mode:Averaged 4.485-5.235(415-485)
Mass Peaks:506 Base Peak:225.70(2013318) Polarity:Pos Segment1 - Event1

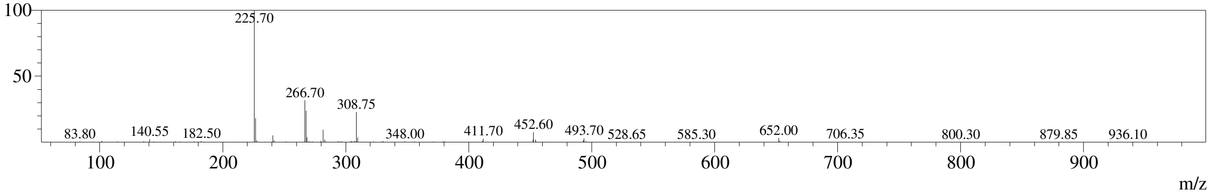


#:2 Ret.Time:Averaged 7.204-7.616(Scan#:666-704)
BG Mode:Averaged 4.496-5.235(416-486)
Mass Peaks:488 Base Peak:409.60(706214) Polarity:Neg Segment1 - Event2

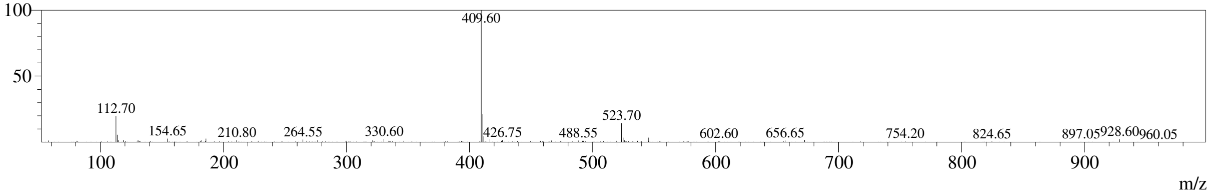

$^1$H NMR
500MHz ($CDCl_3$)
PRAN-1.1

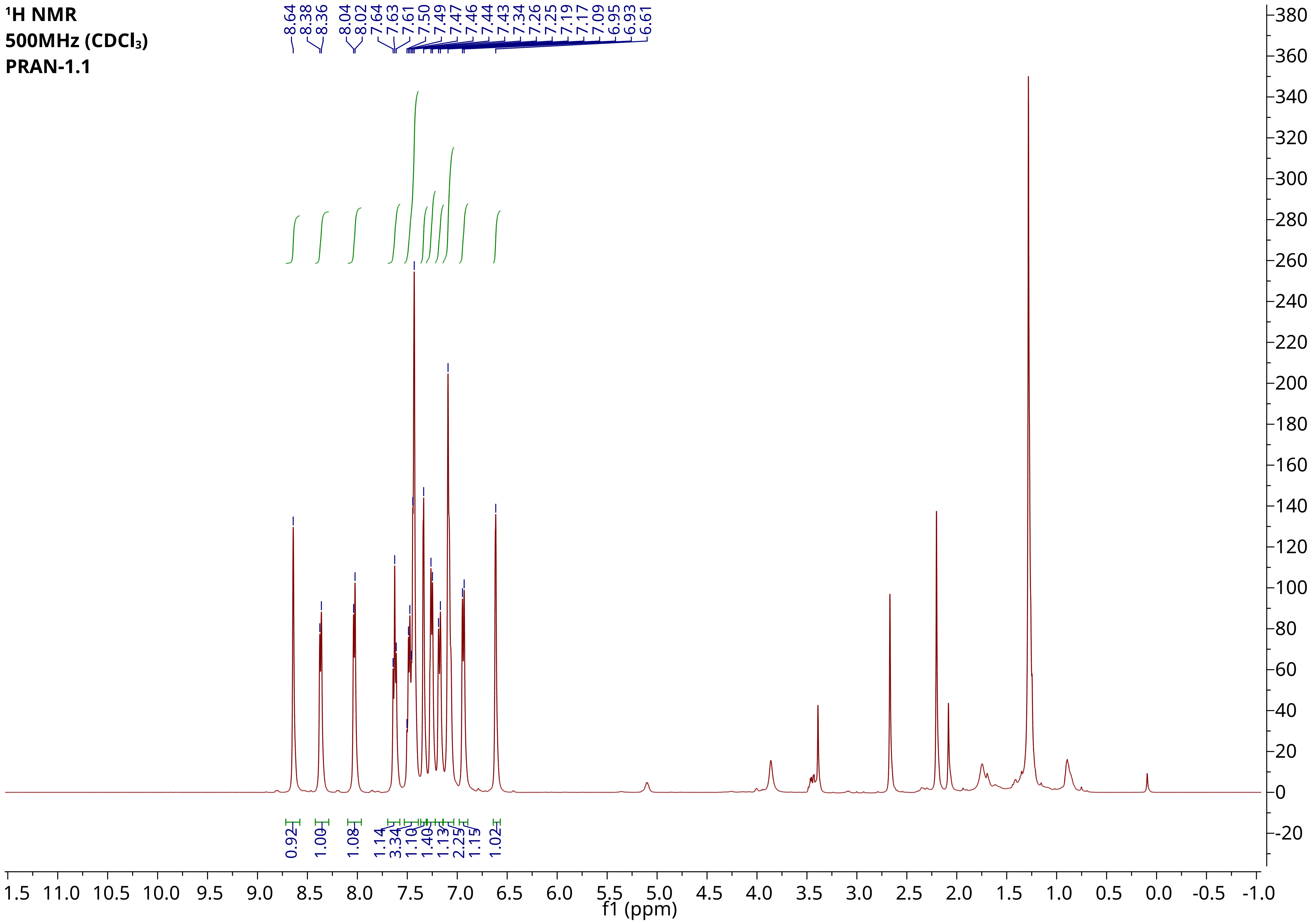

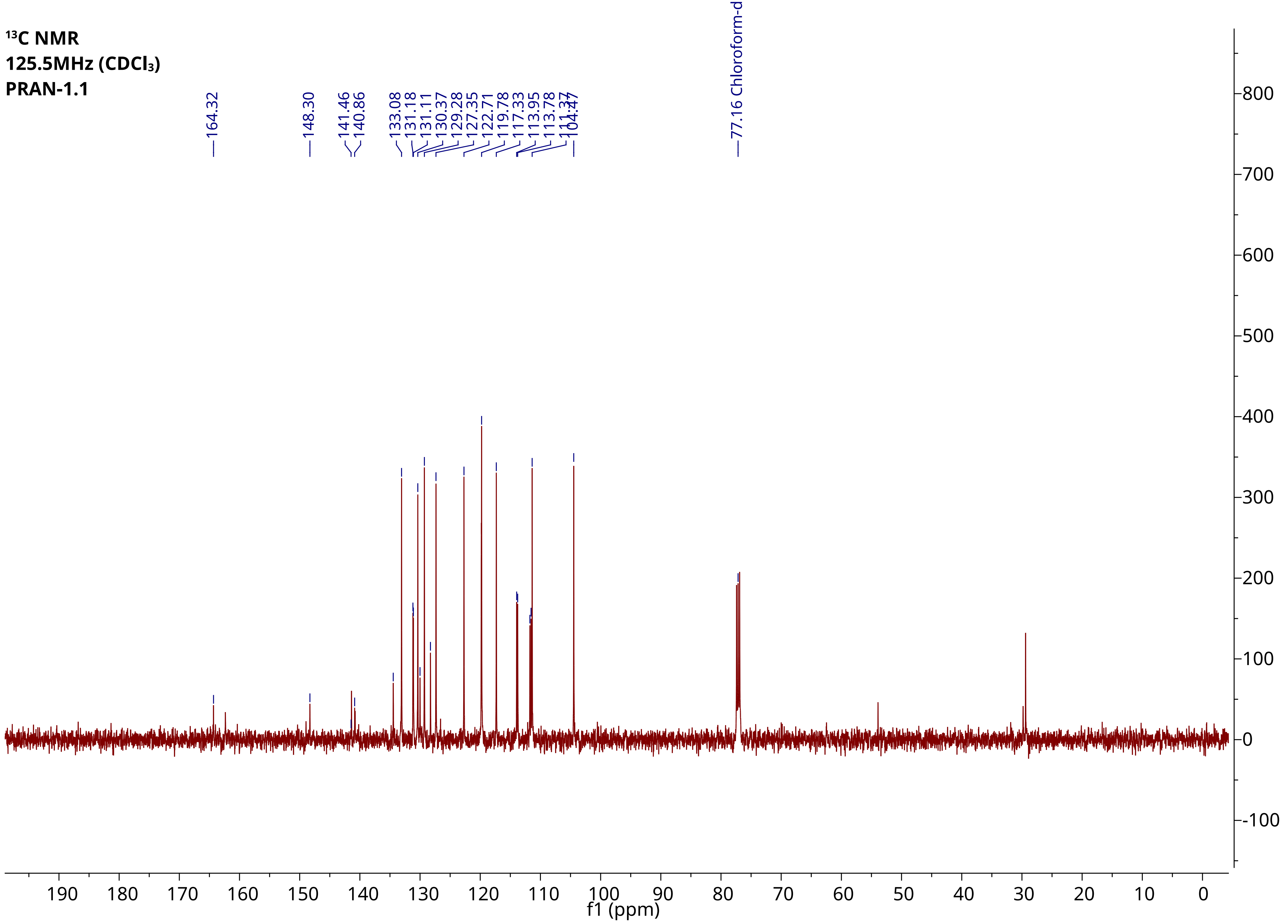

13C NMR
125.5MHz (CDCl3)
PRAN-1.1
164.32
148.30
141.46
140.86
133.08
131.18
131.11
130.37
129.28
127.35
122.71
119.78
117.33
113.95
113.78
111.37
104.47
77.16 Chloroform-d
f1 (ppm)

# ==== Shimadzu LCMSsolution Analysis Report ====

**Sample Name : PRAN-1.2**

Method

Column: Purospher RP-8
Mobile Phase A: H2O + 0.9% acetic acid
Mobile Phase B: ACN
% Pump B Concetrate: 75.0
Flow (ml/min): 0.6000

Detector A:SPD-20A
UV_1.Wavelength: 225
UV_2.Wavelength: 254
LC Program

| Time | Unit | Command | Value |
|---|---|---|---|
| 0.01 | Pumps | B.Conc | 26 |
| 7.00 | Pumps | B.Conc | 60 |
| 12.00 | Pumps | B.Conc | 60 |
| 20.00 | Pumps | B.Conc | 95 |
| 25.00 | Pumps | B.Conc | 95 |
| 25.01 | Pumps | B.Conc | 26 |
| 40.01 | Controller | Stop | |

MS Chromatogram

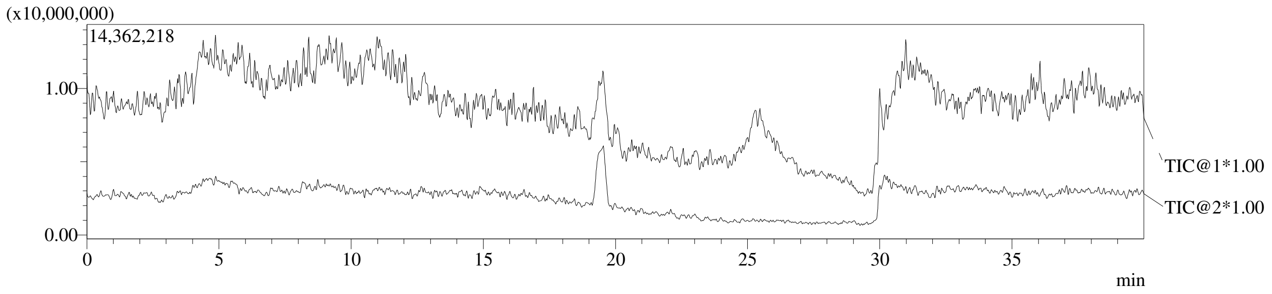


## <LC-UV Chromatogram>

Chromatogram

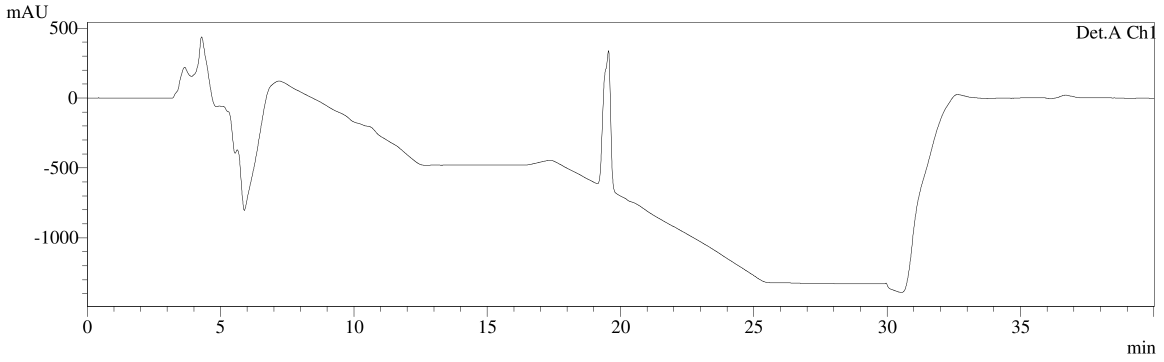

## Sample Name : PRAN-1.2

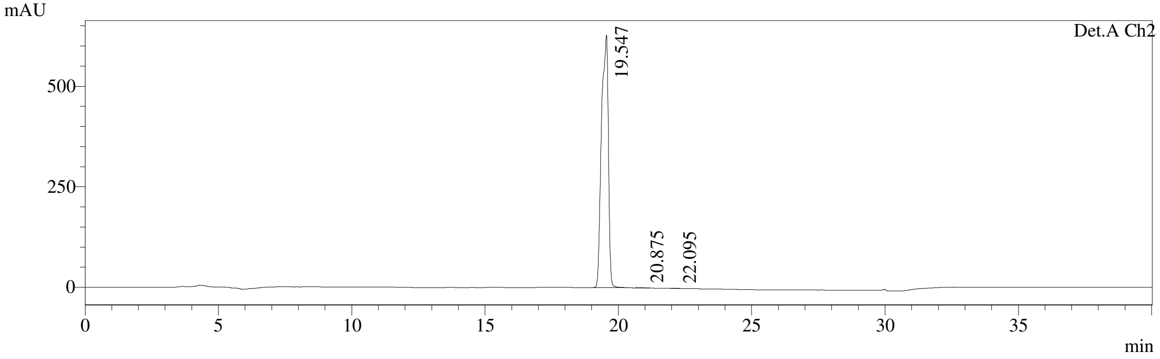


1 Det.A Ch1 / 225nm
2 Det.A Ch2 / 254nm

PeakTable

Detector A Ch2 254nm

| Peak# | Ret. Time | Area | Height | Area % | Height % |
|---|---|---|---|---|---|
| 1 | 19.547 | 11301293 | 628020 | 99.845 | 99.804 |
| 2 | 20.875 | 11450 | 767 | 0.101 | 0.122 |
| 3 | 22.095 | 6076 | 464 | 0.054 | 0.074 |
| Total | | 11318819 | 629251 | 100.000 | 100.000 |

MS Spectrum Graph

#:1 Ret.Time:Averaged 19.103-19.782(Scan#:2085-2159)
BG Mode:Averaged 25.630-27.760(2797-3029)
Mass Peaks:500 Base Peak:101.80(1220194) Polarity:Pos Segment1 - Event1

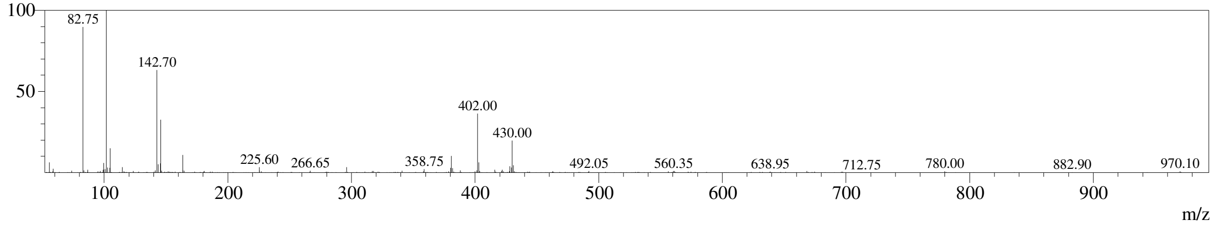


#:2 Ret.Time:Averaged 19.113-19.791(Scan#:2086-2160)
BG Mode:Averaged 25.639-27.760(2798-3030)
Mass Peaks:506 Base Peak:248.60(703611) Polarity:Neg Segment1 - Event2

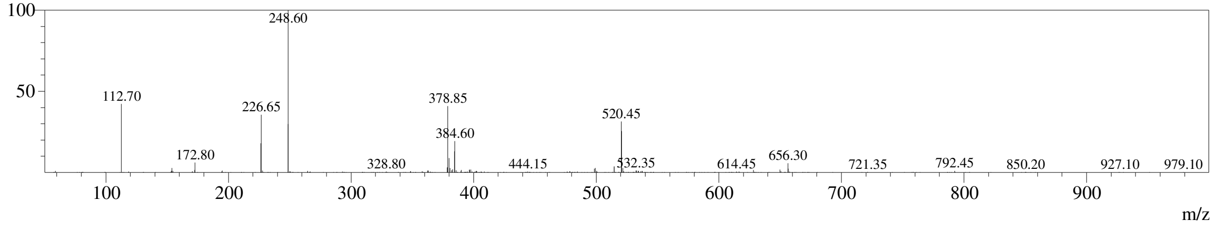

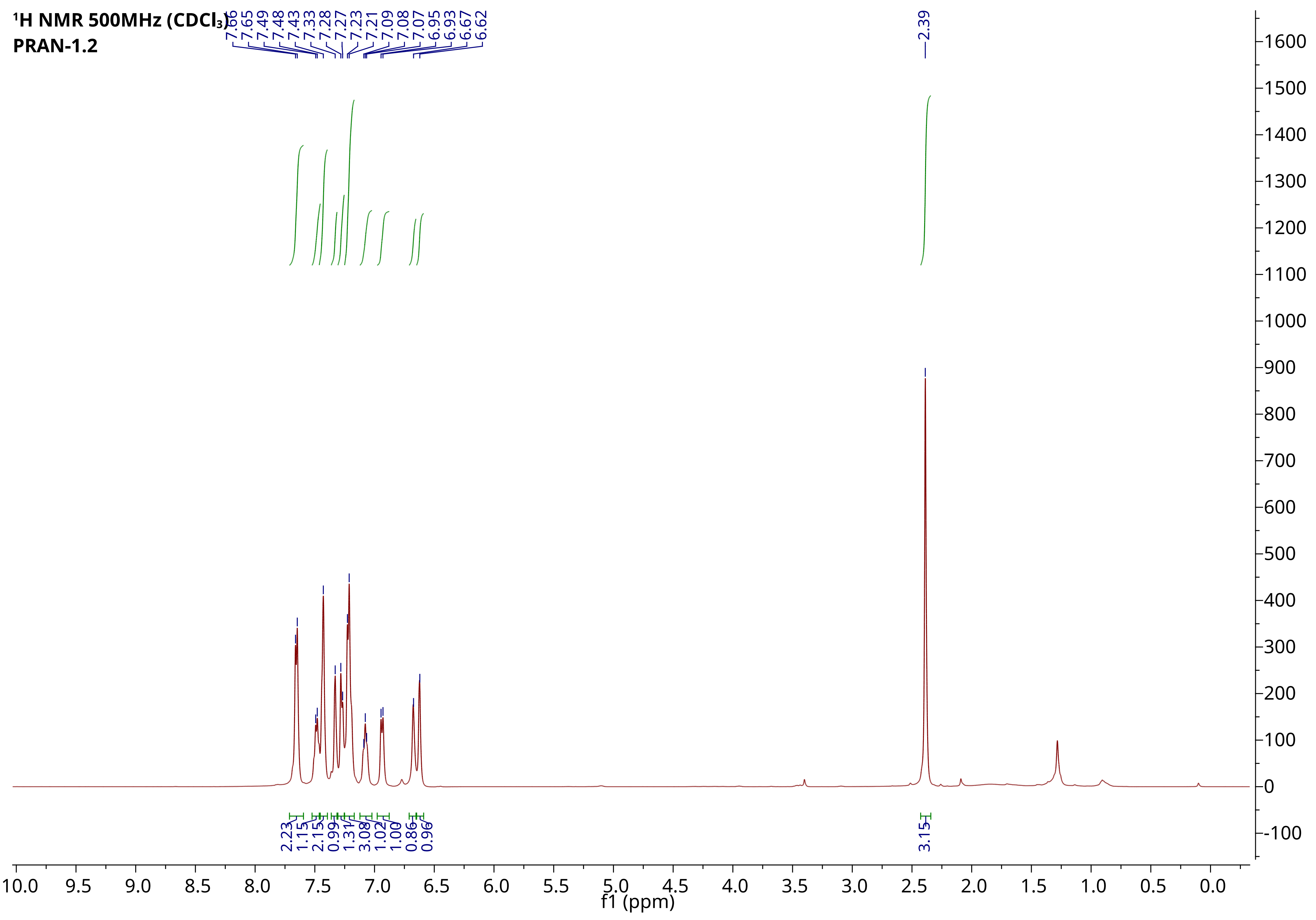

¹H NMR 500MHz (CDCl₃)
PRAN-1.2
7.66
7.65
7.49
7.48
7.43
7.33
7.28
7.27
7.23
7.21
7.09
7.08
7.07
6.95
6.93
6.67
6.62
2.39
2.23
1.15
2.15
0.99
1.31
3.08
1.02
1.00
0.86
0.96
3.15
f1 (ppm)

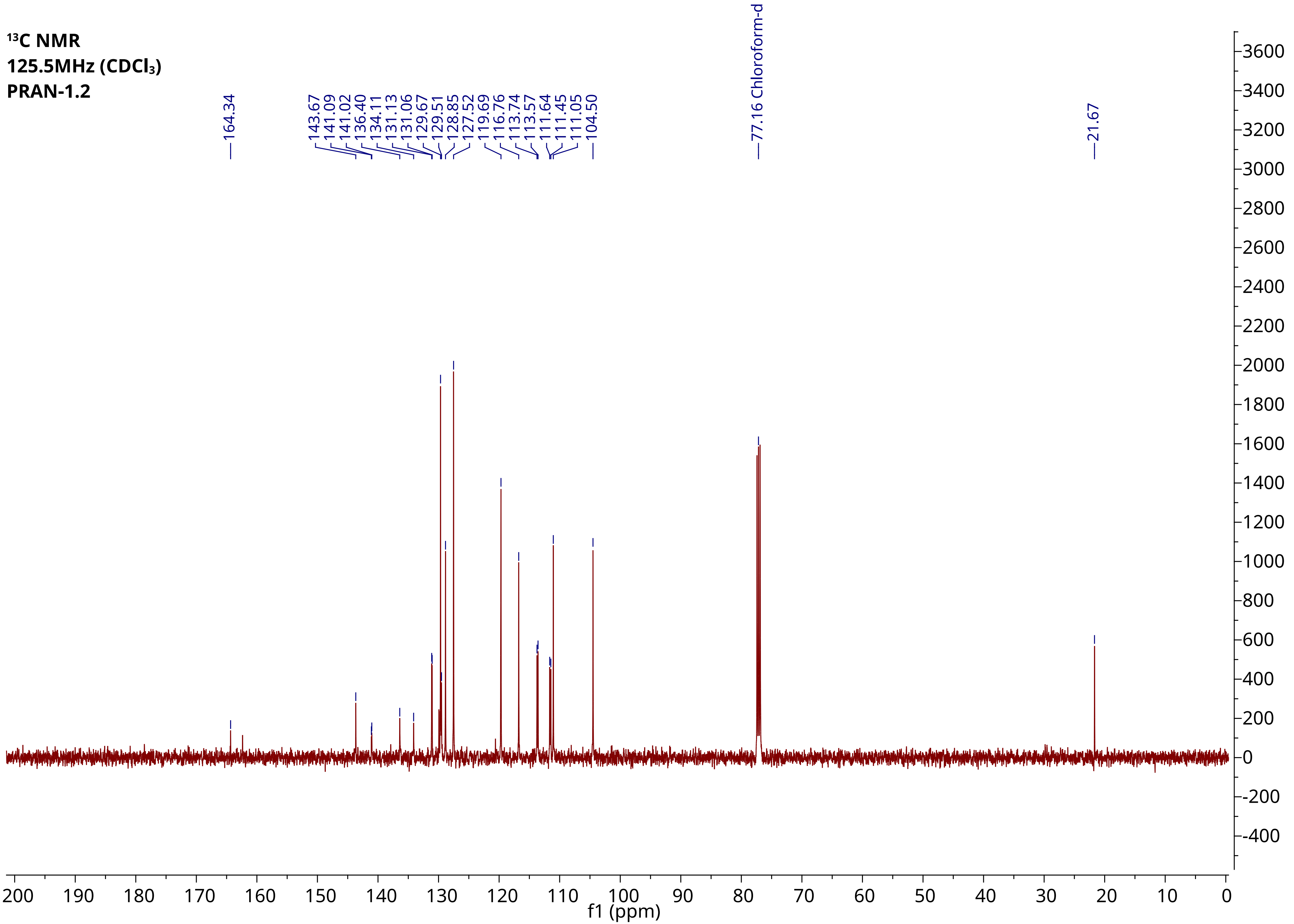

¹³C NMR
125.5MHz (CDCl₃)
PRAN-1.2
164.34
143.67
141.09
141.02
136.40
134.11
131.13
131.06
129.67
129.51
128.85
127.52
119.69
116.76
113.74
113.57
111.64
111.45
111.05
104.50
77.16 Chloroform-d
21.67
f1 (ppm)

# ==== Shimadzu LCMSsolution Analysis Report ====

## Sample Name : PRAN-1.3

Method

Column: Purospher RP-8
Mobile Phase A: H2O + 0.9% acetic acid
Mobile Phase B: ACN
% Pump B Concetrate: 75.0
Flow (ml/min): 0.6000

Detector A:SPD-20A
UV_1.Wavelength: 218
UV_2.Wavelength: 260
LC Program

| Time | Unit | Command | Value |
|---|---|---|---|
| 30.00 | Controller | Stop | |

MS Chromatogram

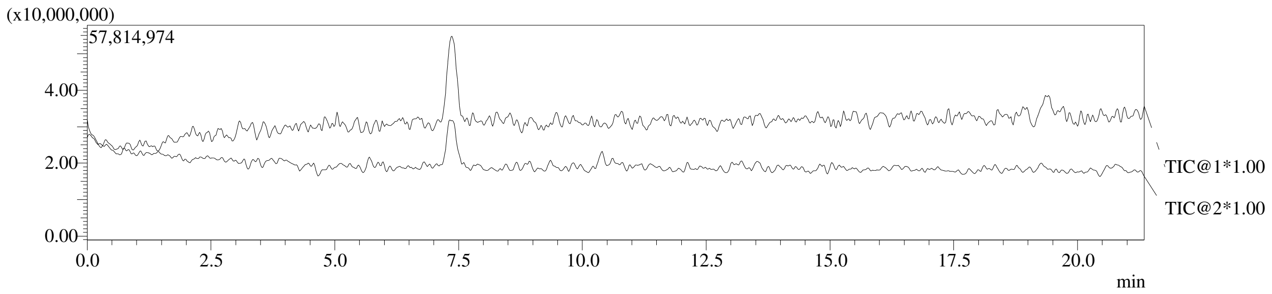


## <LC-UV Chromatogram>

Chromatogram

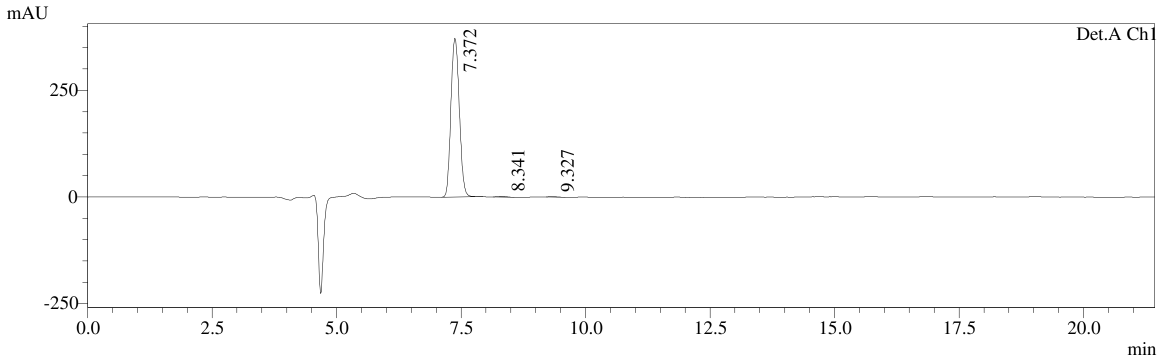

## Sample Name : PRAN-1.3

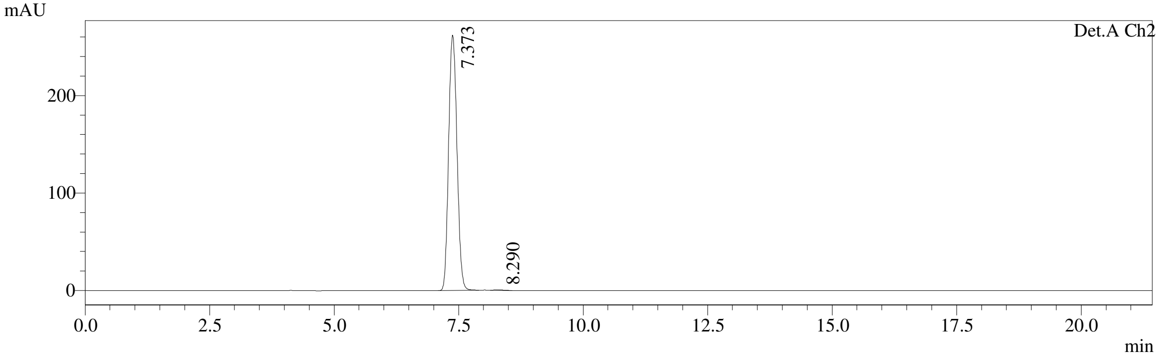


1 Det.A Ch1 / 218nm
2 Det.A Ch2 / 260nm

PeakTable

Detector A Ch2 260nm

| Peak# | Ret. Time | Area | Height | Area % | Height % |
|---|---|---|---|---|---|
| 1 | 7.373 | 3062367 | 261977 | 99.834 | 99.832 |
| 2 | 8.290 | 5107 | 440 | 0.166 | 0.168 |
| Total | | 3067475 | 262417 | 100.000 | 100.000 |

MS Spectrum Graph

#:1 Ret.Time:Averaged 7.258-7.323(Scan#:671-677)
BG Mode:Averaged 11.267-12.768(1041-1179)
Mass Peaks:387 Base Peak:225.70(7789529) Polarity:Pos Segment1 - Event1

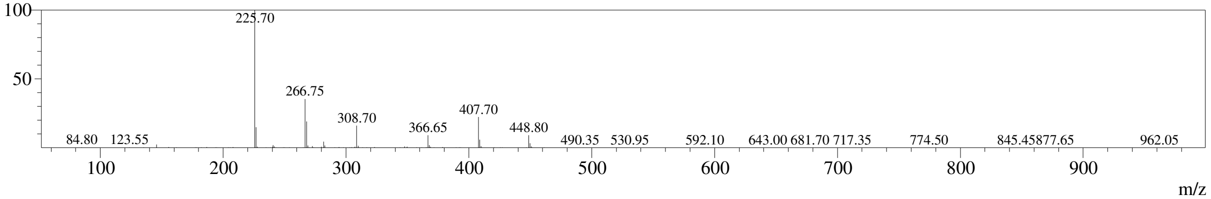


#:2 Ret.Time:Averaged 7.269-7.334(Scan#:672-678)
BG Mode:Averaged 11.278-12.768(1042-1180)
Mass Peaks:384 Base Peak:364.65(8479462) Polarity:Neg Segment1 - Event2

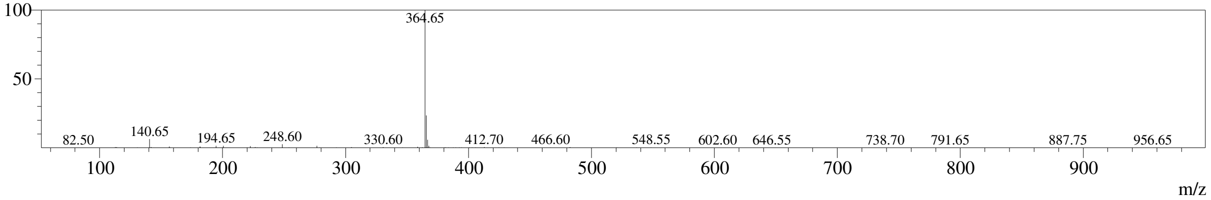

¹H NMR 500MHz ($CDCl_3$)
PRAN-1.3

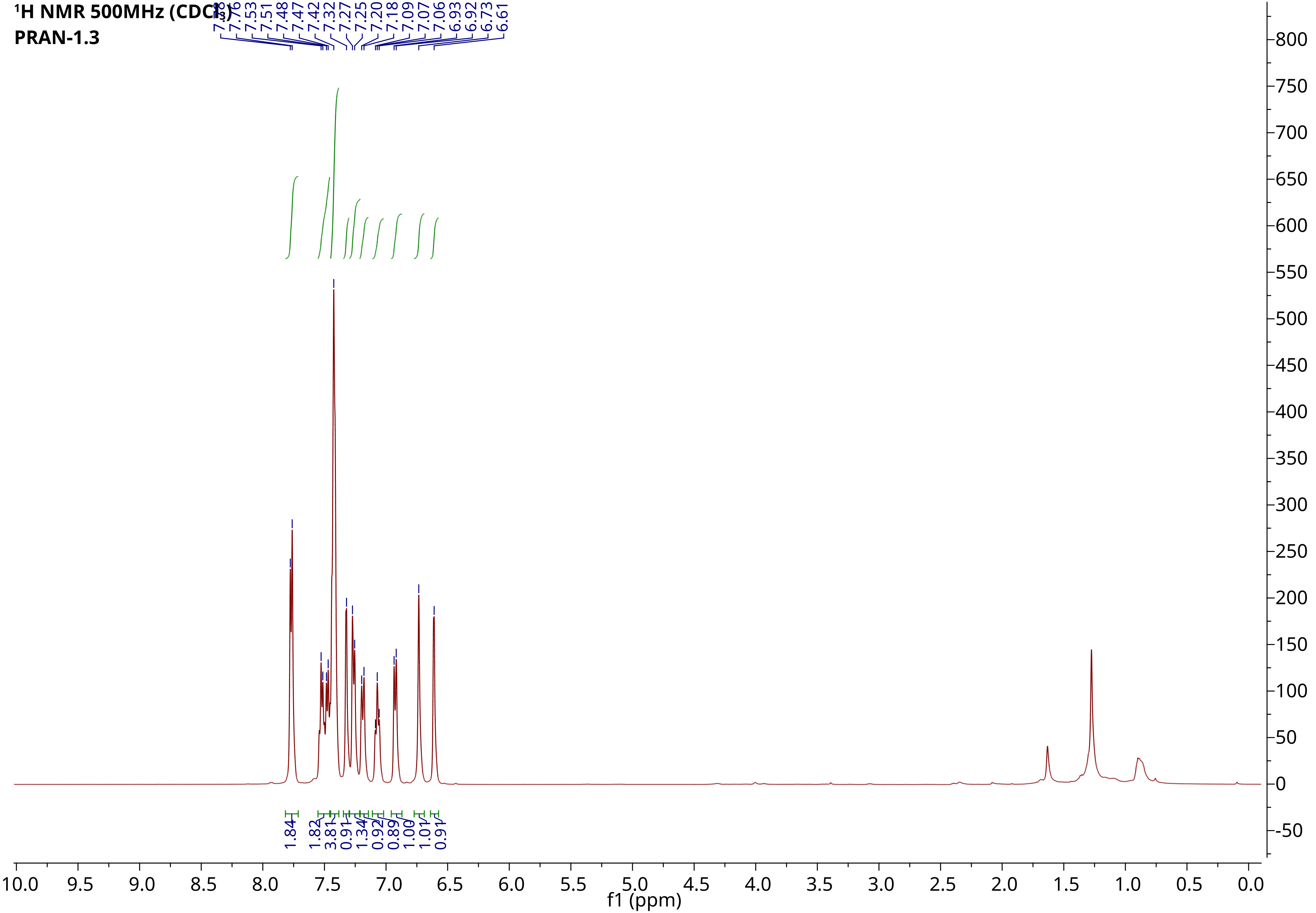

**$^{13}C$ NMR**
**125.5MHz ($CDCl_3$)**
**PRAN-1.3**

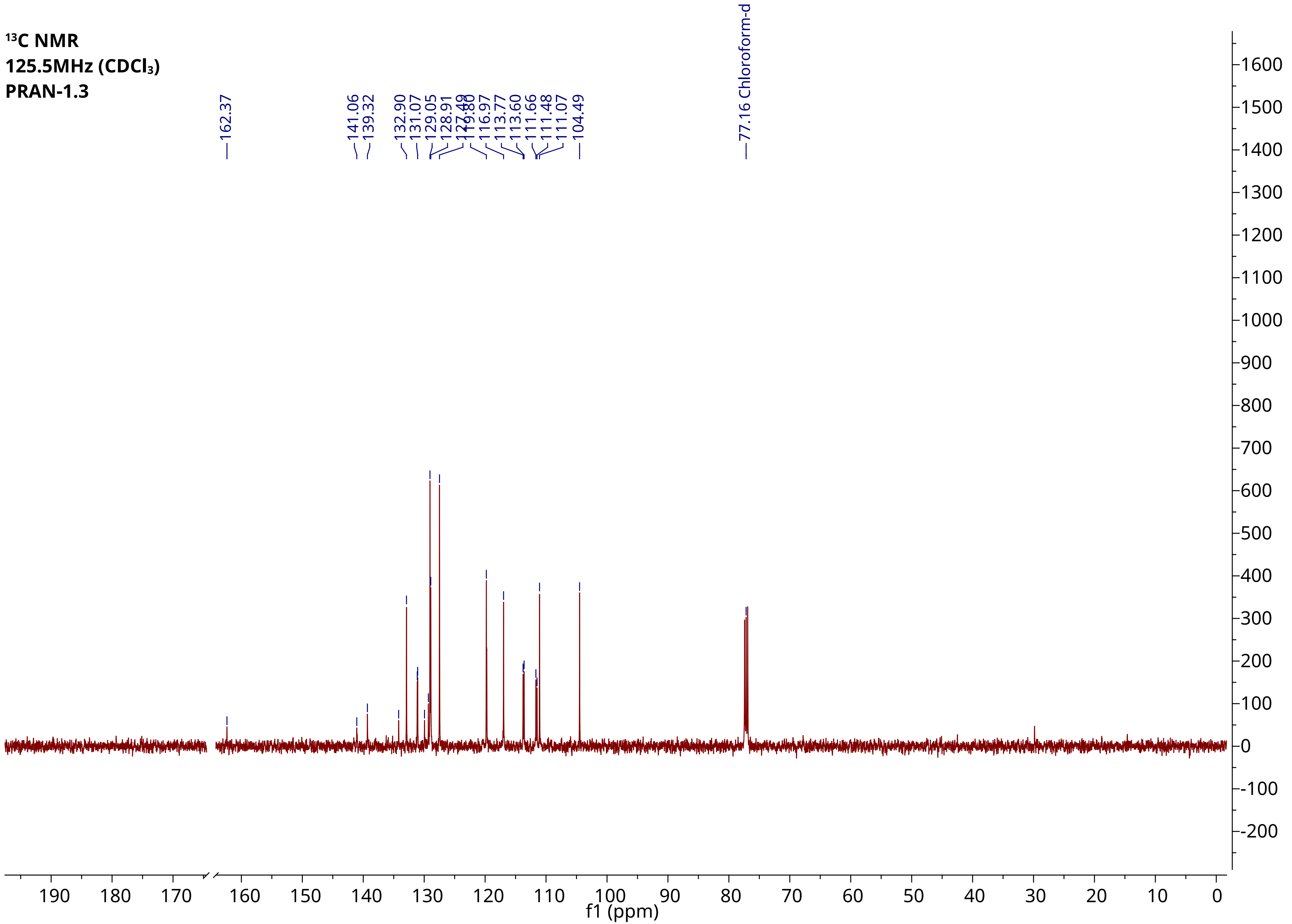

# ==== Shimadzu LCMSsolution Analysis Report ====

**Sample Name : PRAN-1.4**

Method

Column: Shim Pack - XR-ODS
Mobile Phase A: H2O + 0.9% acetic acid
Mobile Phase B: ACN
% Pump B Concetrate: 10.0
Flow (ml/min): 0.6000

Detector A:SPD-20A
UV_1.Wavelength: 217
UV_2.Wavelength: 254
LC Program

| Time | Unit | Command | Value |
|---|---|---|---|
| 0.01 | Pumps | B.Conc | 10 |
| 15.00 | Pumps | B.Conc | 90 |
| 30.00 | Pumps | B.Conc | 90 |
| 30.01 | Pumps | B.Conc | 10 |
| 40.00 | Controller | Stop | |

MS Chromatogram

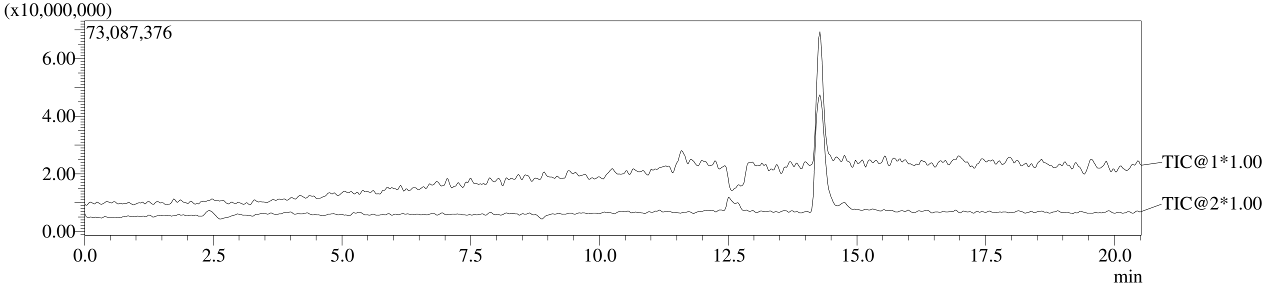


## <LC-UV Chromatogram>

Chromatogram

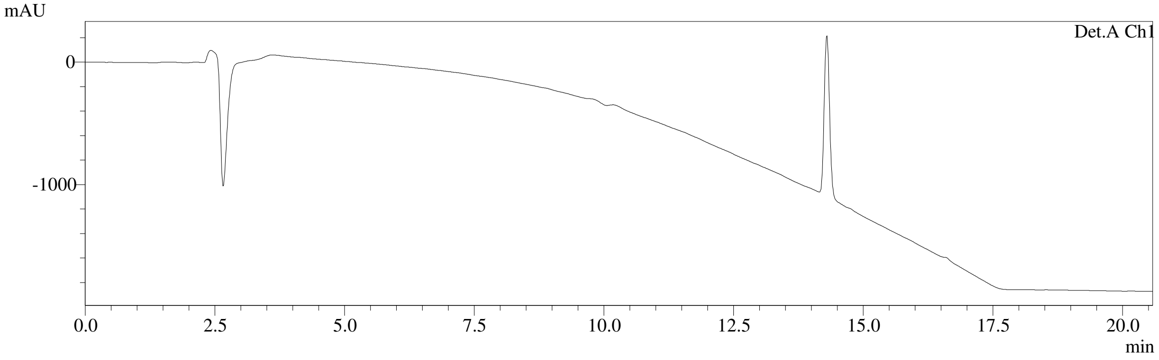

# Sample Name : PRAN-1.4

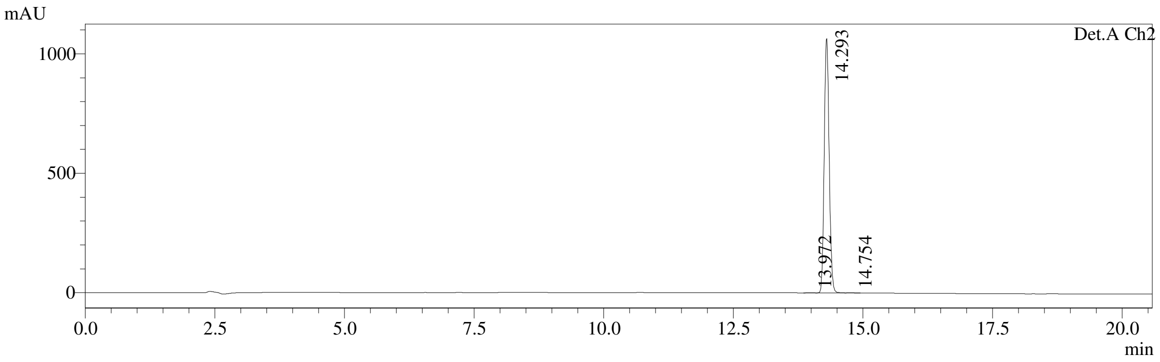


1 Det.A Ch1 / 217nm
2 Det.A Ch2 / 254nm

PeakTable

Detector A Ch2 254nm

| Peak# | Ret. Time | Area | Height | Area % | Height % |
|---|---|---|---|---|---|
| 1 | 13.972 | 10773 | 1534 | 0.150 | 0.144 |
| 2 | 14.293 | 7155068 | 1065207 | 99.679 | 99.703 |
| 3 | 14.754 | 12260 | 1639 | 0.171 | 0.153 |
| Total | | 7178101 | 1068380 | 100.000 | 100.000 |

MS Spectrum Graph

#:1 Ret.Time:Averaged 14.192-14.408(Scan#:1311-1331)
BG Mode:Averaged 15.752-16.202(1455-1497)
Mass Peaks:541 Base Peak:225.70(11061417) Polarity:Pos Segment1 - Event1

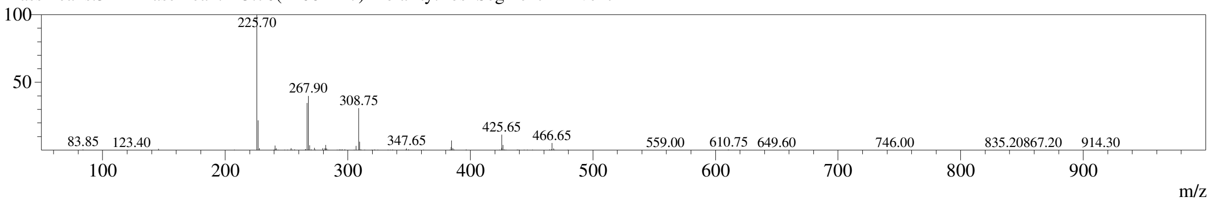


#:2 Ret.Time:Averaged 14.203-14.419(Scan#:1312-1332)
BG Mode:Averaged 15.763-16.202(1456-1498)
Mass Peaks:537 Base Peak:382.60(11569978) Polarity:Neg Segment1 - Event2

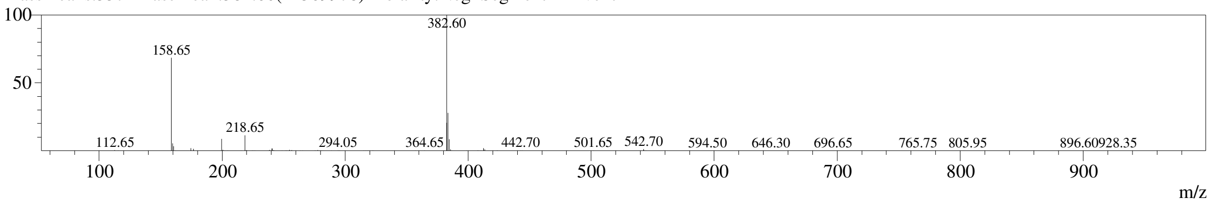

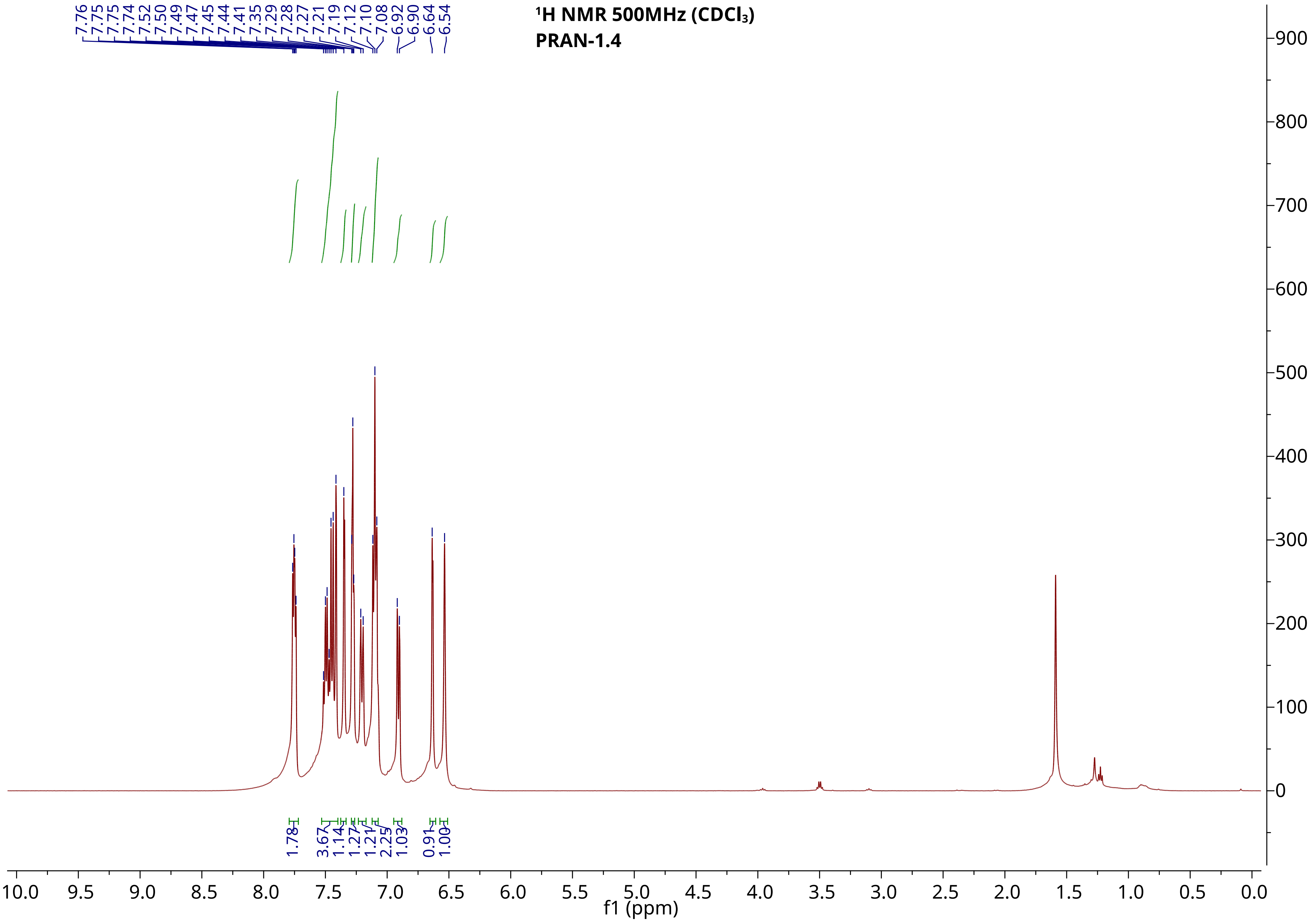

$^{1}$H NMR 500MHz (CDCl3)
PRAN-1.4
7.76
7.75
7.75
7.74
7.52
7.50
7.49
7.47
7.45
7.44
7.41
7.35
7.29
7.28
7.27
7.21
7.19
7.12
7.10
7.08
6.92
6.90
6.64
6.54
1.78
3.67
1.14
1.27
1.21
2.25
1.03
0.91
1.00
f1 (ppm)

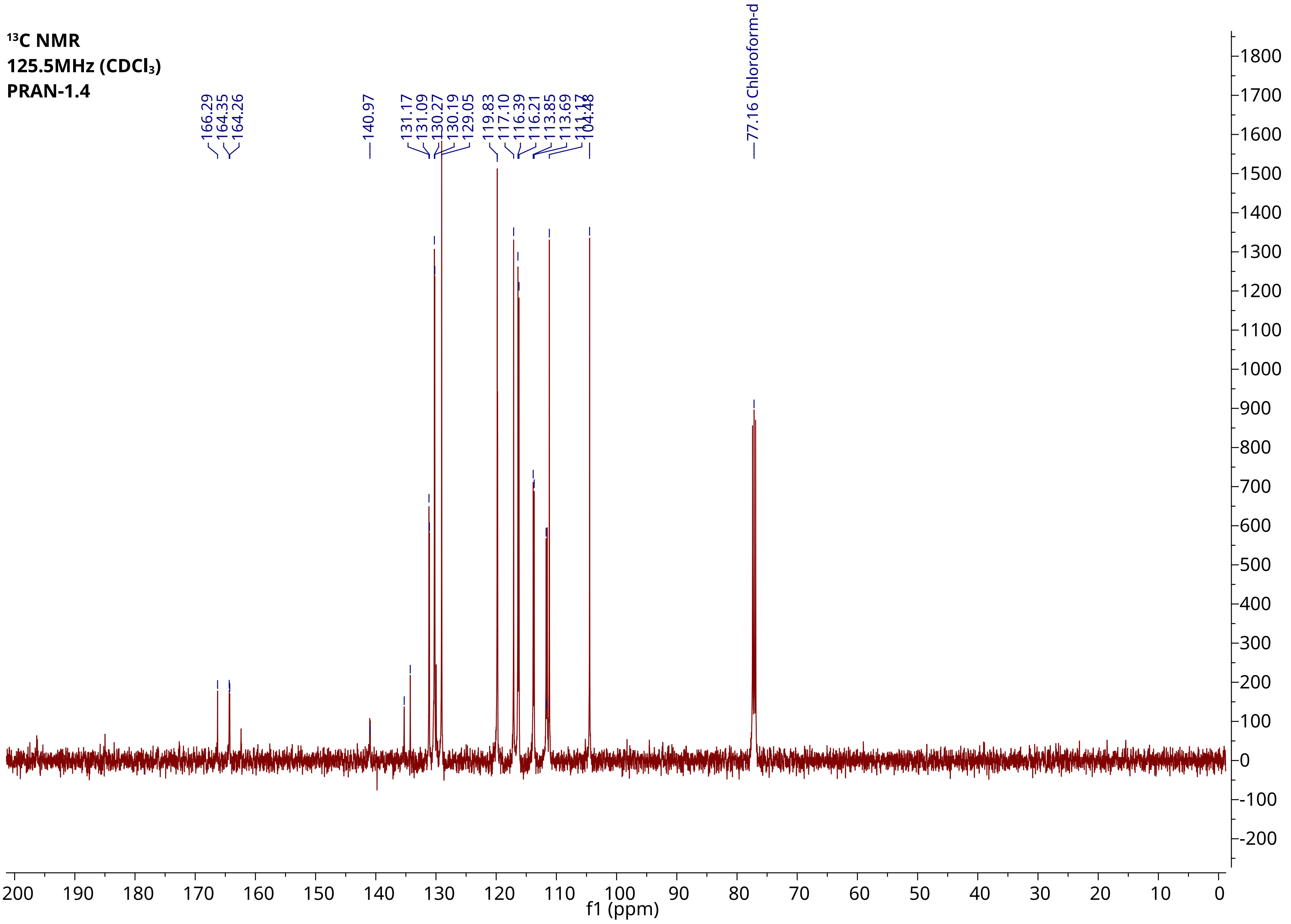

13C NMR
125.5MHz (CDCl3)
PRAN-1.4
166.29
164.35
164.26
140.97
131.17
131.09
130.27
130.19
129.05
119.83
117.10
116.39
116.21
113.85
113.69
111.17
104.48
77.16 Chloroform-d
f1 (ppm)

# ==== Shimadzu LCMSsolution Analysis Report ====

**Sample Name : PRAN-1.5**

Method

Column: Shim Pack - XR-ODS
Mobile Phase A: $H_2O$ + 0.9% acetic acid
Mobile Phase B: ACN
% Pump B Concetrate: 10.0
Flow (ml/min): 0.6000

Detector A:SPD-20A
UV_1.Wavelength: 217
UV_2.Wavelength: 254
LC Program

| Time | Unit | Command | Value |
|---|---|---|---|
| 0.01 | Pumps | B.Conc | 10 |
| 15.00 | Pumps | B.Conc | 90 |
| 30.00 | Pumps | B.Conc | 90 |
| 30.01 | Pumps | B.Conc | 10 |
| 40.00 | Controller | Stop | |

MS Chromatogram

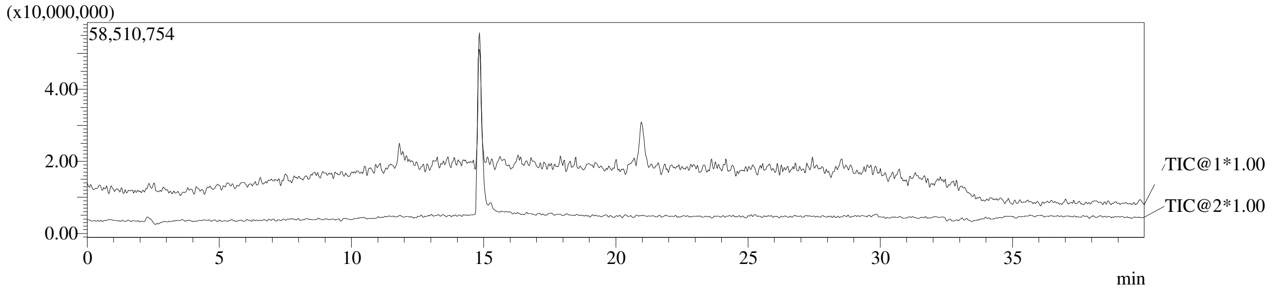


## <LC-UV Chromatogram>

Chromatogram

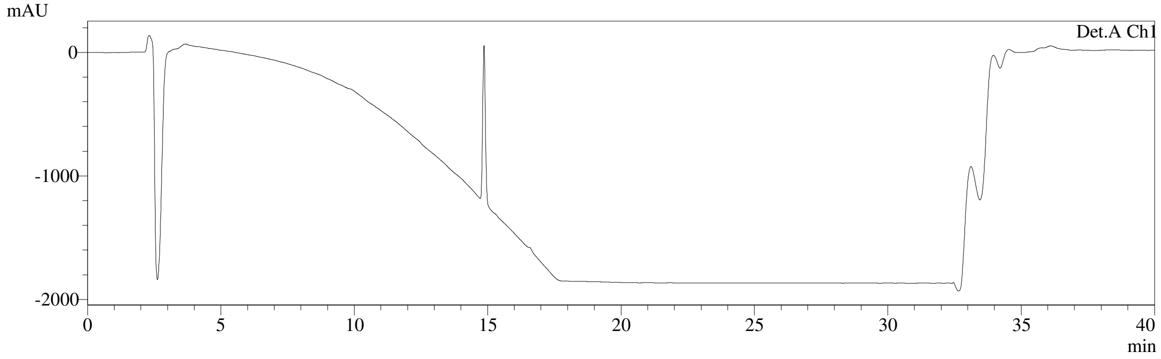

## Sample Name : PRAN-1.5

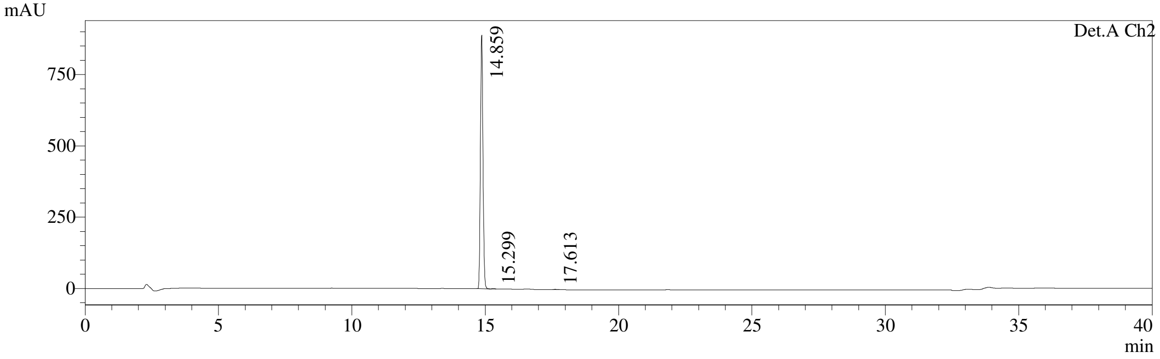


1 Det.A Ch1 / 217nm
2 Det.A Ch2 / 254nm

PeakTable

Detector A Ch2 254nm

| Peak# | Ret. Time | Area | Height | Area % | Height % |
|---|---|---|---|---|---|
| 1 | 14.859 | 5936950 | 889448 | 99.786 | 99.783 |
| 2 | 15.299 | 6680 | 1116 | 0.112 | 0.125 |
| 3 | 17.613 | 6036 | 815 | 0.101 | 0.091 |
| Total | | 5949666 | 891379 | 100.000 | 100.000 |

MS Spectrum Graph

#:1 Ret.Time:Averaged 14.603-15.015(Scan#:1349-1387)
BG Mode:Averaged 17.225-18.609(1591-1719)
Mass Peaks:439 Base Peak:225.70(4812477) Polarity:Pos Segment1 - Event1

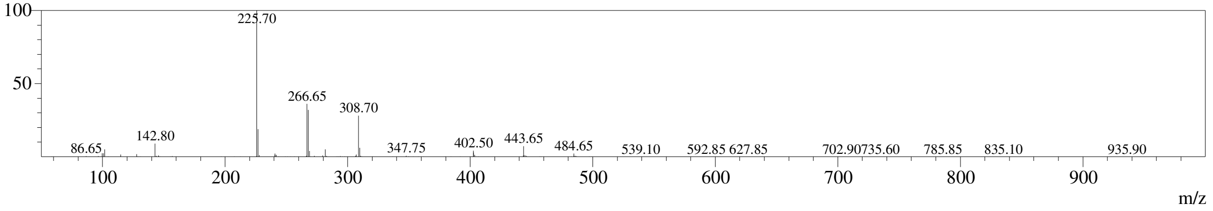


#:2 Ret.Time:Averaged 14.614-15.026(Scan#:1350-1388)
BG Mode:Averaged 17.236-18.609(1592-1720)
Mass Peaks:498 Base Peak:400.85(8293359) Polarity:Neg Segment1 - Event2

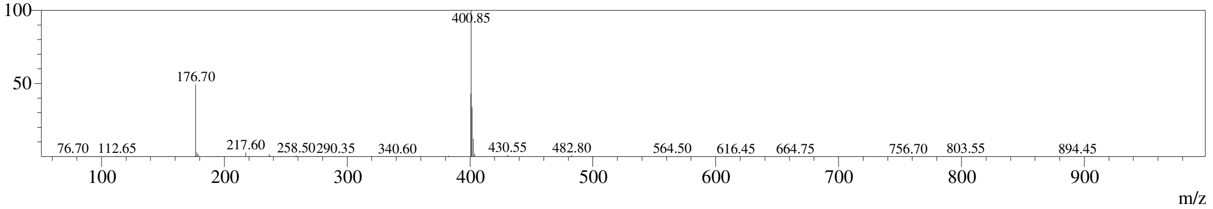

$^{1}$H NMR
500MHz ($CDCl_3$)
PRAN-1.5

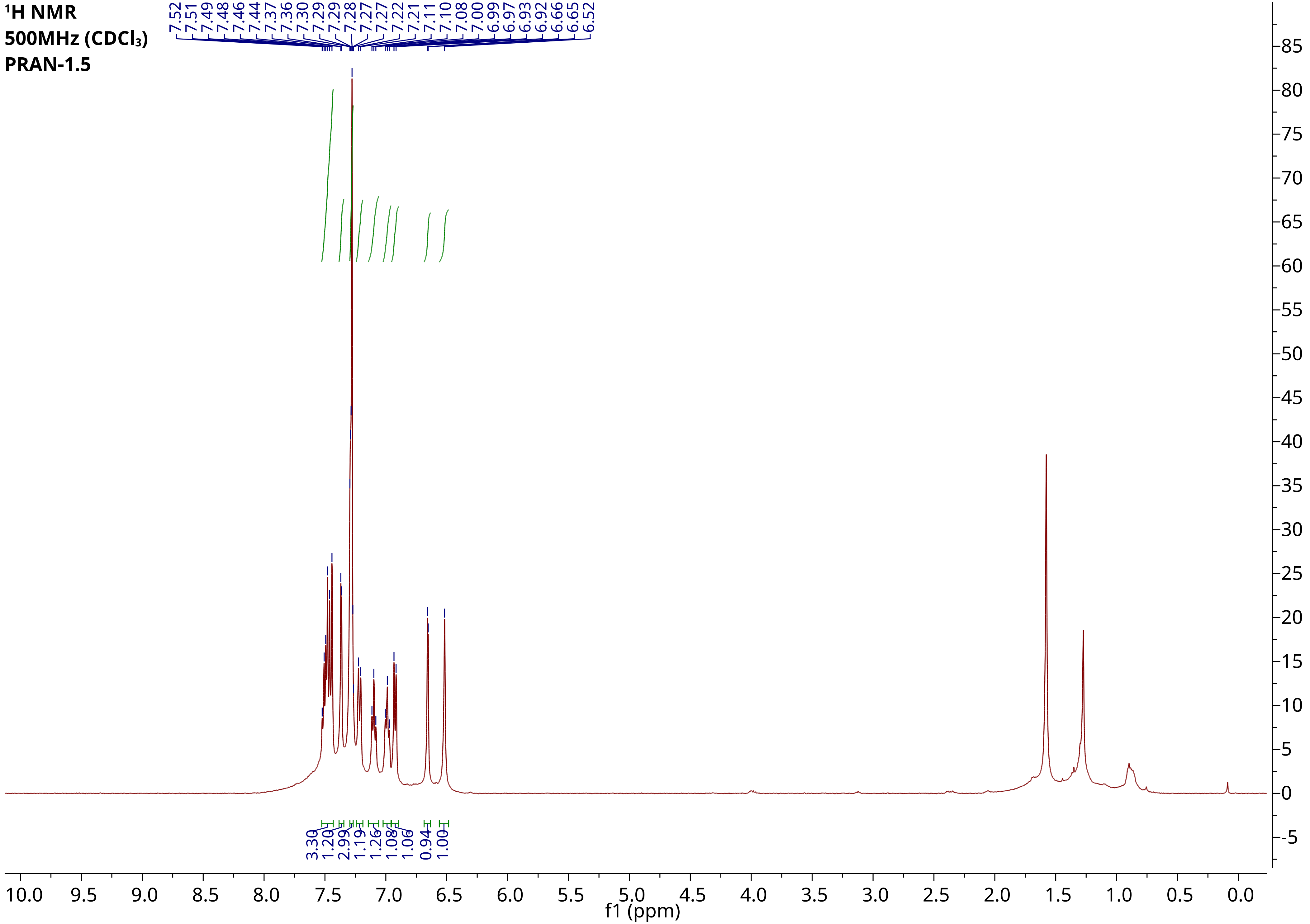

$^{13}C$ NMR
125.5MHz ($CDCl_3$)
PRAN-1.5

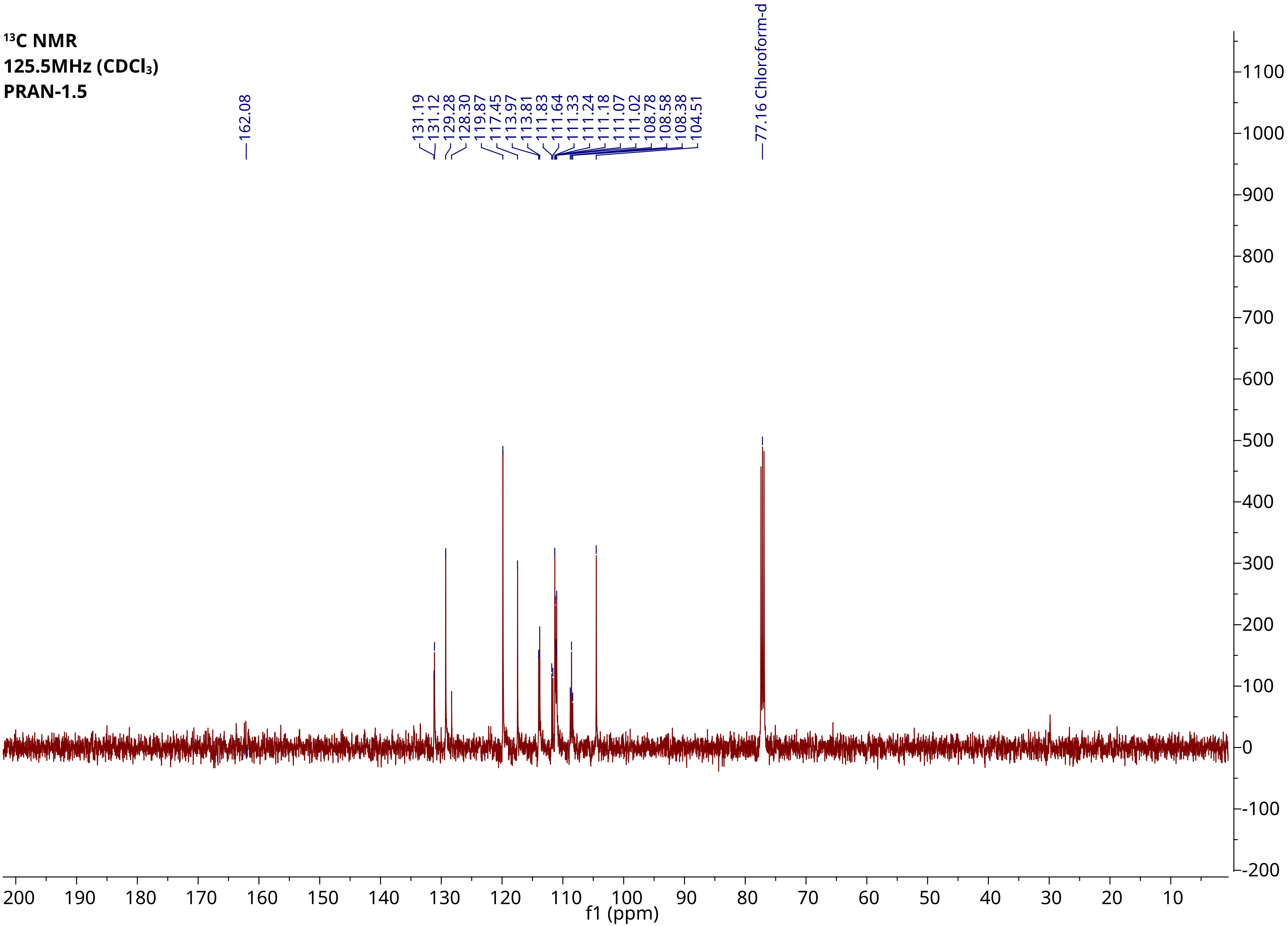

# ==== Shimadzu LCMSsolution Analysis Report ====

**Sample Name : PRAN-1.6**

Method

Column: Purospher RP-8
Mobile Phase A: H2O + 0.9% acetic acid
Mobile Phase B: ACN
% Pump B Concetrate: 50.0
Flow (ml/min): 0.6000

Detector A:SPD-20A
UV_1.Wavelength: 220
UV_2.Wavelength: 260
LC Program

| Time | Unit | Command | Value |
|---|---|---|---|
| 0.01 | Pumps | B.Conc | 50 |
| 15.00 | Pumps | B.Conc | 90 |
| 30.00 | Pumps | B.Conc | 90 |
| 30.01 | Pumps | B.Conc | 50 |
| 40.00 | Controller | Stop | |

MS Chromatogram

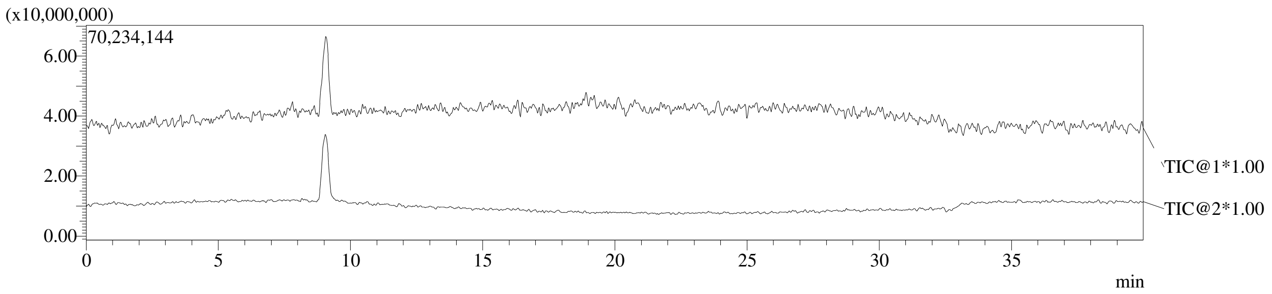


## <LC-UV Chromatogram>

Chromatogram

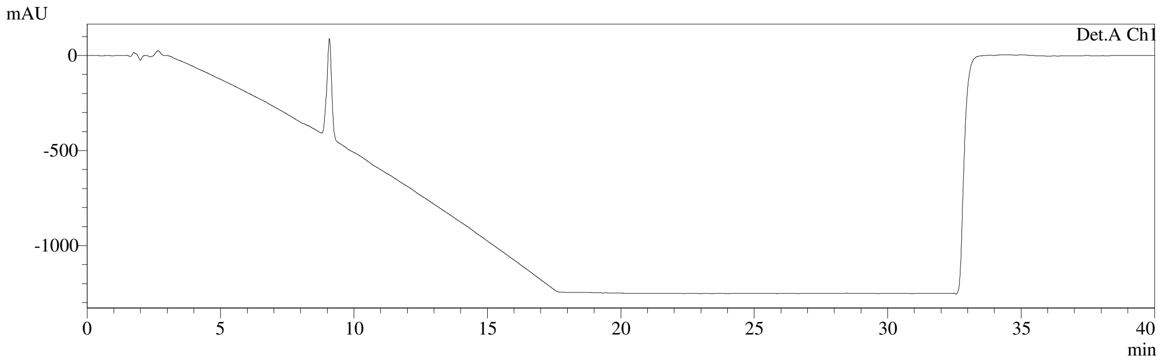

## Sample Name : PRAN-1.6

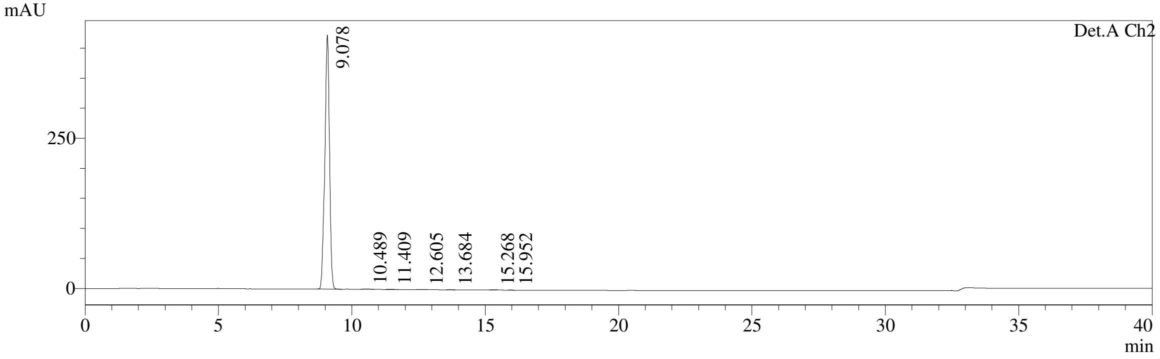


1 Det.A Ch1 / 220nm
2 Det.A Ch2 / 260nm

PeakTable

Detector A Ch2 260nm

| Peak# | Ret. Time | Area | Height | Area % | Height % |
|---|---|---|---|---|---|
| 1 | 9.078 | 5146512 | 422957 | 99.485 | 99.478 |
| 2 | 10.489 | 9167 | 506 | 0.177 | 0.119 |
| 3 | 11.409 | 5376 | 390 | 0.104 | 0.092 |
| 4 | 12.605 | 4938 | 388 | 0.095 | 0.091 |
| 5 | 13.684 | 3957 | 498 | 0.076 | 0.117 |
| 6 | 15.268 | 1642 | 214 | 0.032 | 0.050 |
| 7 | 15.952 | 1572 | 226 | 0.030 | 0.053 |
| Total | | 5173164 | 425178 | 100.000 | 100.000 |

MS Spectrum Graph

#:1 Ret.Time:Averaged 8.818-9.425(Scan#:815-871)
BG Mode:Averaged 16.965-27.923(1567-2579)
Mass Peaks:341 Base Peak:82.85(6144864) Polarity:Pos Segment1 - Event1

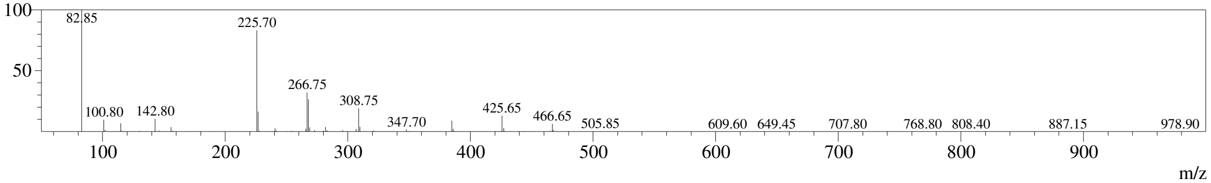


#:2 Ret.Time:Averaged 8.829-9.436(Scan#:816-872)
BG Mode:Averaged 16.976-27.923(1568-2580)
Mass Peaks:650 Base Peak:382.60(4906984) Polarity:Neg Segment1 - Event2

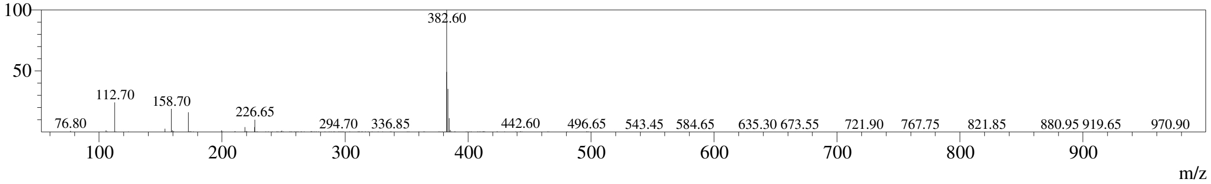

**¹H NMR 500MHz (CDCl$_3$)**
**PRAN-1.6**

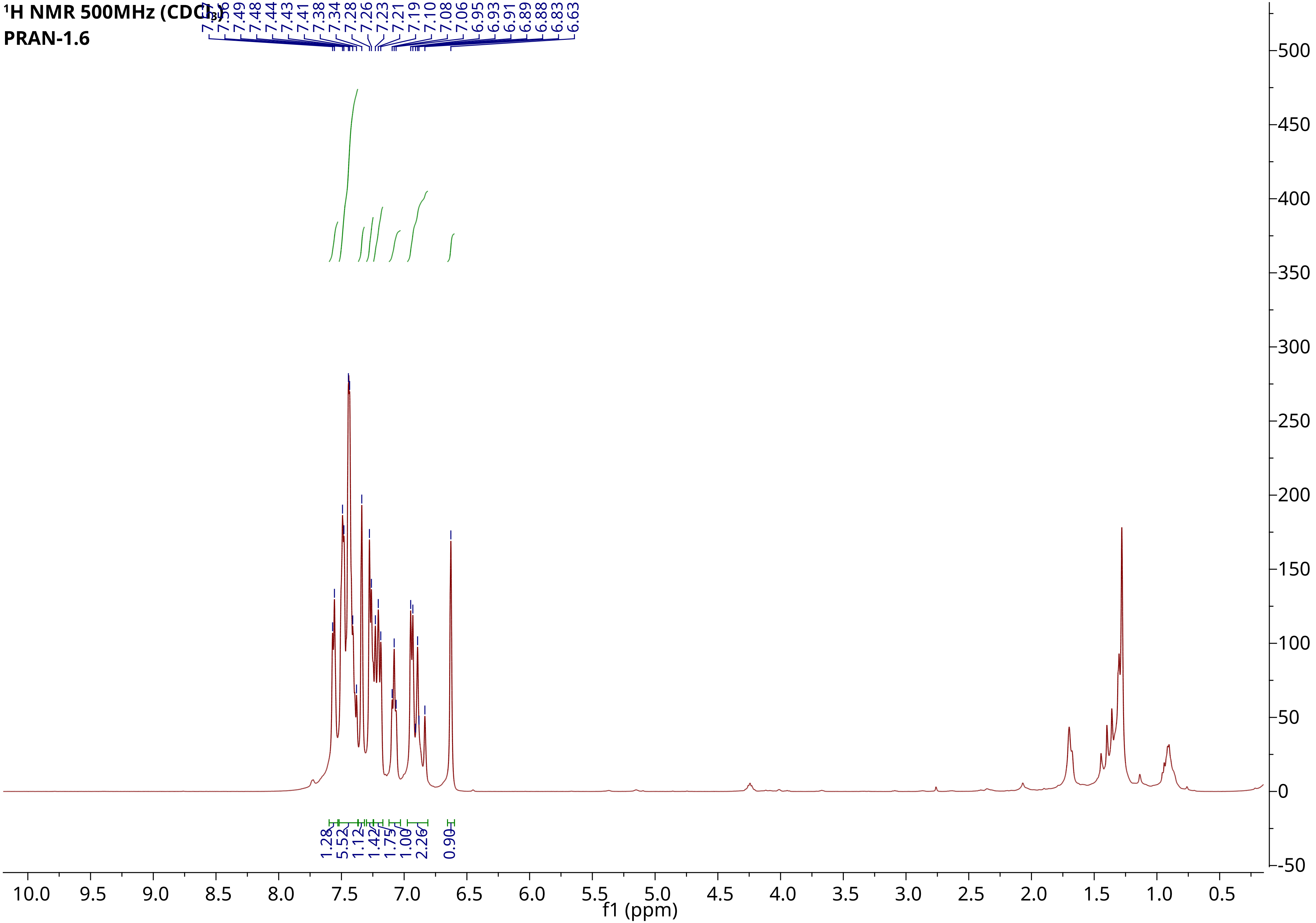

**$^{13}$C NMR**
**125.5MHz ($CDCl_3$)**
**PRAN-1.6**

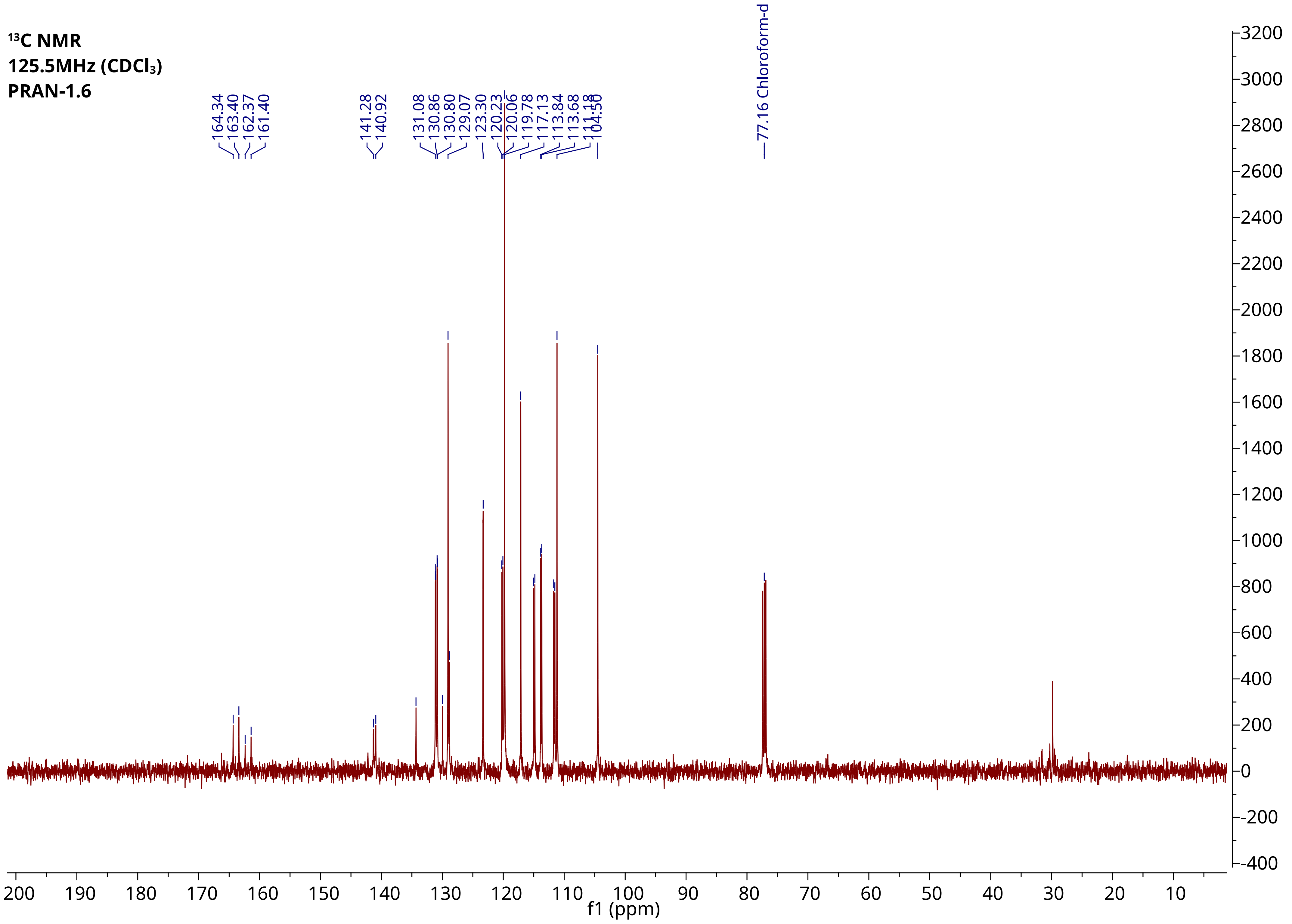

# ==== Shimadzu LCMSsolution Analysis Report ====

**Sample Name : PRAN-1.7**

Method

Column: Purospher RP-8
Mobile Phase A: $H_2O$ + 0.9% acetic acid
Mobile Phase B: ACN
% Pump B Concetrate: 50.0
Flow (ml/min): 0.6000

Detector A:SPD-20A
UV_1.Wavelength: 220
UV_2.Wavelength: 260
LC Program

| Time | Unit | Command | Value |
|---|---|---|---|
| 0.01 | Pumps | B.Conc | 50 |
| 15.00 | Pumps | B.Conc | 90 |
| 30.00 | Pumps | B.Conc | 90 |
| 30.01 | Pumps | B.Conc | 50 |
| 40.00 | Controller | Stop | |

MS Chromatogram

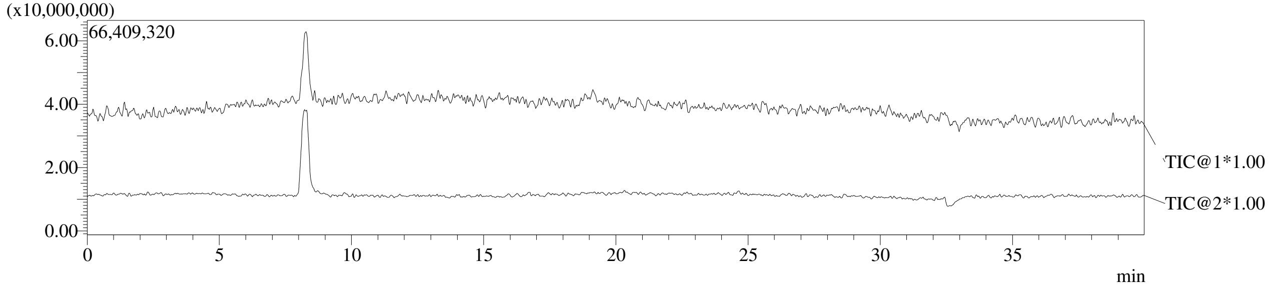


## <LC-UV Chromatogram>

Chromatogram

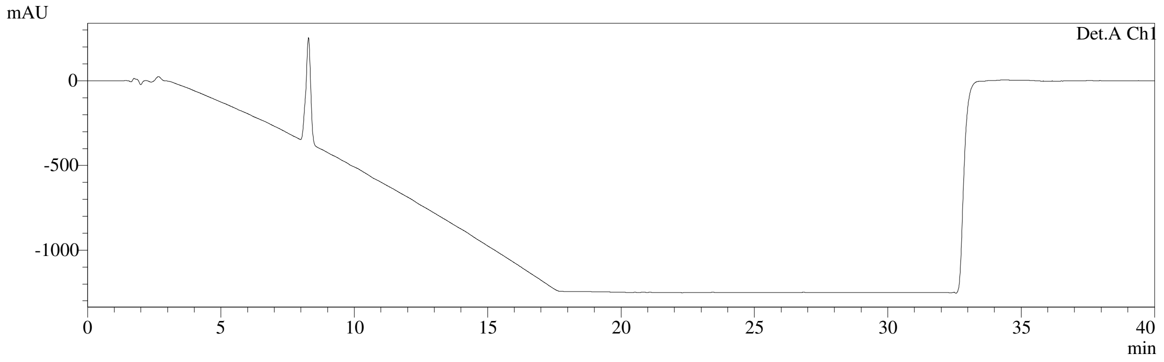

## Sample Name : PRAN-1.7

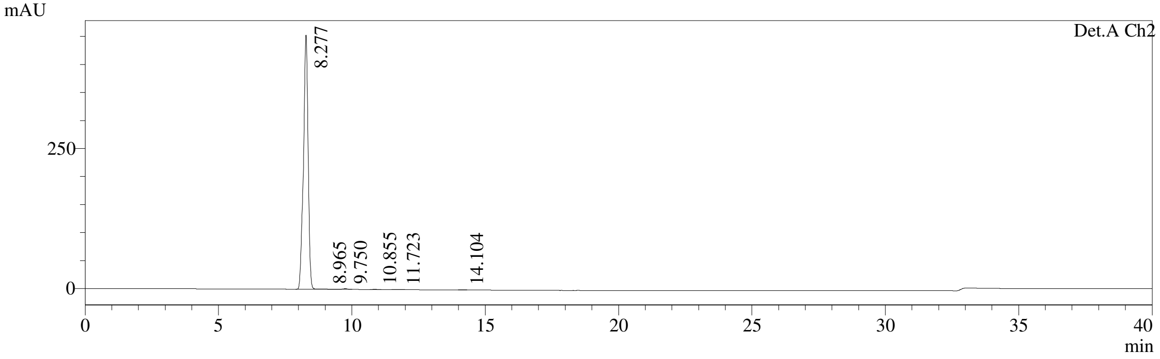


1 Det.A Ch1 / 220nm
2 Det.A Ch2 / 260nm

PeakTable

Detector A Ch2 260nm

| Peak# | Ret. Time | Area | Height | Area % | Height % |
|---|---|---|---|---|---|
| 1 | 8.277 | 5521147 | 453435 | 99.387 | 99.395 |
| 2 | 8.965 | 10057 | 575 | 0.181 | 0.126 |
| 3 | 9.750 | 14237 | 1252 | 0.256 | 0.274 |
| 4 | 10.855 | 3615 | 332 | 0.065 | 0.073 |
| 5 | 11.723 | 3856 | 303 | 0.069 | 0.066 |
| 6 | 14.104 | 2269 | 299 | 0.041 | 0.065 |
| Total | | 5555181 | 456195 | 100.000 | 100.000 |

MS Spectrum Graph

#:1 Ret.Time:Averaged 8.082-9.338(Scan#:747-863)
BG Mode:Averaged 14.733-17.060(1361-1575)
Mass Peaks:500 Base Peak:225.75(1543155) Polarity:Pos Segment1 - Event1

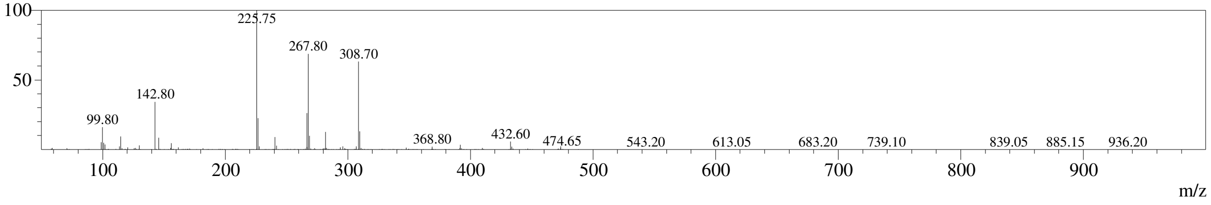


#:2 Ret.Time:Averaged 8.093-9.349(Scan#:748-864)
BG Mode:Averaged 14.744-17.060(1362-1576)
Mass Peaks:536 Base Peak:389.60(2794140) Polarity:Neg Segment1 - Event2

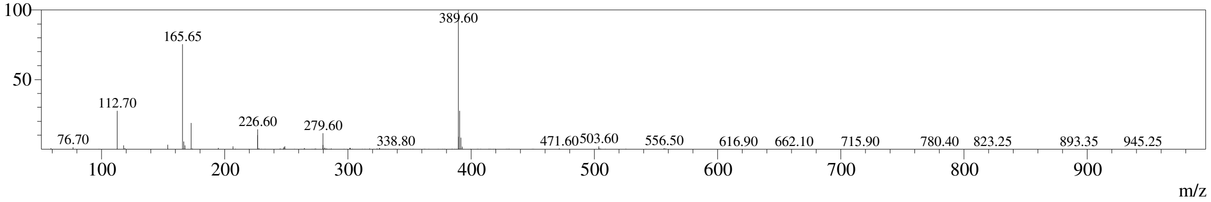

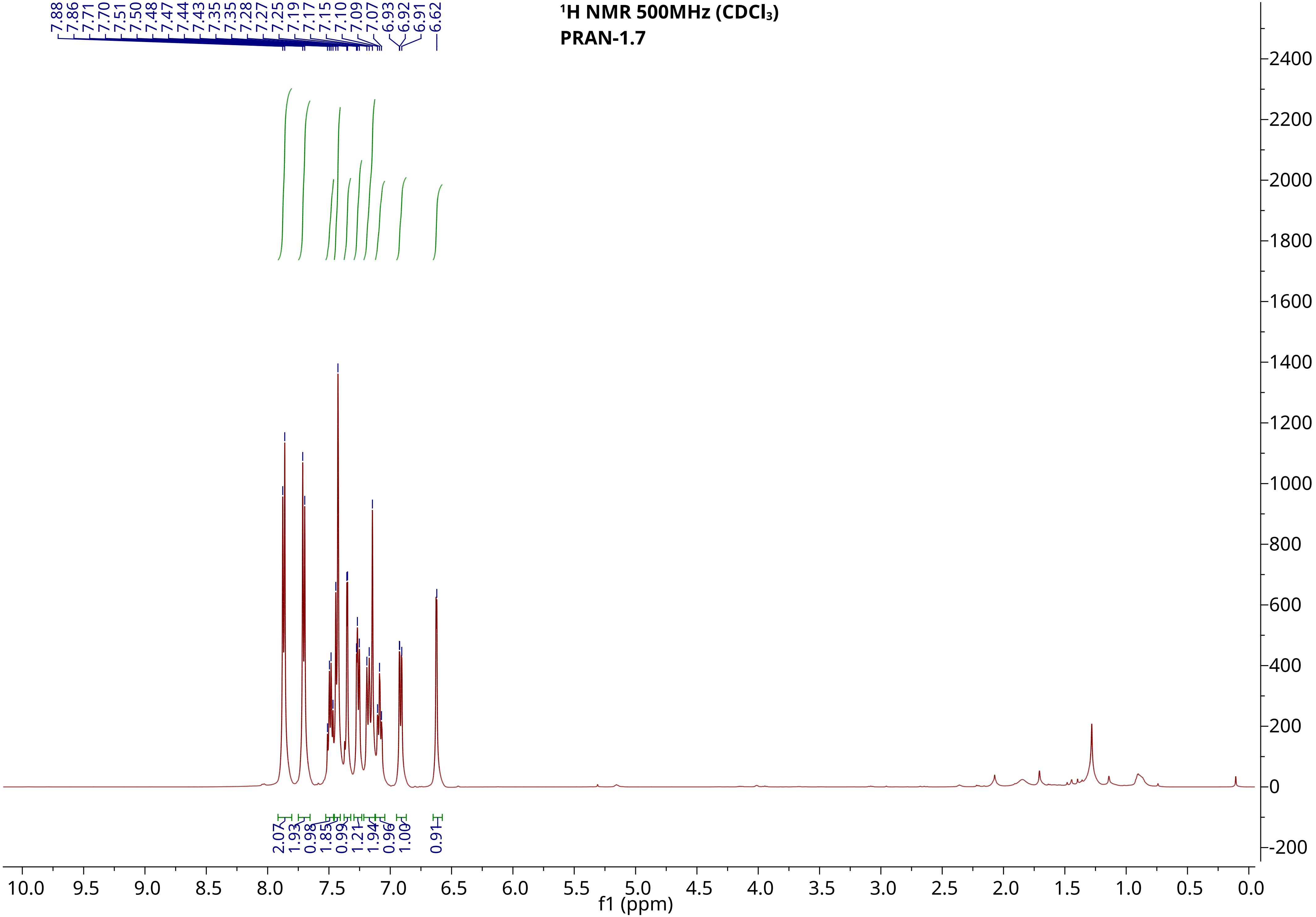
¹H NMR 500MHz (CDCl₃)
PRAN-1.7
7.88
7.86
7.71
7.70
7.51
7.50
7.48
7.47
7.44
7.43
7.35
7.35
7.28
7.27
7.25
7.19
7.17
7.15
7.10
7.09
7.07
6.93
6.92
6.91
6.62
2.07
1.93
0.98
1.85
0.99
1.21
1.94
0.96
1.00
0.91
f1 (ppm)

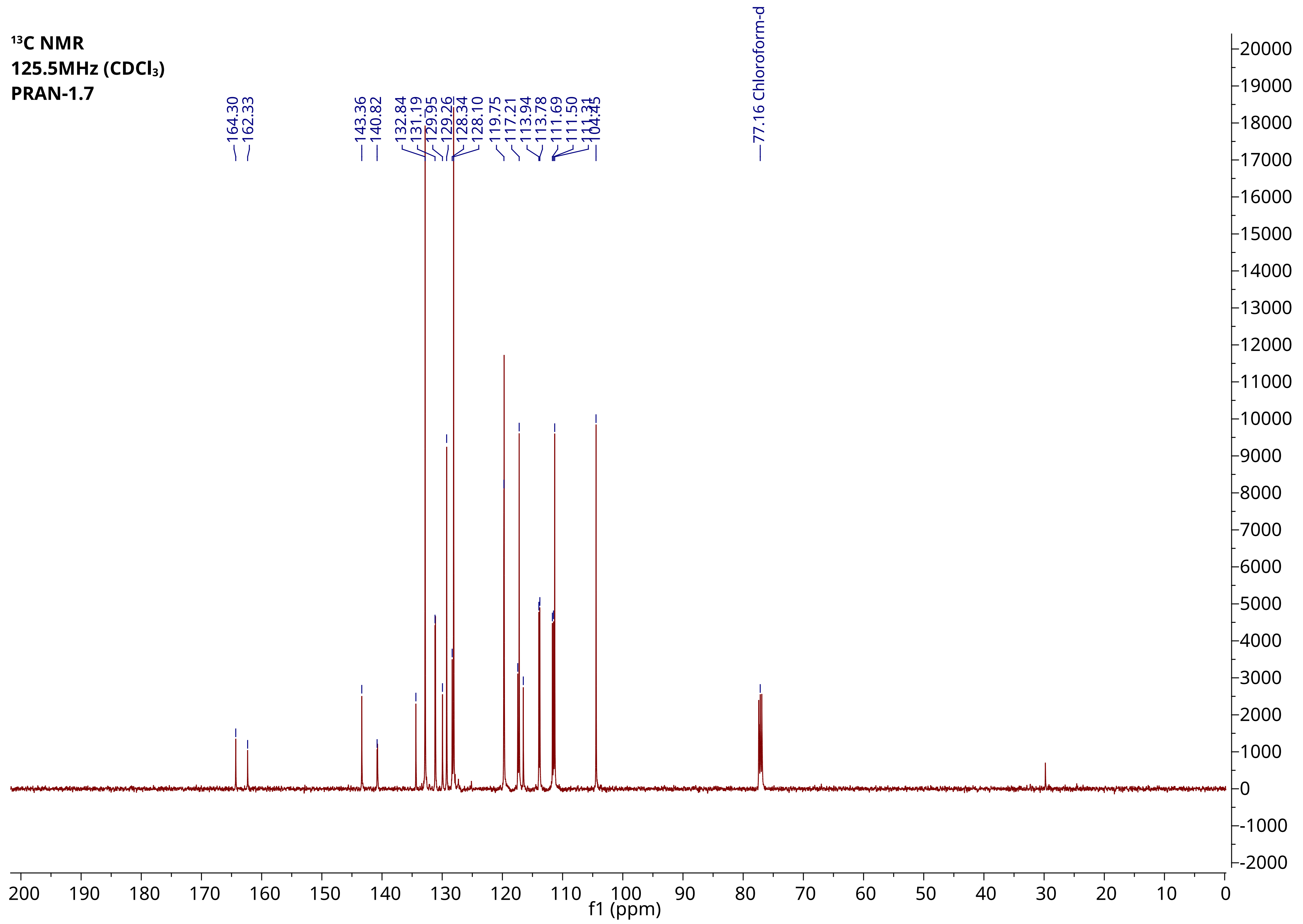

13C NMR
125.5MHz (CDCl3)
PRAN-1.7
164.30
162.33
143.36
140.82
132.84
131.19
129.95
129.26
128.34
128.10
119.75
117.21
113.94
113.78
111.69
111.50
104.45
77.16 Chloroform-d
f1 (ppm)

# ==== Shimadzu LCMSsolution Analysis Report ====

**Sample Name : PRAN-1.8**

Method

Column: Purospher RP-8
Mobile Phase A: H2O + 0.9% acetic acid
Mobile Phase B: ACN
% Pump B Concetrate: 50.0
Flow (ml/min): 0.6000

Detector A:SPD-20A
UV_1.Wavelength: 220
UV_2.Wavelength: 260
LC Program

| Time | Unit | Command | Value |
|---|---|---|---|
| 0.01 | Pumps | B.Conc | 50 |
| 15.00 | Pumps | B.Conc | 90 |
| 30.00 | Pumps | B.Conc | 90 |
| 30.01 | Pumps | B.Conc | 50 |
| 40.00 | Controller | Stop | |

MS Chromatogram

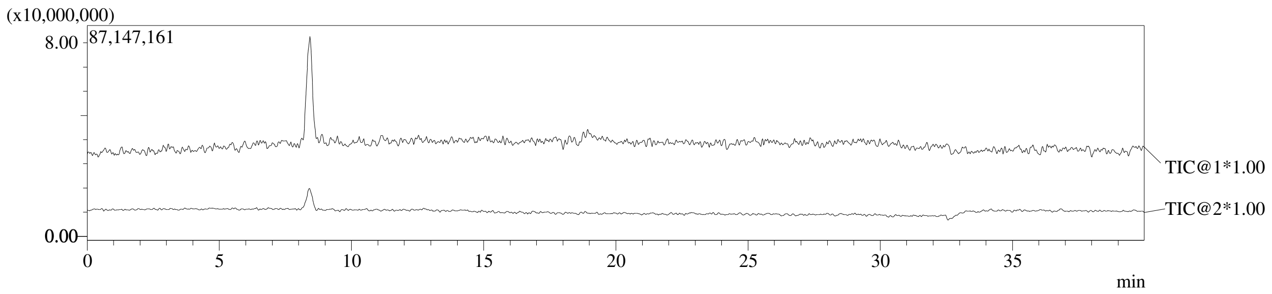


## <LC-UV Chromatogram>

Chromatogram

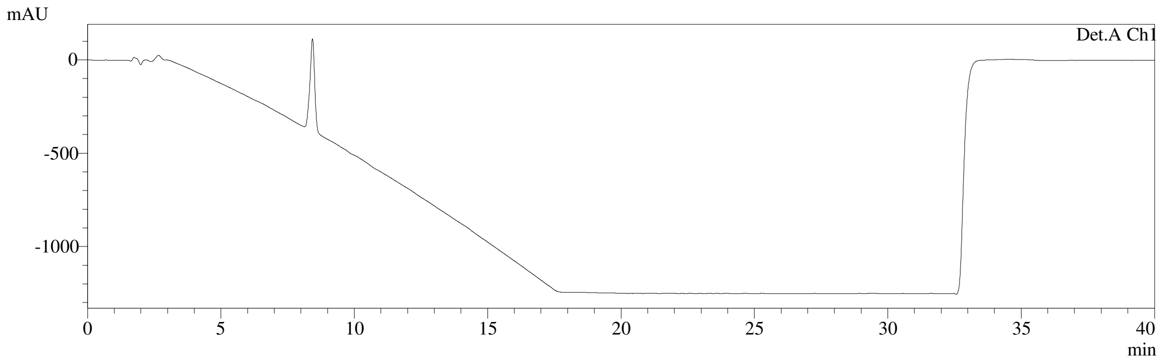

## Sample Name : PRAN-1.8

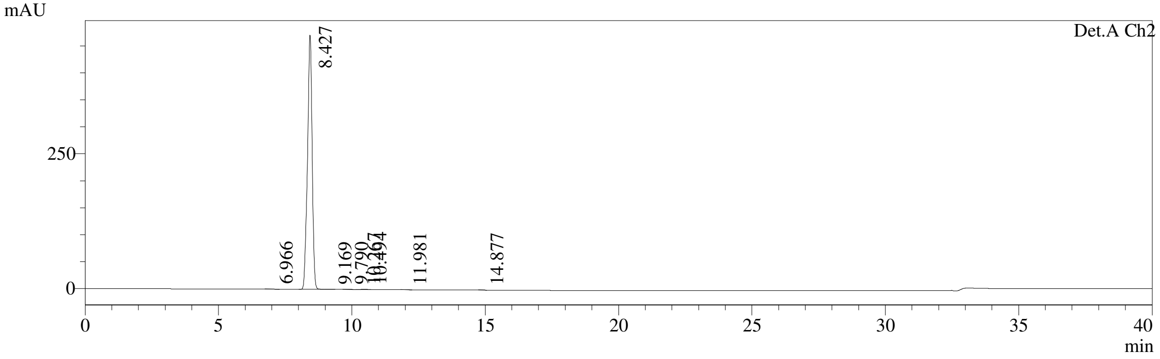


1 Det.A Ch1 / 220nm
2 Det.A Ch2 / 260nm

PeakTable

Detector A Ch2 260nm

| Peak# | Ret. Time | Area | Height | Area % | Height % |
|---|---|---|---|---|---|
| 1 | 6.966 | 3432 | 303 | 0.060 | 0.064 |
| 2 | 8.427 | 5711062 | 470894 | 99.616 | 99.558 |
| 3 | 9.169 | 3783 | 342 | 0.066 | 0.072 |
| 4 | 9.790 | 1658 | 107 | 0.029 | 0.023 |
| 5 | 10.267 | 1101 | 145 | 0.019 | 0.031 |
| 6 | 10.494 | 6106 | 517 | 0.107 | 0.109 |
| 7 | 11.981 | 2688 | 263 | 0.047 | 0.056 |
| 8 | 14.877 | 3266 | 415 | 0.057 | 0.088 |
| Total | | 5733098 | 472986 | 100.000 | 100.000 |

MS Spectrum Graph

#:1 Ret.Time:Averaged 8.407-8.450(Scan#:777-781)
BG Mode:Calc 8.168<->8.775(755<->811)
Mass Peaks:585 Base Peak:225.70(13610574) Polarity:Pos Segment1 - Event1

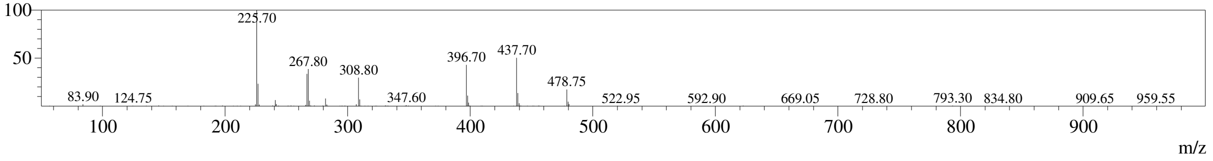


#:2 Ret.Time:Averaged 11.137-11.180(Scan#:1029-1033)
BG Mode:Calc 10.963<->11.418(1013<->1055)
Mass Peaks:504 Base Peak:141.75(2516136) Polarity:Pos Segment1 - Event1

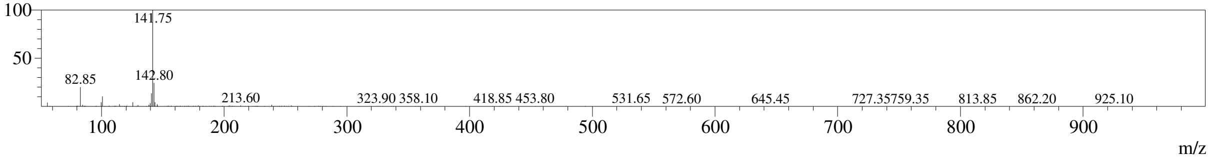

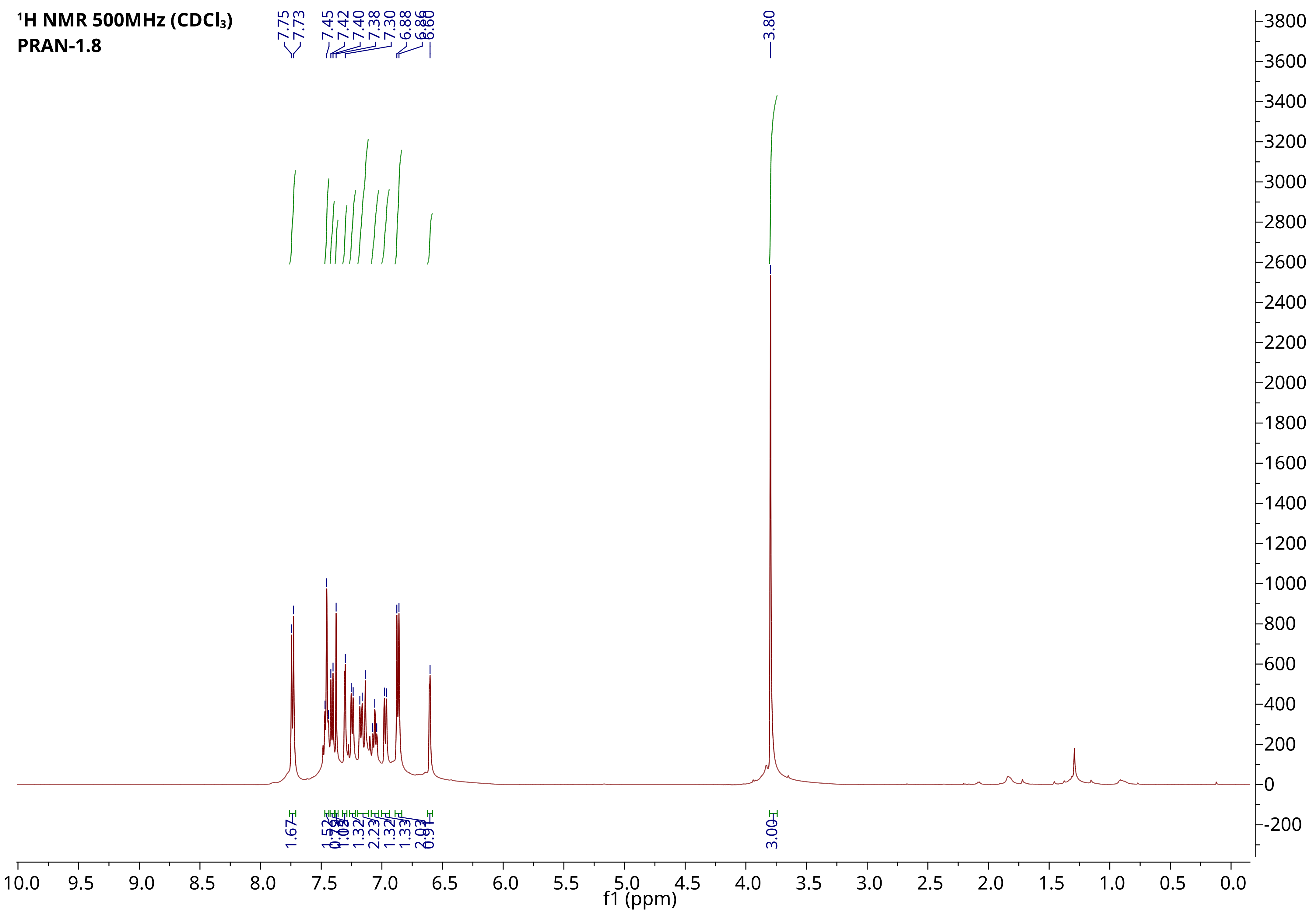

¹H NMR 500MHz (CDCl₃)
PRAN-1.8
7.75
7.73
7.45
7.42
7.40
7.38
7.30
6.88
6.86
6.60
3.80
1.67
1.52
1.32
2.23
1.32
1.33
2.03
0.91
3.00
f1 (ppm)

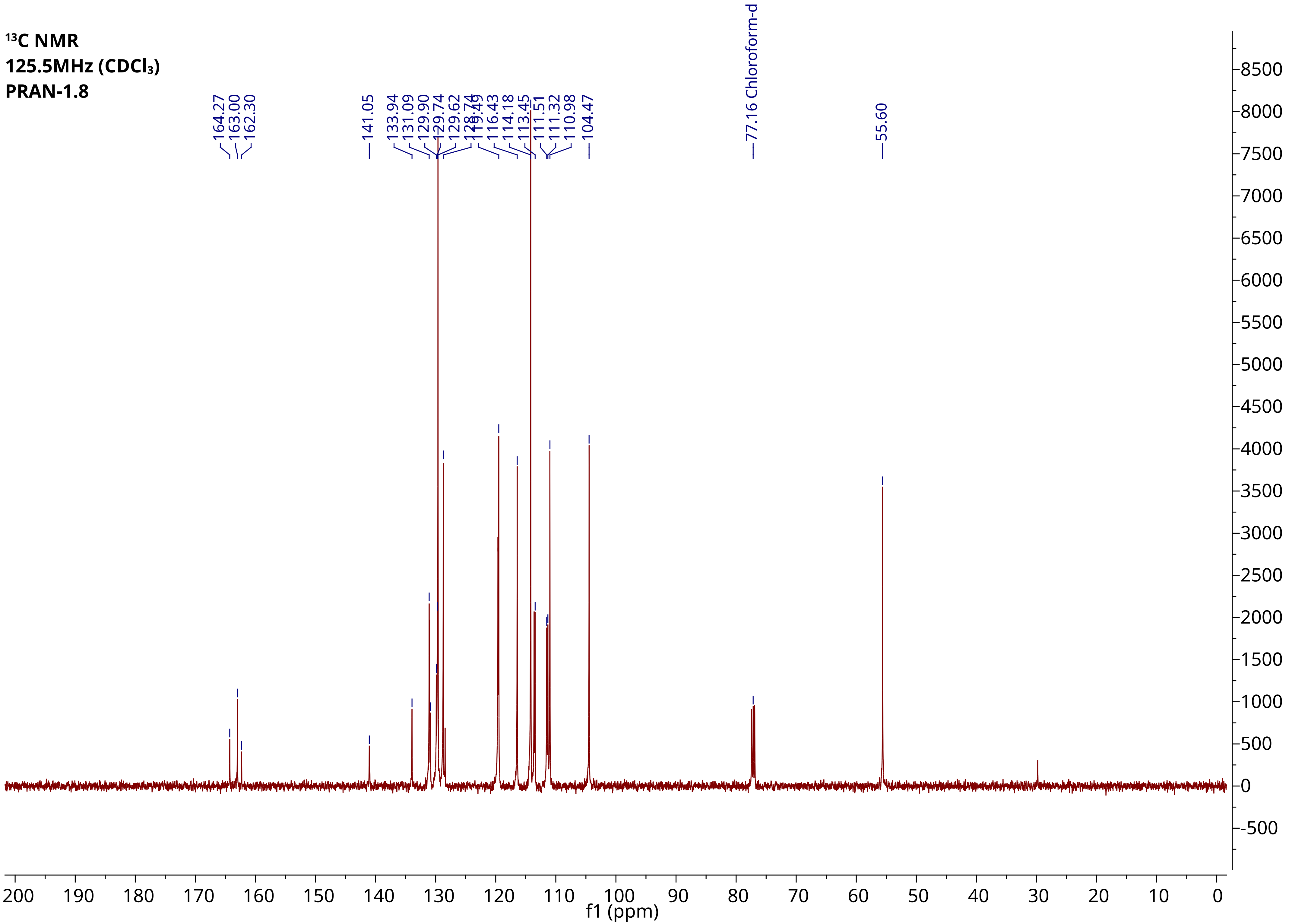

13C NMR
125.5MHz (CDCl3)
PRAN-1.8
164.27
163.00
162.30
141.05
133.94
131.09
129.90
129.74
129.62
128.74
119.49
116.43
114.18
113.45
111.51
111.32
110.98
104.47
77.16 Chloroform-d
55.60
f1 (ppm)

# ==== Shimadzu LCMSsolution Analysis Report ====

**Sample Name : PRAN-1.9**

Method

Column: Purospher RP-8
Mobile Phase A: H2O + 0.9% acetic acid
Mobile Phase B: ACN
% Pump B Concetrate: 70.0
Flow (ml/min): 0.6000

Detector A:SPD-20A
UV_1.Wavelength: 218
UV_2.Wavelength: 260
LC Program

| Time | Unit | Command | Value |
|---|---|---|---|
| 30.00 | Controller | Stop | |

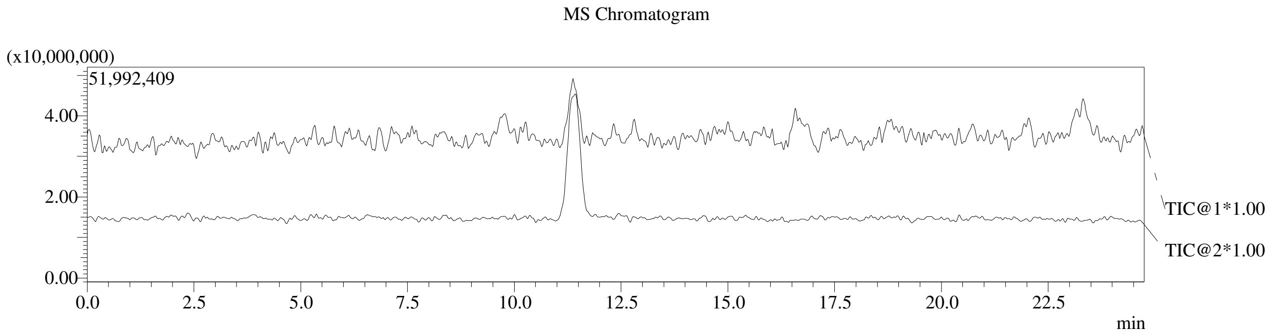


## <LC-UV Chromatogram>

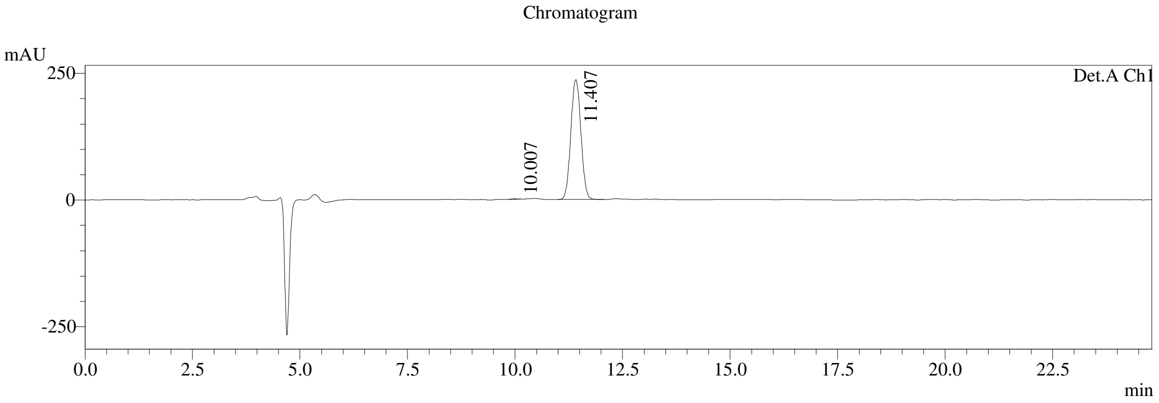

## Sample Name : PRAN-1.9

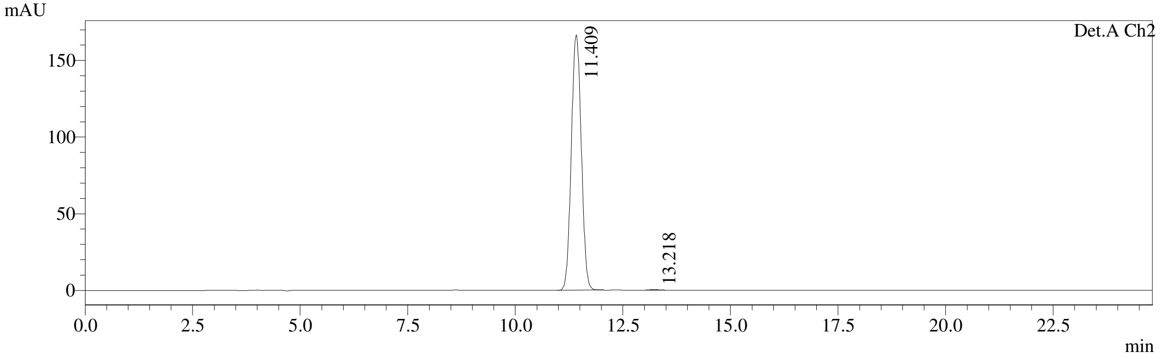


1 Det.A Ch1 / 218nm
2 Det.A Ch2 / 260nm

PeakTable

Detector A Ch1 218nm

| Peak# | Ret. Time | Area | Height | Area % | Height % |
|---|---|---|---|---|---|
| 1 | 10.007 | 7679 | 795 | 0.199 | 0.335 |
| 2 | 11.407 | 3857464 | 236403 | 99.801 | 99.665 |
| Total | | 3865143 | 237198 | 100.000 | 100.000 |

PeakTable

Detector A Ch2 260nm

| Peak# | Ret. Time | Area | Height | Area % | Height % |
|---|---|---|---|---|---|
| 1 | 11.409 | 2692456 | 166435 | 99.818 | 99.796 |
| 2 | 13.218 | 4913 | 340 | 0.182 | 0.204 |
| Total | | 2697369 | 166775 | 100.000 | 100.000 |

MS Spectrum Graph

#:1 Ret.Time:Averaged 11.223-11.657(Scan#:1037-1077)
BG Mode:Averaged 14.083-16.083(1301-1485)
Mass Peaks:474 Base Peak:225.70(4357740) Polarity:Pos Segment1 - Event1

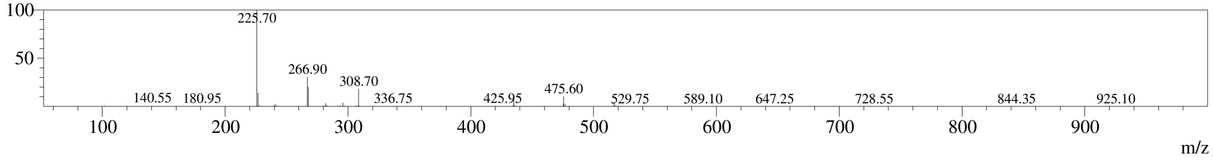


#:2 Ret.Time:Averaged 11.234-11.668(Scan#:1038-1078)
BG Mode:Averaged 14.094-16.083(1302-1486)
Mass Peaks:485 Base Peak:432.60(12391482) Polarity:Neg Segment1 - Event2

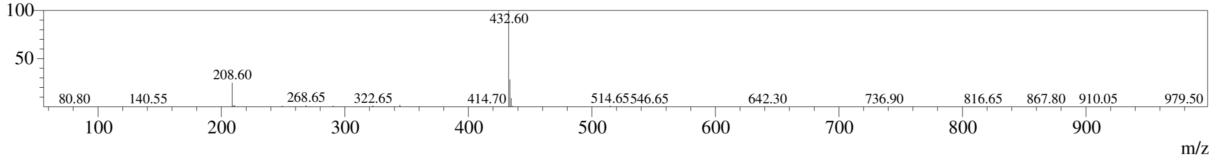

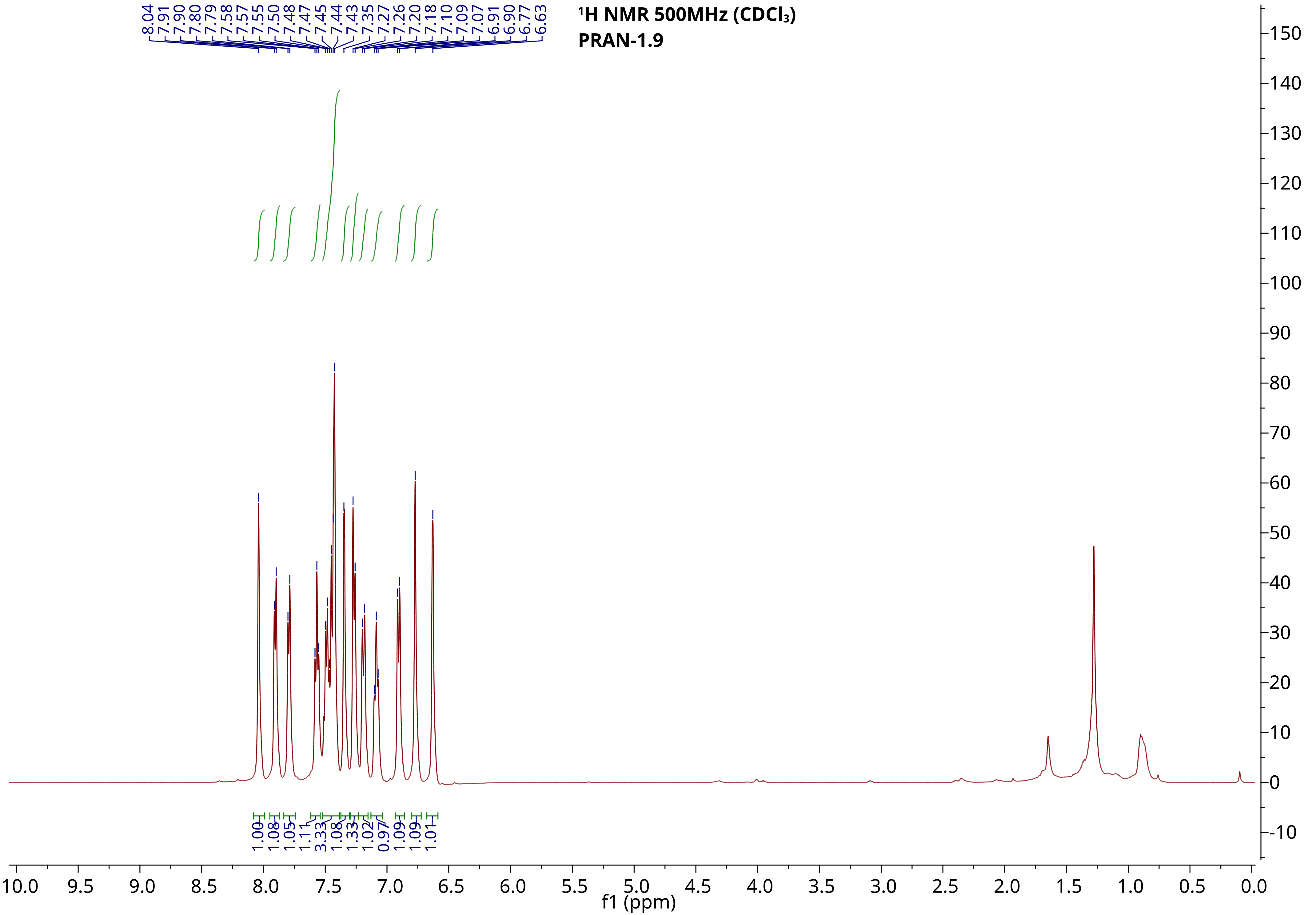

¹H NMR 500MHz (CDCl₃)
PRAN-1.9
8.04 7.91 7.90 7.80 7.79 7.58 7.57 7.55 7.50 7.48 7.47 7.45 7.44 7.43 7.35 7.27 7.26 7.20 7.18 7.10 7.09 7.07 6.91 6.90 6.77 6.63
1.00 1.08 1.05 1.11 3.33 1.08 1.33 1.02 0.97 1.09 1.09 1.01
f1 (ppm)

$^{13}C$ NMR
125.5MHz ($CDCl_3$)
PRAN-1.9

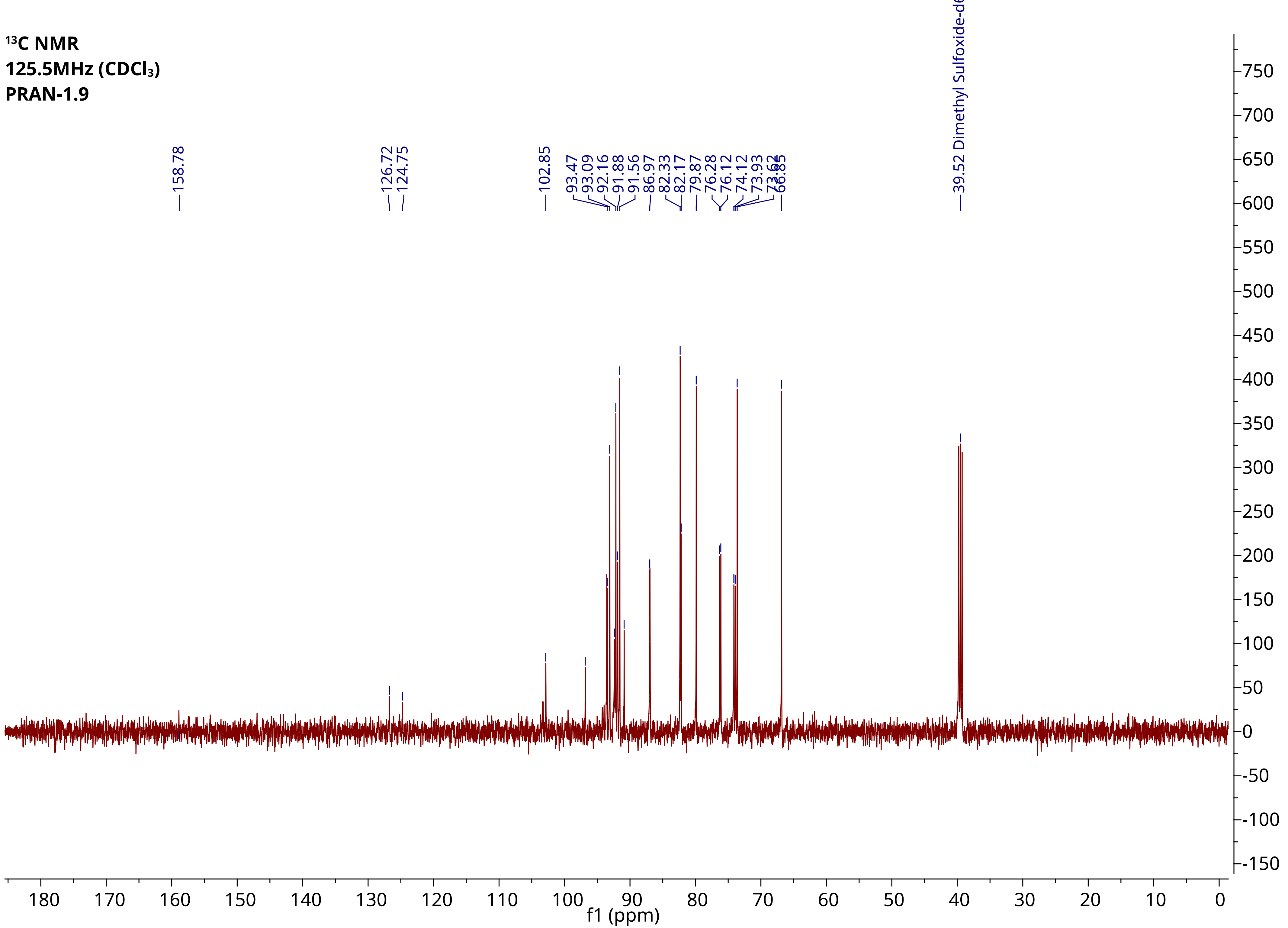

# ==== Shimadzu LCMSsolution Analysis Report ====

**Sample Name : PRAN-1.10**

Method

Column: Purospher RP-8
Mobile Phase A: $H_2O$ + 0.9% acetic acid
Mobile Phase B: ACN
% Pump B Concetrate: 75.0
Flow (ml/min): 0.6000

Detector A:SPD-20A
UV_1.Wavelength: 218
UV_2.Wavelength: 260
LC Program

| Time | Unit | Command | Value |
|---|---|---|---|
| 30.00 | Controller | Stop | |

MS Chromatogram

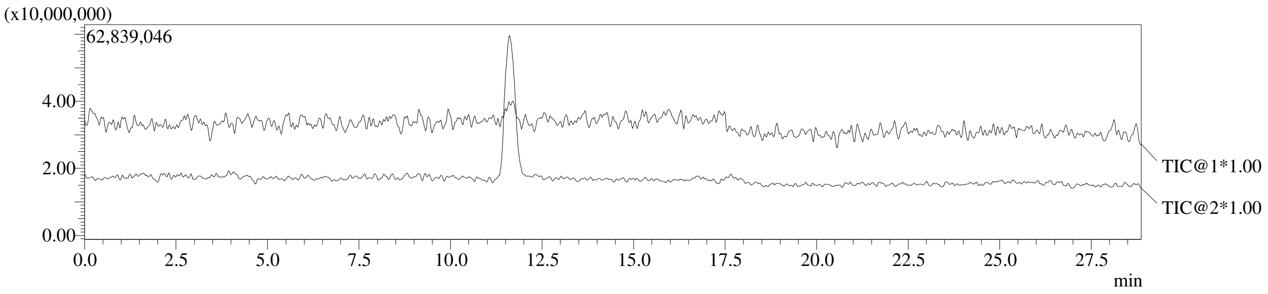


## <LC-UV Chromatogram>

Chromatogram

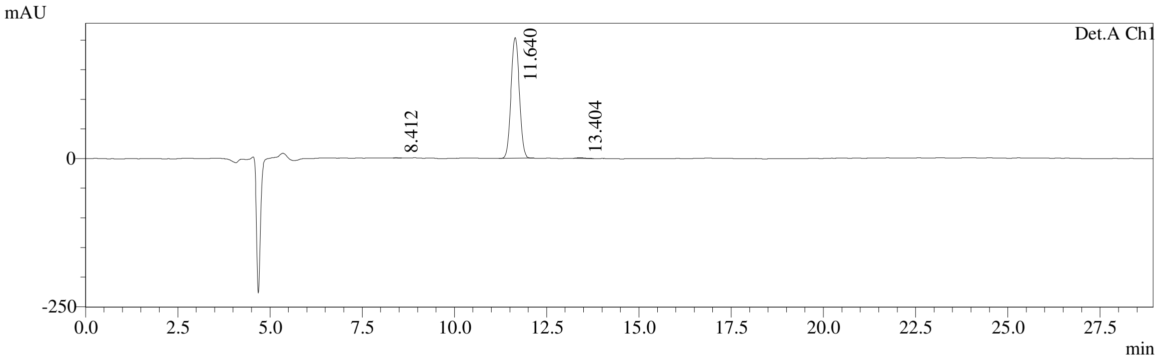

## Sample Name : PRAN-1.10

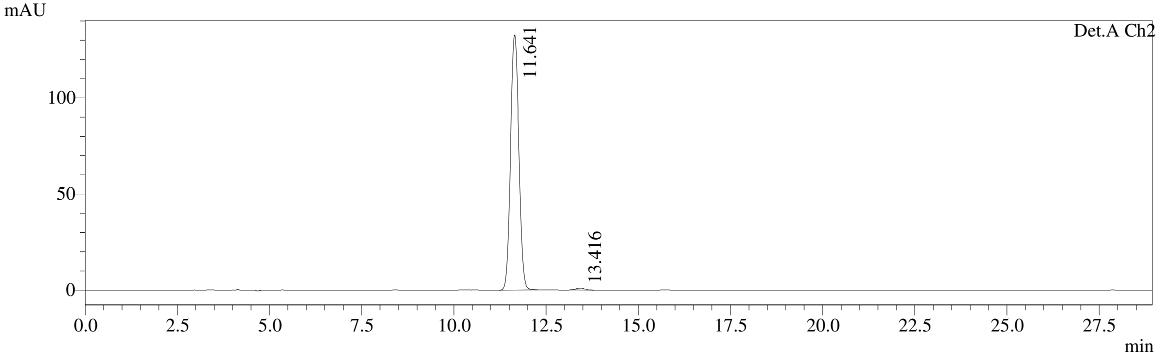


1 Det.A Ch1 / 218nm
2 Det.A Ch2 / 260nm

PeakTable

Detector A Ch1 218nm

| Peak# | Ret. Time | Area | Height | Area % | Height % |
|---|---|---|---|---|---|
| 1 | 8.412 | 3006 | 526 | 0.093 | 0.256 |
| 2 | 11.640 | 3197654 | 203675 | 99.317 | 99.124 |
| 3 | 13.404 | 18983 | 1274 | 0.590 | 0.620 |
| Total | | 3219643 | 205475 | 100.000 | 100.000 |

PeakTable

Detector A Ch2 260nm

| Peak# | Ret. Time | Area | Height | Area % | Height % |
|---|---|---|---|---|---|
| 1 | 11.641 | 2078735 | 132590 | 99.223 | 99.281 |
| 2 | 13.416 | 16283 | 960 | 0.777 | 0.719 |
| Total | | 2095018 | 133550 | 100.000 | 100.000 |

MS Spectrum Graph

#:1 Ret.Time:Averaged 11.245-11.938(Scan#:1039-1103)
BG Mode:Averaged 5.222-6.767(483-625)
Mass Peaks:505 Base Peak:225.65(999205) Polarity:Pos Segment1 - Event1

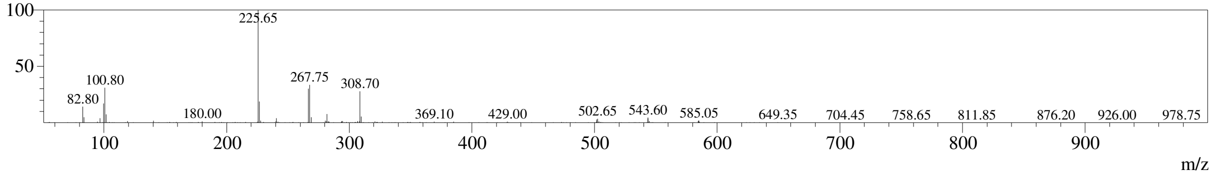


#:2 Ret.Time:Averaged 11.256-11.949(Scan#:1040-1104)
BG Mode:Averaged 5.232-6.767(484-626)
Mass Peaks:541 Base Peak:500.65(9510272) Polarity:Neg Segment1 - Event2

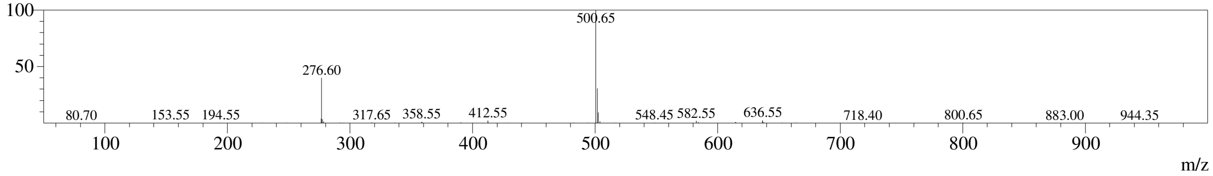

**¹H NMR 500MHz (CDCl₃)**
**PRAN-1.10**

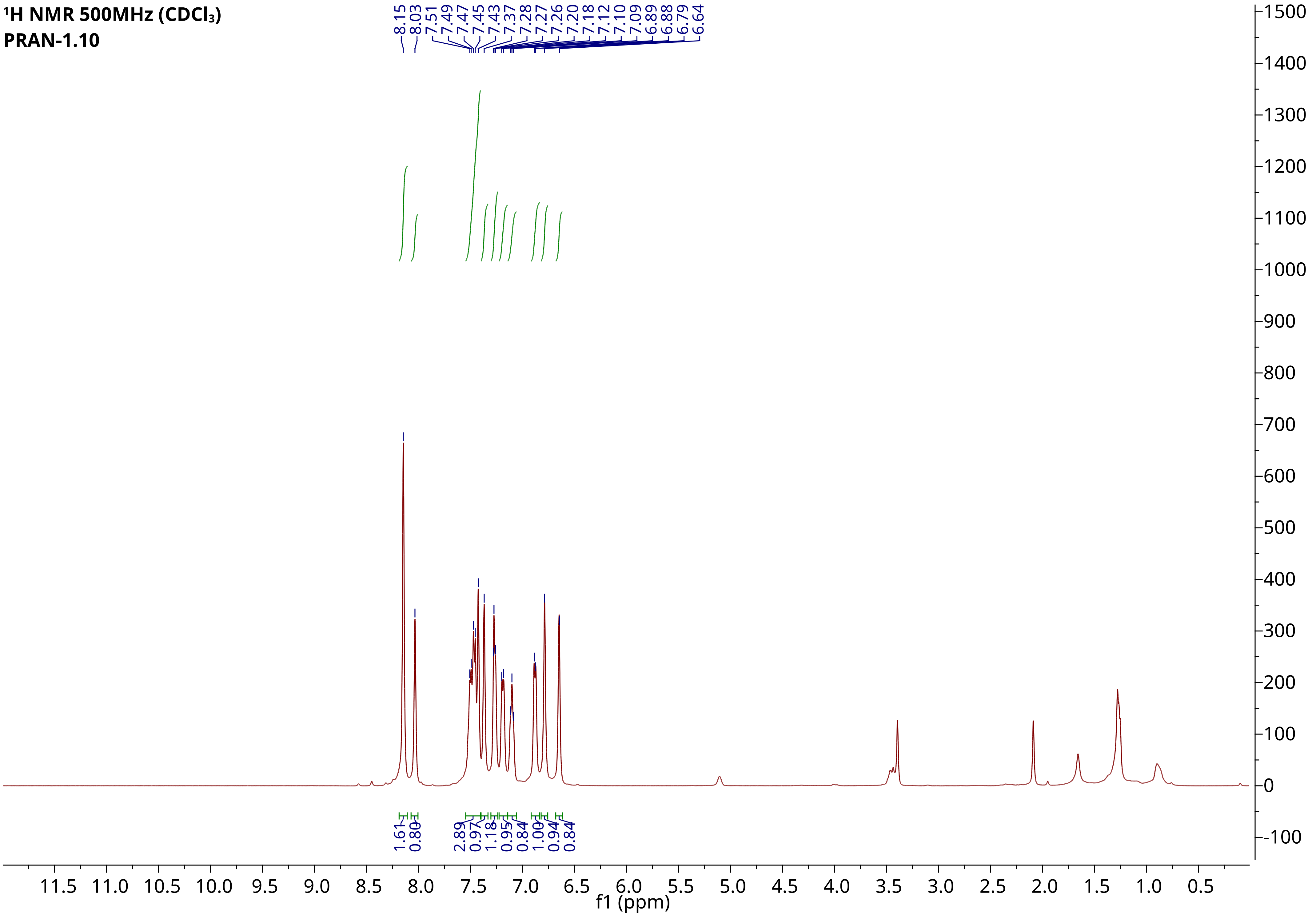

$^{13}C$ NMR
125.5MHz ($CDCl_3$)
PRAN-1.10

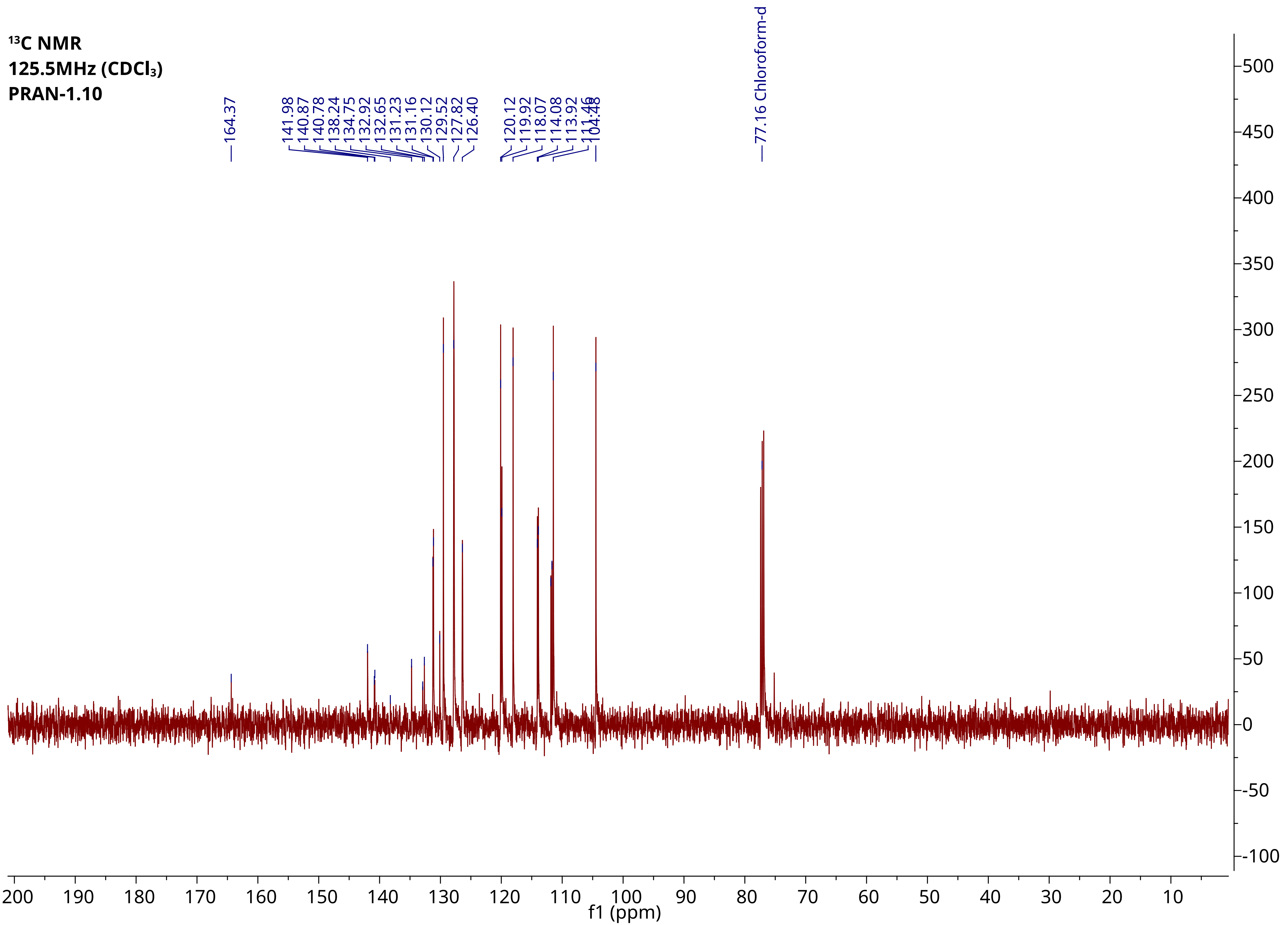

# ==== Shimadzu LCMSsolution Analysis Report ====

**Sample Name : PRAN-2.1**

Method

Column: Shim Pack - XR-ODS
Mobile Phase A: H2O + 0.9% acetic acid
Mobile Phase B: ACN
% Pump B Concetrate: 10.0
Flow (ml/min): 0.6000

Detector A:SPD-20A
UV_1.Wavelength: 217
UV_2.Wavelength: 254
LC Program

| Time | Unit | Command | Value |
|---|---|---|---|
| 0.01 | Pumps | B.Conc | 10 |
| 15.00 | Pumps | B.Conc | 90 |
| 30.00 | Pumps | B.Conc | 90 |
| 30.01 | Pumps | B.Conc | 10 |
| 40.00 | Controller | Stop | |

MS Chromatogram

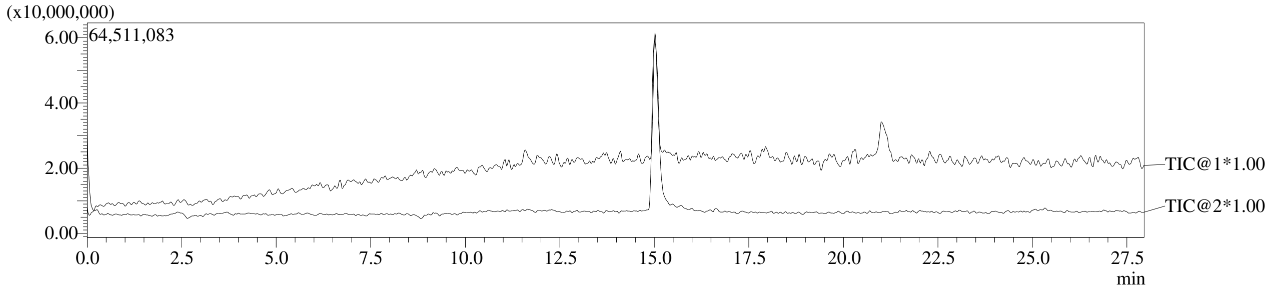


## <LC-UV Chromatogram>

Chromatogram

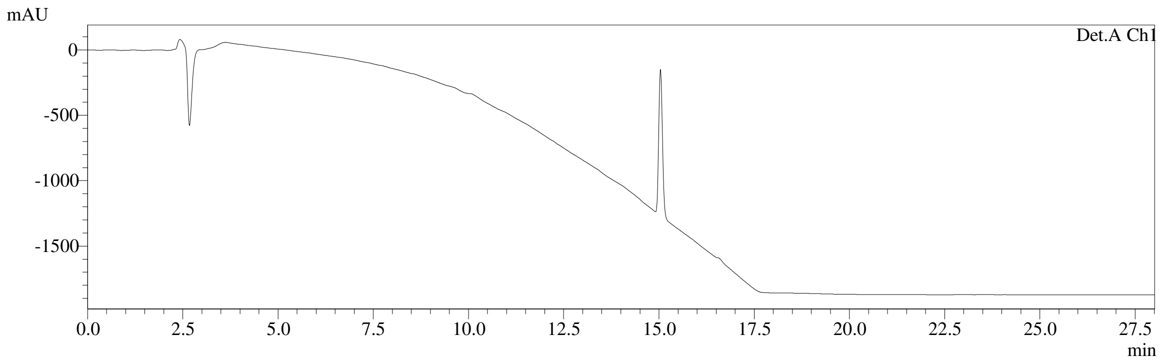

## Sample Name : PRAN-2.1

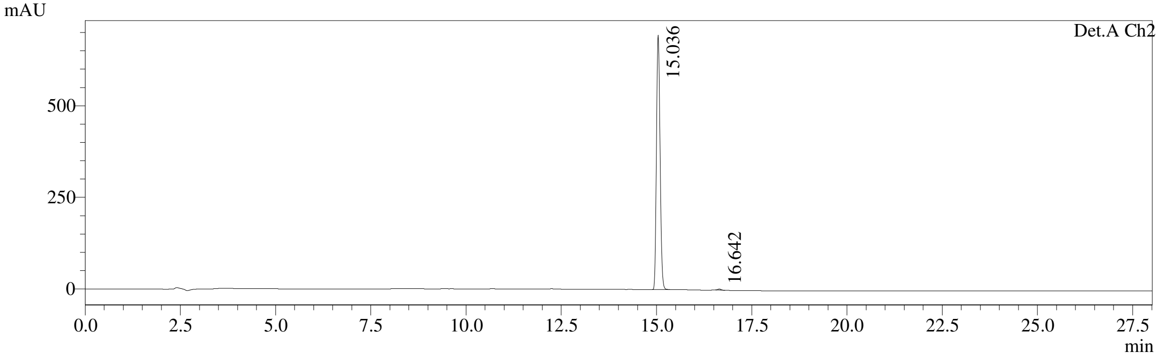


1 Det.A Ch1 / 217nm
2 Det.A Ch2 / 254nm

PeakTable

Detector A Ch2 254nm

| Peak# | Ret. Time | Area | Height | Area % | Height % |
|---|---|---|---|---|---|
| 1 | 15.036 | 4553154 | 694857 | 99.593 | 99.556 |
| 2 | 16.642 | 18590 | 3099 | 0.407 | 0.444 |
| Total | | 4571744 | 697956 | 100.000 | 100.000 |

MS Spectrum Graph

#:1 Ret.Time:Averaged 14.993-15.275(Scan#:1385-1411)
BG Mode:Averaged 19.045-19.854(1759-1833)
Mass Peaks:592 Base Peak:275.70(5562103) Polarity:Pos Segment1 - Event1

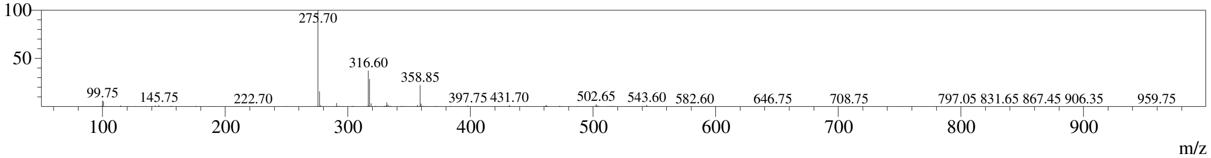


#:2 Ret.Time:Averaged 15.004-15.286(Scan#:1386-1412)
BG Mode:Averaged 19.056-19.854(1760-1834)
Mass Peaks:644 Base Peak:459.95(16456187) Polarity:Neg Segment1 - Event2

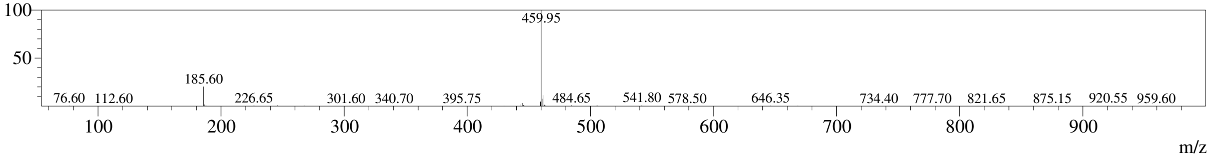

$^{1}$H NMR 500MHz ($CDCl_3$)
**PRAN-2.1**

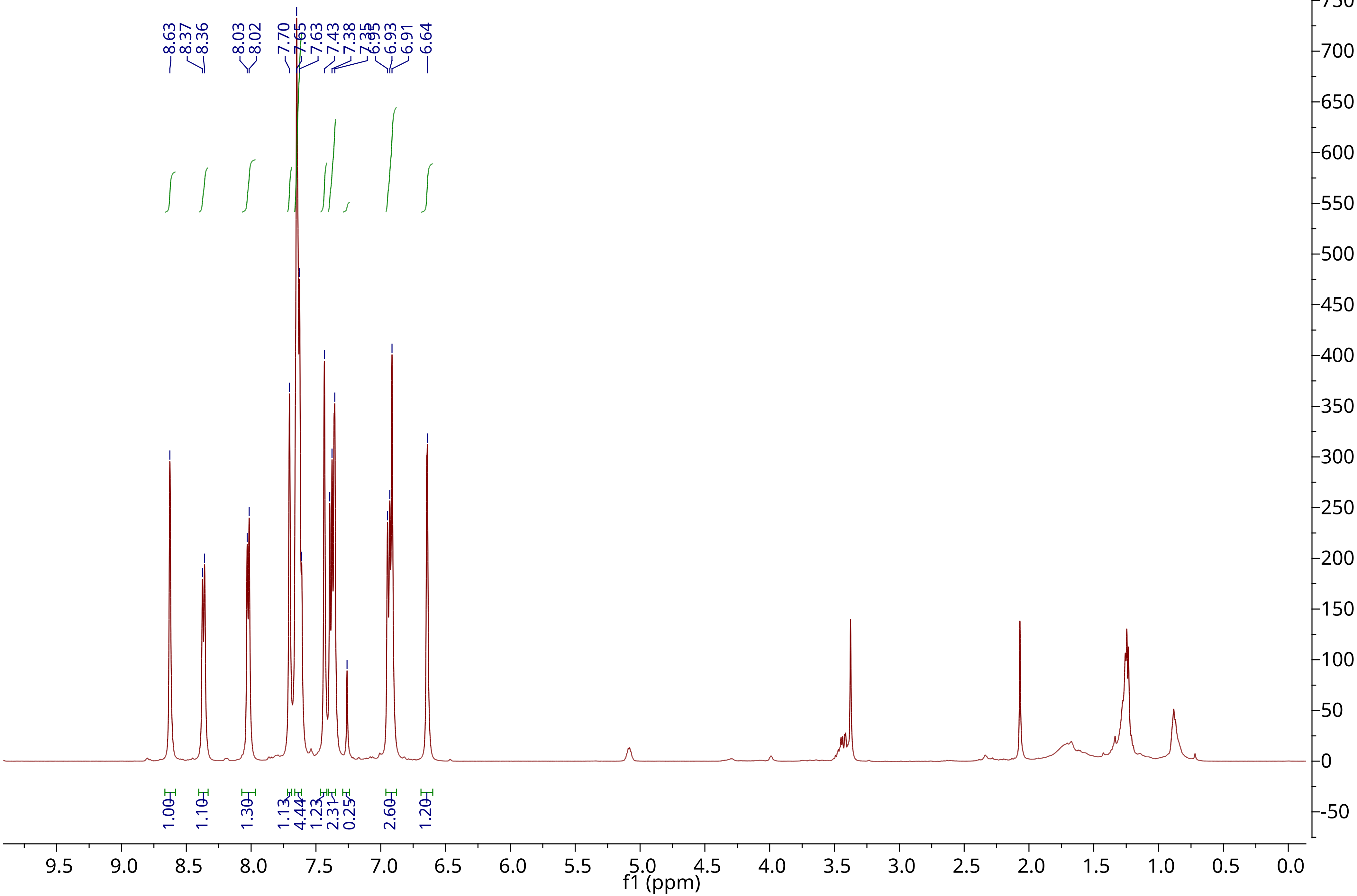

$^{13}C$ NMR
125.5MHz ($CDCl_3$)
PRAN-2.1

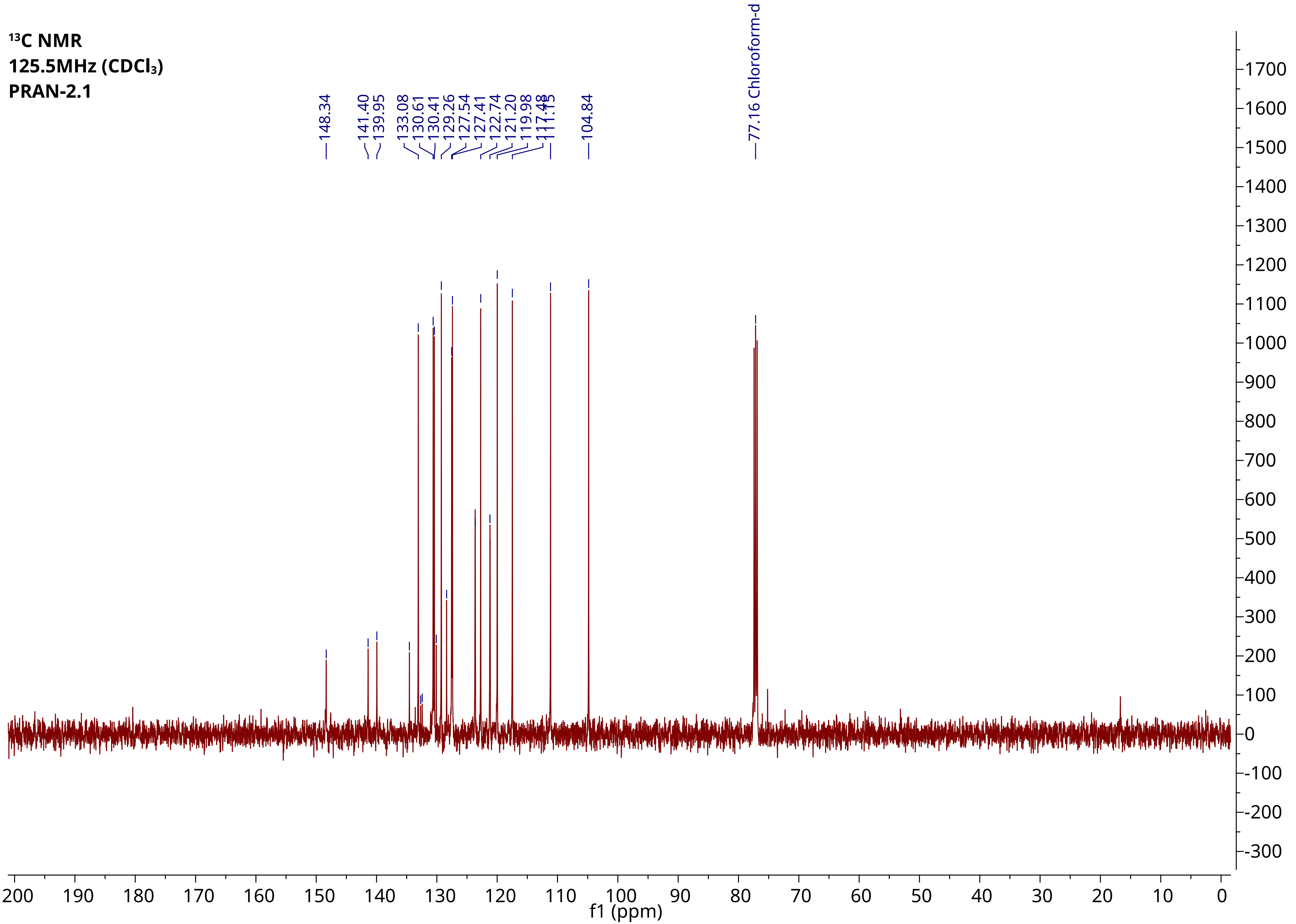

# ==== Shimadzu LCMSsolution Analysis Report ====

**Sample Name : PRAN-2.2**

Method

Column: Purospher RP-8
Mobile Phase A: H2O + 0.9% acetic acid
Mobile Phase B: ACN
% Pump B Concetrate: 50.0
Flow (ml/min): 0.6000

Detector A:SPD-20A
UV_1.Wavelength: 216
UV_2.Wavelength: 264
LC Program

| Time | Unit | Command | Value |
|---|---|---|---|
| 0.01 | Pumps | B.Conc | 50 |
| 15.00 | Pumps | B.Conc | 90 |
| 30.00 | Pumps | B.Conc | 90 |
| 30.01 | Pumps | B.Conc | 50 |
| 40.00 | Controller | Stop | |

MS Chromatogram

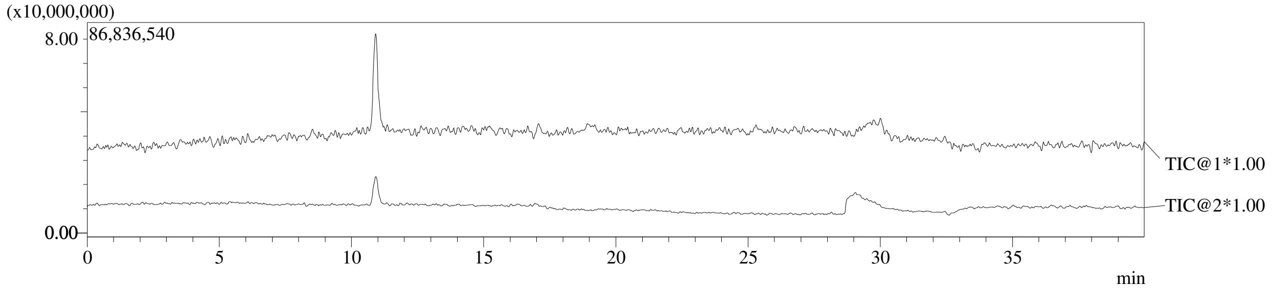


## <LC-UV Chromatogram>

Chromatogram

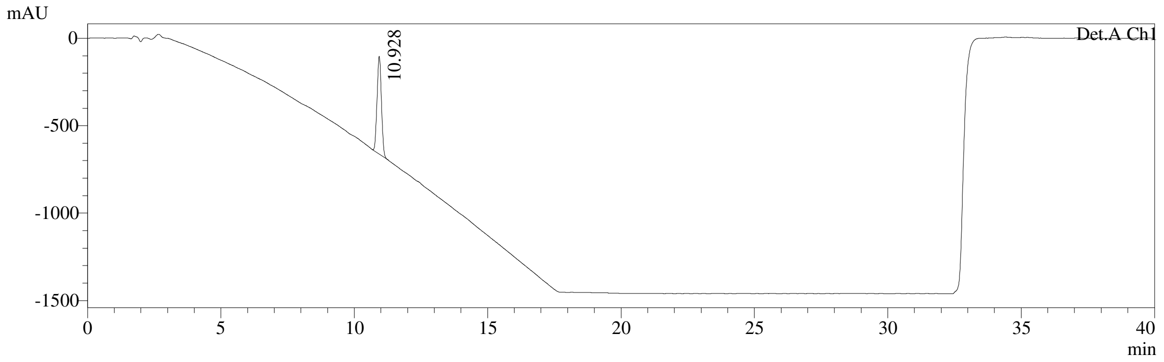

## Sample Name : PRAN-2.2

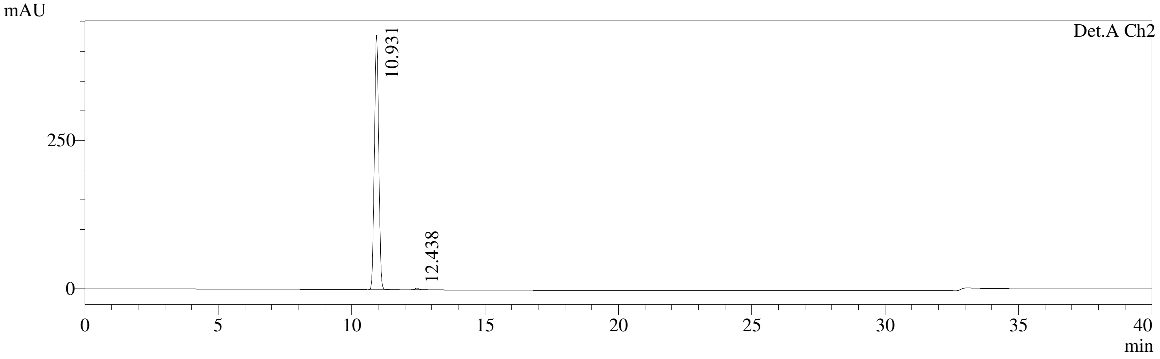


1 Det.A Ch1 / 216nm
2 Det.A Ch2 / 264nm

PeakTable

Detector A Ch2 264nm

| Peak# | Ret. Time | Area | Height | Area % | Height % |
|---|---|---|---|---|---|
| 1 | 10.931 | 4759880 | 428958 | 99.399 | 99.293 |
| 2 | 12.438 | 28782 | 3056 | 0.601 | 0.707 |
| Total | | 4788663 | 432014 | 100.000 | 100.000 |

MS Spectrum Graph

#:1 Ret.Time:Averaged 10.183-11.613(Scan#:941-1073)
BG Mode:Averaged 32.998-39.838(3047-3679)
Mass Peaks:725 Base Peak:275.75(2369678) Polarity:Pos Segment1 - Event1

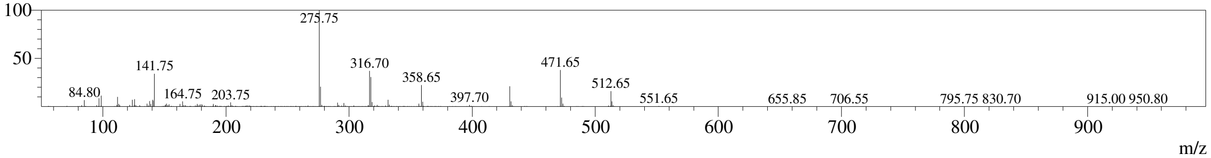


#:2 Ret.Time:Averaged 10.194-11.624(Scan#:942-1074)
BG Mode:Averaged 33.009-39.838(3048-3680)
Mass Peaks:747 Base Peak:428.60(714873) Polarity:Neg Segment1 - Event2

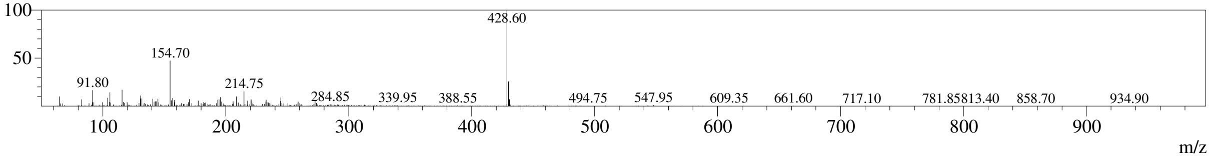

**$^1$H NMR 500MHz ($CDCl_3$)**
**PRAN-2.2**

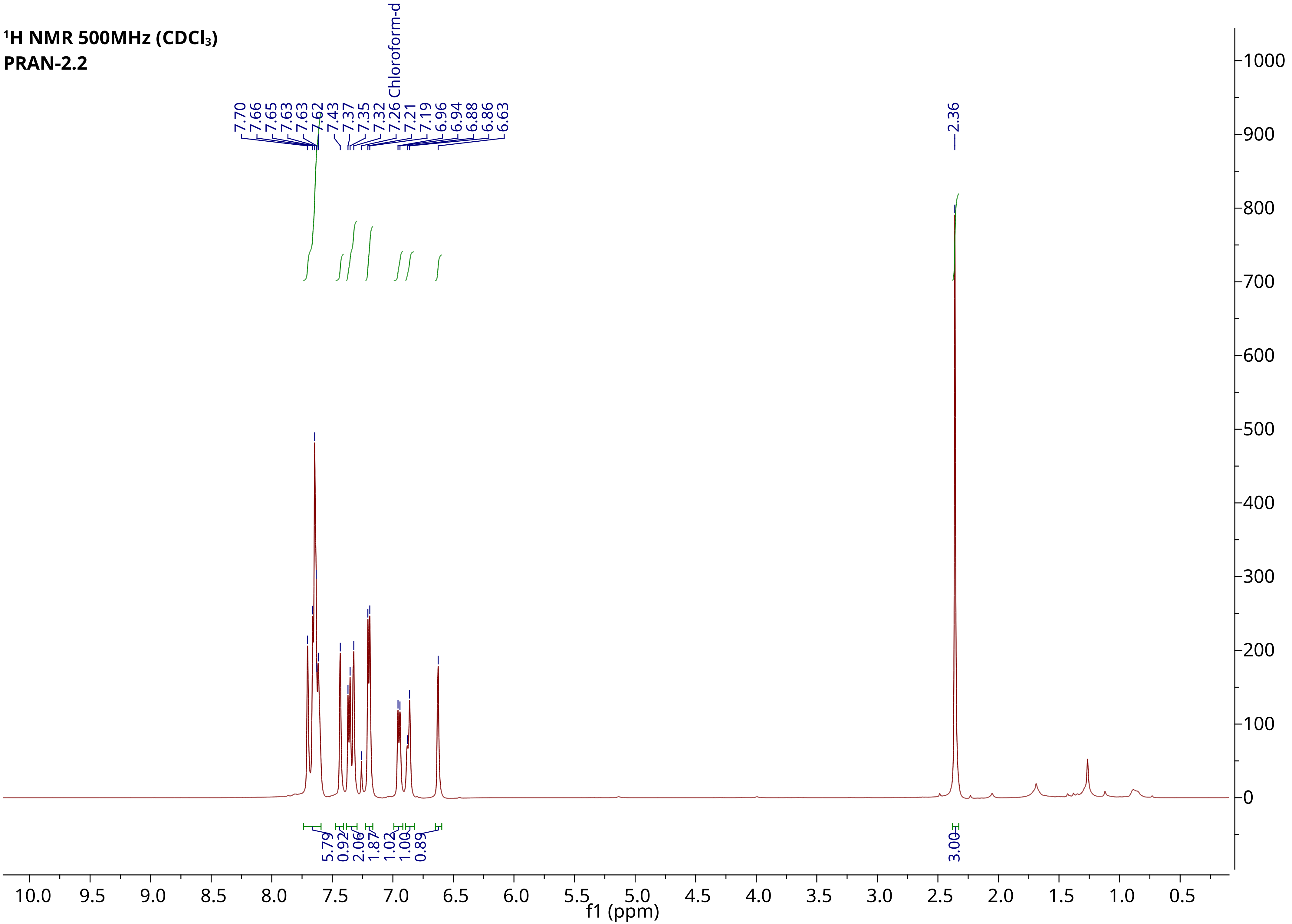

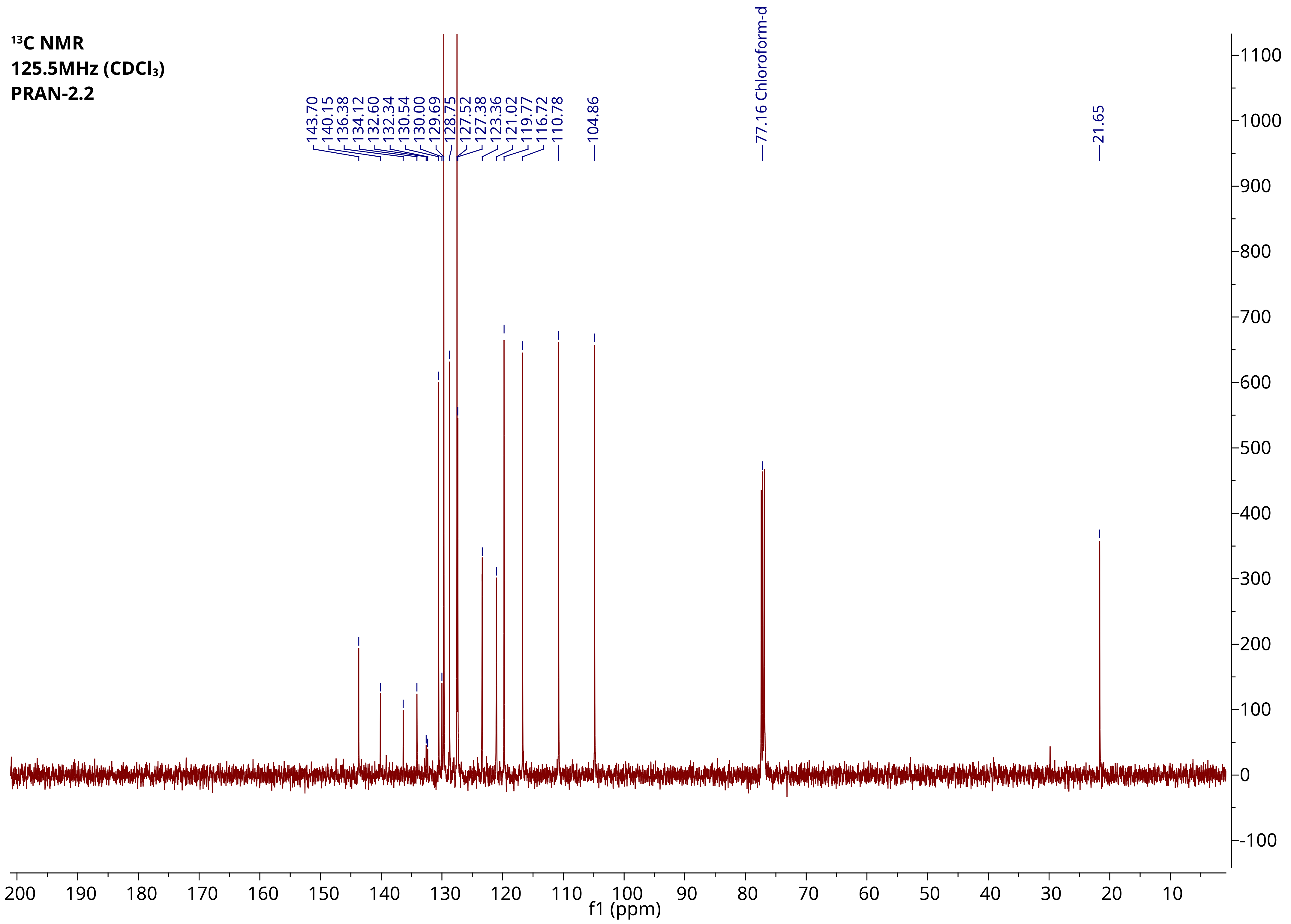

¹³C NMR
125.5MHz (CDCl₃)
PRAN-2.2
143.70
140.15
136.38
134.12
132.60
132.34
130.54
130.00
129.69
128.75
127.52
127.38
123.36
121.02
119.77
116.72
110.78
104.86
77.16 Chloroform-d
21.65
f1 (ppm)

# ==== Shimadzu LCMSsolution Analysis Report ====

**Sample Name : PRAN-2.3**

Method

Column: Purospher RP-8
Mobile Phase A: H2O + 0.9% acetic acid
Mobile Phase B: ACN
% Pump B Concetrate: 50.0
Flow (ml/min): 0.6000

Detector A:SPD-20A
UV_1.Wavelength: 216
UV_2.Wavelength: 264
LC Program

| Time | Unit | Command | Value |
|---|---|---|---|
| 0.01 | Pumps | B.Conc | 50 |
| 15.00 | Pumps | B.Conc | 90 |
| 30.00 | Pumps | B.Conc | 90 |
| 30.01 | Pumps | B.Conc | 50 |
| 40.00 | Controller | Stop | |

MS Chromatogram

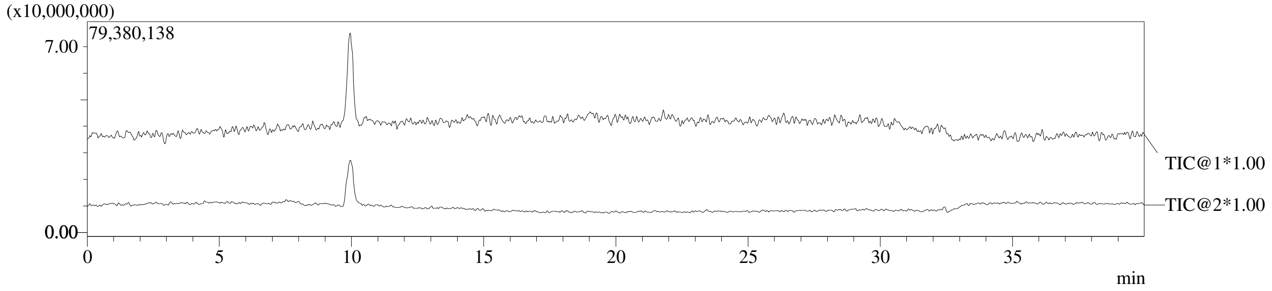


## <LC-UV Chromatogram>

Chromatogram

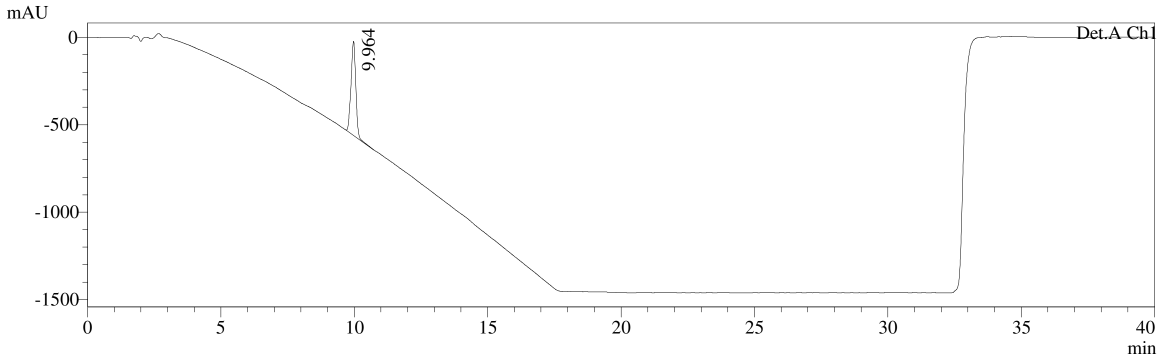

## Sample Name : PRAN-2.3

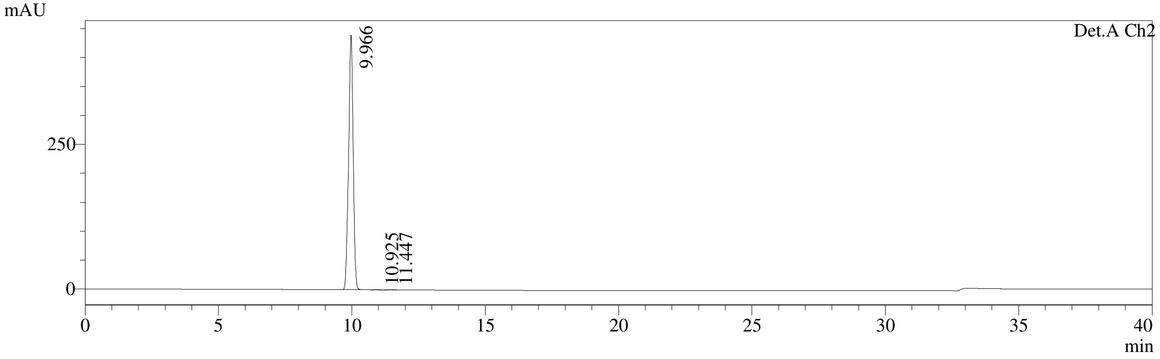


1 Det.A Ch1 / 216nm
2 Det.A Ch2 / 264nm

PeakTable

Detector A Ch2 264nm

| Peak# | Ret. Time | Area | Height | Area % | Height % |
|---|---|---|---|---|---|
| 1 | 9.966 | 5229960 | 440142 | 99.875 | 99.860 |
| 2 | 10.925 | 2646 | 248 | 0.051 | 0.056 |
| 3 | 11.447 | 3911 | 370 | 0.075 | 0.084 |
| Total | | 5236517 | 440760 | 100.000 | 100.000 |

MS Spectrum Graph

#:1 Ret.Time:Averaged 9.533-10.552(Scan#:881-975)
BG Mode:Averaged 23.682-37.472(2187-3459)
Mass Peaks:423 Base Peak:82.90(5905629) Polarity:Pos Segment1 - Event1

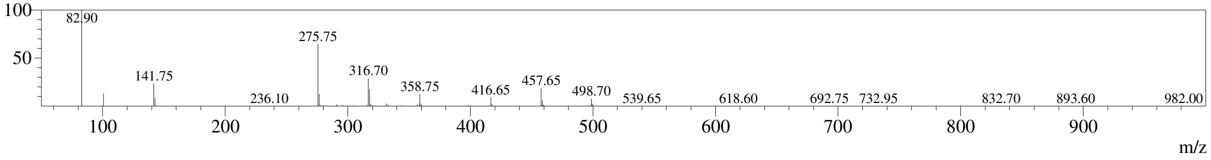


#:2 Ret.Time:Averaged 9.544-10.563(Scan#:882-976)
BG Mode:Averaged 23.693-37.472(2188-3460)
Mass Peaks:584 Base Peak:414.60(2193618) Polarity:Neg Segment1 - Event2

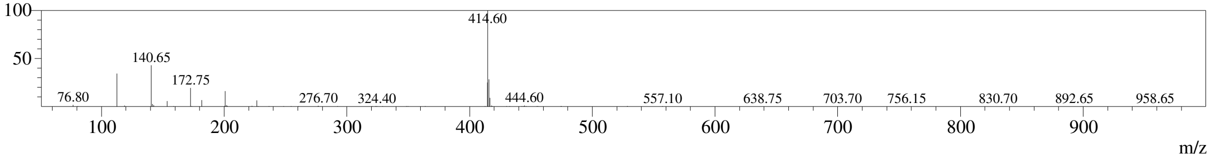

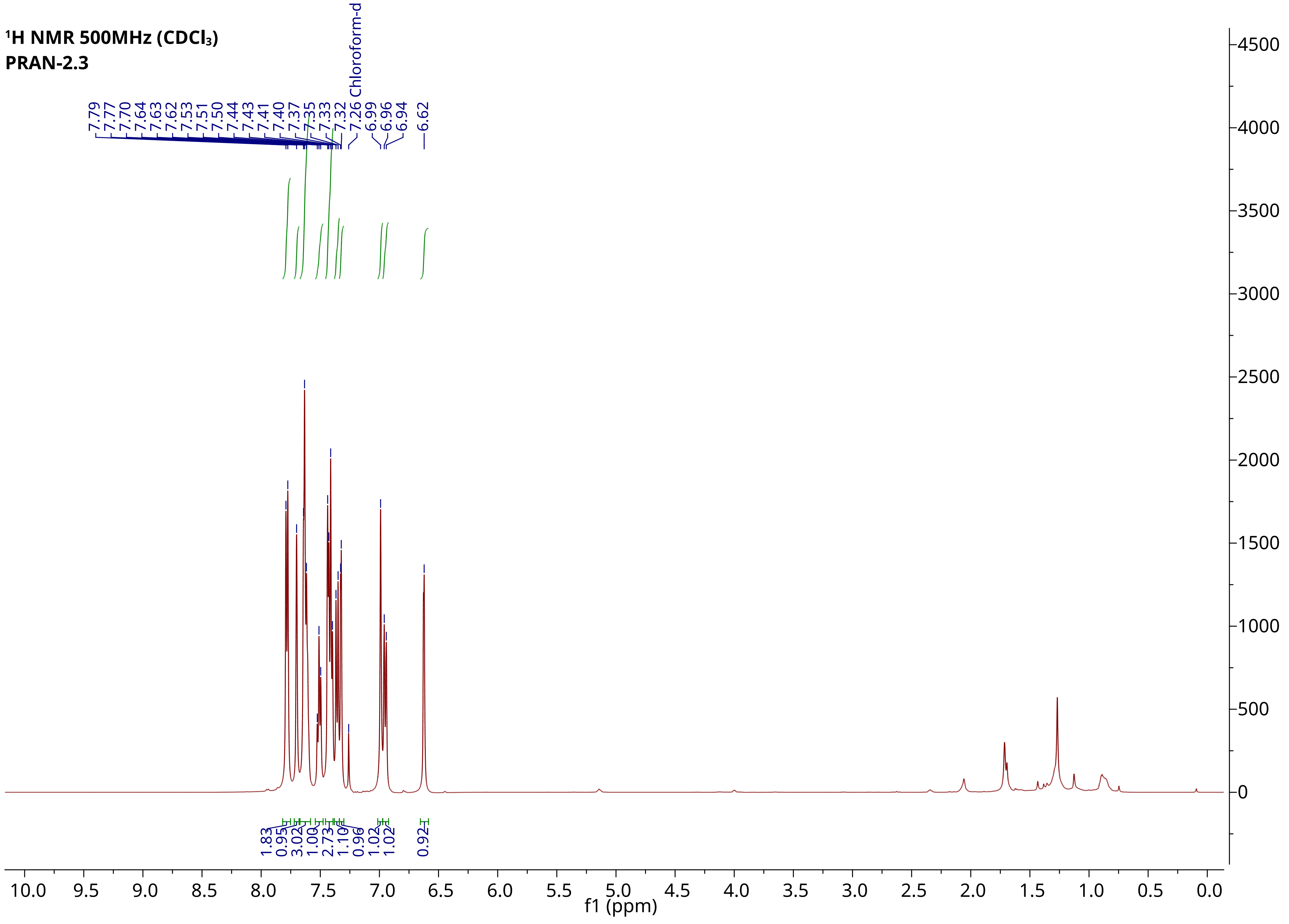

¹H NMR 500MHz (CDCl₃)
PRAN-2.3
7.79
7.77
7.70
7.64
7.63
7.62
7.53
7.51
7.50
7.44
7.43
7.41
7.40
7.37
7.35
7.33
7.32
7.26 Chloroform-d
6.99
6.96
6.94
6.62
1.83
0.95
3.02
1.00
2.73
1.10
0.96
1.02
1.02
0.92
f1 (ppm)

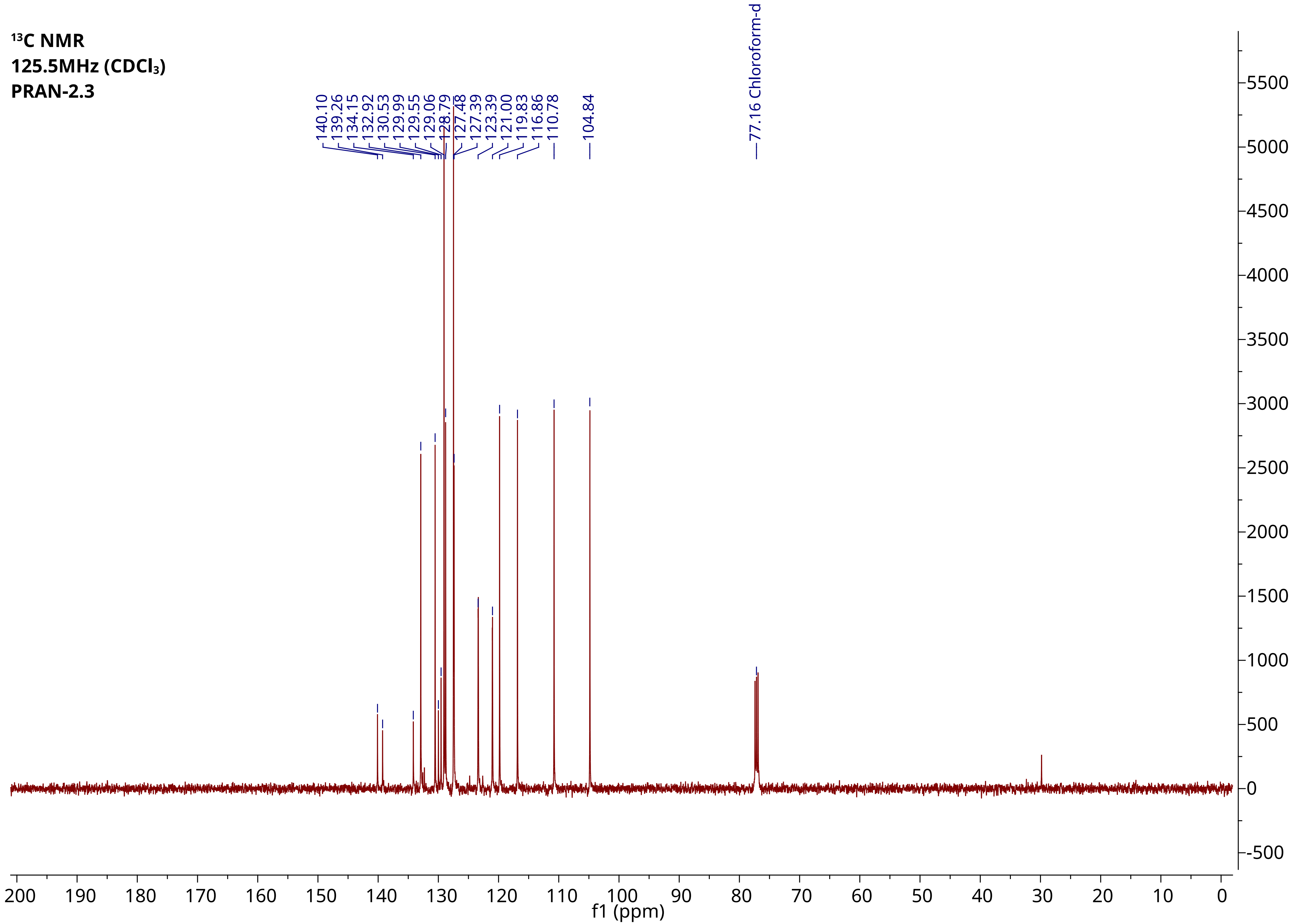

13C NMR
125.5MHz (CDCl3)
PRAN-2.3
140.10
139.26
134.15
132.92
130.53
129.99
129.55
129.06
128.79
127.48
127.39
123.39
121.00
119.83
116.86
110.78
104.84
77.16 Chloroform-d
f1 (ppm)

# ==== Shimadzu LCMSsolution Analysis Report ====

**Sample Name : PRAN-2.4**

Method

Column: Purosphser RP-8
Mobile Phase A: H2O + 0.9% acetic acid
Mobile Phase B: ACN
% Pump B Concetrate: 50.0
Flow (ml/min): 0.6000

Detector A:SPD-20A
UV_1.Wavelength: 216
UV_2.Wavelength: 264
LC Program

| Time | Unit | Command | Value |
|---|---|---|---|
| 0.01 | Pumps | B.Conc | 50 |
| 15.00 | Pumps | B.Conc | 90 |
| 30.00 | Pumps | B.Conc | 90 |
| 30.01 | Pumps | B.Conc | 50 |
| 40.00 | Controller | Stop | |

MS Chromatogram

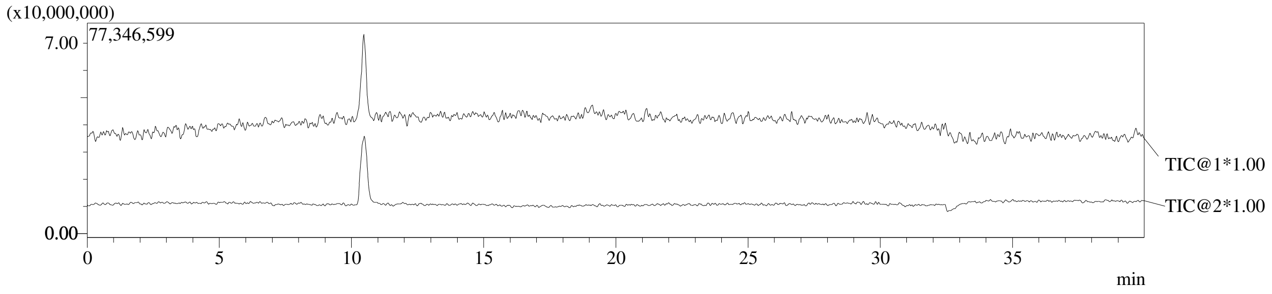


## <LC-UV Chromatogram>

Chromatogram

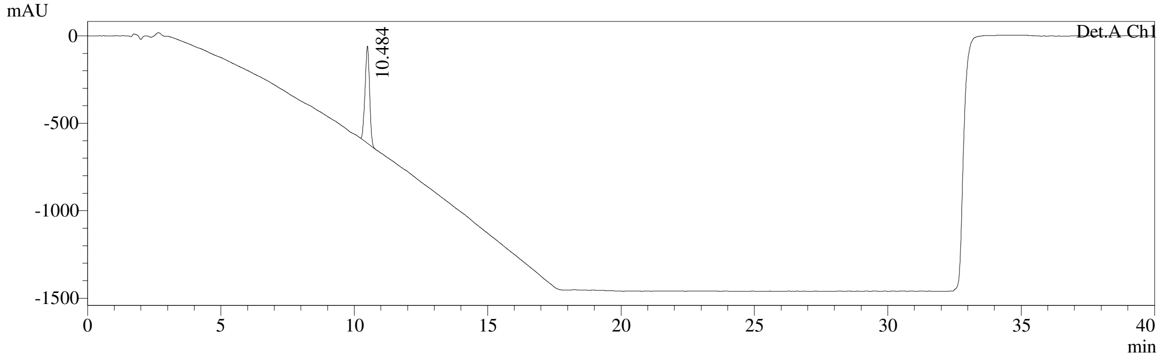

## Sample Name : PRAN-2.4

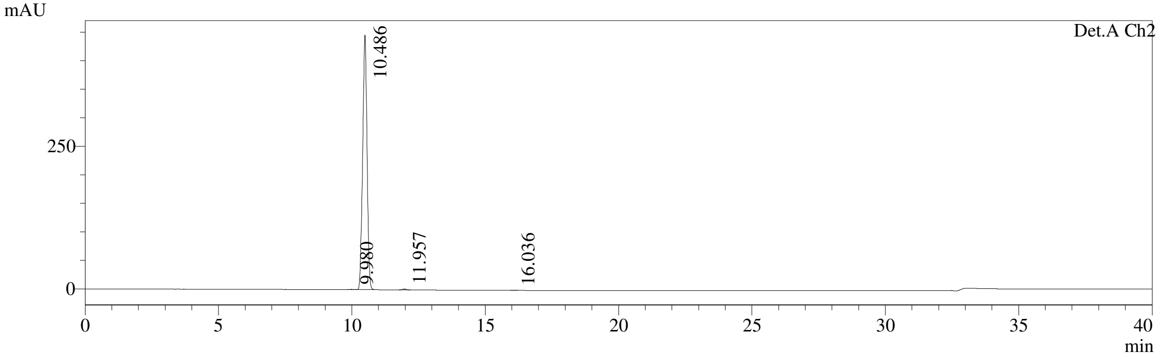


1 Det.A Ch1 / 216nm
2 Det.A Ch2 / 264nm

PeakTable

Detector A Ch2 264nm

| Peak# | Ret. Time | Area | Height | Area % | Height % |
|---|---|---|---|---|---|
| 1 | 9.980 | 4515 | 459 | 0.088 | 0.102 |
| 2 | 10.486 | 5094797 | 445878 | 99.530 | 99.425 |
| 3 | 11.957 | 17181 | 1777 | 0.336 | 0.396 |
| 4 | 16.036 | 2364 | 345 | 0.046 | 0.077 |
| Total | | 5118857 | 448458 | 100.000 | 100.000 |

MS Spectrum Graph

#:1 Ret.Time:Averaged 10.010-11.158(Scan#:925-1031)
BG Mode:Averaged 24.678-38.893(2279-3591)
Mass Peaks:516 Base Peak:82.85(5908891) Polarity:Pos Segment1 - Event1

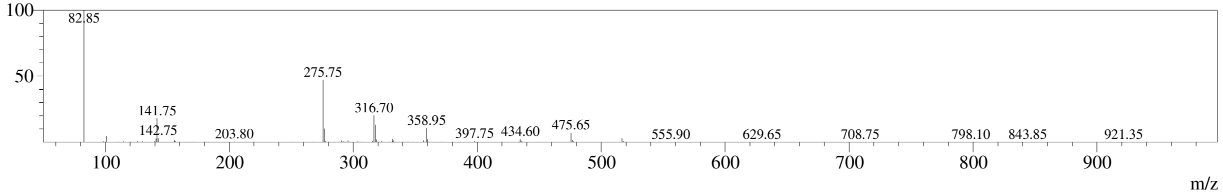


#:2 Ret.Time:Averaged 10.021-11.169(Scan#:926-1032)
BG Mode:Averaged 24.689-38.893(2280-3592)
Mass Peaks:540 Base Peak:432.60(3188893) Polarity:Neg Segment1 - Event2

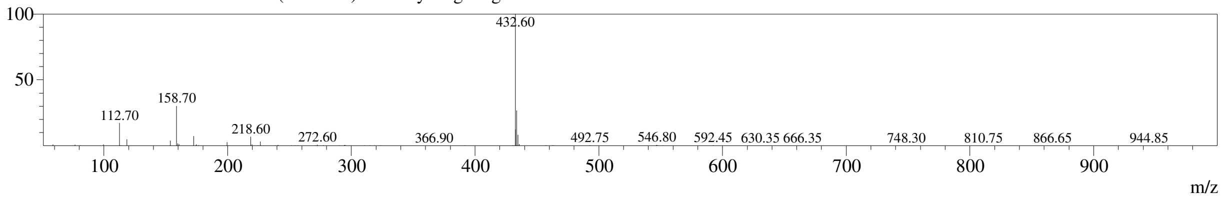

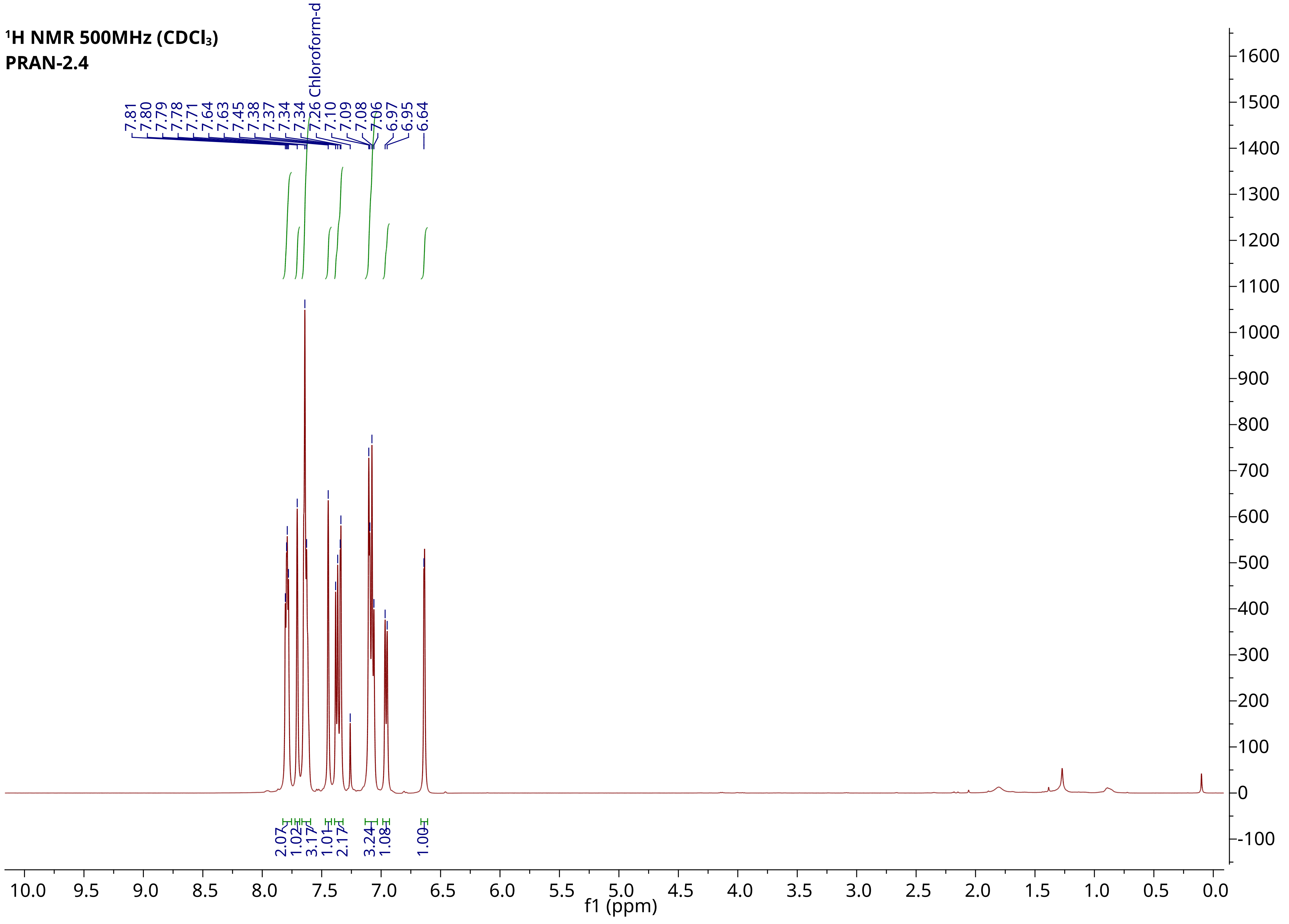

¹H NMR 500MHz (CDCl₃)
PRAN-2.4
7.81
7.80
7.79
7.78
7.71
7.64
7.63
7.45
7.38
7.37
7.34
7.34
7.26 Chloroform-d
7.10
7.09
7.08
7.06
6.97
6.95
6.64
2.07
1.02
3.17
1.01
2.17
3.24
1.08
1.00
f1 (ppm)

**$^{13}C$ NMR**
**125.5MHz ($CDCl_3$)**
**PRAN-2.4**

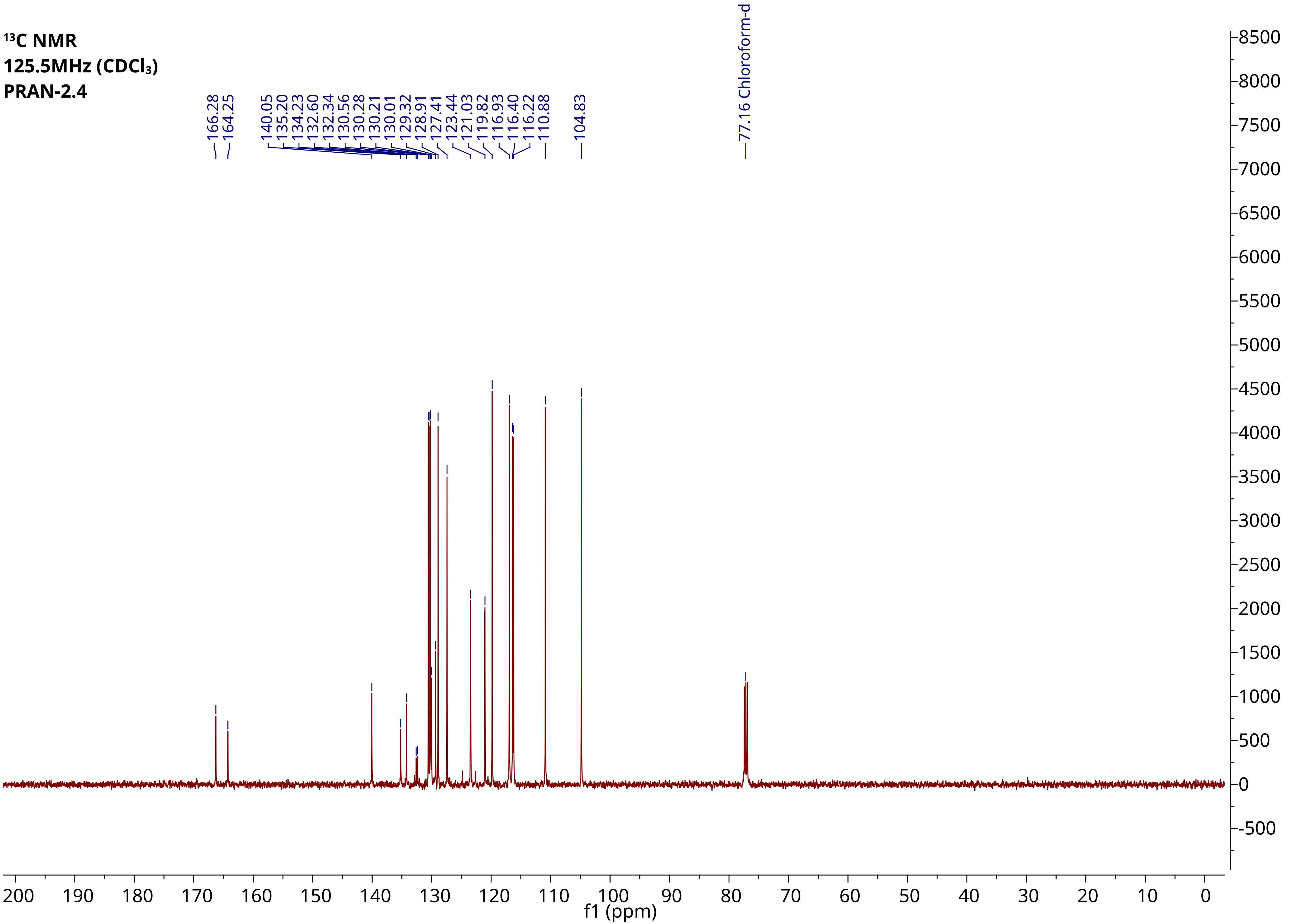

# ==== Shimadzu LCMSsolution Analysis Report ====

**Sample Name : PRAN-2.5**

Method

Column: Purospher RP-8
Mobile Phase A: H2O + 0.9% acetic acid
Mobile Phase B: ACN
% Pump B Concetrate: 50.0
Flow (ml/min): 0.6000

Detector A:SPD-20A
UV_1.Wavelength: 216
UV_2.Wavelength: 264
LC Program

| Time | Unit | Command | Value |
|---|---|---|---|
| 0.01 | Pumps | B.Conc | 50 |
| 15.00 | Pumps | B.Conc | 90 |
| 30.00 | Pumps | B.Conc | 90 |
| 30.01 | Pumps | B.Conc | 50 |
| 40.00 | Controller | Stop | |

MS Chromatogram

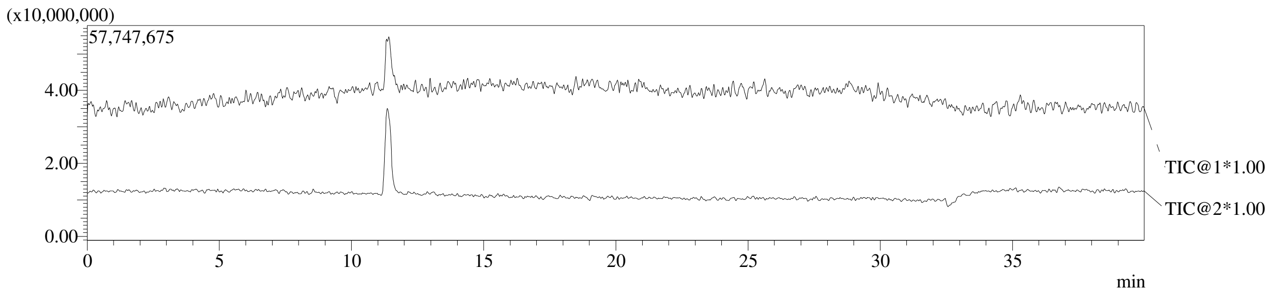


## <LC-UV Chromatogram>

Chromatogram

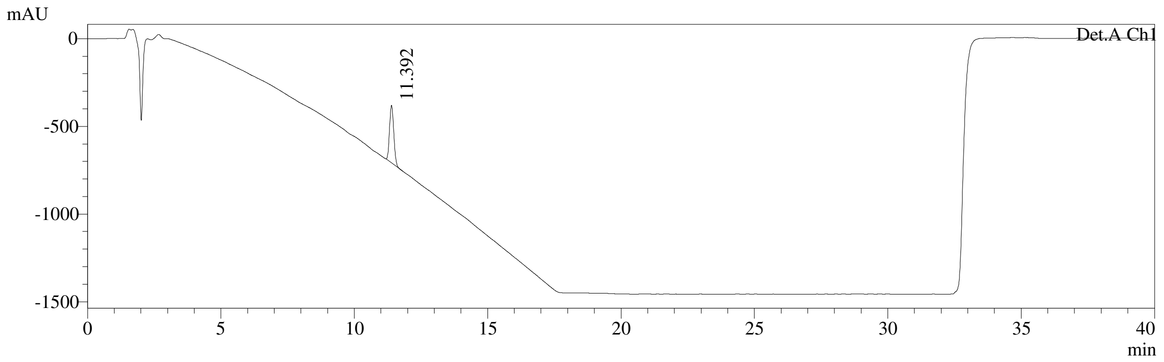

## Sample Name : PRAN-2.5

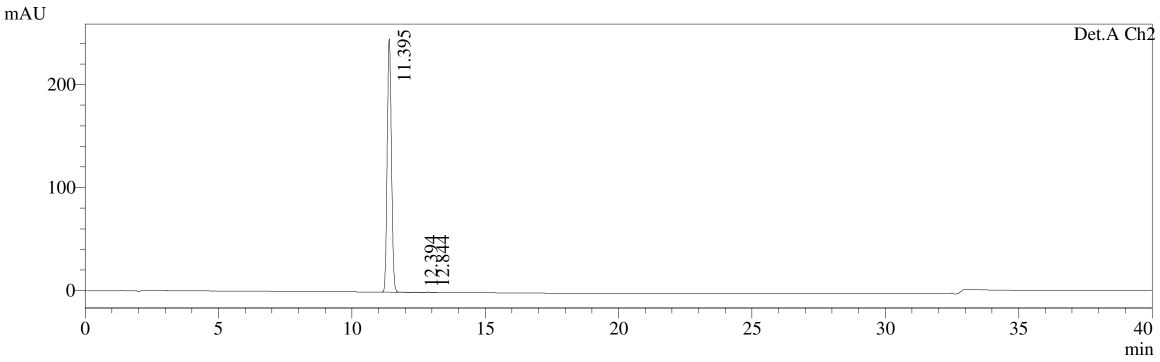


1 Det.A Ch1 / 216nm
2 Det.A Ch2 / 264nm

PeakTable

Detector A Ch2 264nm

| Peak# | Ret. Time | Area | Height | Area % | Height % |
|---|---|---|---|---|---|
| 1 | 11.395 | 2706107 | 246234 | 99.815 | 99.803 |
| 2 | 12.394 | 1357 | 100 | 0.050 | 0.041 |
| 3 | 12.844 | 3645 | 385 | 0.134 | 0.156 |
| Total | | 2711109 | 246719 | 100.000 | 100.000 |

MS Spectrum Graph

#:1 Ret.Time:Averaged 11.028-11.765(Scan#:1019-1087)
BG Mode:Averaged 25.848-39.231(2387-3623)
Mass Peaks:544 Base Peak:82.85(6490438) Polarity:Pos Segment1 - Event1

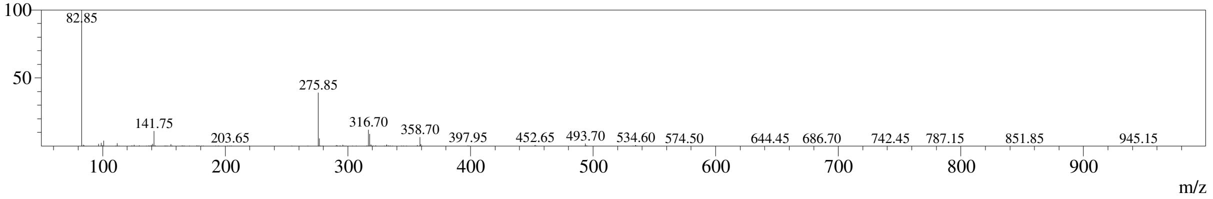


#:2 Ret.Time:Averaged 11.039-11.776(Scan#:1020-1088)
BG Mode:Averaged 25.859-39.231(2388-3624)
Mass Peaks:632 Base Peak:450.55(3061814) Polarity:Neg Segment1 - Event2

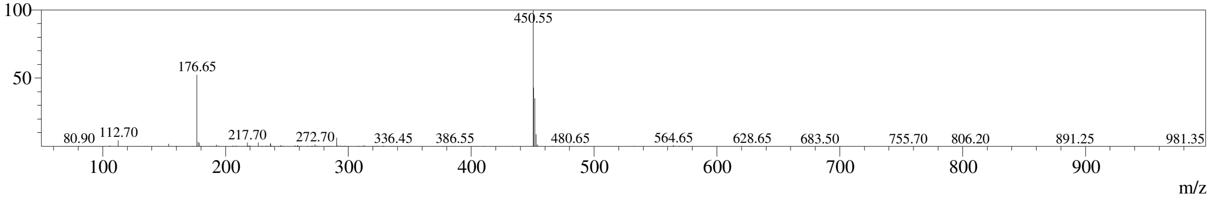

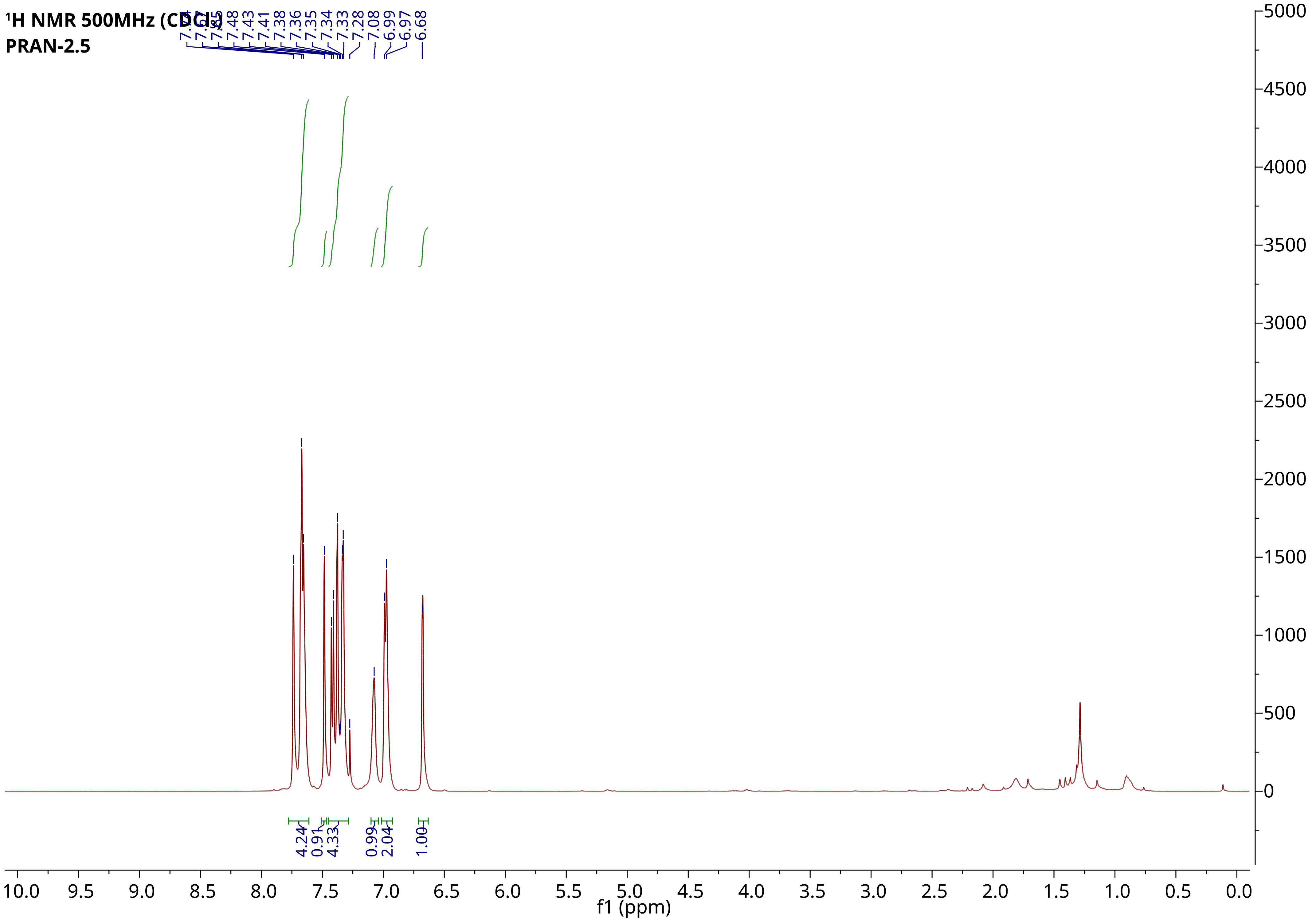

¹H NMR 500MHz (CDCl3)
PRAN-2.5
7.74
7.67
7.65
7.48
7.43
7.41
7.38
7.36
7.35
7.34
7.33
7.28
7.08
6.99
6.97
6.68
4.24
0.91
4.33
0.99
2.04
1.00
f1 (ppm)

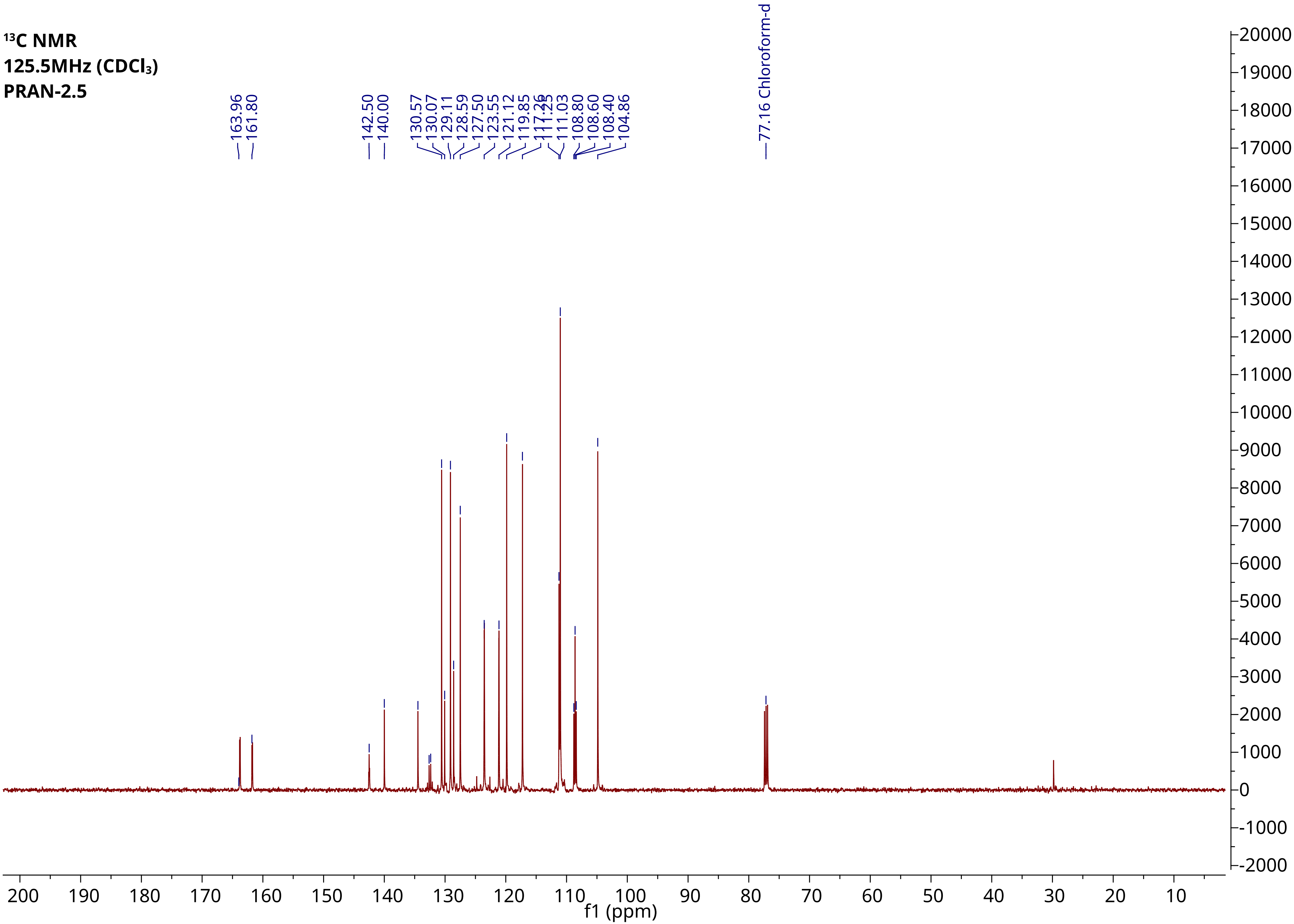

$^{13}$C NMR
125.5MHz (CDCl$_3$)
PRAN-2.5
163.96
161.80
142.50
140.00
130.57
130.07
129.11
128.59
127.50
123.55
121.12
119.85
111.26
111.25
111.03
108.80
108.60
108.40
104.86
77.16 Chloroform-d
f1 (ppm)

# ==== Shimadzu LCMSsolution Analysis Report ====

**Sample Name : PRAN-2.6**

Method

Column: Purospher RP-8
Mobile Phase A: $H2O$ + 0.9% acetic acid
Mobile Phase B: ACN
% Pump B Concetrate: 50.0
Flow (ml/min): 0.6000

Detector A:SPD-20A
UV_1.Wavelength: 216
UV_2.Wavelength: 264
LC Program

| Time | Unit | Command | Value |
|---|---|---|---|
| 0.01 | Pumps | B.Conc | 50 |
| 15.00 | Pumps | B.Conc | 90 |
| 30.00 | Pumps | B.Conc | 90 |
| 30.01 | Pumps | B.Conc | 50 |
| 40.00 | Controller | Stop | |

MS Chromatogram

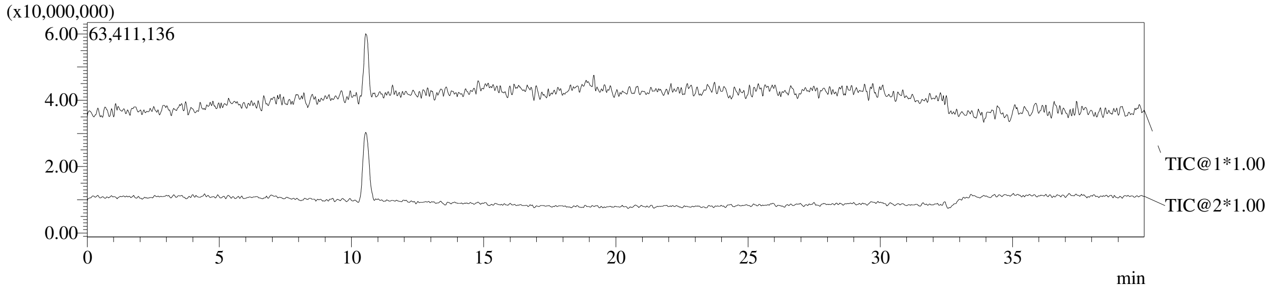


## <LC-UV Chromatogram>

Chromatogram

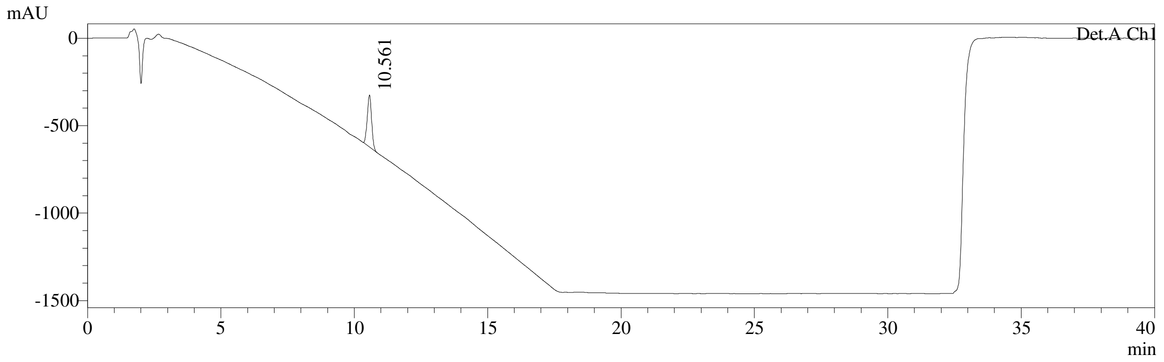

## Sample Name : PRAN-2.6

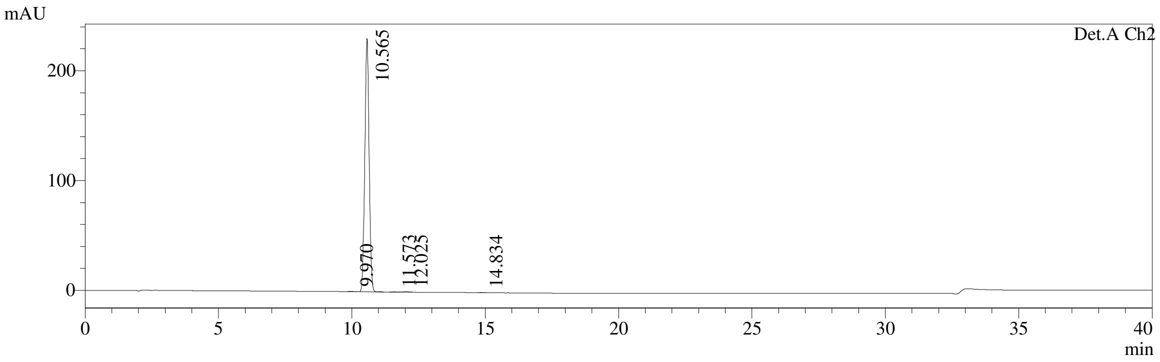


1 Det.A Ch1 / 216nm
2 Det.A Ch2 / 264nm

PeakTable

Detector A Ch2 264nm

| Peak# | Ret. Time | Area | Height | Area % | Height % |
|---|---|---|---|---|---|
| 1 | 9.970 | 2415 | 206 | 0.092 | 0.089 |
| 2 | 10.565 | 2611857 | 230800 | 99.631 | 99.582 |
| 3 | 11.573 | 1536 | 138 | 0.059 | 0.060 |
| 4 | 12.025 | 4536 | 480 | 0.173 | 0.207 |
| 5 | 14.834 | 1178 | 145 | 0.045 | 0.062 |
| Total | | 2621524 | 231768 | 100.000 | 100.000 |

MS Spectrum Graph

#:1 Ret.Time:Averaged 10.140-11.158(Scan#:937-1031)
BG Mode:Averaged 26.585-39.637(2455-3659)
Mass Peaks:415 Base Peak:82.85(7187583) Polarity:Pos Segment1 - Event1

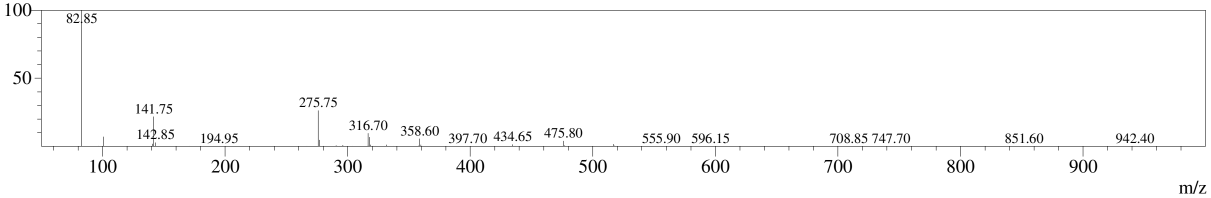


#:2 Ret.Time:Averaged 10.151-11.169(Scan#:938-1032)
BG Mode:Averaged 26.596-39.637(2456-3660)
Mass Peaks:470 Base Peak:432.60(2999861) Polarity:Neg Segment1 - Event2

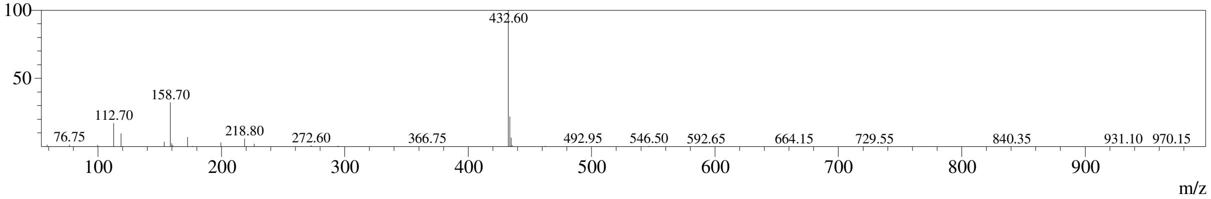

$^1$H NMR 500MHz ($CDCl_3$)
**PRAN-2.6**

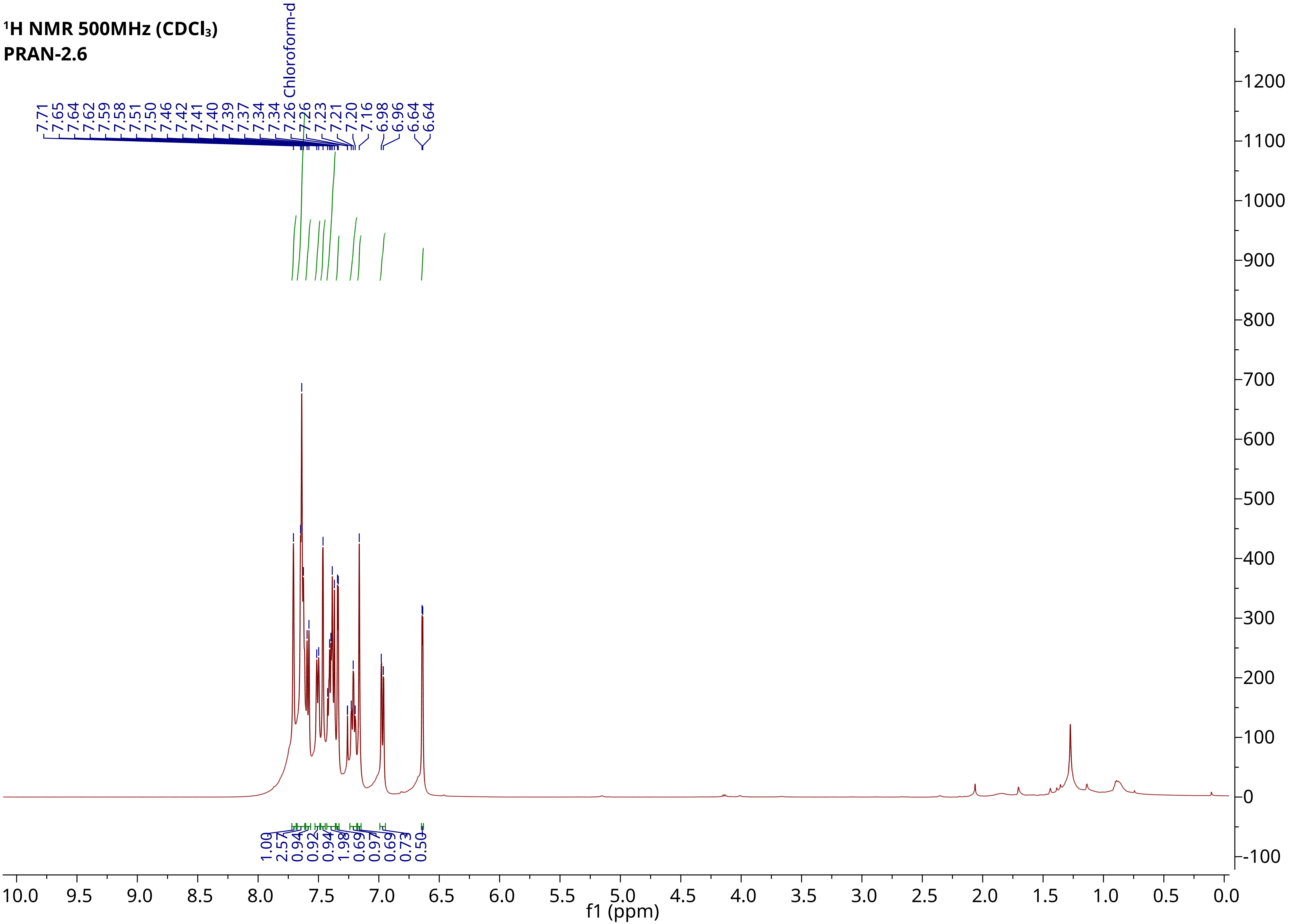

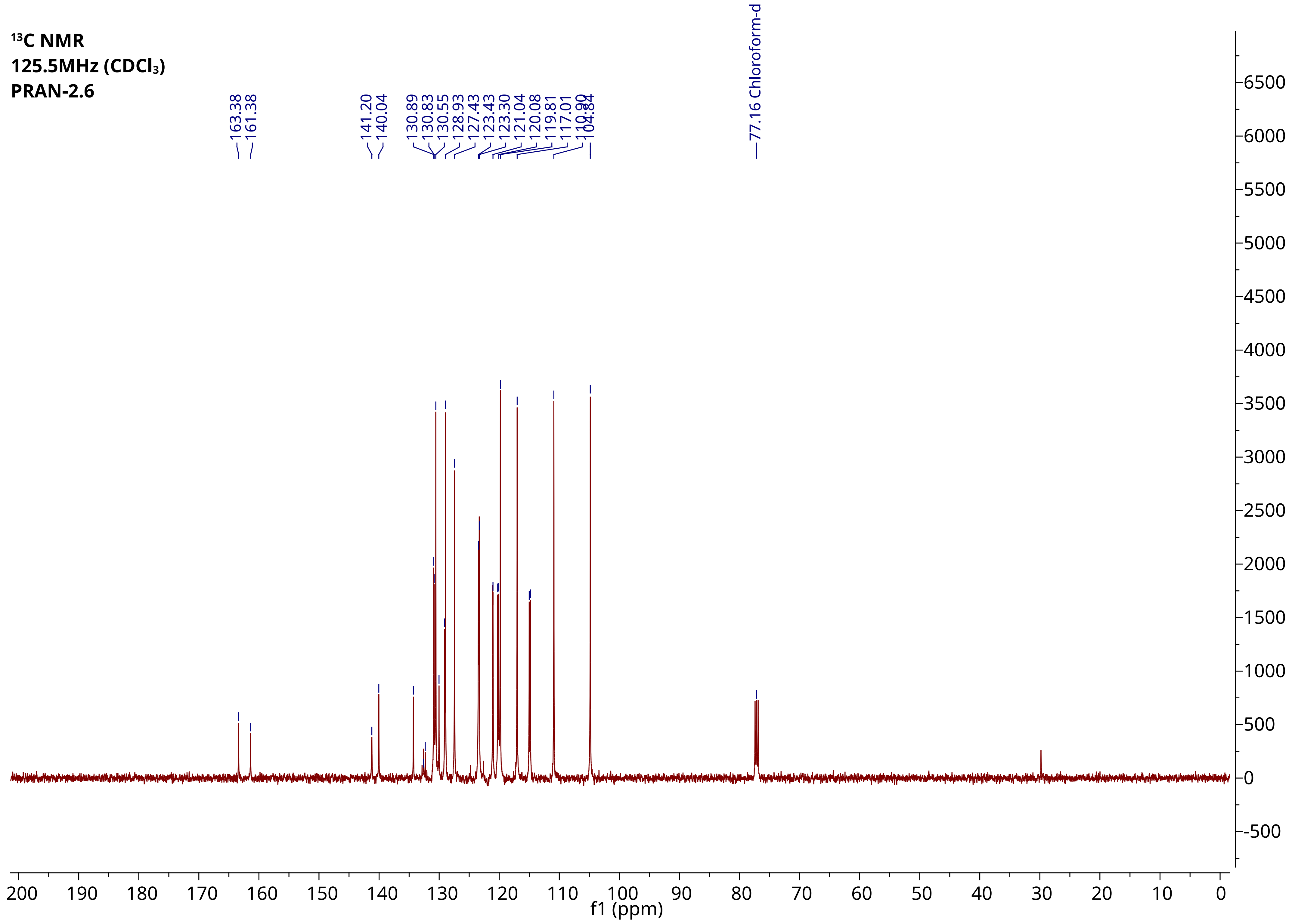

$^{13}C$ NMR
125.5MHz ($CDCl_3$)
PRAN-2.6
163.38
161.38
141.20
140.04
130.89
130.83
130.55
128.93
127.43
123.43
123.30
121.04
120.08
119.81
117.01
110.90
104.84
77.16 Chloroform-d
f1 (ppm)

# ==== Shimadzu LCMSsolution Analysis Report ====

**Sample Name : PRAN-2.7**

Method

Column: Purospher RP-8
Mobile Phase A: H2O + 0.9% acetic acid
Mobile Phase B: ACN
% Pump B Concetrate: 50.0
Flow (ml/min): 0.6000

Detector A:SPD-20A
UV_1.Wavelength: 216
UV_2.Wavelength: 264
LC Program

| Time | Unit | Command | Value |
|---|---|---|---|
| 0.01 | Pumps | B.Conc | 50 |
| 15.00 | Pumps | B.Conc | 90 |
| 30.00 | Pumps | B.Conc | 90 |
| 30.01 | Pumps | B.Conc | 50 |
| 40.00 | Controller | Stop | |

MS Chromatogram

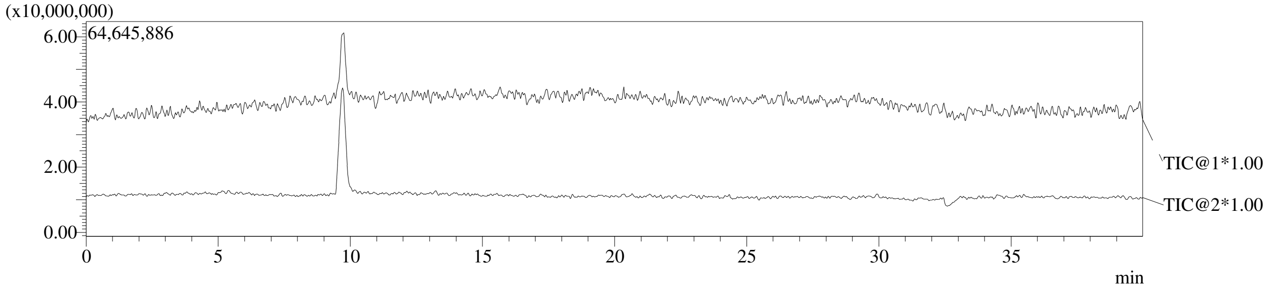


## <LC-UV Chromatogram>

Chromatogram

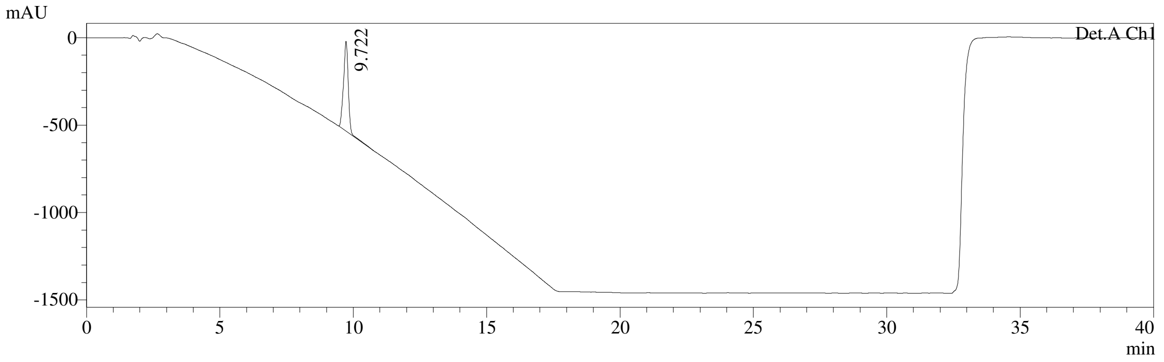

## Sample Name : PRAN-2.7

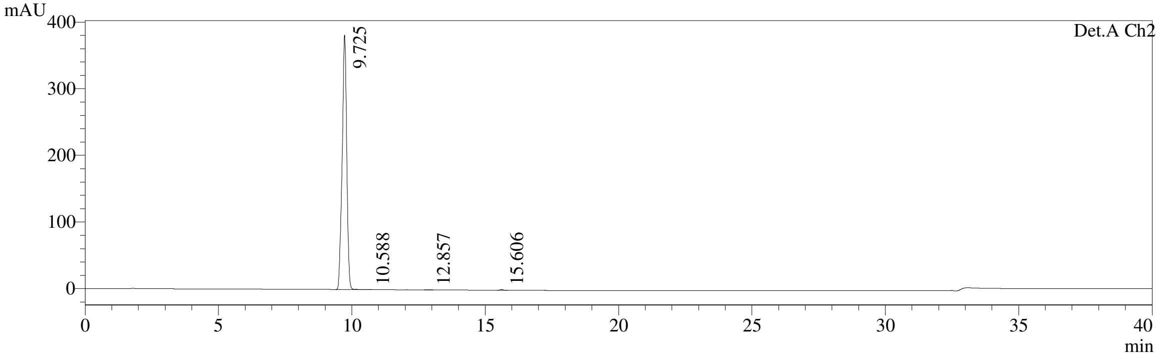


1 Det.A Ch1 / 216nm
2 Det.A Ch2 / 264nm

PeakTable

Detector A Ch2 264nm

| Peak# | Ret. Time | Area | Height | Area % | Height % |
|---|---|---|---|---|---|
| 1 | 9.725 | 4554257 | 381764 | 99.771 | 99.642 |
| 2 | 10.588 | 1029 | 124 | 0.023 | 0.032 |
| 3 | 12.857 | 1986 | 238 | 0.043 | 0.062 |
| 4 | 15.606 | 7430 | 1012 | 0.163 | 0.264 |
| Total | | 4564702 | 383137 | 100.000 | 100.000 |

MS Spectrum Graph

#:1 Ret.Time:Averaged 9.273-10.010(Scan#:857-925)
BG Mode:Averaged 20.432-38.149(1887-3523)
Mass Peaks:477 Base Peak:82.85(9838791) Polarity:Pos Segment1 - Event1

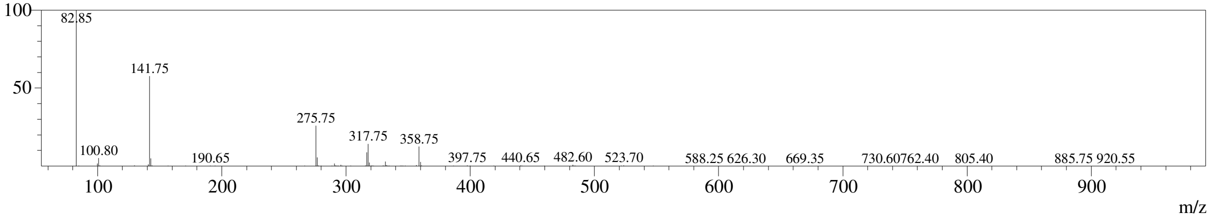


#:2 Ret.Time:Averaged 9.284-10.021(Scan#:858-926)
BG Mode:Averaged 20.443-38.149(1888-3524)
Mass Peaks:599 Base Peak:439.60(5577977) Polarity:Neg Segment1 - Event2

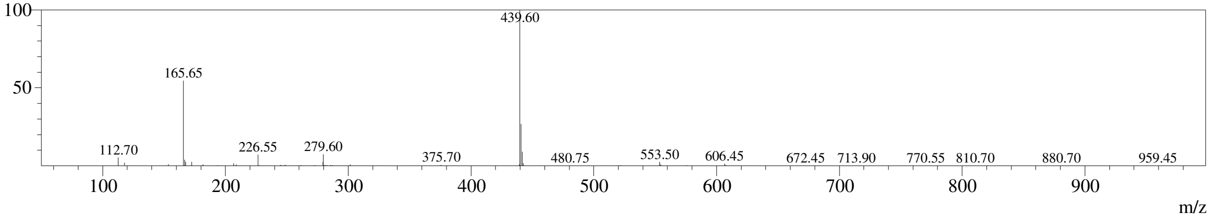

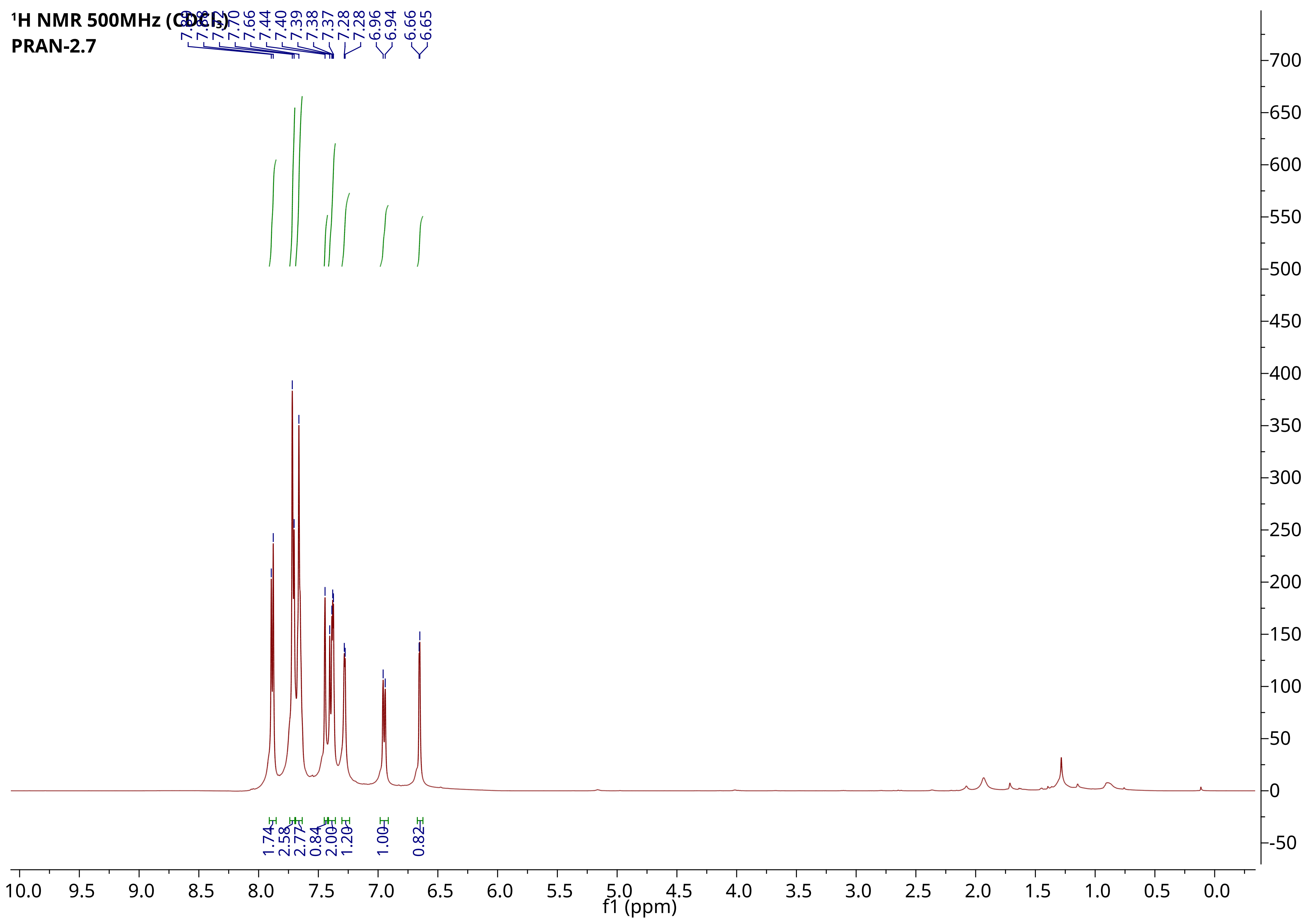

**$^{13}$C NMR**
**125.5MHz ($CDCl_3$)**
**PRAN-2.7**

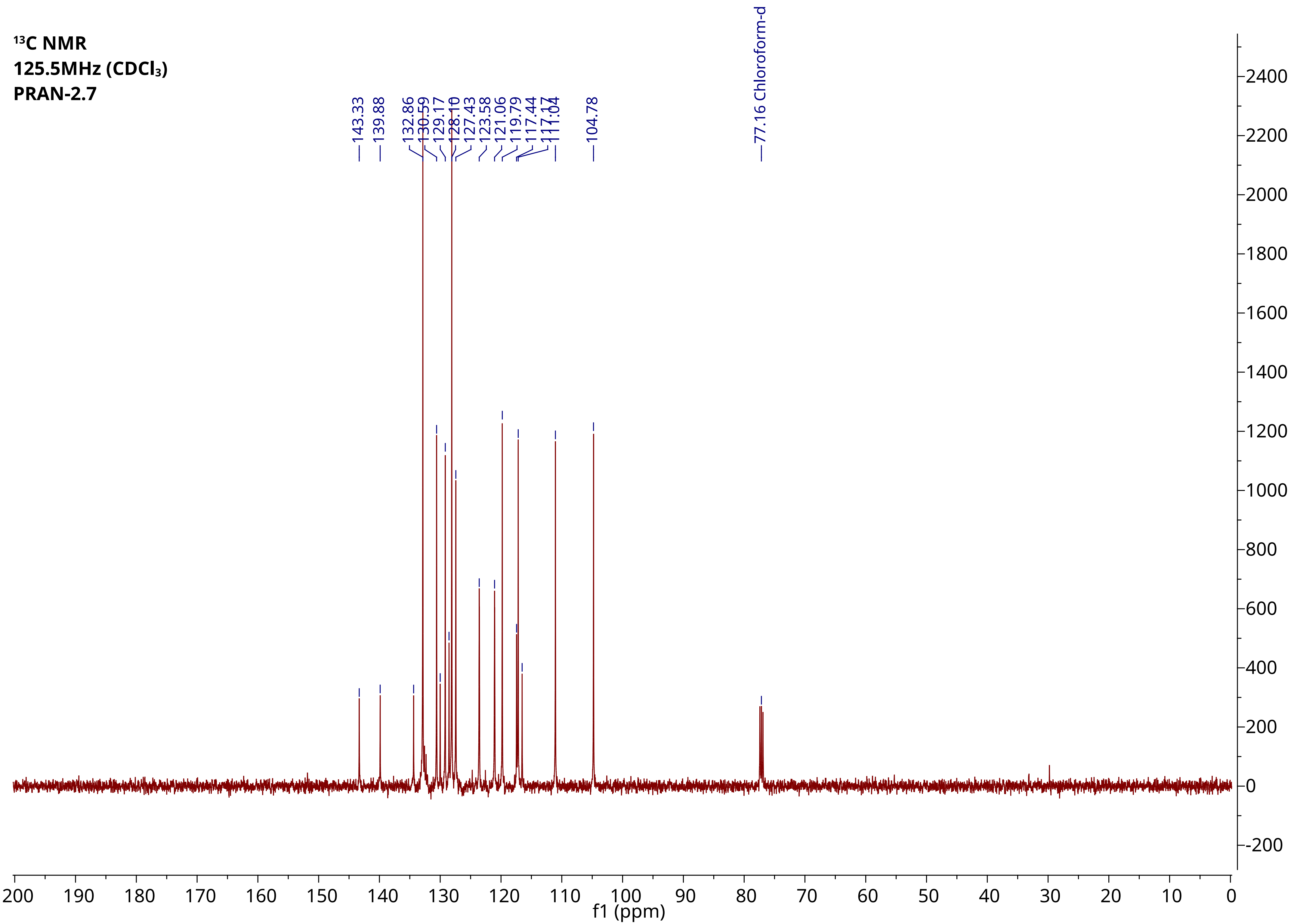

# ==== Shimadzu LCMSsolution Analysis Report ====

**Sample Name : PRAN-2.8**

Method

Column: Purospher RP-8
Mobile Phase A: H2O + 0.9% acetic acid
Mobile Phase B: ACN
% Pump B Concetrate: 50.0
Flow (ml/min): 0.6000

Detector A:SPD-20A
UV_1.Wavelength: 216
UV_2.Wavelength: 264
LC Program

| Time | Unit | Command | Value |
|---|---|---|---|
| 0.01 | Pumps | B.Conc | 50 |
| 15.00 | Pumps | B.Conc | 90 |
| 30.00 | Pumps | B.Conc | 90 |
| 30.01 | Pumps | B.Conc | 50 |
| 40.00 | Controller | Stop | |

MS Chromatogram

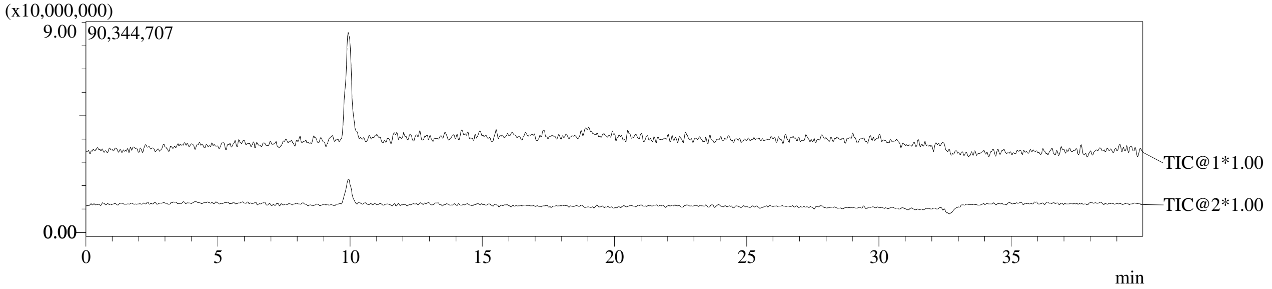


## <LC-UV Chromatogram>

Chromatogram

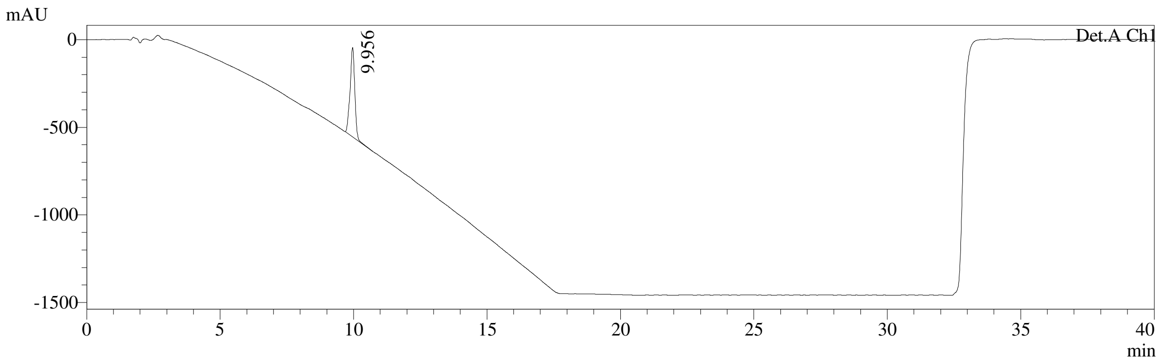

## Sample Name : PRAN-2.8

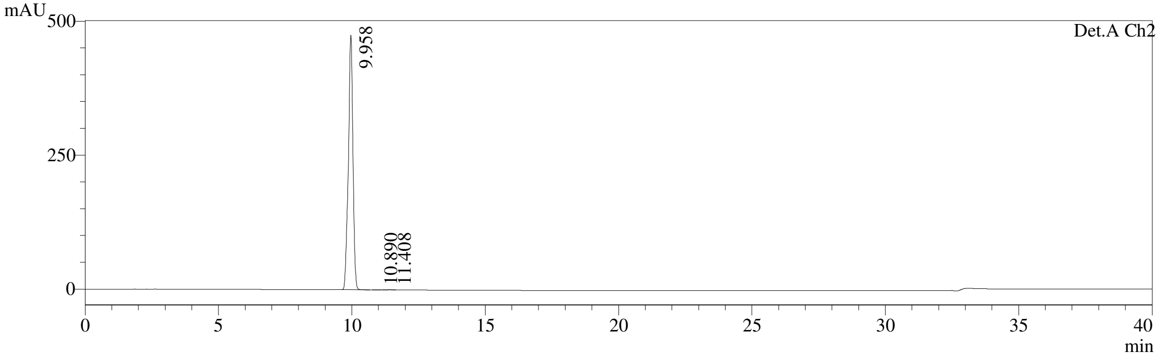


1 Det.A Ch1 / 216nm
2 Det.A Ch2 / 264nm

PeakTable

Detector A Ch2 264nm

| Peak# | Ret. Time | Area | Height | Area % | Height % |
|---|---|---|---|---|---|
| 1 | 9.958 | 5655359 | 475196 | 99.887 | 99.878 |
| 2 | 10.890 | 1348 | 136 | 0.024 | 0.029 |
| 3 | 11.408 | 5075 | 442 | 0.090 | 0.093 |
| Total | | 5661782 | 475775 | 100.000 | 100.000 |

MS Spectrum Graph

#:1 Ret.Time:Averaged 9.165-10.357(Scan#:847-957)
BG Mode:Averaged 21.212-24.189(1959-2233)
Mass Peaks:507 Base Peak:275.70(3185221) Polarity:Pos Segment1 - Event1

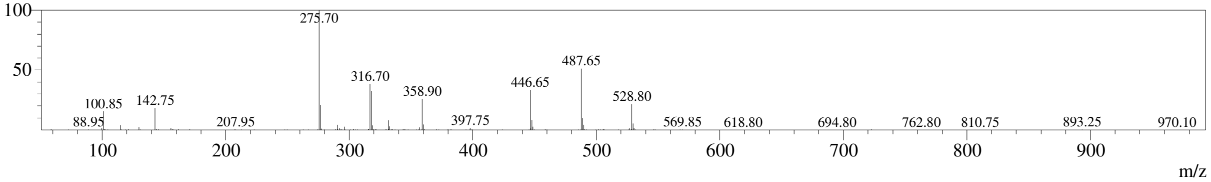


#:2 Ret.Time:Averaged 9.176-10.368(Scan#:848-958)
BG Mode:Averaged 21.223-24.189(1960-2234)
Mass Peaks:564 Base Peak:112.70(1245681) Polarity:Neg Segment1 - Event2

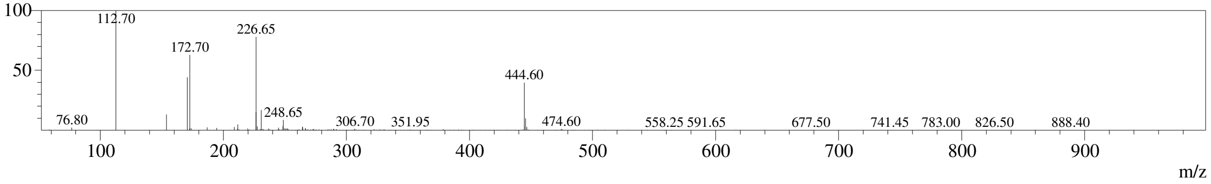

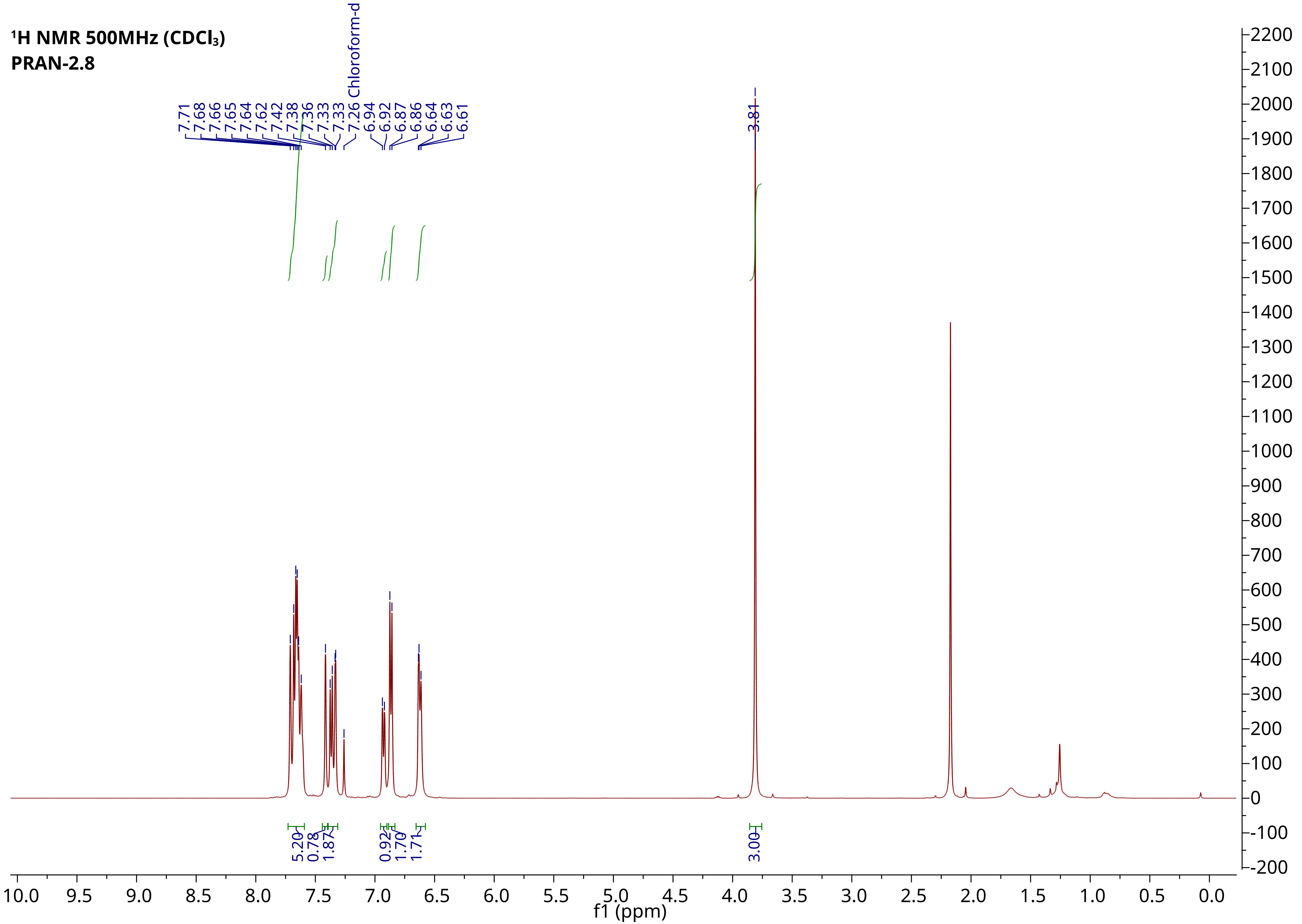

¹H NMR 500MHz (CDCl₃)
PRAN-2.8
7.71
7.68
7.66
7.65
7.64
7.62
7.42
7.38
7.36
7.33
7.33
7.26 Chloroform-d
6.94
6.92
6.87
6.86
6.64
6.63
6.61
3.81
5.20
0.78
1.87
0.92
1.70
1.71
3.00
f1 (ppm)

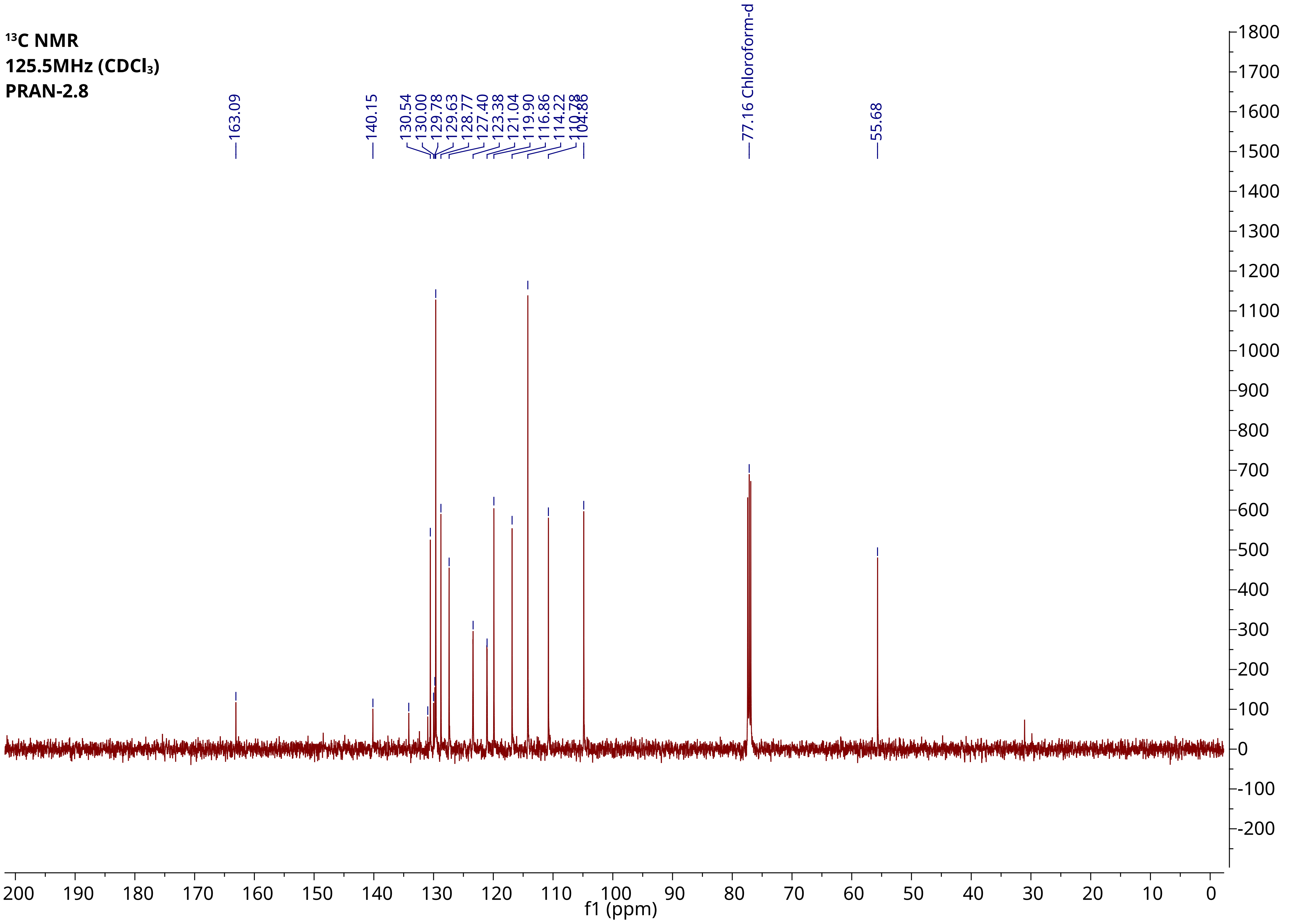

¹³C NMR
125.5MHz (CDCl₃)
PRAN-2.8
163.09
140.15
130.54
130.00
129.78
129.63
128.77
127.40
123.38
121.04
119.90
116.86
114.22
104.86
77.16 Chloroform-d
55.68
f1 (ppm)

# ==== Shimadzu LCMSsolution Analysis Report ====

**Sample Name : PRAN-2.9**

Method

Column: Purospher RP-8
Mobile Phase A: H2O + 0.9% acetic acid
Mobile Phase B: ACN
% Pump B Concetrate: 50.0
Flow (ml/min): 0.6000

Detector A:SPD-20A
UV_1.Wavelength: 216
UV_2.Wavelength: 264
LC Program

| Time | Unit | Command | Value |
|---|---|---|---|
| 0.01 | Pumps | B.Conc | 50 |
| 15.00 | Pumps | B.Conc | 90 |
| 30.00 | Pumps | B.Conc | 90 |
| 30.01 | Pumps | B.Conc | 50 |
| 40.00 | Controller | Stop | |

MS Chromatogram

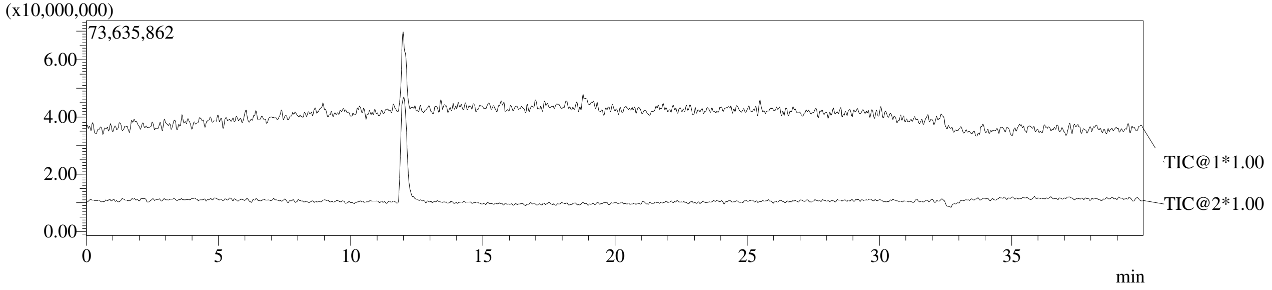


## <LC-UV Chromatogram>

Chromatogram

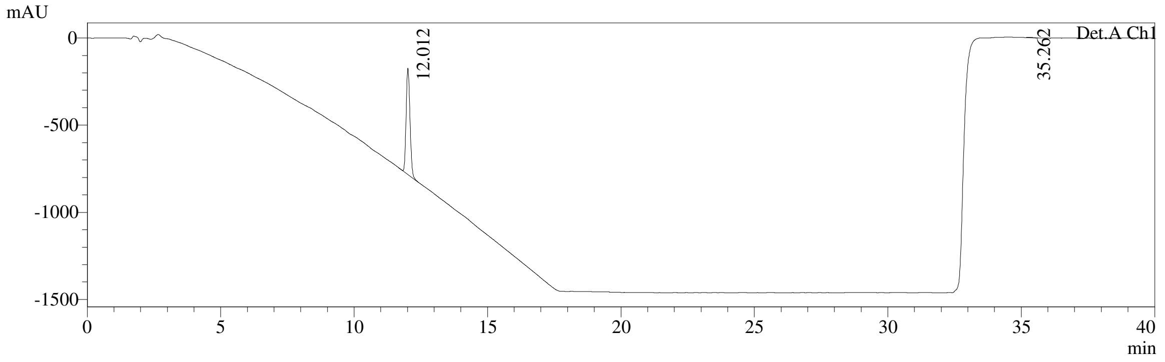

## Sample Name : PRAN-2.9

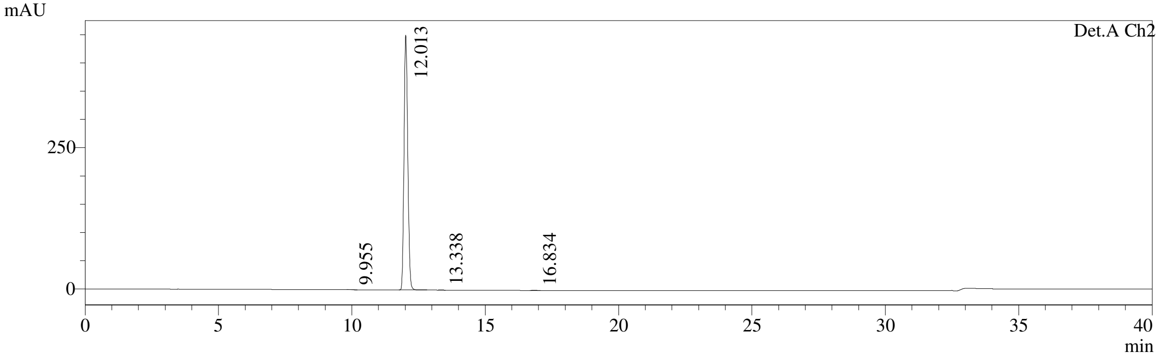


1 Det.A Ch1 / 216nm
2 Det.A Ch2 / 264nm

PeakTable

Detector A Ch2 264nm

| Peak# | Ret. Time | Area | Height | Area % | Height % |
|---|---|---|---|---|---|
| 1 | 9.955 | 1169 | 106 | 0.026 | 0.023 |
| 2 | 12.013 | 4410938 | 450894 | 99.813 | 99.779 |
| 3 | 13.338 | 2691 | 358 | 0.061 | 0.079 |
| 4 | 16.834 | 4383 | 535 | 0.099 | 0.118 |
| Total | | 4419181 | 451894 | 100.000 | 100.000 |

MS Spectrum Graph

#:1 Ret.Time:Averaged 11.505-12.523(Scan#:1063-1157)
BG Mode:Averaged 19.478-39.028(1799-3603)
Mass Peaks:473 Base Peak:82.85(8966218) Polarity:Pos Segment1 - Event1

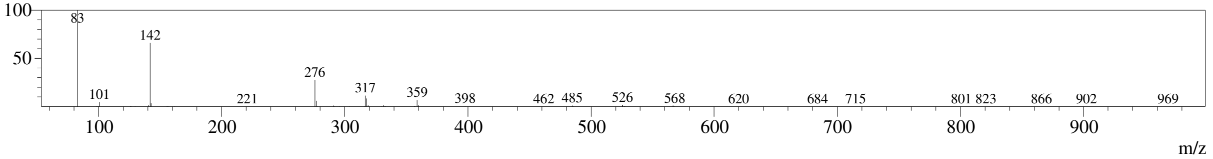


#:2 Ret.Time:Averaged 11.516-12.534(Scan#:1064-1158)
BG Mode:Averaged 19.489-39.028(1800-3604)
Mass Peaks:522 Base Peak:482.60(3906372) Polarity:Neg Segment1 - Event2

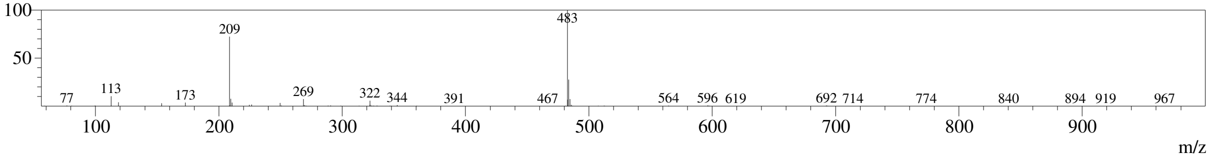

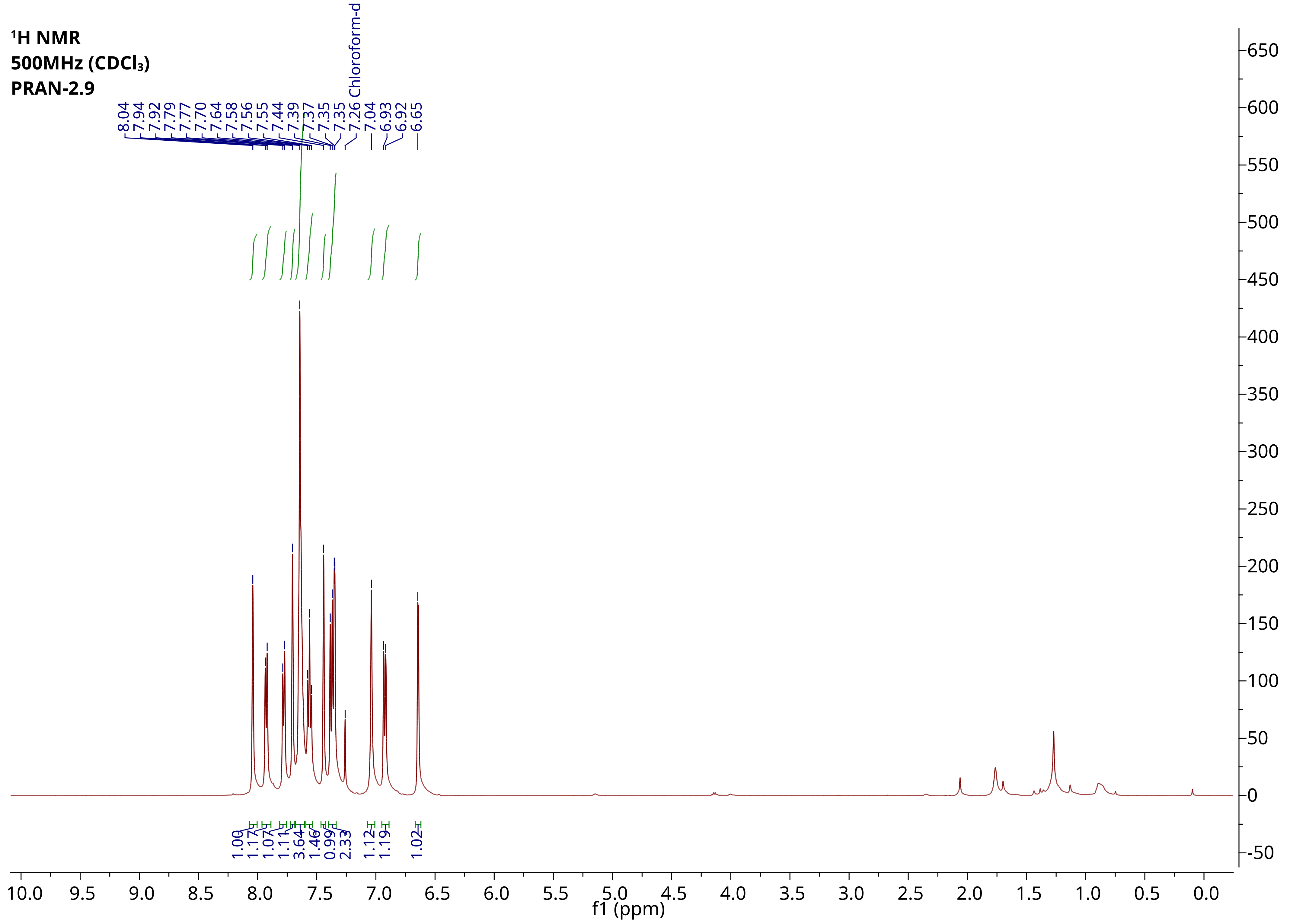

¹H NMR
500MHz (CDCl₃)
PRAN-2.9
8.04
7.94
7.92
7.79
7.77
7.70
7.64
7.58
7.56
7.55
7.44
7.39
7.37
7.35
7.35
7.26 Chloroform-d
7.04
6.93
6.92
6.65
1.00
1.17
1.07
1.11
3.64
1.46
0.99
2.33
1.12
1.19
1.02
f1 (ppm)

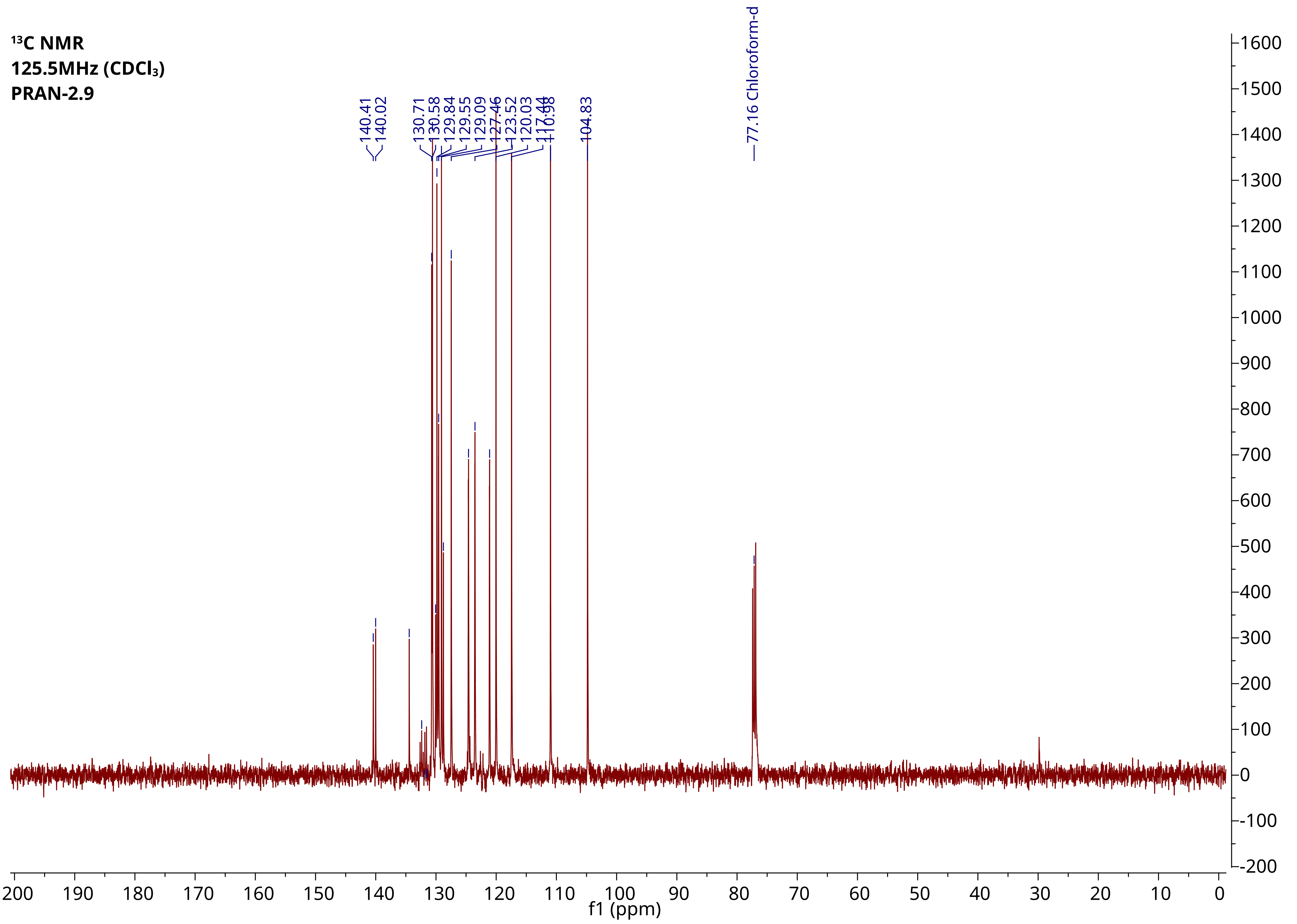

13C NMR
125.5MHz (CDCl3)
PRAN-2.9
140.41
140.02
130.71
130.58
129.84
129.55
129.09
127.46
123.52
120.03
117.44
110.98
104.83
77.16 Chloroform-d
f1 (ppm)

# ==== Shimadzu LCMSsolution Analysis Report ====

**Sample Name : PRAN-2.10**

Method

Column: Purospher RP-8
Mobile Phase A: H2O + 0.9% acetic acid
Mobile Phase B: ACN
% Pump B Concetrate: 50.0
Flow (ml/min): 0.6000

Detector A:SPD-20A
UV_1.Wavelength: 216
UV_2.Wavelength: 264
LC Program

| Time | Unit | Command | Value |
|---|---|---|---|
| 0.01 | Pumps | B.Conc | 50 |
| 15.00 | Pumps | B.Conc | 90 |
| 30.00 | Pumps | B.Conc | 90 |
| 30.01 | Pumps | B.Conc | 50 |
| 40.00 | Controller | Stop | |

MS Chromatogram

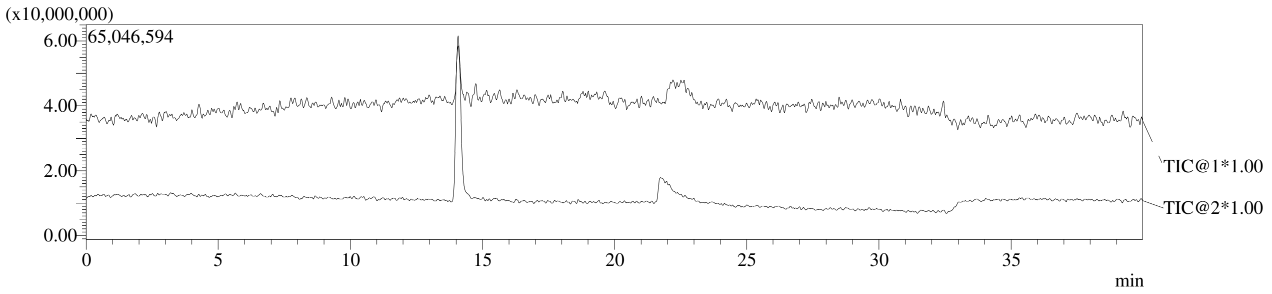


## <LC-UV Chromatogram>

Chromatogram

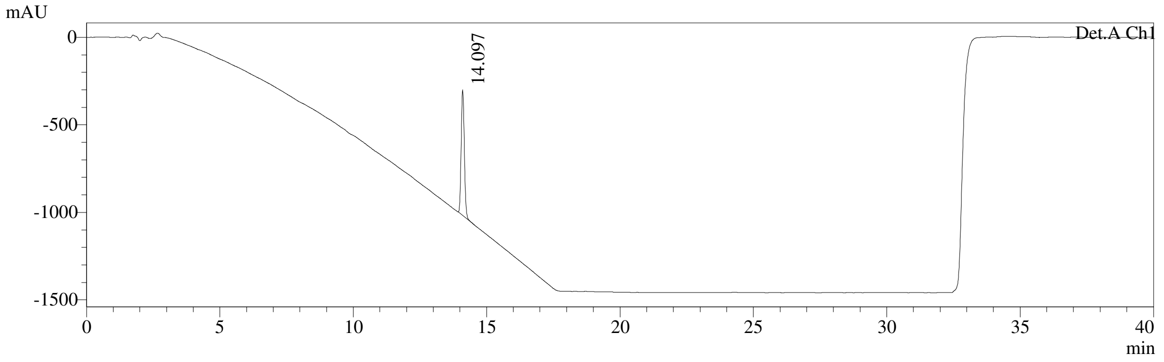

## Sample Name : PRAN-2.10

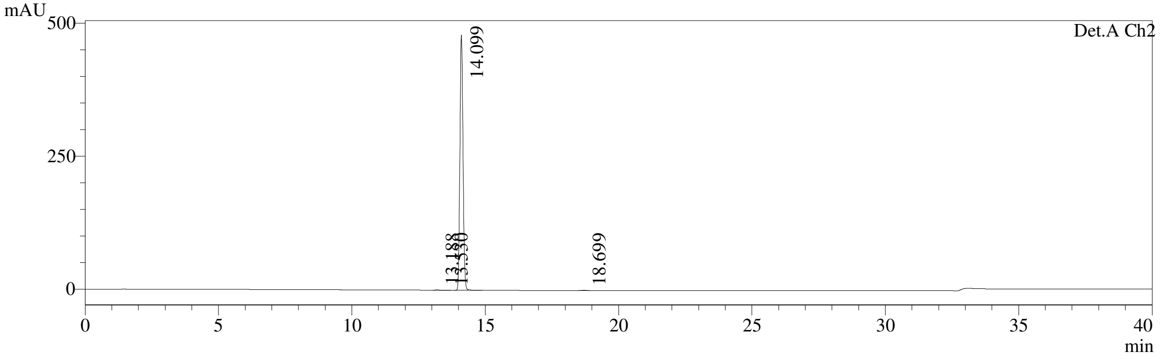


1 Det.A Ch1 / 216nm
2 Det.A Ch2 / 264nm

PeakTable

Detector A Ch2 264nm

| Peak# | Ret. Time | Area | Height | Area % | Height % |
|---|---|---|---|---|---|
| 1 | 13.188 | 2364 | 286 | 0.061 | 0.059 |
| 2 | 13.530 | 1123 | 122 | 0.029 | 0.025 |
| 3 | 14.099 | 3881852 | 479568 | 99.753 | 99.786 |
| 4 | 18.699 | 6109 | 619 | 0.157 | 0.129 |
| Total | | 3891449 | 480594 | 100.000 | 100.000 |

MS Spectrum Graph

#:1 Ret.Time:Averaged 13.585-14.668(Scan#:1255-1355)
BG Mode:Averaged 27.863-39.299(2573-3629)
Mass Peaks:595 Base Peak:82.85(8949042) Polarity:Pos Segment1 - Event1

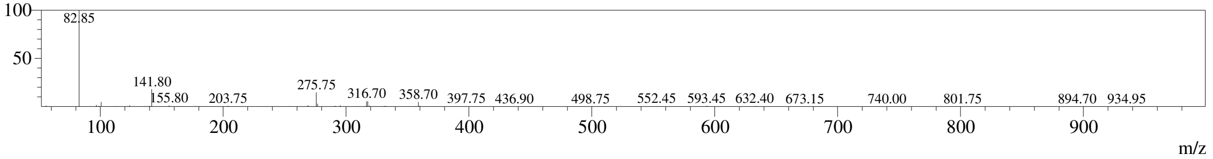


#:2 Ret.Time:Averaged 13.596-14.679(Scan#:1256-1356)
BG Mode:Averaged 27.874-39.299(2574-3630)
Mass Peaks:766 Base Peak:550.50(4336294) Polarity:Neg Segment1 - Event2

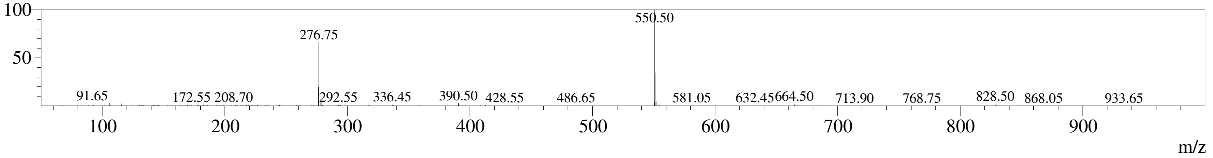

**$^1$H NMR 500MHz ($CDCl_3$)**
**PRAN-2.10**

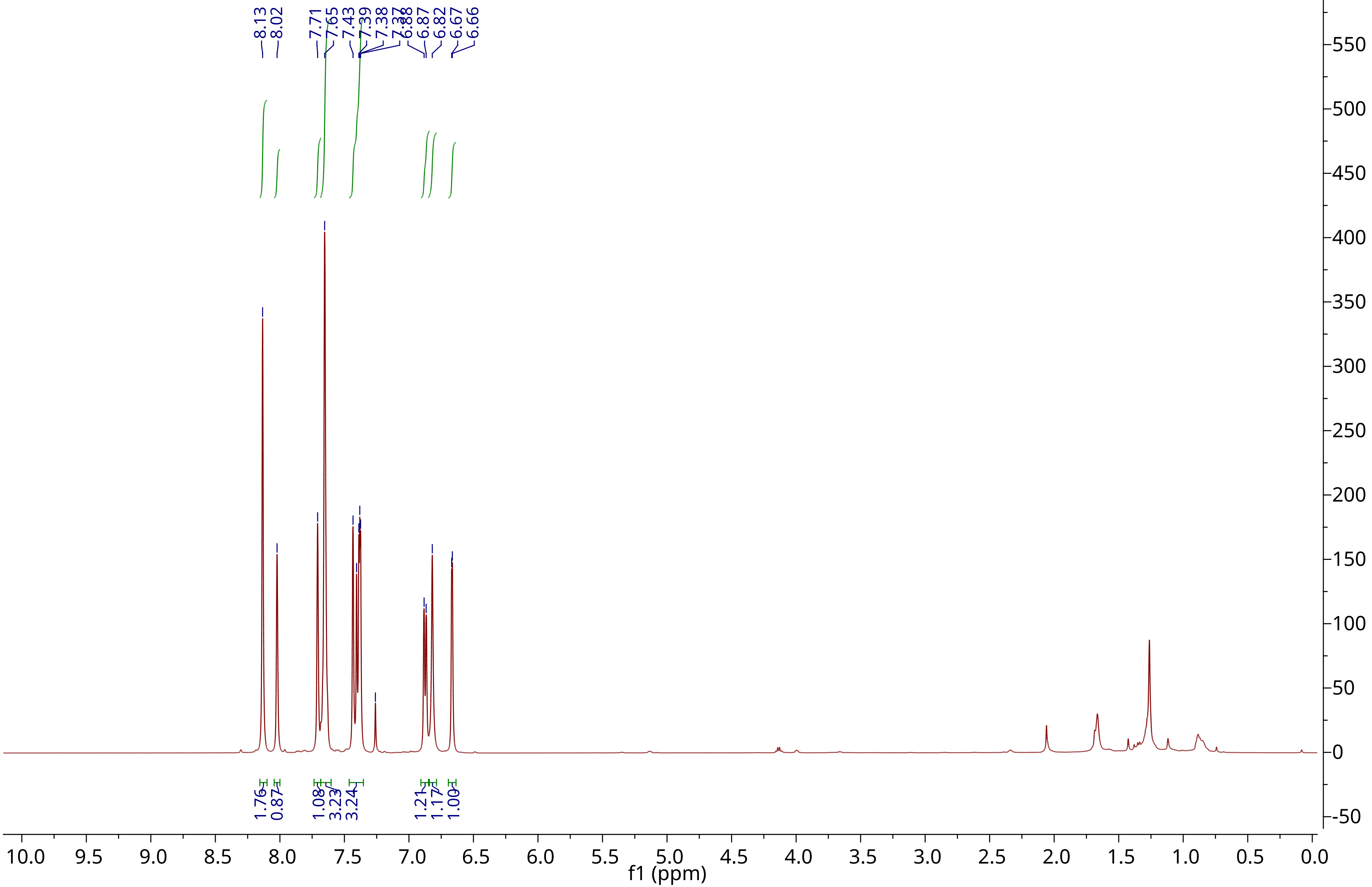

$^{13}$C NMR
125.5MHz ($CDCl_3$)
PRAN-2.10

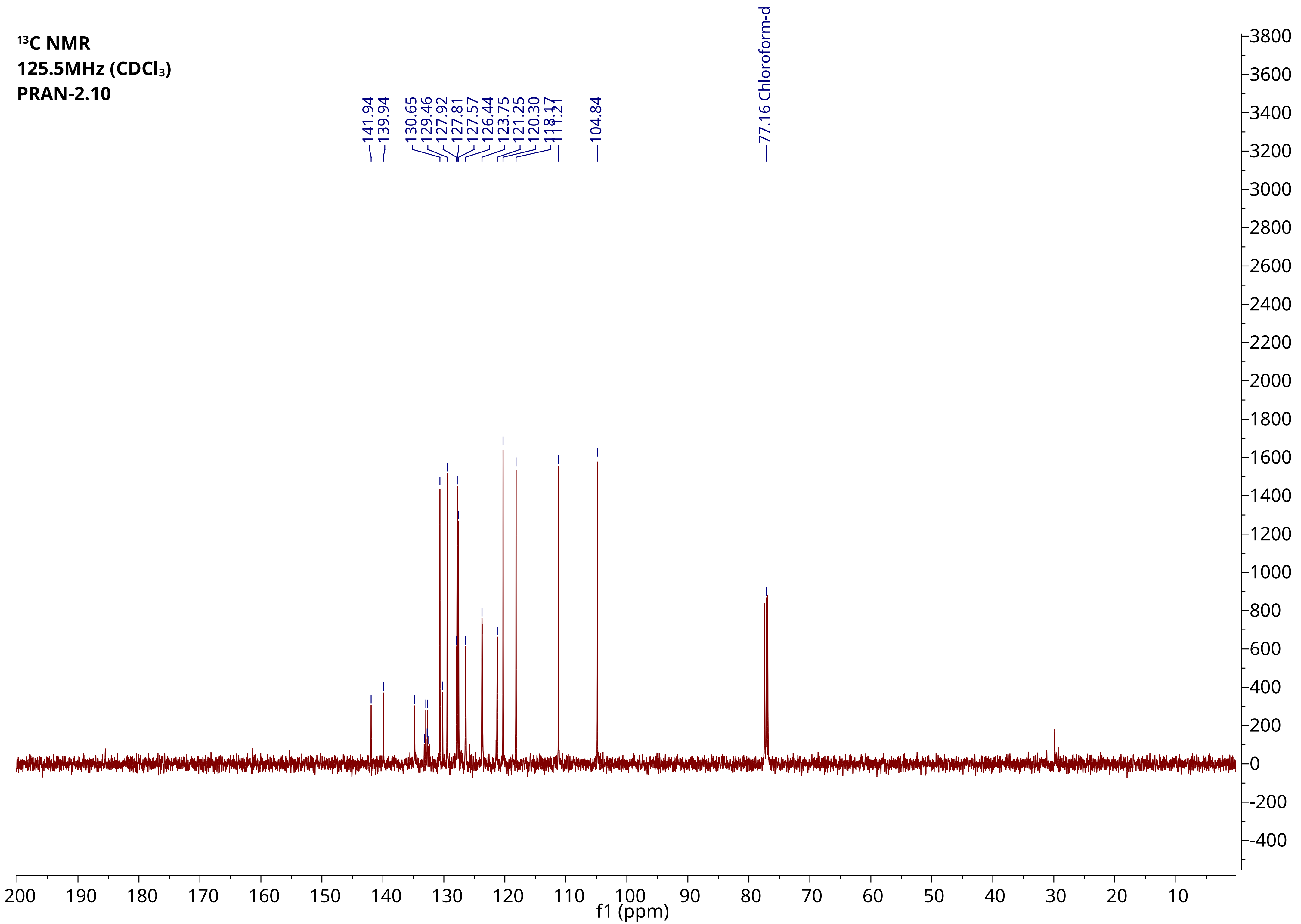

# ==== Shimadzu LCMSsolution Analysis Report ====

**Sample Name : PRAN-3.1**

Method

Column: Purospher RP-8
Mobile Phase A: H2O + 0.9% acetic acid
Mobile Phase B: ACN
% Pump B Concetrate: 50.0
Flow (ml/min): 0.6000

Detector A:SPD-20A
UV_1.Wavelength: 216
UV_2.Wavelength: 264
LC Program

| Time | Unit | Command | Value |
|---|---|---|---|
| 0.01 | Pumps | B.Conc | 50 |
| 15.00 | Pumps | B.Conc | 90 |
| 30.00 | Pumps | B.Conc | 90 |
| 30.01 | Pumps | B.Conc | 50 |
| 40.00 | Controller | Stop | |

MS Chromatogram

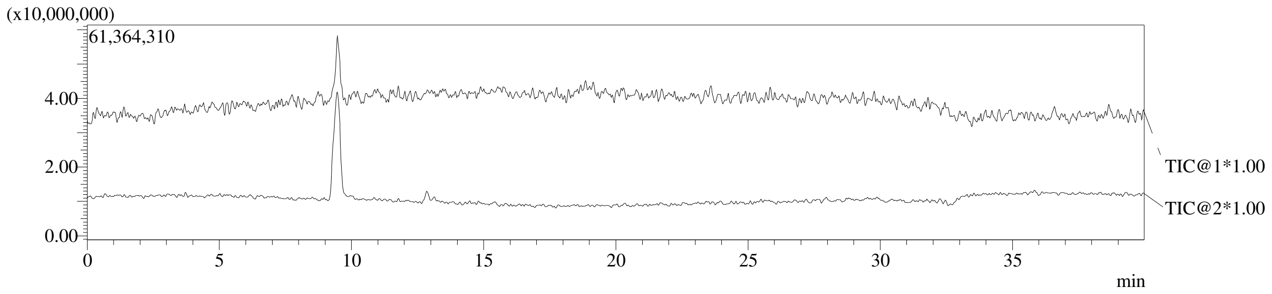


## <LC-UV Chromatogram>

Chromatogram

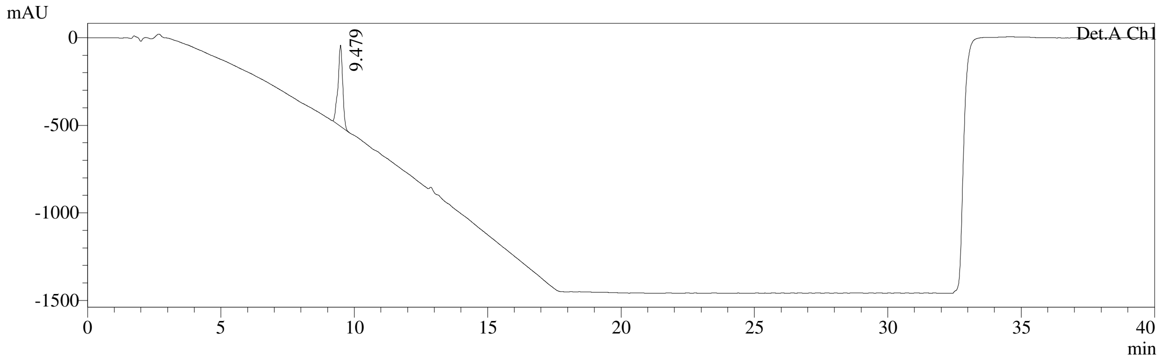

## Sample Name : PRAN-3.1

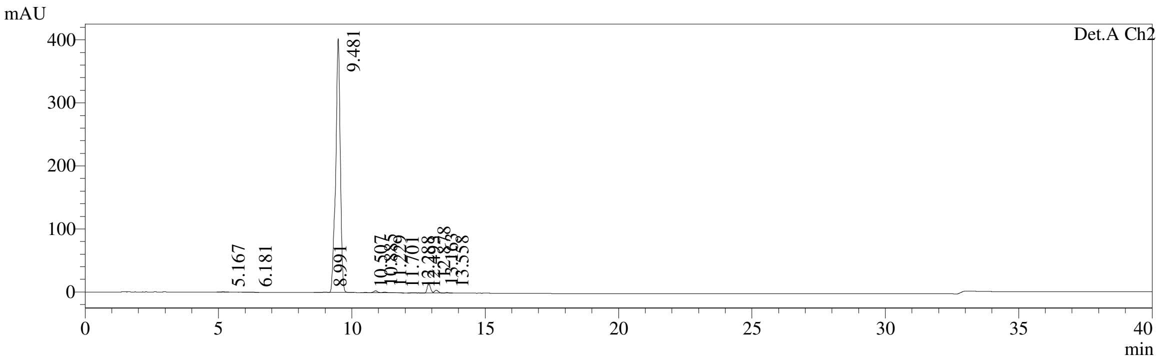


1 Det.A Ch1 / 216nm
2 Det.A Ch2 / 264nm

PeakTable

Detector A Ch2 264nm

| Peak# | Ret. Time | Area | Height | Area % | Height % |
|---|---|---|---|---|---|
| 1 | 5.167 | 5579 | 531 | 0.109 | 0.123 |
| 2 | 6.181 | 4622 | 317 | 0.090 | 0.074 |
| 3 | 8.991 | 10130 | 771 | 0.198 | 0.179 |
| 4 | 9.481 | 4858245 | 403183 | 94.953 | 93.512 |
| 5 | 10.507 | 3099 | 319 | 0.061 | 0.074 |
| 6 | 10.885 | 32251 | 2974 | 0.630 | 0.690 |
| 7 | 11.229 | 12078 | 1045 | 0.236 | 0.242 |
| 8 | 11.701 | 2986 | 305 | 0.058 | 0.071 |
| 9 | 12.288 | 2934 | 240 | 0.057 | 0.056 |
| 10 | 12.493 | 1343 | 141 | 0.026 | 0.033 |
| 11 | 12.878 | 130938 | 15578 | 2.559 | 3.613 |
| 12 | 13.163 | 44382 | 4649 | 0.867 | 1.078 |
| 13 | 13.558 | 7900 | 1103 | 0.154 | 0.256 |
| Total | | 5116485 | 431156 | 100.000 | 100.000 |

MS Spectrum Graph

#:1 Ret.Time:Averaged 9.447-9.490(Scan#:873-877)
BG Mode:Calc 9.317<->9.707(861<->897)
Mass Peaks:589 Base Peak:243.75(6233496) Polarity:Pos Segment1 - Event1

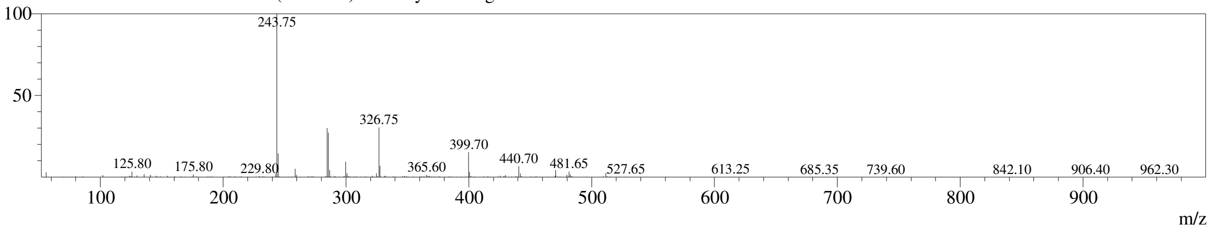

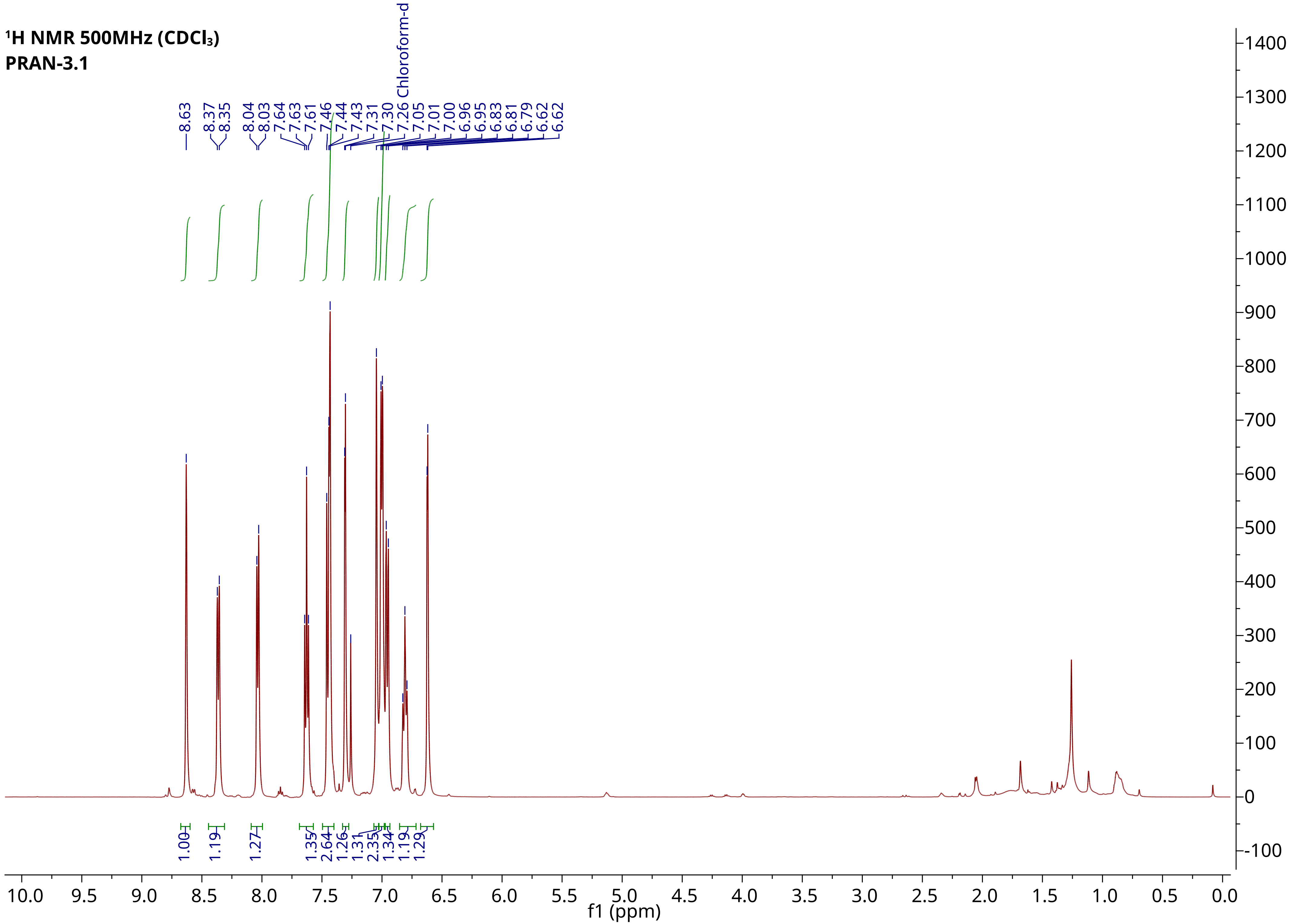

¹H NMR 500MHz (CDCl₃)
PRAN-3.1
8.63
8.37
8.35
8.04
8.03
7.64
7.63
7.61
7.46
7.44
7.43
7.31
7.30
7.26 Chloroform-d
7.05
7.01
7.00
6.96
6.95
6.83
6.81
6.79
6.62
6.62
1.00
1.19
1.27
1.35
2.64
1.26
1.31
2.35
1.34
1.19
1.29
f1 (ppm)

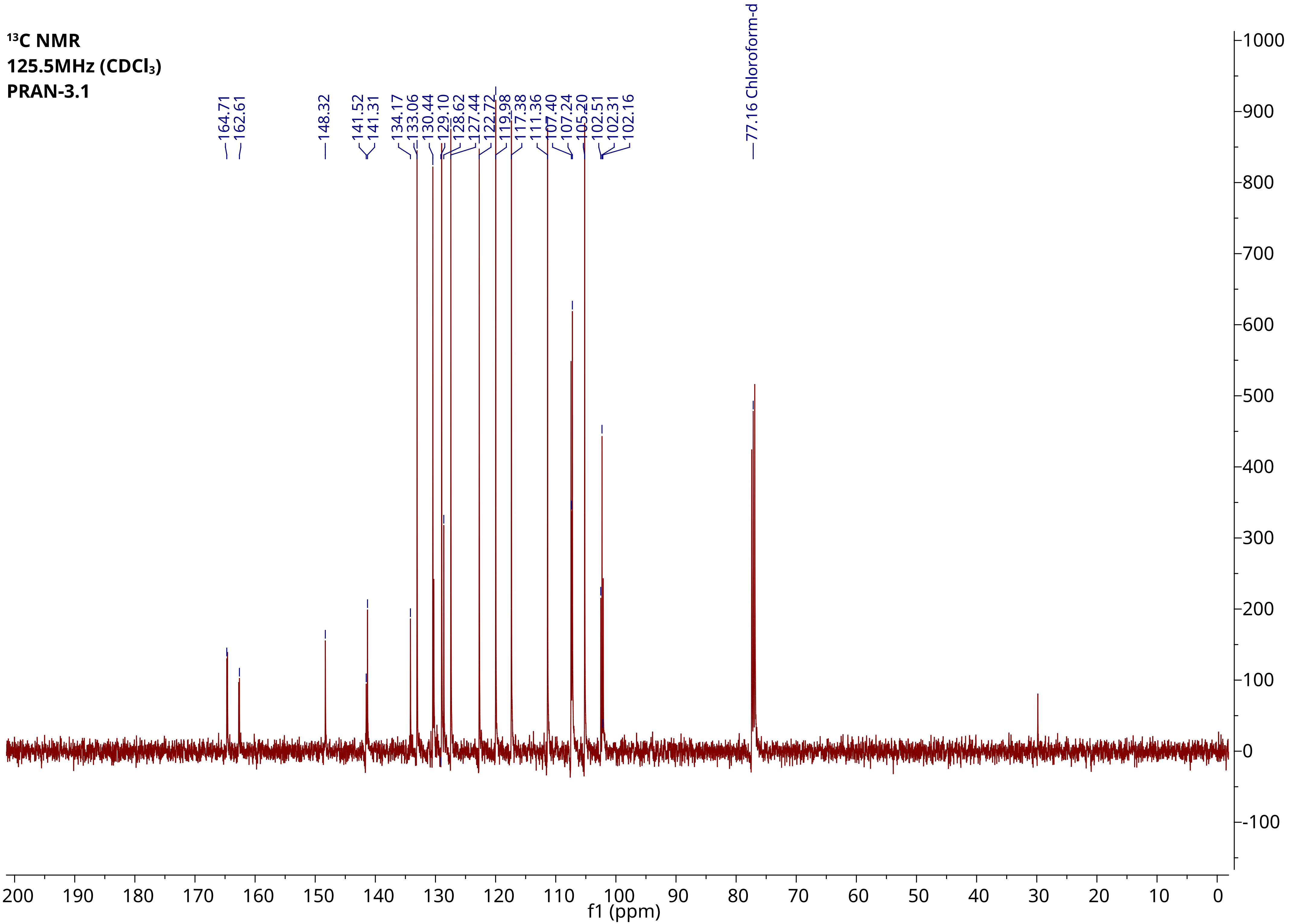
13C NMR
125.5MHz (CDCl3)
PRAN-3.1
164.71
162.61
148.32
141.52
141.31
134.17
133.06
130.44
129.10
128.62
127.44
122.72
119.98
117.38
111.36
107.40
107.24
105.20
102.51
102.31
102.16
77.16 Chloroform-d
f1 (ppm)

# ==== Shimadzu LCMSsolution Analysis Report ====

**Sample Name : PRAN-3.2**

Method

Column: Purospher RP-8
Mobile Phase A: H2O + 0.9% acetic acid
Mobile Phase B: ACN
% Pump B Concetrate: 50.0
Flow (ml/min): 0.6000

Detector A:SPD-20A
UV_1.Wavelength: 216
UV_2.Wavelength: 264
LC Program

| Time | Unit | Command | Value |
|---|---|---|---|
| 0.01 | Pumps | B.Conc | 50 |
| 15.00 | Pumps | B.Conc | 90 |
| 30.00 | Pumps | B.Conc | 90 |
| 30.01 | Pumps | B.Conc | 50 |
| 40.00 | Controller | Stop | |

MS Chromatogram

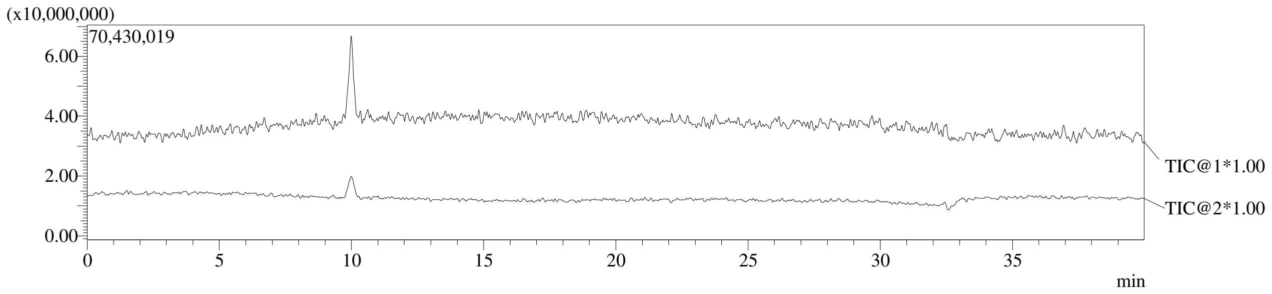


## <LC-UV Chromatogram>

Chromatogram

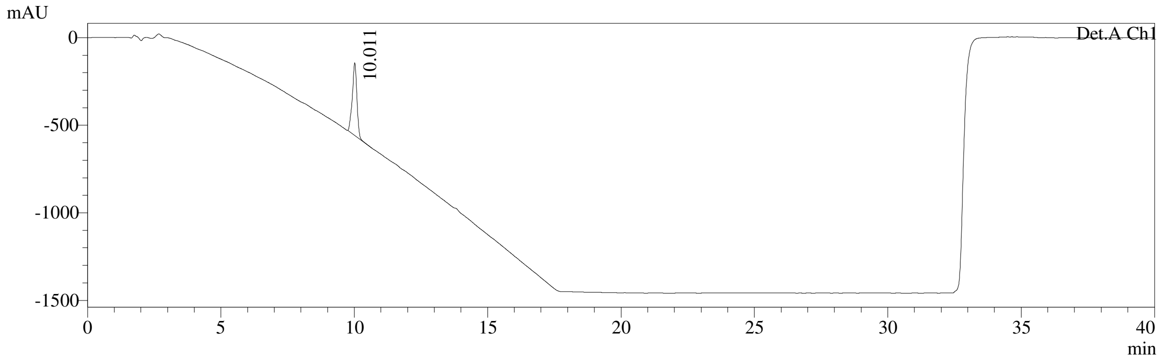

## Sample Name : PRAN-3.2

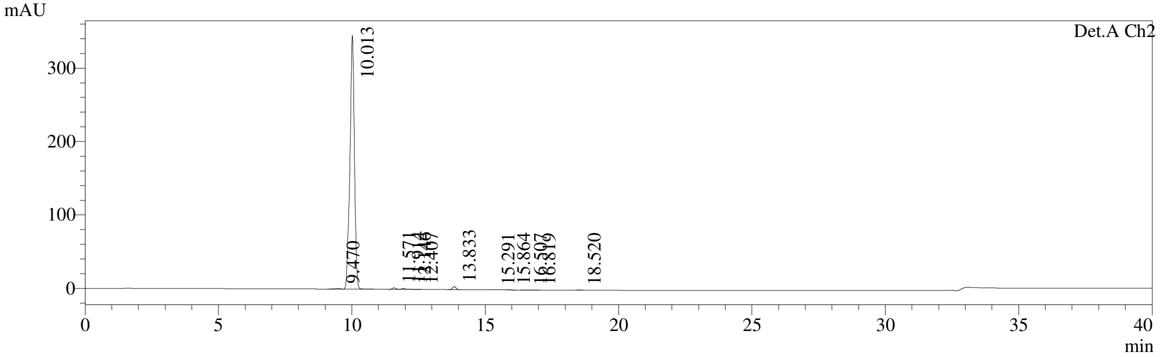


1 Det.A Ch1 / 216nm
2 Det.A Ch2 / 264nm

PeakTable

Detector A Ch2 264nm

| Peak# | Ret. Time | Area | Height | Area % | Height % |
|---|---|---|---|---|---|
| 1 | 9.470 | 11642 | 900 | 0.279 | 0.253 |
| 2 | 10.013 | 4078112 | 345737 | 97.654 | 97.211 |
| 3 | 11.571 | 19754 | 1972 | 0.473 | 0.555 |
| 4 | 11.914 | 10889 | 1061 | 0.261 | 0.298 |
| 5 | 12.146 | 4173 | 482 | 0.100 | 0.136 |
| 6 | 12.407 | 1808 | 240 | 0.043 | 0.068 |
| 7 | 13.833 | 39191 | 4321 | 0.938 | 1.215 |
| 8 | 15.291 | 1443 | 124 | 0.035 | 0.035 |
| 9 | 15.864 | 5007 | 474 | 0.120 | 0.133 |
| 10 | 16.507 | 1884 | 108 | 0.045 | 0.030 |
| 11 | 16.819 | 1174 | 129 | 0.028 | 0.036 |
| 12 | 18.520 | 1018 | 108 | 0.024 | 0.030 |
| Total | | 4176094 | 355656 | 100.000 | 100.000 |

MS Spectrum Graph

#:1 Ret.Time:Averaged 9.685-10.270(Scan#:895-949)
BG Mode:Averaged 17.658-31.063(1631-2869)
Mass Peaks:503 Base Peak:82.85(4659621) Polarity:Pos Segment1 - Event1

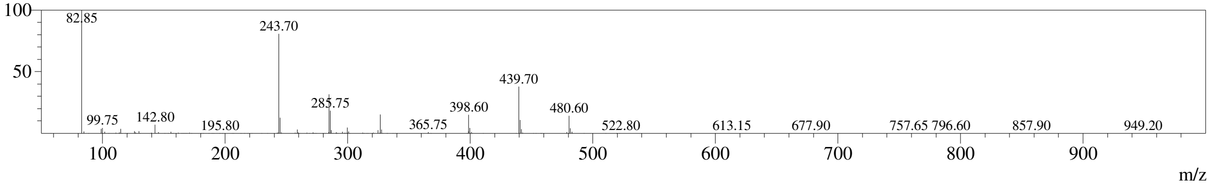


#:2 Ret.Time:Averaged 9.696-10.281(Scan#:896-950)
BG Mode:Averaged 17.669-31.063(1632-2870)
Mass Peaks:481 Base Peak:226.65(1736415) Polarity:Neg Segment1 - Event2

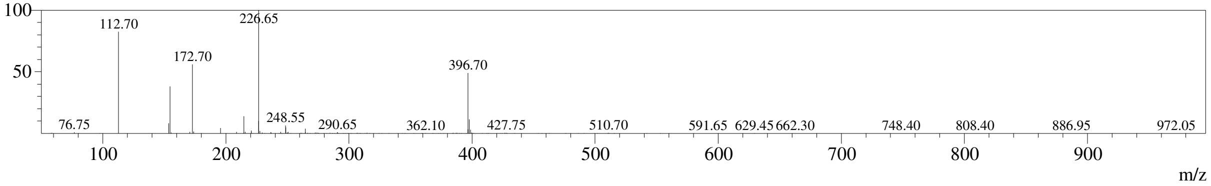

$^{1}H$ NMR 500MHz ($CDCl_3$)
PRAN-3.2

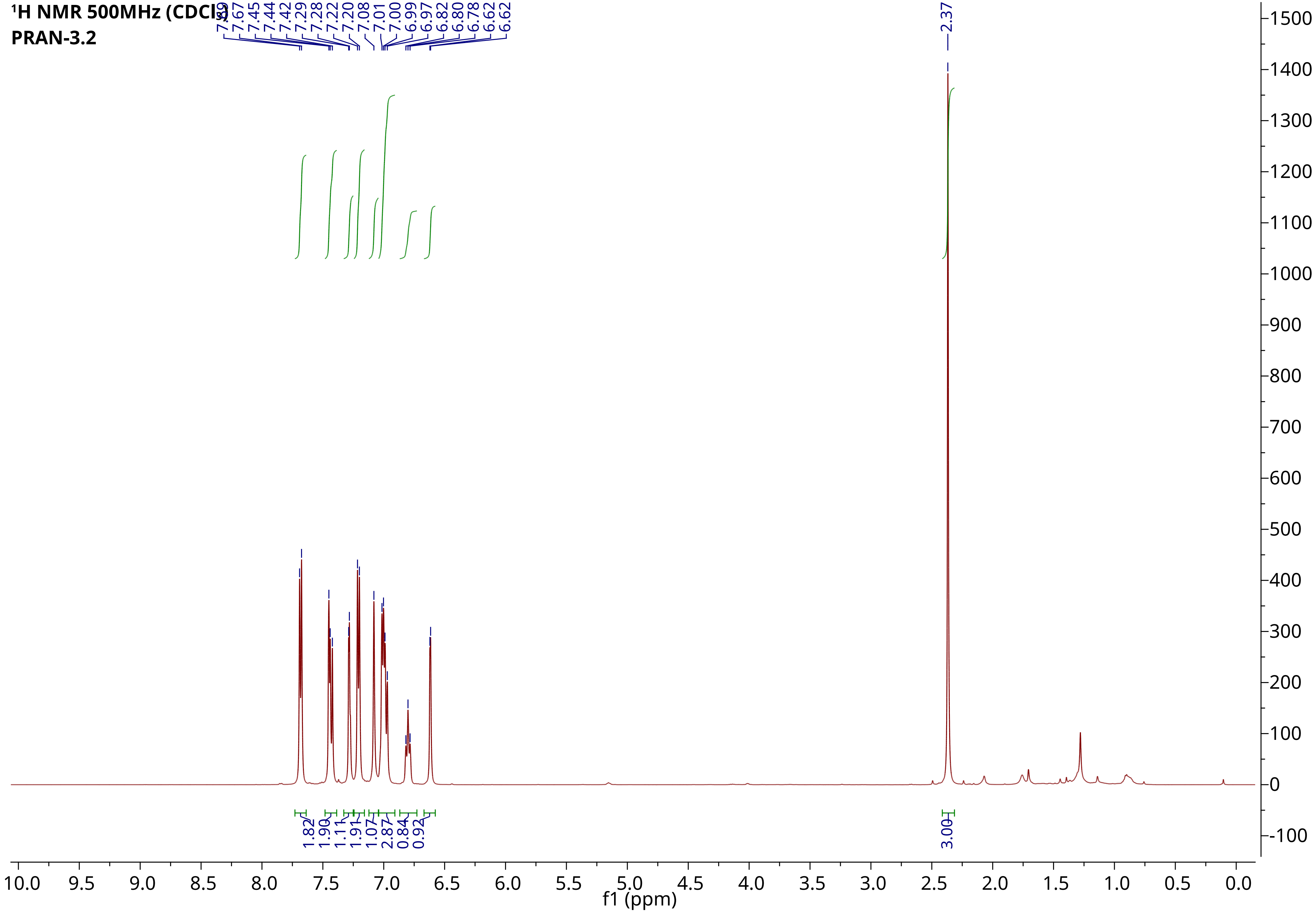

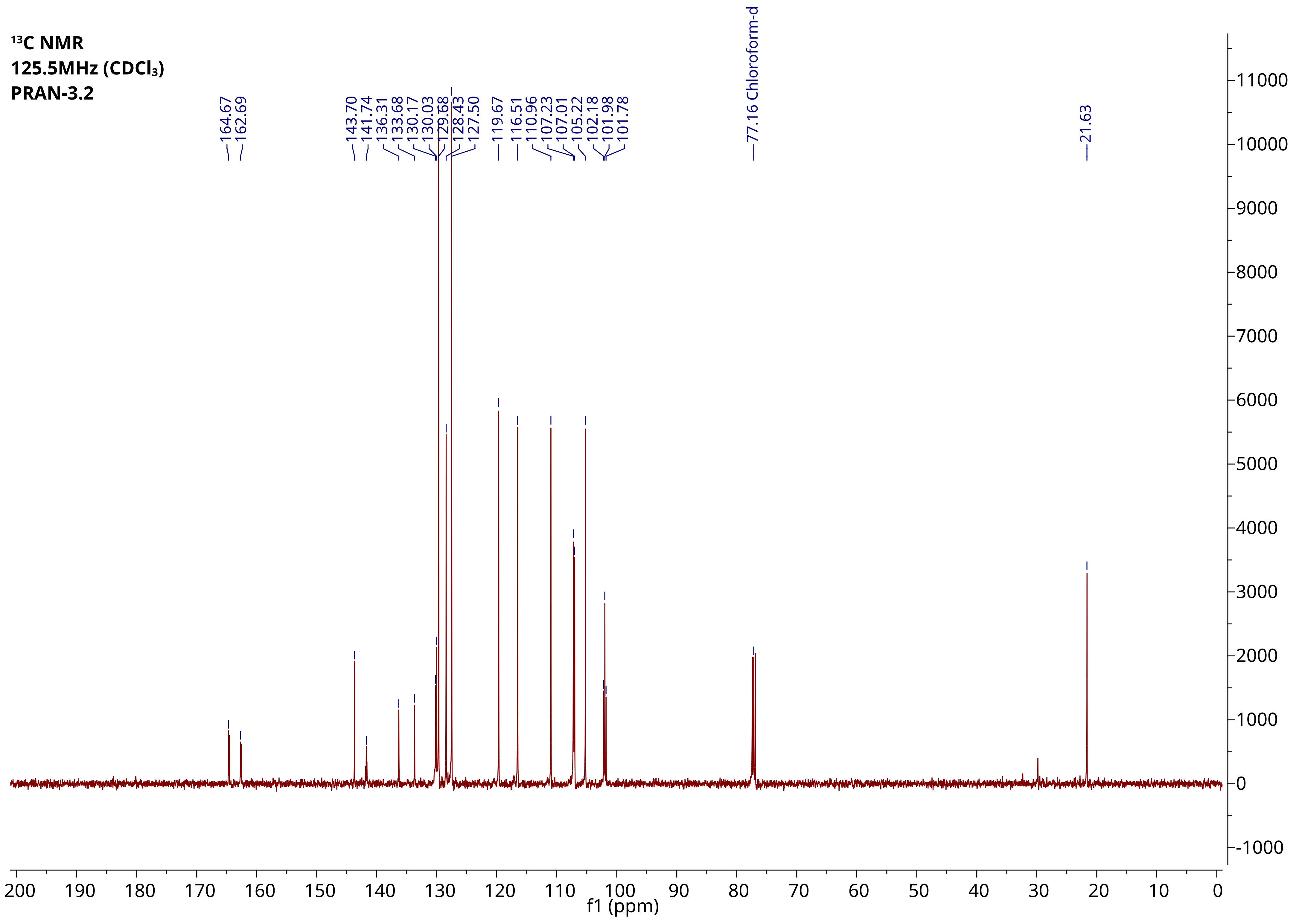

13C NMR
125.5MHz (CDCl3)
PRAN-3.2
164.67
162.69
143.70
141.74
136.31
133.68
130.17
130.03
129.68
128.43
127.50
119.67
116.51
110.96
107.23
107.01
105.22
102.18
101.98
101.78
77.16 Chloroform-d
21.63
f1 (ppm)

# ==== Shimadzu LCMSsolution Analysis Report ====

**Sample Name : PRAN-3.3**

Method

Column: Purospher RP-8
Mobile Phase A: H2O + 0.9% acetic acid
Mobile Phase B: ACN
% Pump B Concetrate: 50.0
Flow (ml/min): 0.6000

Detector A:SPD-20A
UV_1.Wavelength: 216
UV_2.Wavelength: 264
LC Program

| Time | Unit | Command | Value |
|---|---|---|---|
| 0.01 | Pumps | B.Conc | 50 |
| 15.00 | Pumps | B.Conc | 90 |
| 30.00 | Pumps | B.Conc | 90 |
| 30.01 | Pumps | B.Conc | 50 |
| 40.00 | Controller | Stop | |

MS Chromatogram

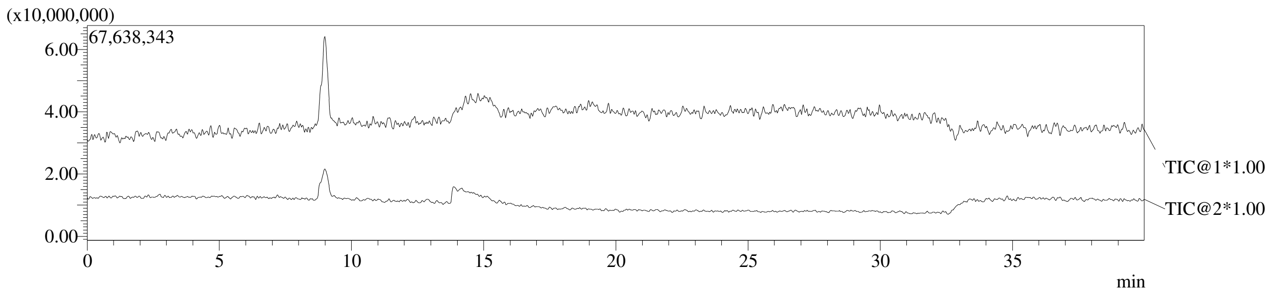


**<LC-UV Chromatogram>**

Chromatogram

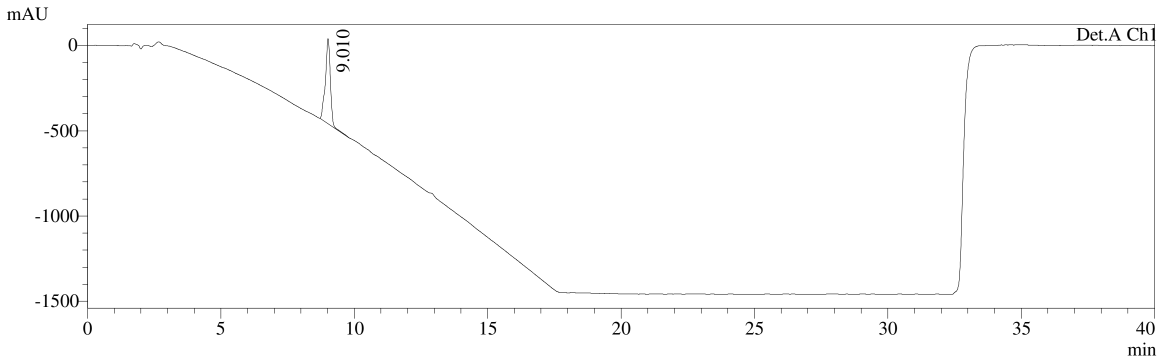

## Sample Name : PRAN-3.3

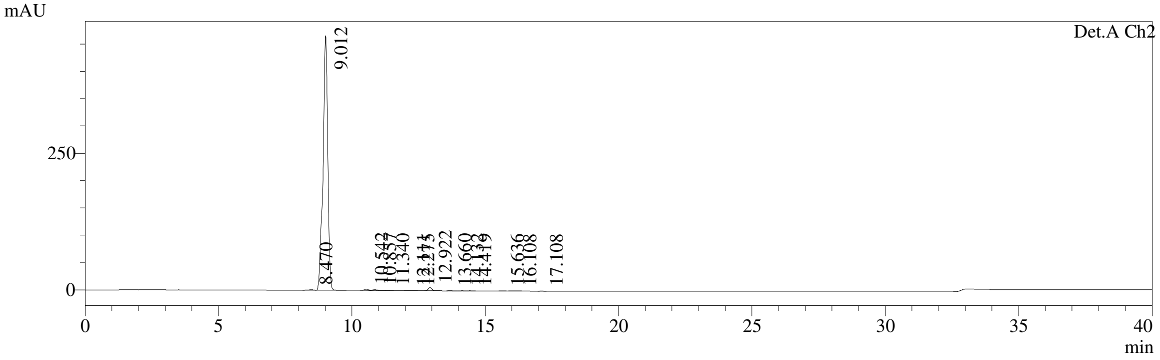


1 Det.A Ch1 / 216nm
2 Det.A Ch2 / 264nm

PeakTable

Detector A Ch2 264nm

| Peak# | Ret. Time | Area | Height | Area % | Height % |
|---|---|---|---|---|---|
| 1 | 8.470 | 16211 | 1160 | 0.275 | 0.242 |
| 2 | 9.012 | 5755487 | 466080 | 97.542 | 97.323 |
| 3 | 10.542 | 27203 | 2349 | 0.461 | 0.491 |
| 4 | 10.857 | 27642 | 1512 | 0.468 | 0.316 |
| 5 | 11.340 | 3859 | 422 | 0.065 | 0.088 |
| 6 | 12.111 | 2035 | 172 | 0.034 | 0.036 |
| 7 | 12.275 | 1144 | 147 | 0.019 | 0.031 |
| 8 | 12.922 | 55788 | 5881 | 0.945 | 1.228 |
| 9 | 13.660 | 1697 | 158 | 0.029 | 0.033 |
| 10 | 14.132 | 1686 | 169 | 0.029 | 0.035 |
| 11 | 14.419 | 1954 | 198 | 0.033 | 0.041 |
| 12 | 15.636 | 2774 | 360 | 0.047 | 0.075 |
| 13 | 16.108 | 1993 | 166 | 0.034 | 0.035 |
| 14 | 17.108 | 1029 | 126 | 0.017 | 0.026 |
| Total | | 5900501 | 478901 | 100.000 | 100.000 |

MS Spectrum Graph

#:1 Ret.Time:Averaged 8.667-9.252(Scan#:801-855)
BG Mode:Averaged 19.522-27.159(1803-2509)
Mass Peaks:378 Base Peak:82.90(9550761) Polarity:Pos Segment1 - Event1

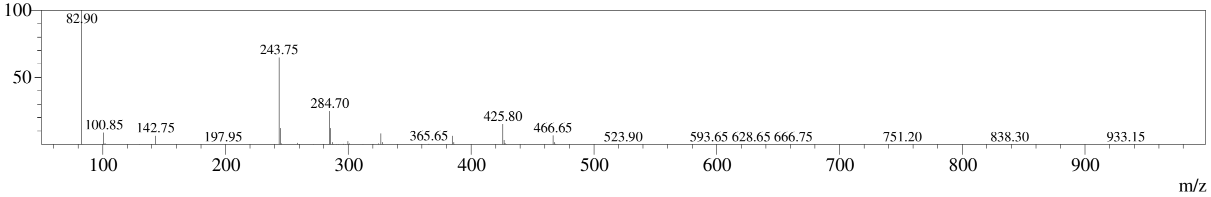


#:2 Ret.Time:Averaged 8.678-9.263(Scan#:802-856)
BG Mode:Averaged 19.533-27.159(1804-2510)
Mass Peaks:519 Base Peak:226.65(1836907) Polarity:Neg Segment1 - Event2

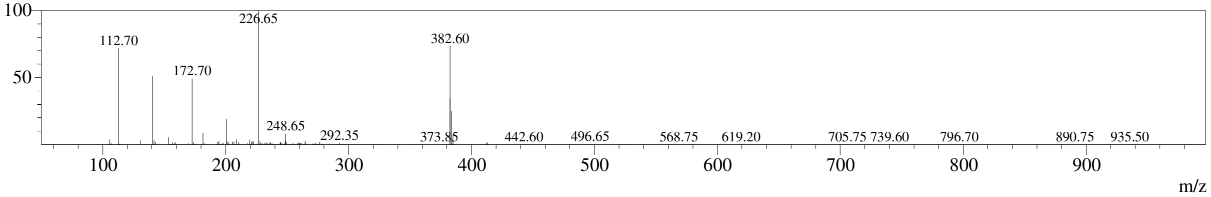

**¹H NMR 500MHz ($CDCl_3$)**
**PRAN-3.3**

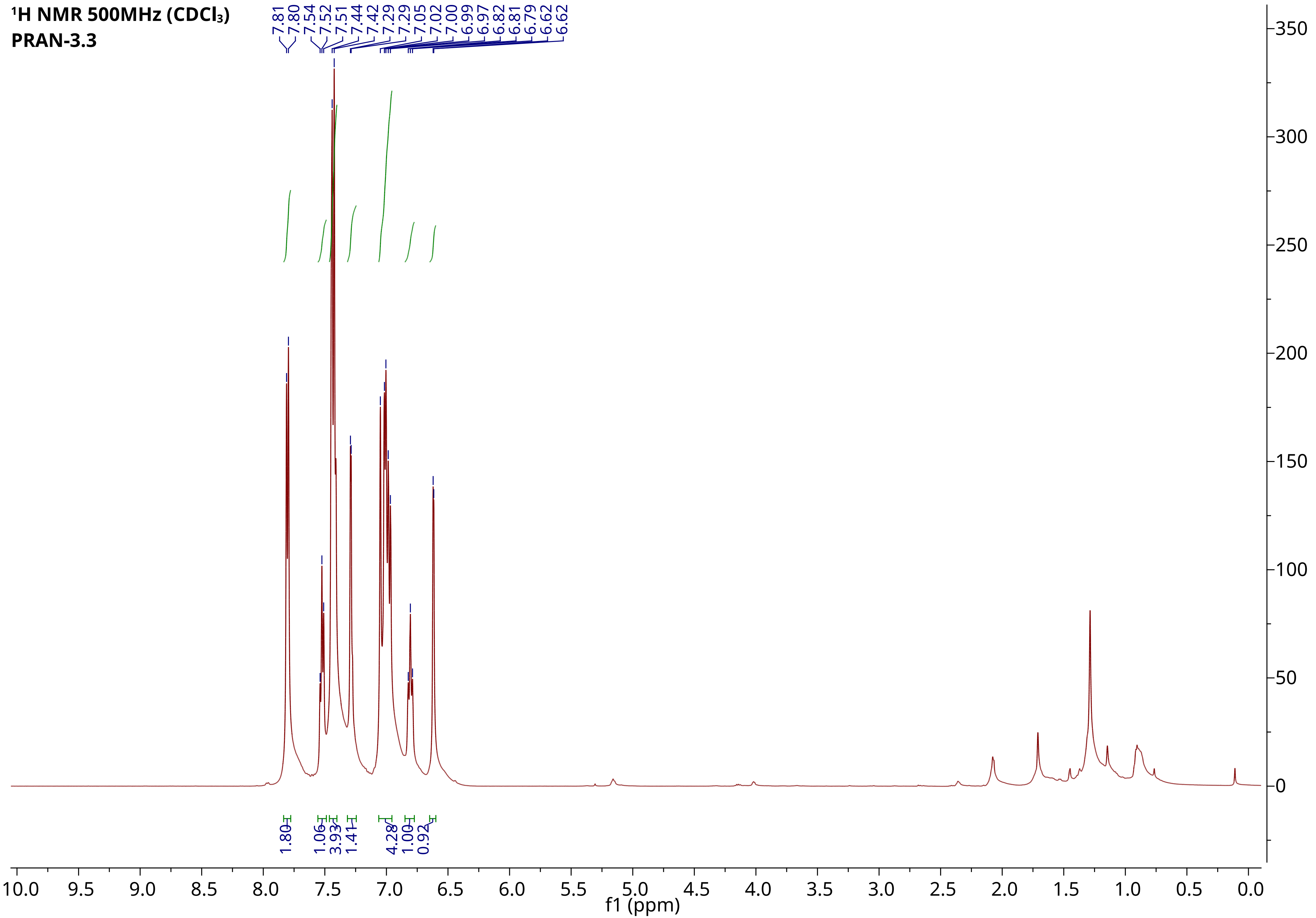

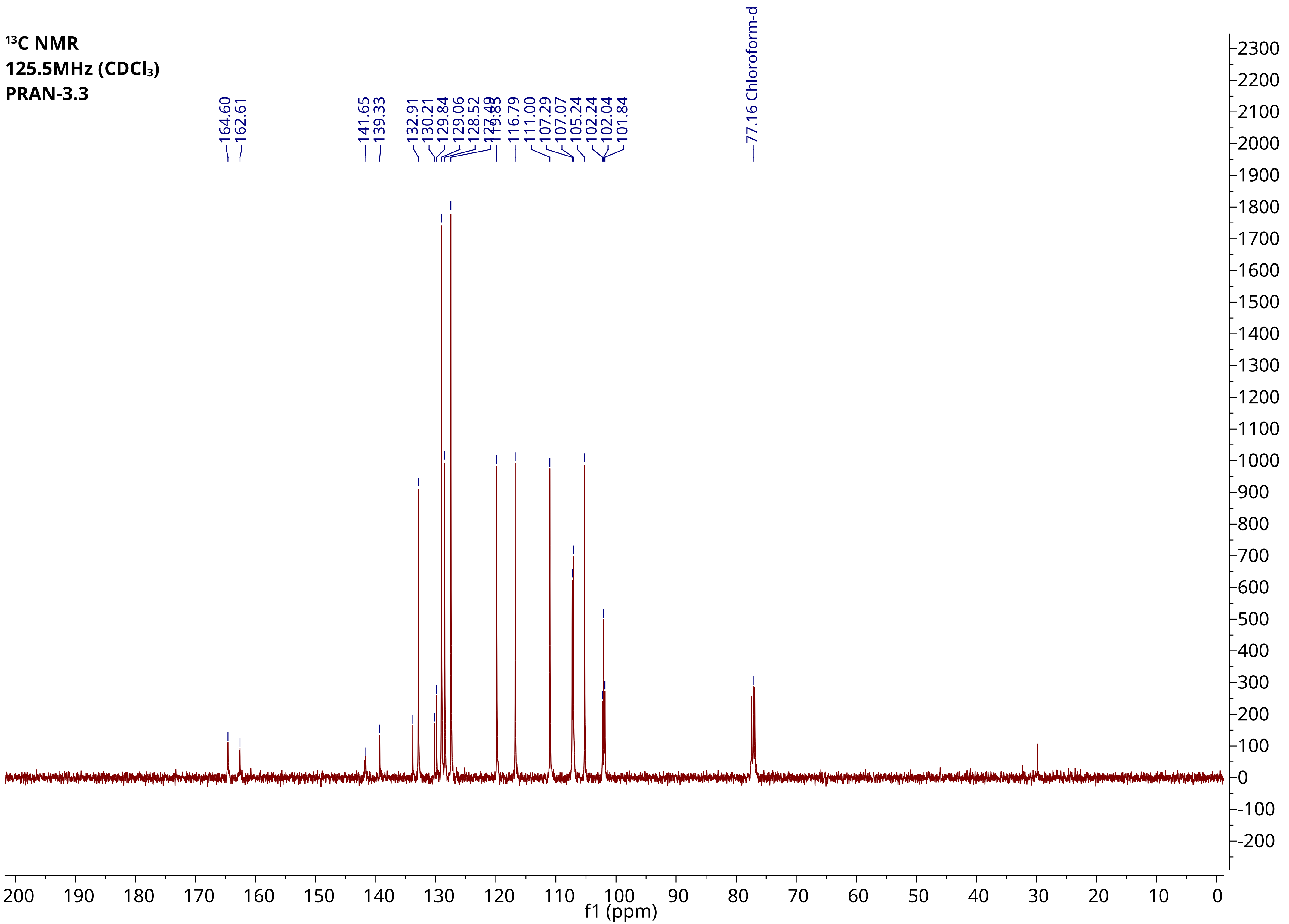

13C NMR
125.5MHz (CDCl3)
PRAN-3.3
164.60
162.61
141.65
139.33
132.91
130.21
129.84
129.06
128.52
127.49
119.85
116.79
111.00
107.29
107.07
105.24
102.24
102.04
101.84
77.16 Chloroform-d
f1 (ppm)

# ==== Shimadzu LCMSsolution Analysis Report ====

**Sample Name : PRAN-3.4**

Method

Column: Purospher RP-8
Mobile Phase A: H2O + 0.9% acetic acid
Mobile Phase B: ACN
% Pump B Concetrate: 50.0
Flow (ml/min): 0.6000

Detector A:SPD-20A
UV_1.Wavelength: 216
UV_2.Wavelength: 264
LC Program

| Time | Unit | Command | Value |
|---|---|---|---|
| 0.01 | Pumps | B.Conc | 50 |
| 15.00 | Pumps | B.Conc | 90 |
| 30.00 | Pumps | B.Conc | 90 |
| 30.01 | Pumps | B.Conc | 50 |
| 40.00 | Controller | Stop | |

MS Chromatogram

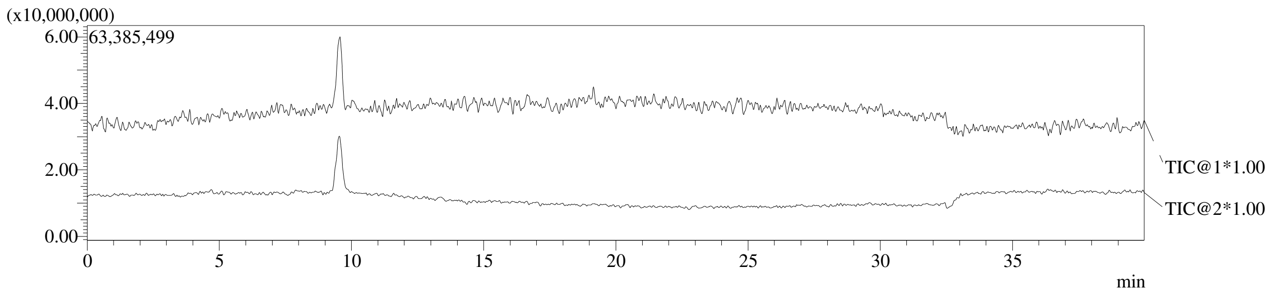


## <LC-UV Chromatogram>

Chromatogram

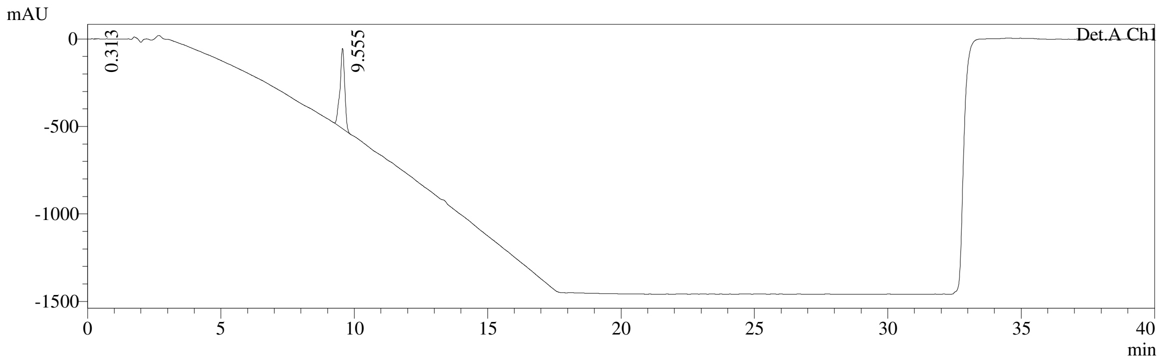

## Sample Name : PRAN-3.4

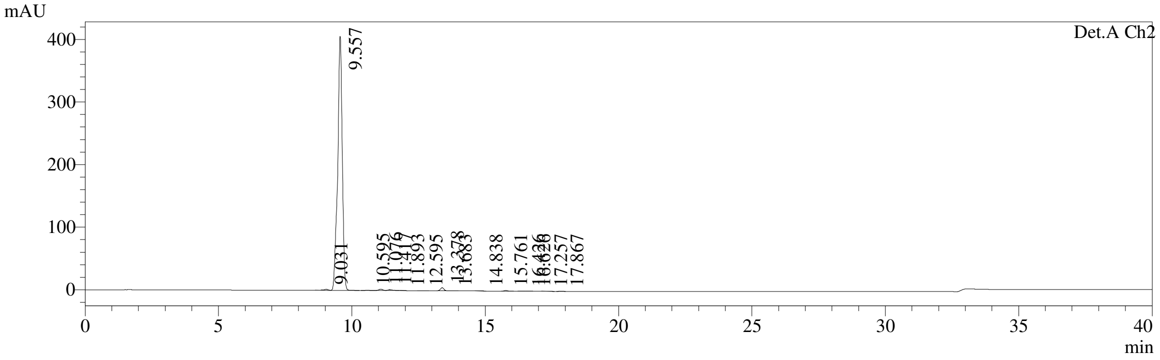


1 Det.A Ch1 / 216nm
2 Det.A Ch2 / 264nm

PeakTable

Detector A Ch2 264nm

| Peak# | Ret. Time | Area | Height | Area % | Height % |
|---|---|---|---|---|---|
| 1 | 9.031 | 21376 | 1615 | 0.425 | 0.385 |
| 2 | 9.557 | 4884335 | 405932 | 97.199 | 96.815 |
| 3 | 10.595 | 4654 | 366 | 0.093 | 0.087 |
| 4 | 11.076 | 23611 | 2215 | 0.470 | 0.528 |
| 5 | 11.417 | 19269 | 1365 | 0.383 | 0.326 |
| 6 | 11.893 | 1802 | 233 | 0.036 | 0.056 |
| 7 | 12.595 | 1747 | 95 | 0.035 | 0.023 |
| 8 | 13.378 | 46739 | 4980 | 0.930 | 1.188 |
| 9 | 13.683 | 3569 | 358 | 0.071 | 0.085 |
| 10 | 14.838 | 2054 | 245 | 0.041 | 0.058 |
| 11 | 15.761 | 7631 | 946 | 0.152 | 0.226 |
| 12 | 16.426 | 1532 | 157 | 0.030 | 0.037 |
| 13 | 16.626 | 2330 | 298 | 0.046 | 0.071 |
| 14 | 17.257 | 3228 | 342 | 0.064 | 0.082 |
| 15 | 17.867 | 1194 | 139 | 0.024 | 0.033 |
| Total | | 5025071 | 419287 | 100.000 | 100.000 |

MS Spectrum Graph

#:1 Ret.Time:Averaged 9.252-10.097(Scan#:855-933)
BG Mode:Averaged 31.308-33.779(2891-3119)
Mass Peaks:436 Base Peak:243.75(3643068) Polarity:Pos Segment1 - Event1

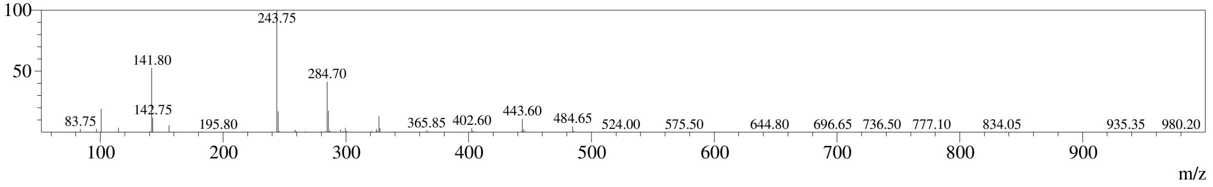


#:2 Ret.Time:Averaged 9.263-10.108(Scan#:856-934)
BG Mode:Averaged 31.319-33.779(2892-3120)
Mass Peaks:661 Base Peak:400.60(2587092) Polarity:Neg Segment1 - Event2

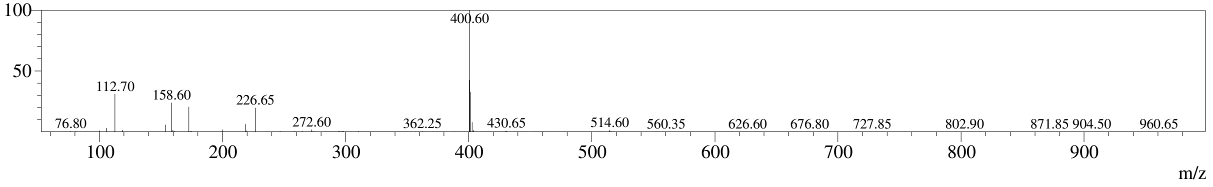

$^1H$ NMR 500MHz ($CDCl_3$)
PRAN-3.4

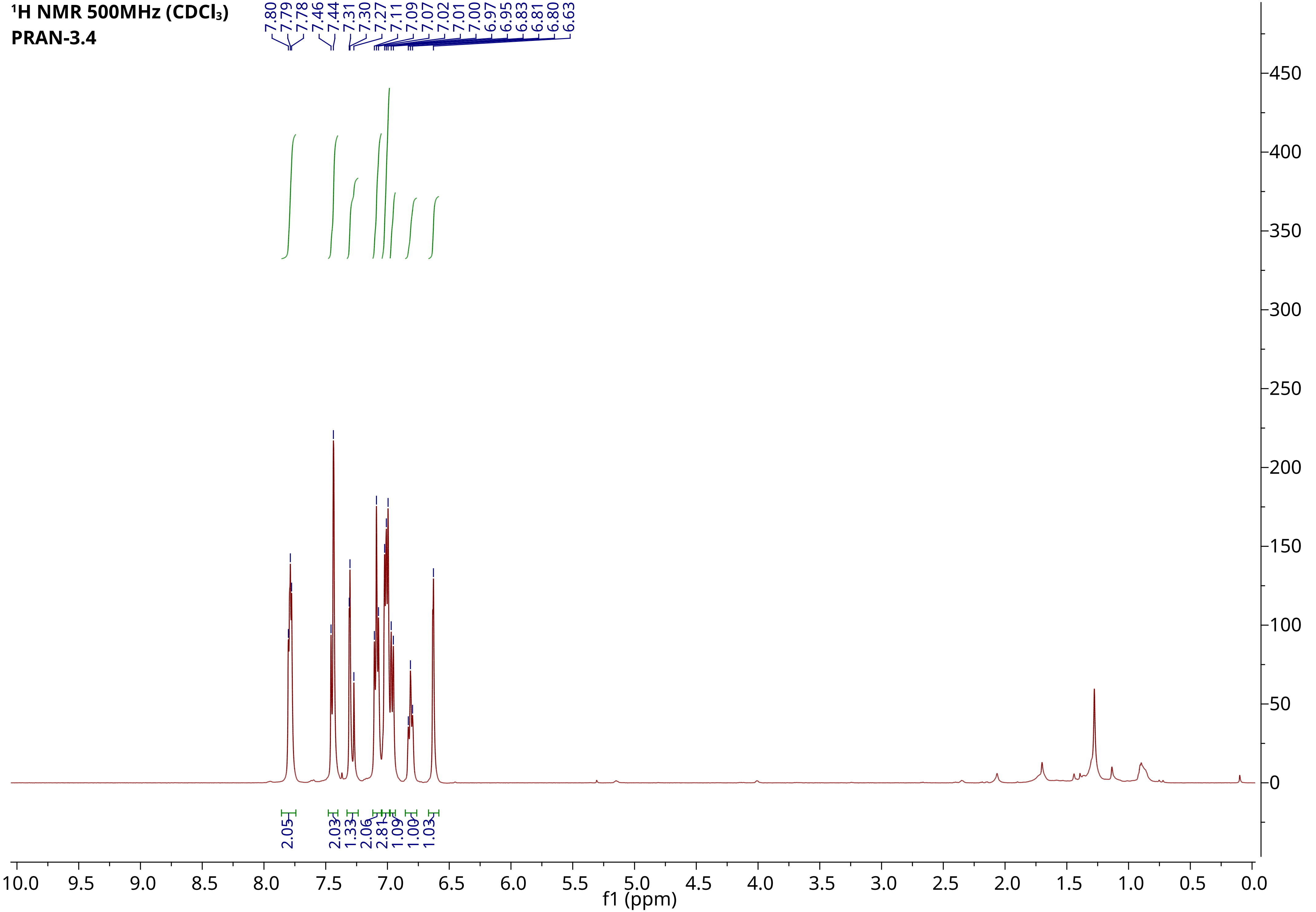

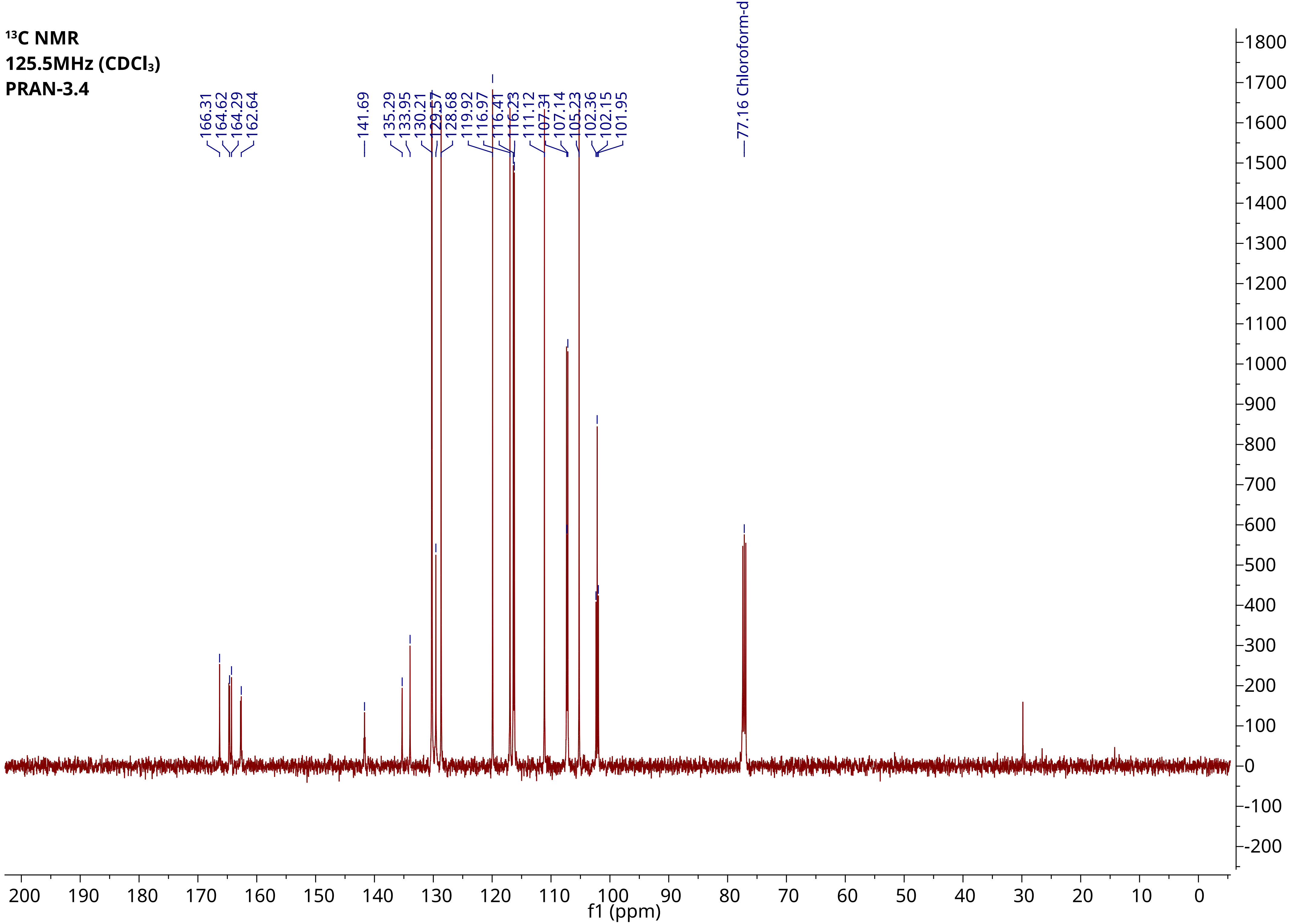

$^{13}$C NMR
125.5MHz ($CDCl_3$)
PRAN-3.4
166.31
164.62
164.29
162.64
141.69
135.29
133.95
130.21
129.57
128.68
119.92
116.97
116.41
116.23
111.12
107.31
107.14
105.23
102.36
102.15
101.95
77.16 Chloroform-d
f1 (ppm)

# ==== Shimadzu LCMSsolution Analysis Report ====

**Sample Name : PRAN-3.5**

Method

Column: Purospher RP-8
Mobile Phase A: H2O + 0.9% acetic acid
Mobile Phase B: ACN
% Pump B Concetrate: 50.0
Flow (ml/min): 0.6000

Detector A:SPD-20A
UV_1.Wavelength: 216
UV_2.Wavelength: 264
LC Program

| Time | Unit | Command | Value |
|---|---|---|---|
| 0.01 | Pumps | B.Conc | 50 |
| 15.00 | Pumps | B.Conc | 90 |
| 30.00 | Pumps | B.Conc | 90 |
| 30.01 | Pumps | B.Conc | 50 |
| 40.00 | Controller | Stop | |

MS Chromatogram

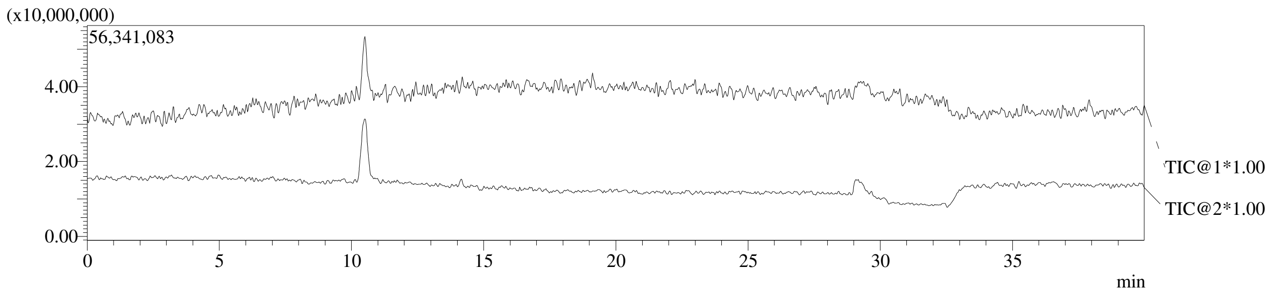


## <LC-UV Chromatogram>

Chromatogram

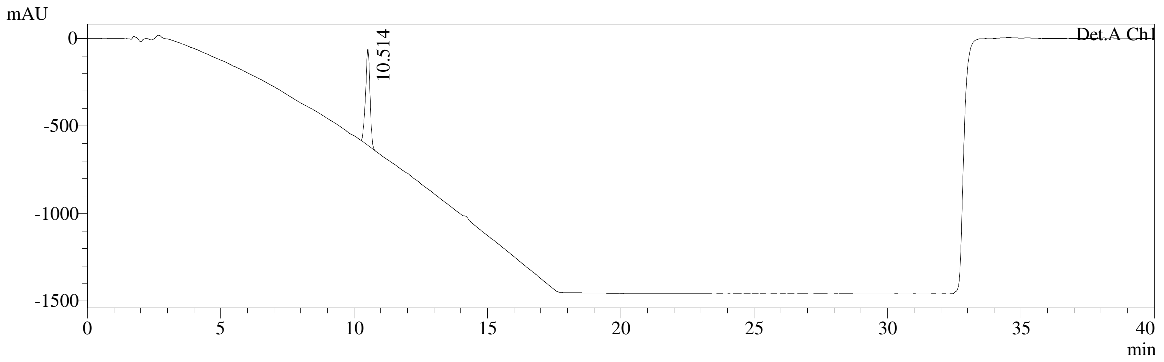

**Sample Name : PRAN-3.5**

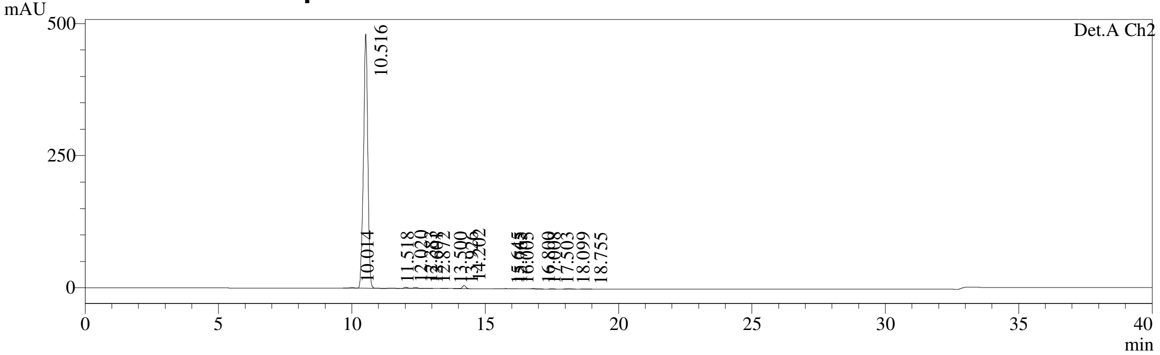


1 Det.A Ch1 / 216nm
2 Det.A Ch2 / 264nm

PeakTable

Detector A Ch2 264nm

| Peak# | Ret. Time | Area | Height | Area % | Height % |
|---|---|---|---|---|---|
| 1 | 10.014 | 15392 | 1269 | 0.275 | 0.255 |
| 2 | 10.516 | 5440565 | 481353 | 97.246 | 96.779 |
| 3 | 11.518 | 7419 | 489 | 0.133 | 0.098 |
| 4 | 12.020 | 22205 | 2259 | 0.397 | 0.454 |
| 5 | 12.383 | 19615 | 1860 | 0.351 | 0.374 |
| 6 | 12.601 | 1935 | 279 | 0.035 | 0.056 |
| 7 | 12.872 | 3196 | 405 | 0.057 | 0.081 |
| 8 | 13.500 | 1081 | 130 | 0.019 | 0.026 |
| 9 | 13.926 | 1384 | 211 | 0.025 | 0.042 |
| 10 | 14.202 | 51953 | 5936 | 0.929 | 1.193 |
| 11 | 15.645 | 2157 | 244 | 0.039 | 0.049 |
| 12 | 15.765 | 2634 | 360 | 0.047 | 0.072 |
| 13 | 16.005 | 2215 | 195 | 0.040 | 0.039 |
| 14 | 16.800 | 9728 | 1046 | 0.174 | 0.210 |
| 15 | 17.008 | 1184 | 166 | 0.021 | 0.033 |
| 16 | 17.503 | 3439 | 457 | 0.061 | 0.092 |
| 17 | 18.099 | 6368 | 504 | 0.114 | 0.101 |
| 18 | 18.755 | 2189 | 211 | 0.039 | 0.042 |
| Total | | 5594658 | 497374 | 100.000 | 100.000 |

MS Spectrum Graph

#:1 Ret.Time:Averaged 10.097-11.115(Scan#:933-1027)
BG Mode:Averaged 20.800-38.532(1921-3557)
Mass Peaks:435 Base Peak:82.90(9531131) Polarity:Pos Segment1 - Event1

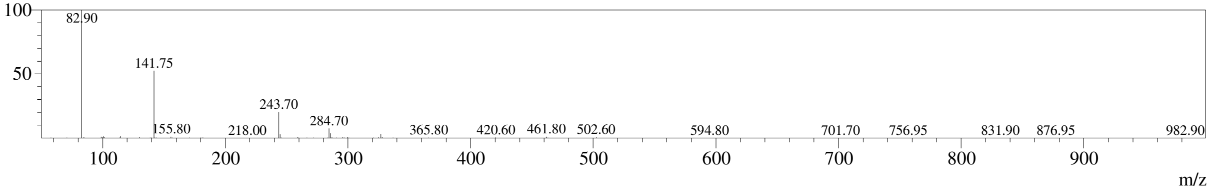


#:2 Ret.Time:Averaged 10.108-11.126(Scan#:934-1028)
BG Mode:Averaged 20.811-38.532(1922-3558)
Mass Peaks:518 Base Peak:176.65(1824030) Polarity:Neg Segment1 - Event2

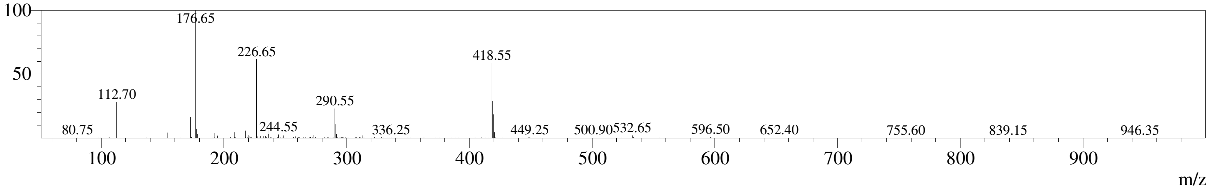

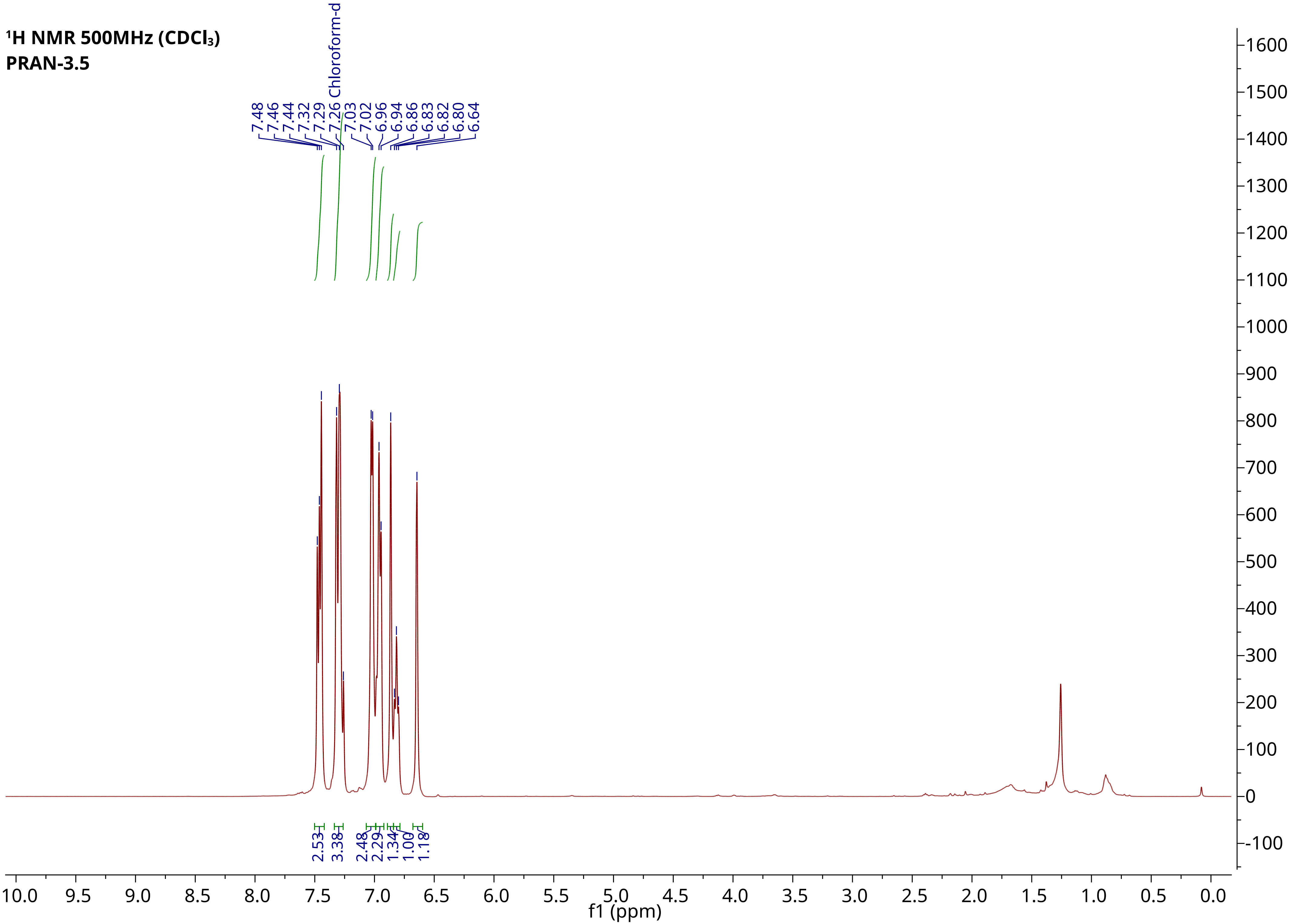

1H NMR 500MHz (CDCl3)
PRAN-3.5
7.48
7.46
7.44
7.32
7.29
7.26 Chloroform-d
7.03
7.02
6.96
6.94
6.86
6.83
6.82
6.80
6.64
2.53
3.38
2.48
2.29
1.34
1.00
1.18
f1 (ppm)

13C NMR
125.5MHz ($CDCl_3$)
PRAN-3.5

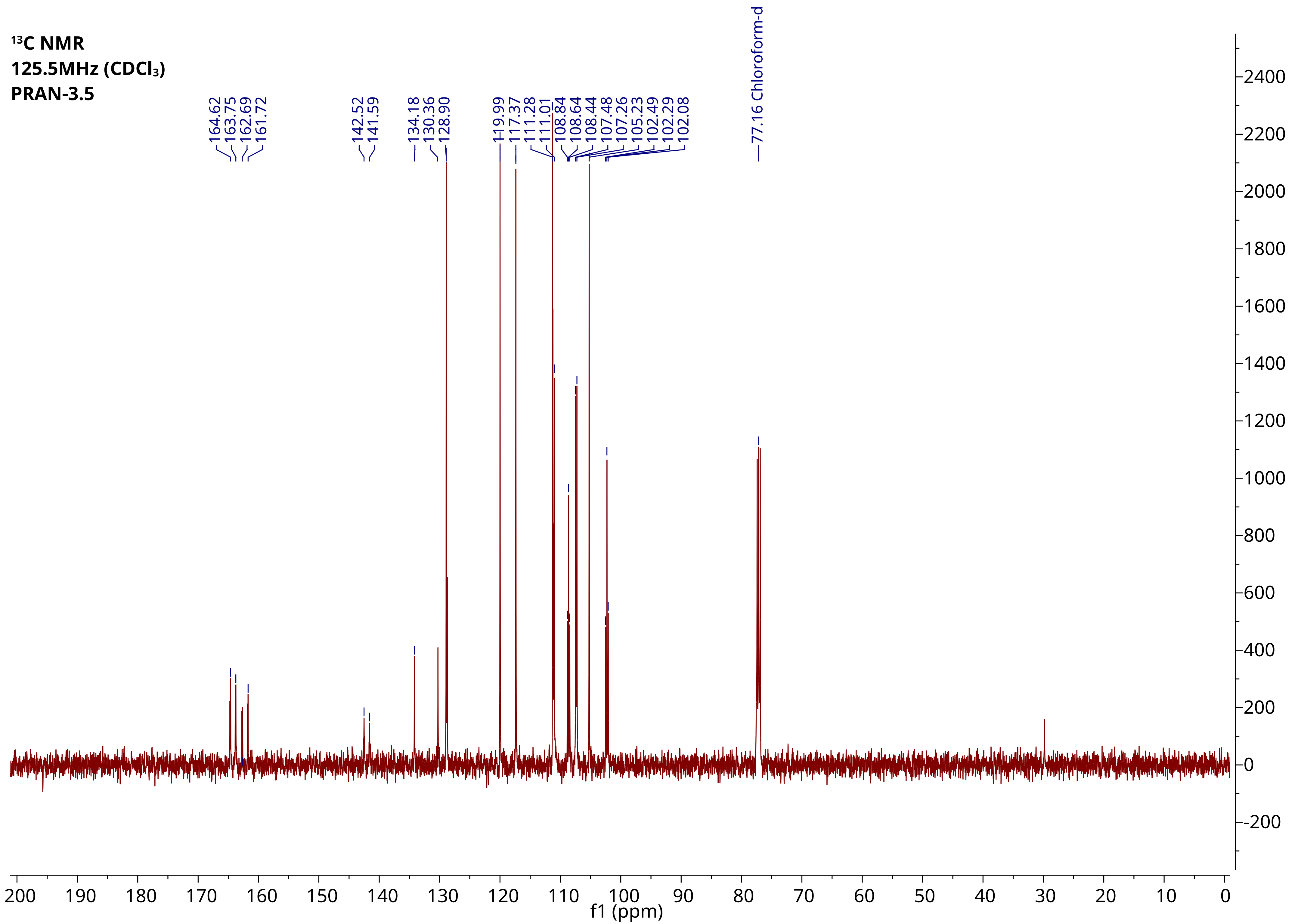

# ==== Shimadzu LCMSsolution Analysis Report ====

**Sample Name : PRAN-3.6**

Method

Column: Purospher RP-8
Mobile Phase A: H2O + 0.9% acetic acid
Mobile Phase B: ACN
% Pump B Concetrate: 50.0
Flow (ml/min): 0.6000

Detector A:SPD-20A
UV_1.Wavelength: 216
UV_2.Wavelength: 264
LC Program

| Time | Unit | Command | Value |
|---|---|---|---|
| 0.01 | Pumps | B.Conc | 50 |
| 15.00 | Pumps | B.Conc | 90 |
| 30.00 | Pumps | B.Conc | 90 |
| 30.01 | Pumps | B.Conc | 50 |
| 40.00 | Controller | Stop | |

MS Chromatogram

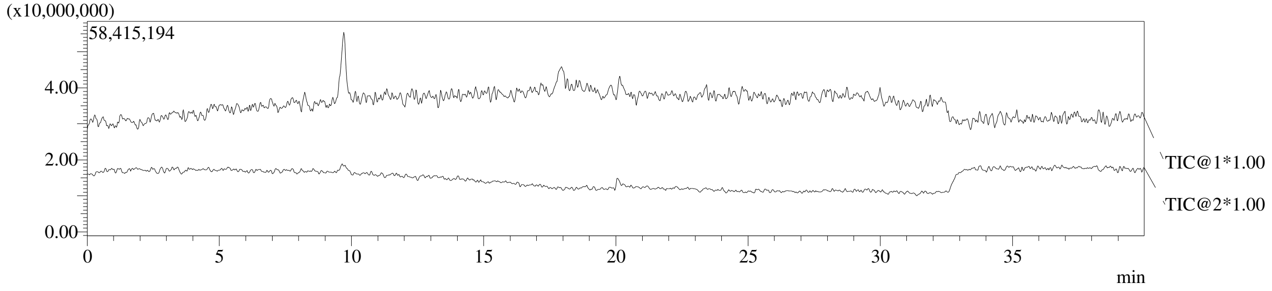


## <LC-UV Chromatogram>

Chromatogram

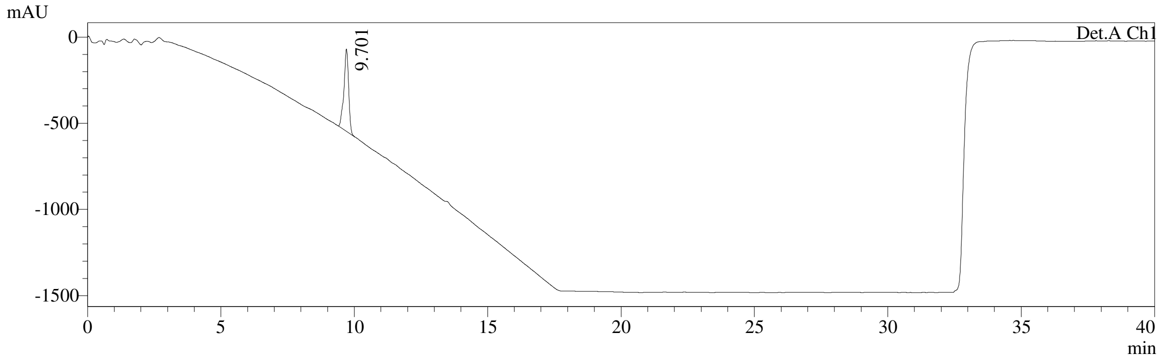

**Sample Name : PRAN-3.6**

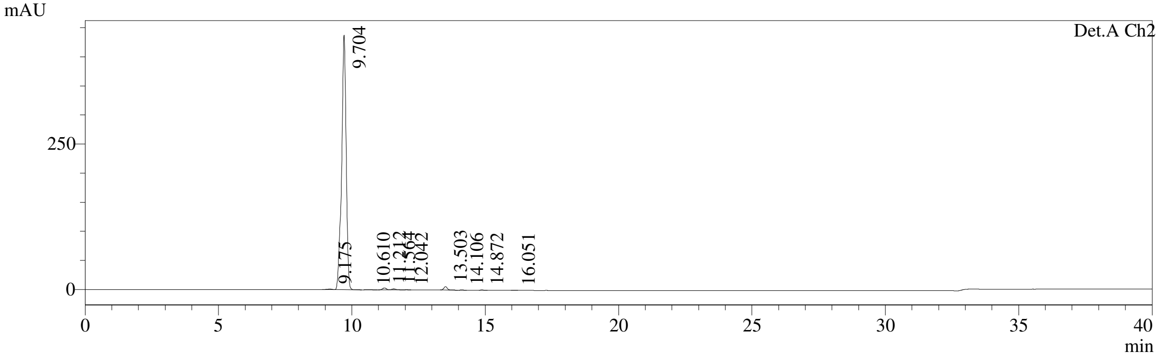


1 Det.A Ch1 / 216nm
2 Det.A Ch2 / 264nm

PeakTable

Detector A Ch2 264nm

| Peak# | Ret. Time | Area | Height | Area % | Height % |
|---|---|---|---|---|---|
| 1 | 9.175 | 16628 | 1274 | 0.311 | 0.282 |
| 2 | 9.704 | 5206333 | 437573 | 97.376 | 96.939 |
| 3 | 10.610 | 5591 | 436 | 0.105 | 0.097 |
| 4 | 11.212 | 34902 | 3339 | 0.653 | 0.740 |
| 5 | 11.564 | 21989 | 1866 | 0.411 | 0.413 |
| 6 | 12.042 | 2619 | 328 | 0.049 | 0.073 |
| 7 | 13.503 | 53486 | 5897 | 1.000 | 1.306 |
| 8 | 14.106 | 1700 | 206 | 0.032 | 0.046 |
| 9 | 14.872 | 2096 | 264 | 0.039 | 0.058 |
| 10 | 16.051 | 1265 | 209 | 0.024 | 0.046 |
| Total | | 5346609 | 451392 | 100.000 | 100.000 |

MS Spectrum Graph

#:1 Ret.Time:Averaged 9.425-9.837(Scan#:871-909)
BG Mode:Averaged 25.458-26.735(2351-2469)
Mass Peaks:367 Base Peak:243.75(5816097) Polarity:Pos Segment1 - Event1

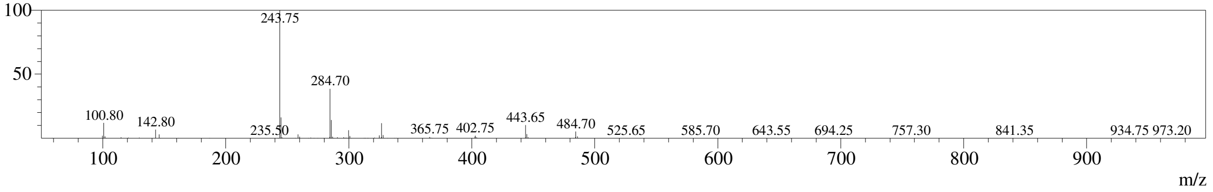


#:2 Ret.Time:Averaged 9.436-9.848(Scan#:872-910)
BG Mode:Averaged 25.469-26.735(2352-2470)
Mass Peaks:433 Base Peak:226.60(5882677) Polarity:Neg Segment1 - Event2

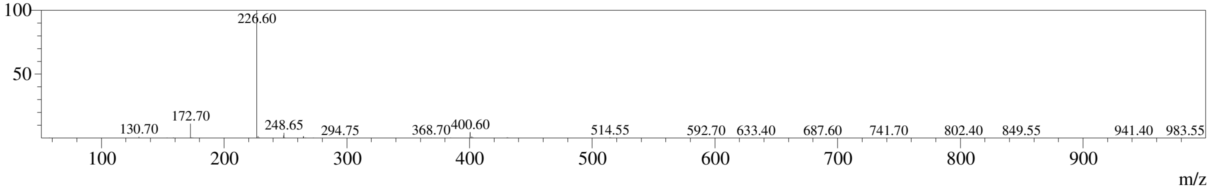

**¹H NMR 500MHz ($CDCl_3$)**
**PRAN-3.6**

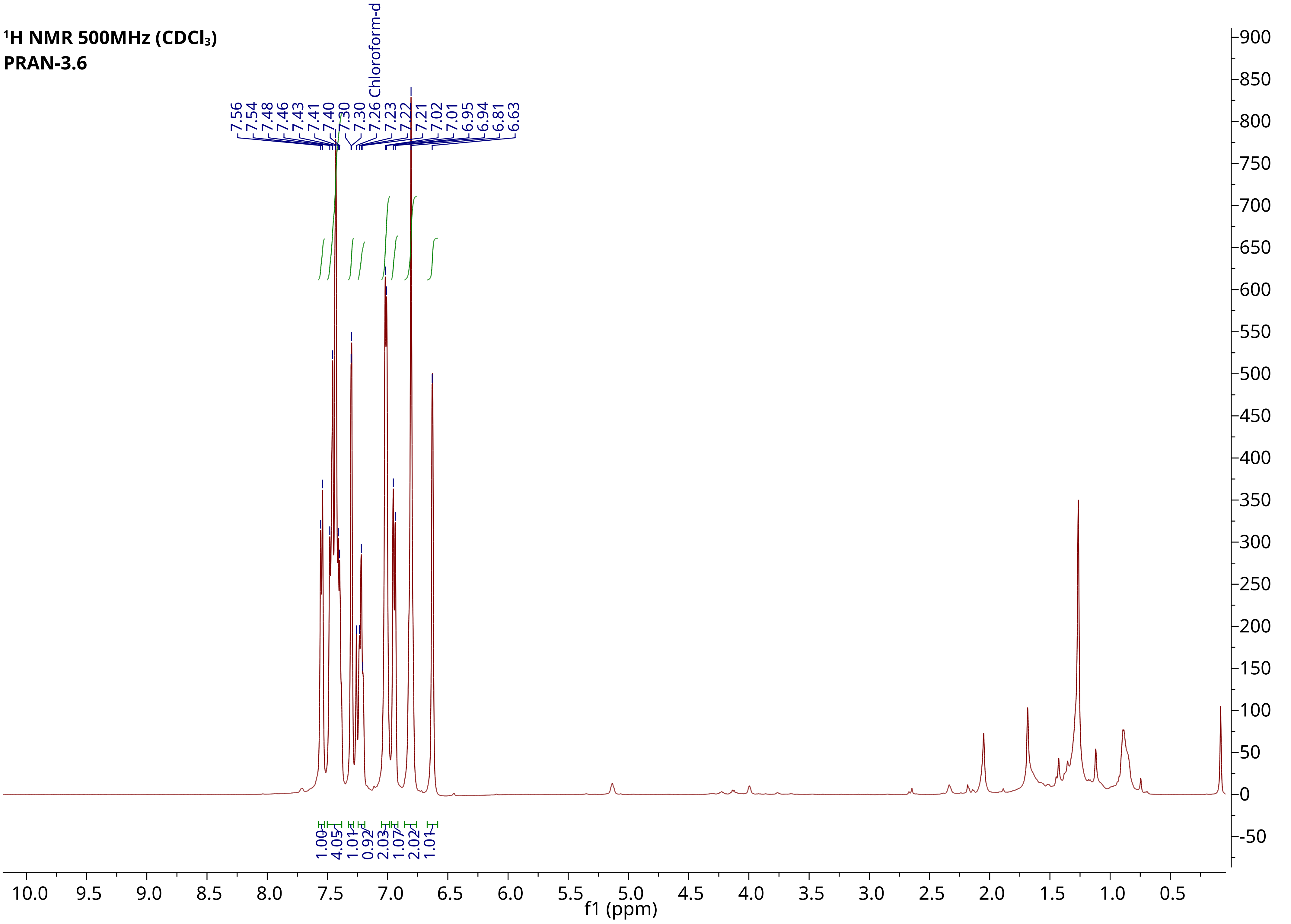

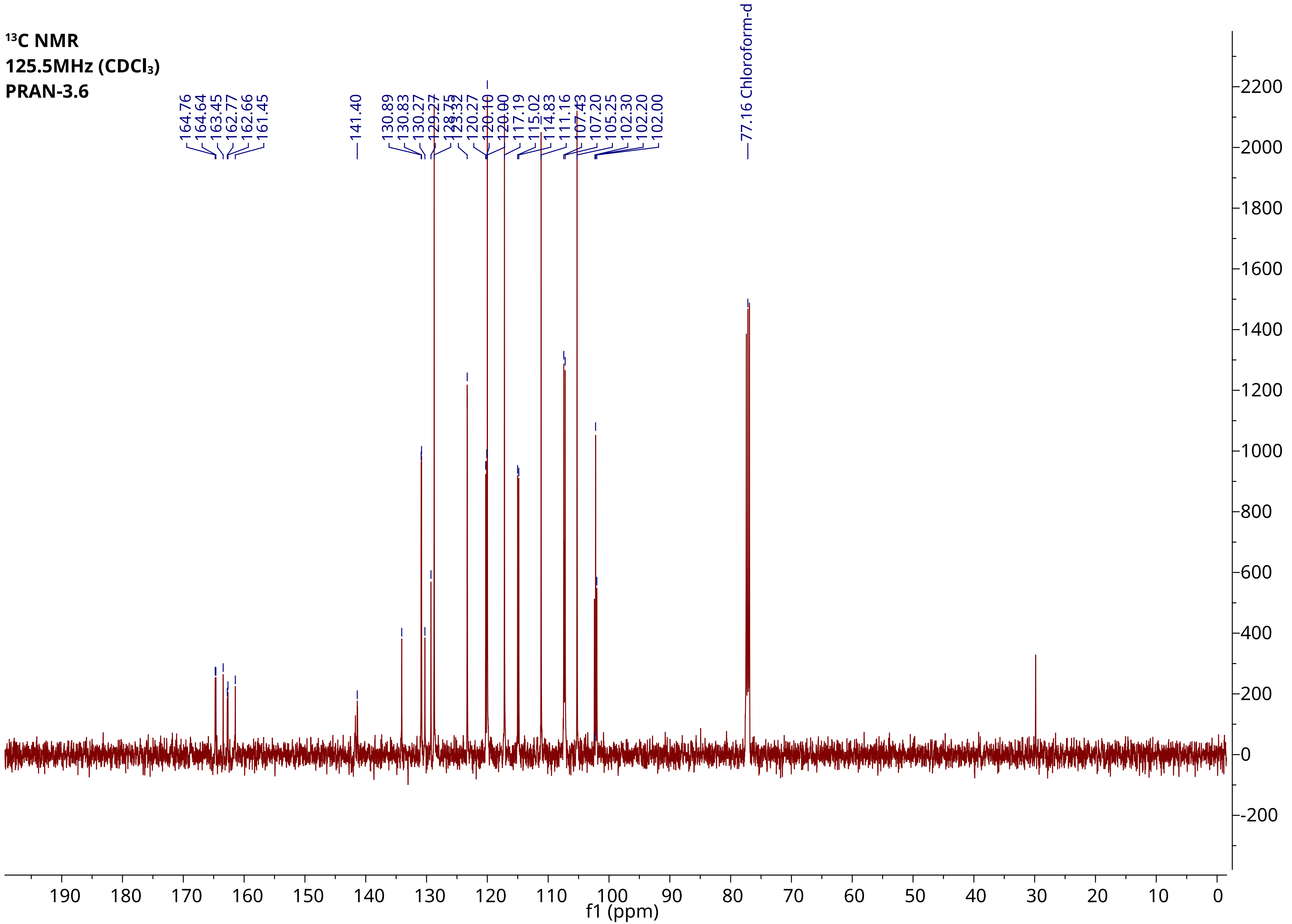

$^{13}$C NMR
125.5MHz (CDCl$_3$)
PRAN-3.6
164.76
164.64
163.45
162.77
162.66
161.45
141.40
130.89
130.83
130.27
129.27
128.75
123.32
120.27
120.10
120.00
117.19
115.02
114.83
111.16
107.43
107.20
105.25
102.30
102.20
102.00
77.16 Chloroform-d
f1 (ppm)

# ==== Shimadzu LCMSsolution Analysis Report ====

**Sample Name : PRAN-3.7**

Method

Column: Purospher RP-8
Mobile Phase A: H2O + 0.9% acetic acid
Mobile Phase B: ACN
% Pump B Concetrate: 50.0
Flow (ml/min): 0.6000

Detector A:SPD-20A
UV_1.Wavelength: 216
UV_2.Wavelength: 264
LC Program

| Time | Unit | Command | Value |
|---|---|---|---|
| 0.01 | Pumps | B.Conc | 50 |
| 15.00 | Pumps | B.Conc | 90 |
| 30.00 | Pumps | B.Conc | 90 |
| 30.01 | Pumps | B.Conc | 50 |
| 40.00 | Controller | Stop | |

MS Chromatogram

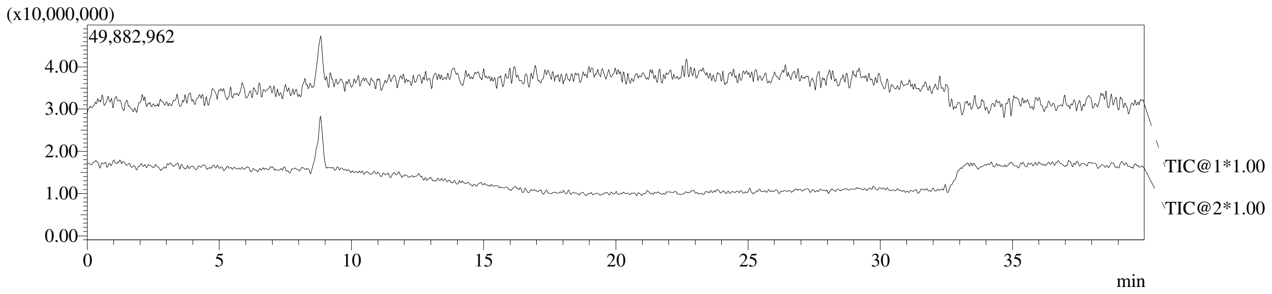


## <LC-UV Chromatogram>

Chromatogram

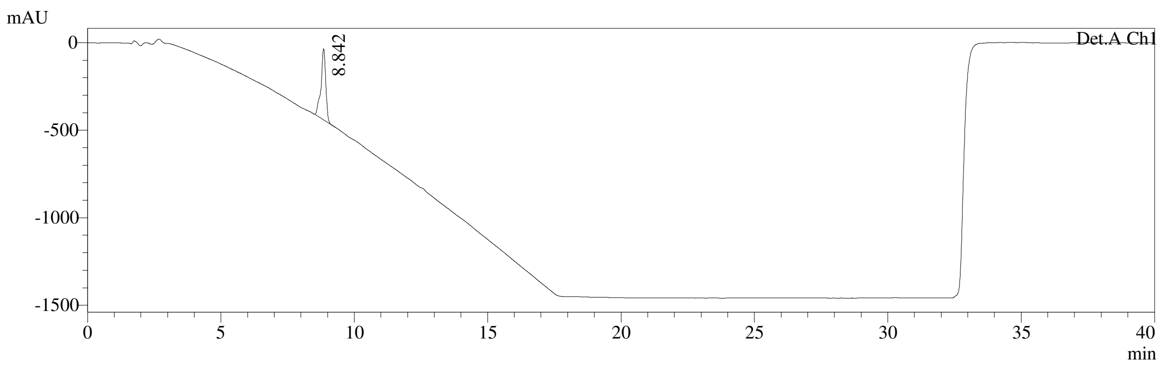

## Sample Name : PRAN-3.7

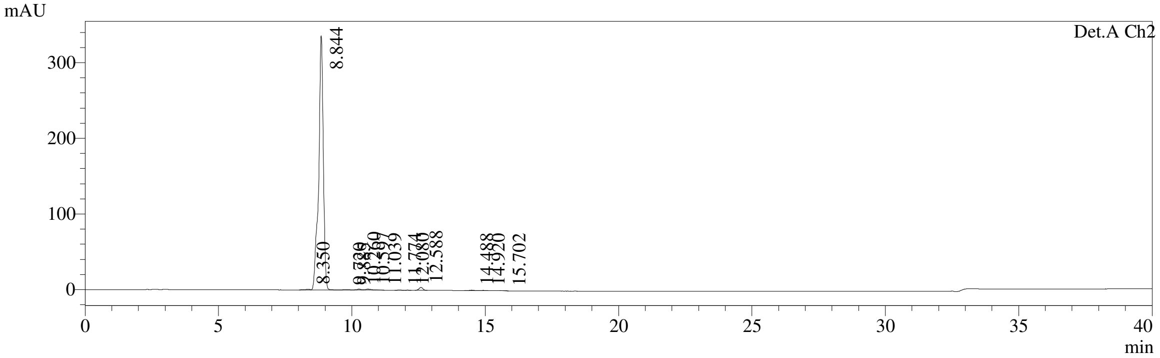


1 Det.A Ch1 / 216nm
2 Det.A Ch2 / 264nm

PeakTable

Detector A Ch2 264nm

| Peak# | Ret. Time | Area | Height | Area % | Height % |
|---|---|---|---|---|---|
| 1 | 8.350 | 10765 | 856 | 0.257 | 0.248 |
| 2 | 8.844 | 4105306 | 335919 | 97.827 | 97.500 |
| 3 | 9.720 | 2114 | 197 | 0.050 | 0.057 |
| 4 | 9.885 | 1277 | 173 | 0.030 | 0.050 |
| 5 | 10.260 | 11601 | 1111 | 0.276 | 0.322 |
| 6 | 10.597 | 15184 | 1114 | 0.362 | 0.323 |
| 7 | 11.039 | 1181 | 147 | 0.028 | 0.043 |
| 8 | 11.774 | 1856 | 184 | 0.044 | 0.053 |
| 9 | 12.080 | 1073 | 134 | 0.026 | 0.039 |
| 10 | 12.588 | 37488 | 3727 | 0.893 | 1.082 |
| 11 | 14.488 | 5503 | 589 | 0.131 | 0.171 |
| 12 | 14.920 | 2053 | 267 | 0.049 | 0.078 |
| 13 | 15.702 | 1116 | 114 | 0.027 | 0.033 |
| Total | | 4196517 | 344532 | 100.000 | 100.000 |

MS Spectrum Graph

#:1 Ret.Time:Averaged 8.320-9.165(Scan#:769-847)
BG Mode:Averaged 15.448-17.738(1427-1639)
Mass Peaks:312 Base Peak:243.75(1499707) Polarity:Pos Segment1 - Event1

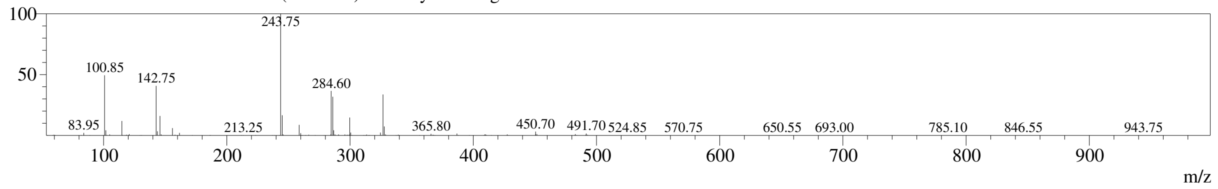


#:2 Ret.Time:Averaged 8.331-9.176(Scan#:770-848)
BG Mode:Averaged 15.459-17.738(1428-1640)
Mass Peaks:523 Base Peak:226.65(3798619) Polarity:Neg Segment1 - Event2

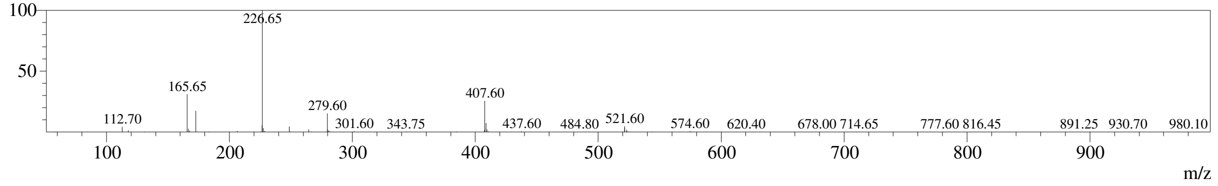

¹H NMR 500MHz (CDCl₃)

**PRAN-3.7**

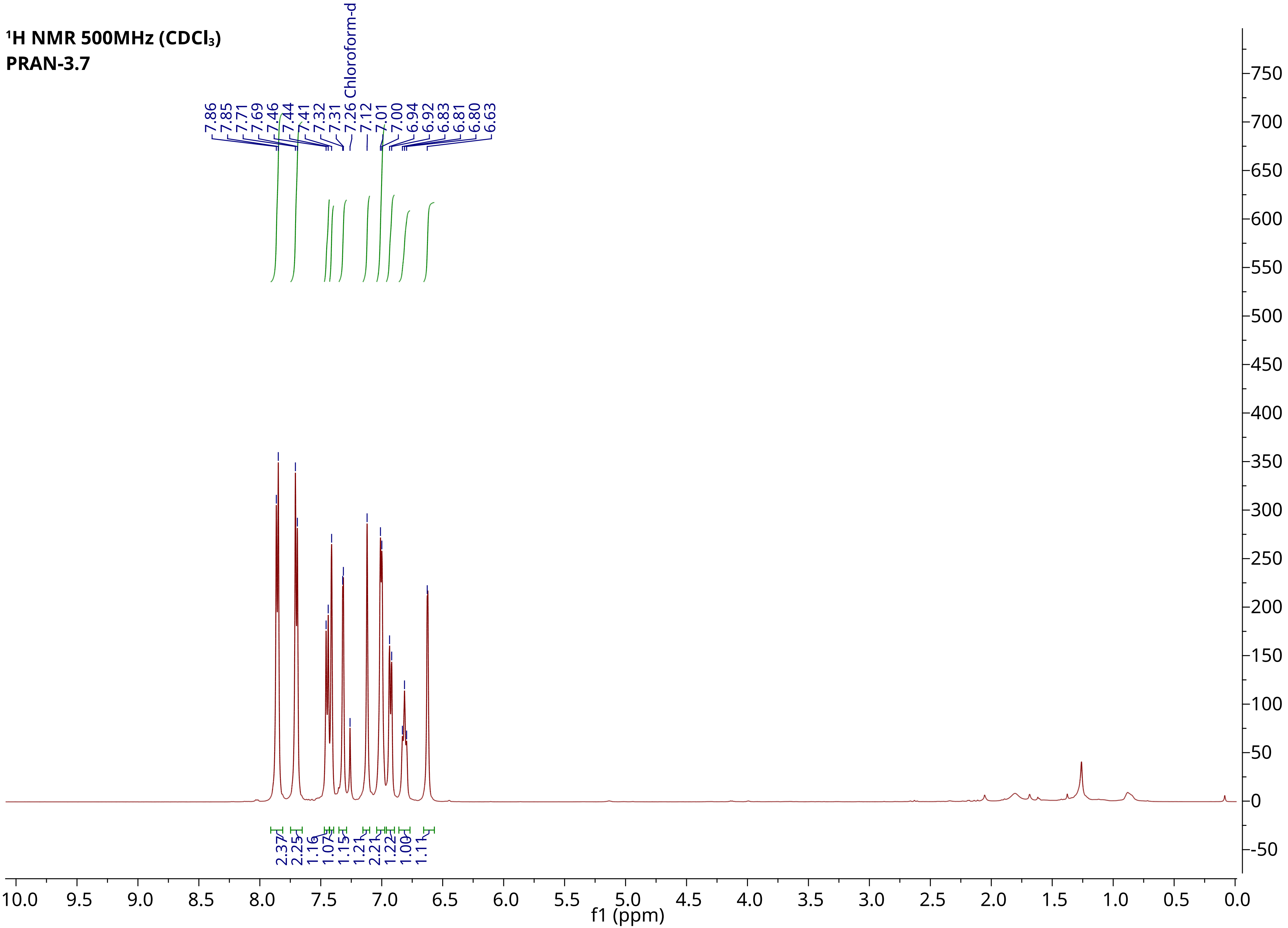

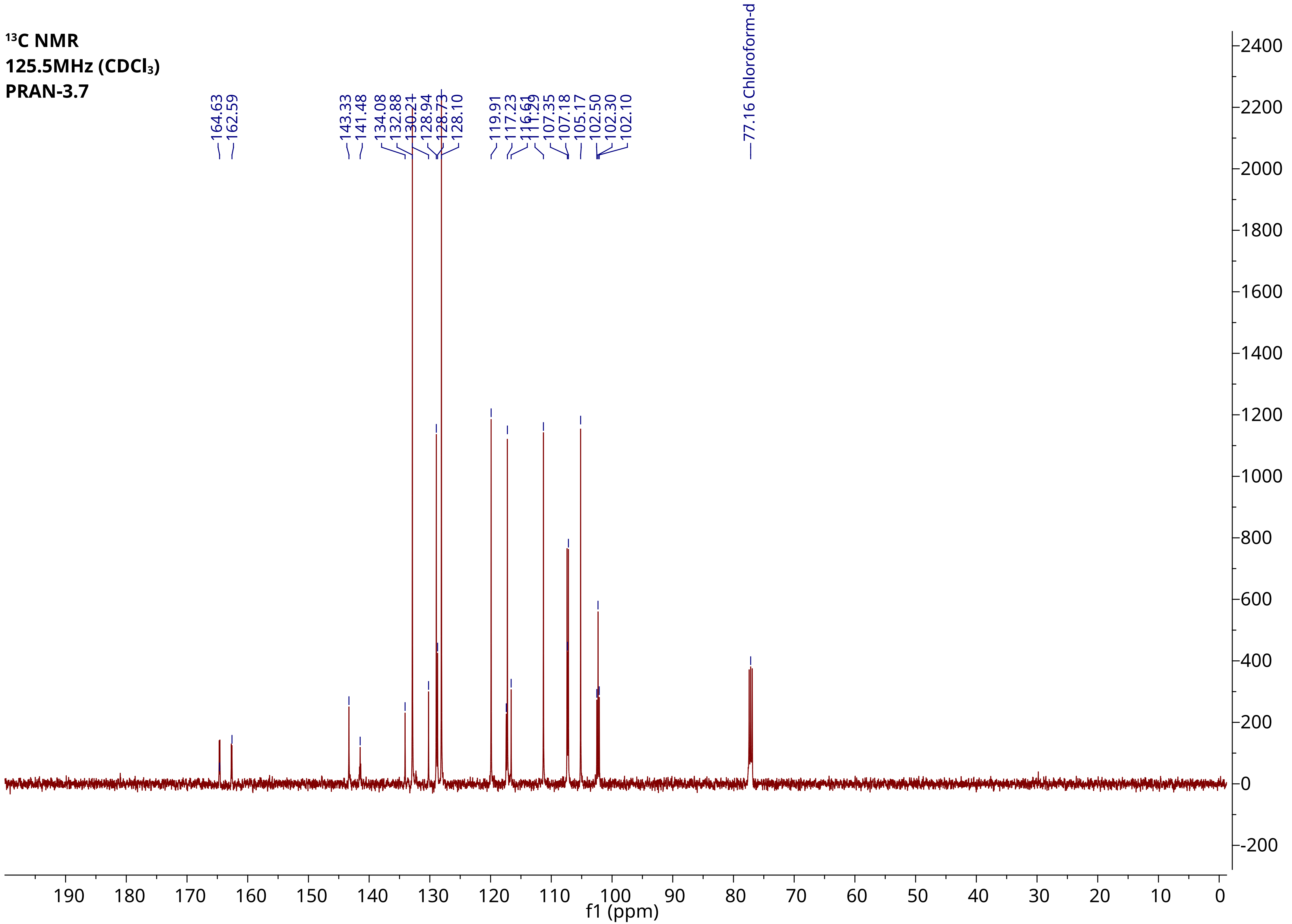

$^{13}$C NMR
125.5MHz ($CDCl_3$)
PRAN-3.7
164.63
162.59
143.33
141.48
134.08
132.88
130.21
128.94
128.73
128.10
119.91
117.23
116.61
111.29
107.35
107.18
105.17
102.50
102.30
102.10
77.16 Chloroform-d
f1 (ppm)

# ==== Shimadzu LCMSsolution Analysis Report ====

**Sample Name : PRAN-3.8**

Method

Column: Purospher RP-8
Mobile Phase A: H2O + 0.9% acetic acid
Mobile Phase B: ACN
% Pump B Concetrate: 50.0
Flow (ml/min): 0.6000

Detector A:SPD-20A
UV_1.Wavelength: 216
UV_2.Wavelength: 264
LC Program

| Time | Unit | Command | Value |
|---|---|---|---|
| 0.01 | Pumps | B.Conc | 50 |
| 15.00 | Pumps | B.Conc | 90 |
| 30.00 | Pumps | B.Conc | 90 |
| 30.01 | Pumps | B.Conc | 50 |
| 40.00 | Controller | Stop | |

MS Chromatogram

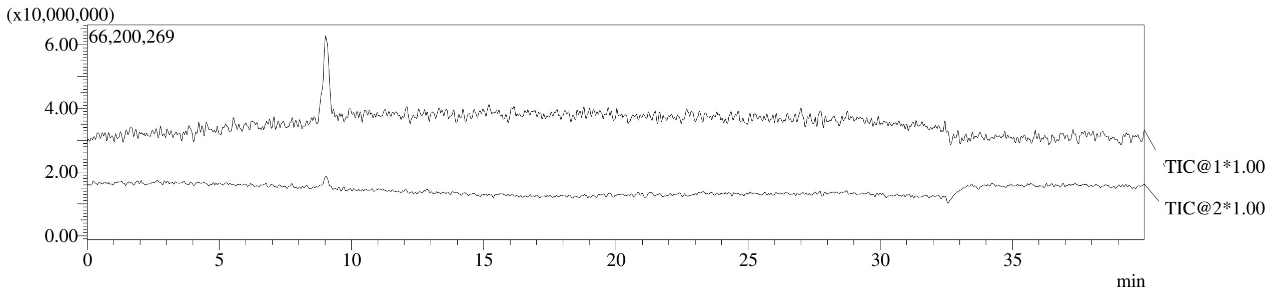


## <LC-UV Chromatogram>

Chromatogram

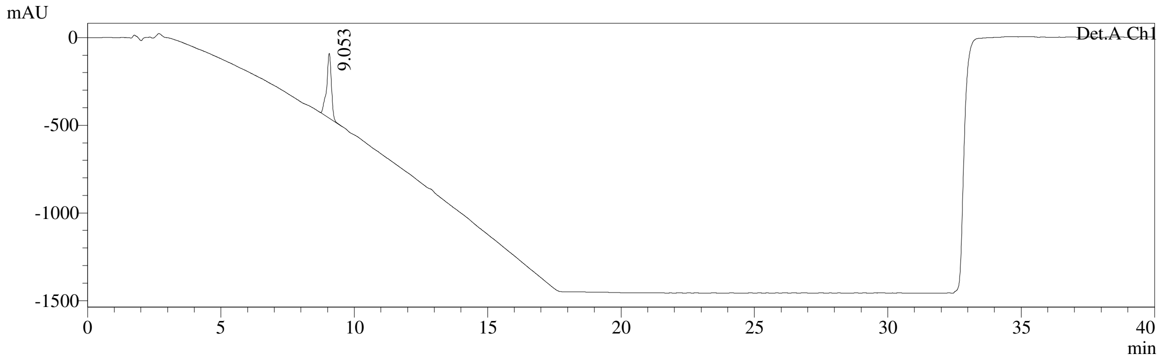

## Sample Name : PRAN-3.8

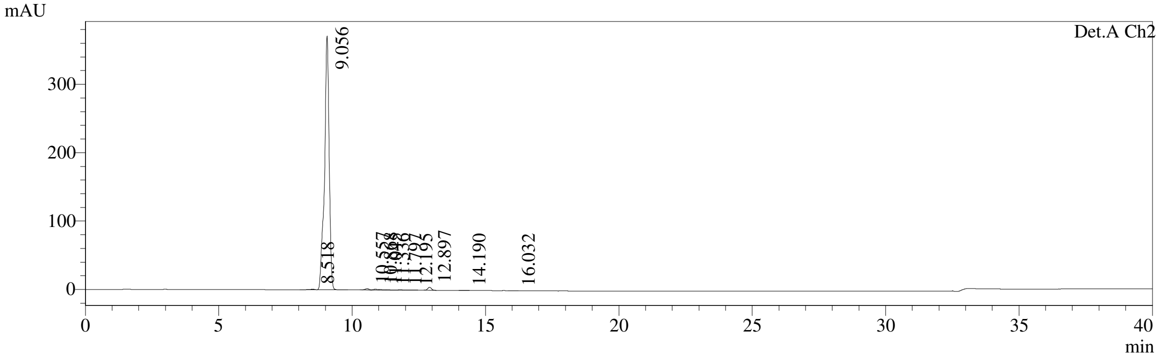


1 Det.A Ch1 / 216nm
2 Det.A Ch2 / 264nm

PeakTable

Detector A Ch2 264nm

| Peak# | Ret. Time | Area | Height | Area % | Height % |
|---|---|---|---|---|---|
| 1 | 8.518 | 14099 | 999 | 0.303 | 0.262 |
| 2 | 9.056 | 4541343 | 371329 | 97.610 | 97.296 |
| 3 | 10.557 | 21097 | 1870 | 0.453 | 0.490 |
| 4 | 10.868 | 14191 | 1204 | 0.305 | 0.316 |
| 5 | 11.045 | 7113 | 662 | 0.153 | 0.173 |
| 6 | 11.336 | 2857 | 307 | 0.061 | 0.080 |
| 7 | 11.797 | 4439 | 411 | 0.095 | 0.108 |
| 8 | 12.195 | 2378 | 155 | 0.051 | 0.041 |
| 9 | 12.897 | 41935 | 4419 | 0.901 | 1.158 |
| 10 | 14.190 | 1566 | 161 | 0.034 | 0.042 |
| 11 | 16.032 | 1515 | 130 | 0.033 | 0.034 |
| Total | | 4652533 | 381647 | 100.000 | 100.000 |

MS Spectrum Graph

#:1 Ret.Time:Averaged 8.667-9.685(Scan#:801-895)
BG Mode:Averaged 17.138-26.989(1583-2493)
Mass Peaks:430 Base Peak:82.85(7111445) Polarity:Pos Segment1 - Event1

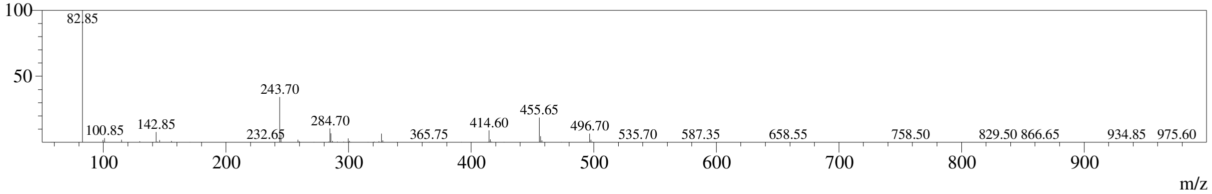


#:2 Ret.Time:Averaged 8.678-9.696(Scan#:802-896)
BG Mode:Averaged 17.149-26.989(1584-2494)
Mass Peaks:382 Base Peak:226.65(4115992) Polarity:Neg Segment1 - Event2

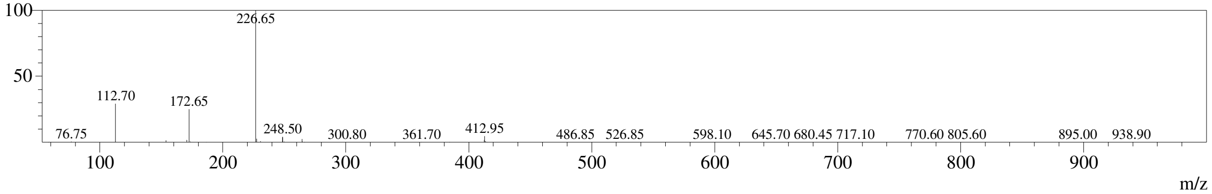

**¹H NMR**
**500MHz ($CDCl_3$)**
**PRAN-3.8**

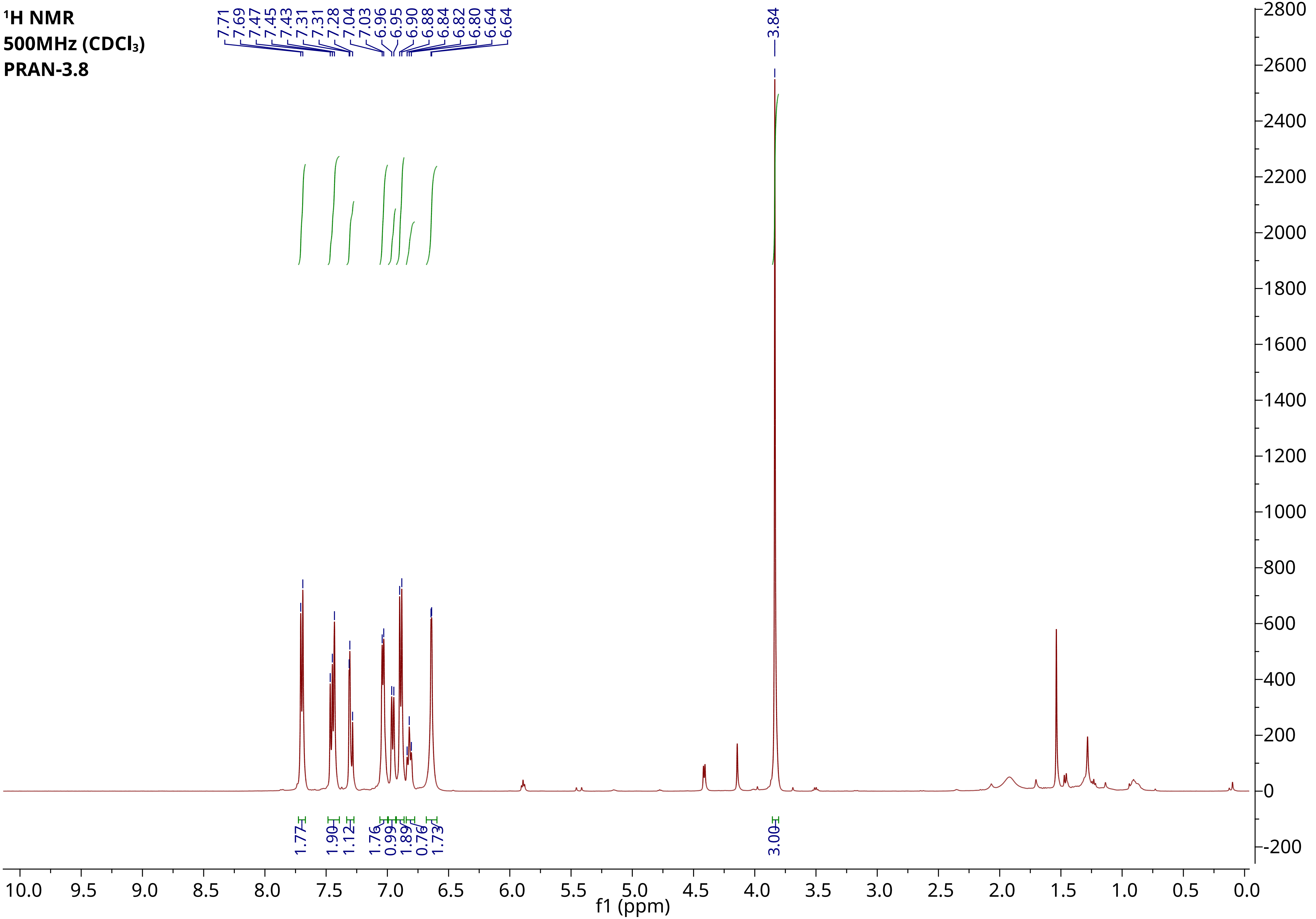

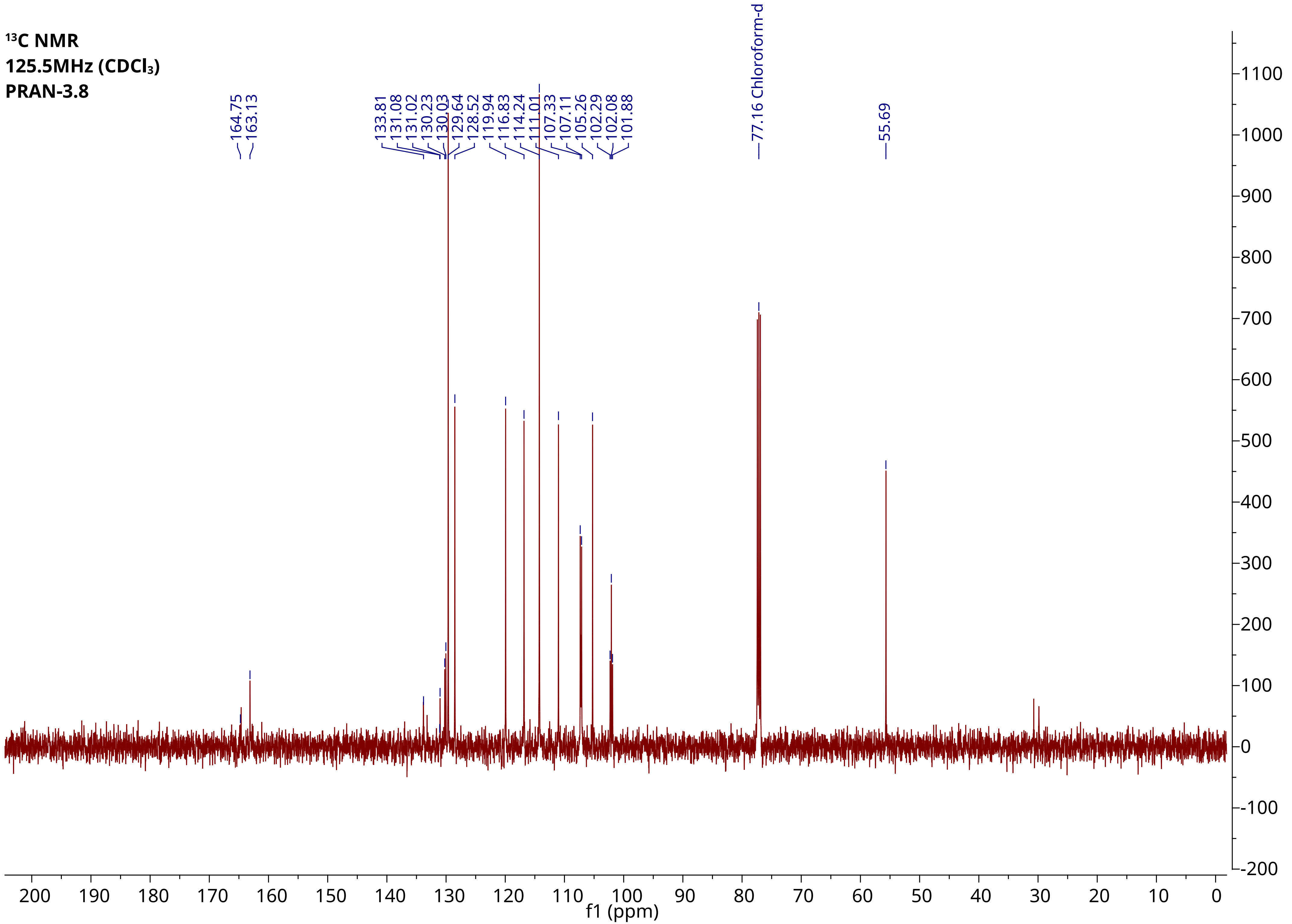

13C NMR
125.5MHz (CDCl3)
PRAN-3.8
164.75
163.13
133.81
131.08
131.02
130.23
130.03
129.64
128.52
119.94
116.83
114.24
111.01
107.33
107.11
105.26
102.29
102.08
101.88
77.16 Chloroform-d
55.69
f1 (ppm)

# ==== Shimadzu LCMSsolution Analysis Report ====

**Sample Name : PRAN-3.9**

Method

Column: Purospher RP-8
Mobile Phase A: $H_2O$ + 0.9% acetic acid
Mobile Phase B: ACN
% Pump B Concetrate: 50.0
Flow (ml/min): 0.6000

Detector A:SPD-20A
UV_1.Wavelength: 216
UV_2.Wavelength: 264
LC Program

| Time | Unit | Command | Value |
|---|---|---|---|
| 0.01 | Pumps | B.Conc | 50 |
| 15.00 | Pumps | B.Conc | 90 |
| 30.00 | Pumps | B.Conc | 90 |
| 30.01 | Pumps | B.Conc | 50 |
| 40.00 | Controller | Stop | |

MS Chromatogram

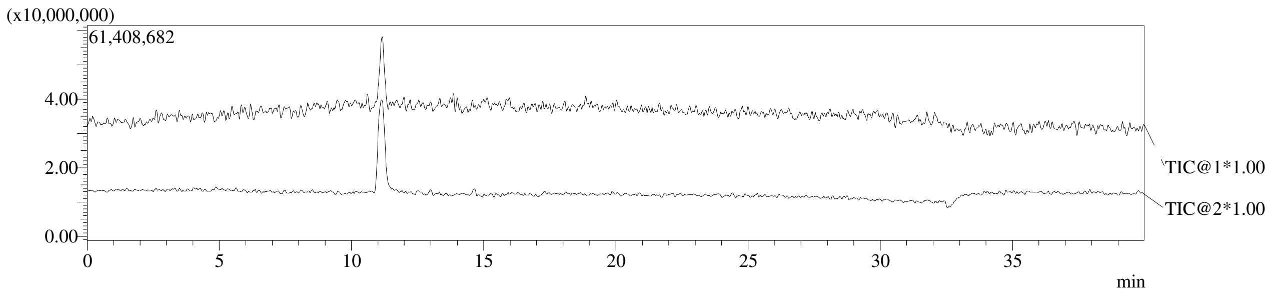


## <LC-UV Chromatogram>

Chromatogram

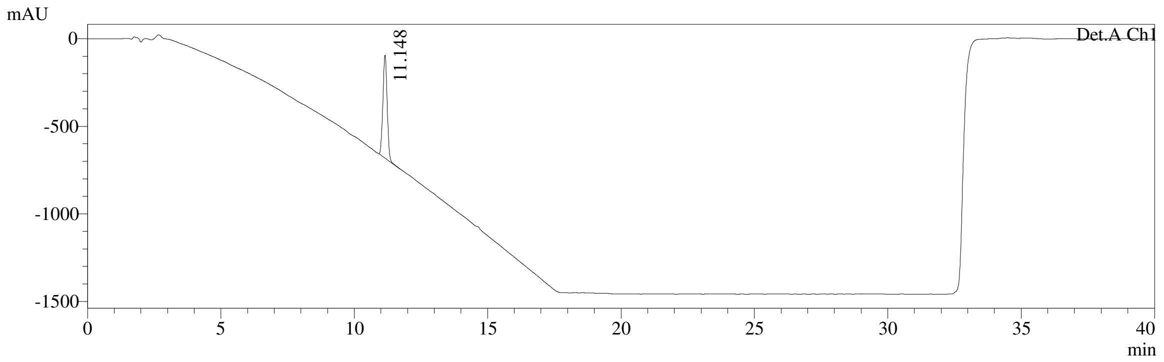

## Sample Name : PRAN-3.9

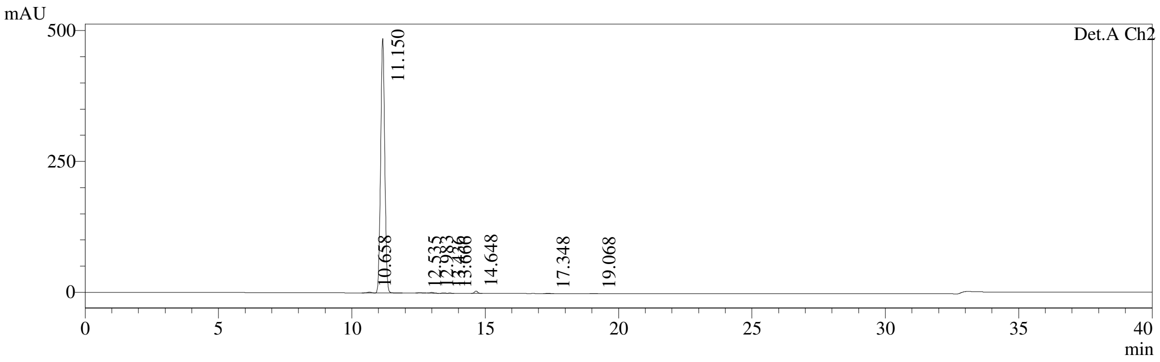


1 Det.A Ch1 / 216nm
2 Det.A Ch2 / 264nm

PeakTable

Detector A Ch2 264nm

| Peak# | Ret. Time | Area | Height | Area % | Height % |
|---|---|---|---|---|---|
| 1 | 10.658 | 18965 | 1592 | 0.356 | 0.321 |
| 2 | 11.150 | 5235994 | 486174 | 98.368 | 98.035 |
| 3 | 12.535 | 9405 | 1068 | 0.177 | 0.215 |
| 4 | 12.983 | 13142 | 1445 | 0.247 | 0.291 |
| 5 | 13.436 | 2721 | 328 | 0.051 | 0.066 |
| 6 | 13.666 | 1477 | 197 | 0.028 | 0.040 |
| 7 | 14.648 | 35630 | 4439 | 0.669 | 0.895 |
| 8 | 17.348 | 4511 | 570 | 0.085 | 0.115 |
| 9 | 19.068 | 1024 | 105 | 0.019 | 0.021 |
| Total | | 5322869 | 495917 | 100.000 | 100.000 |

MS Spectrum Graph

#:1 Ret.Time:Averaged 10.703-11.960(Scan#:989-1105)
BG Mode:Averaged 22.750-24.953(2101-2305)
Mass Peaks:558 Base Peak:243.70(1908001) Polarity:Pos Segment1 - Event1

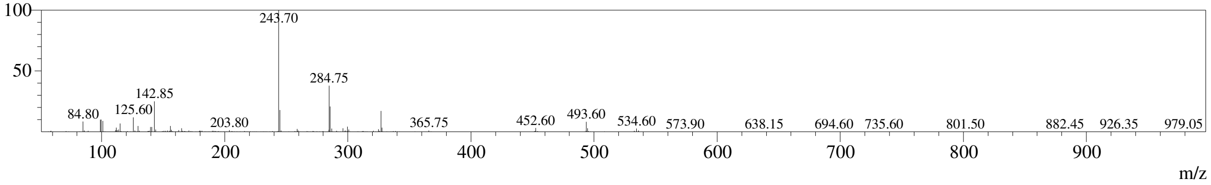


#:2 Ret.Time:Averaged 10.714-11.971(Scan#:990-1106)
BG Mode:Averaged 22.761-24.953(2102-2306)
Mass Peaks:607 Base Peak:208.60(2672712) Polarity:Neg Segment1 - Event2

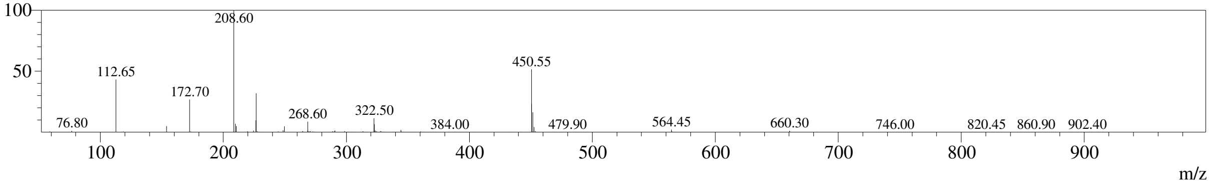

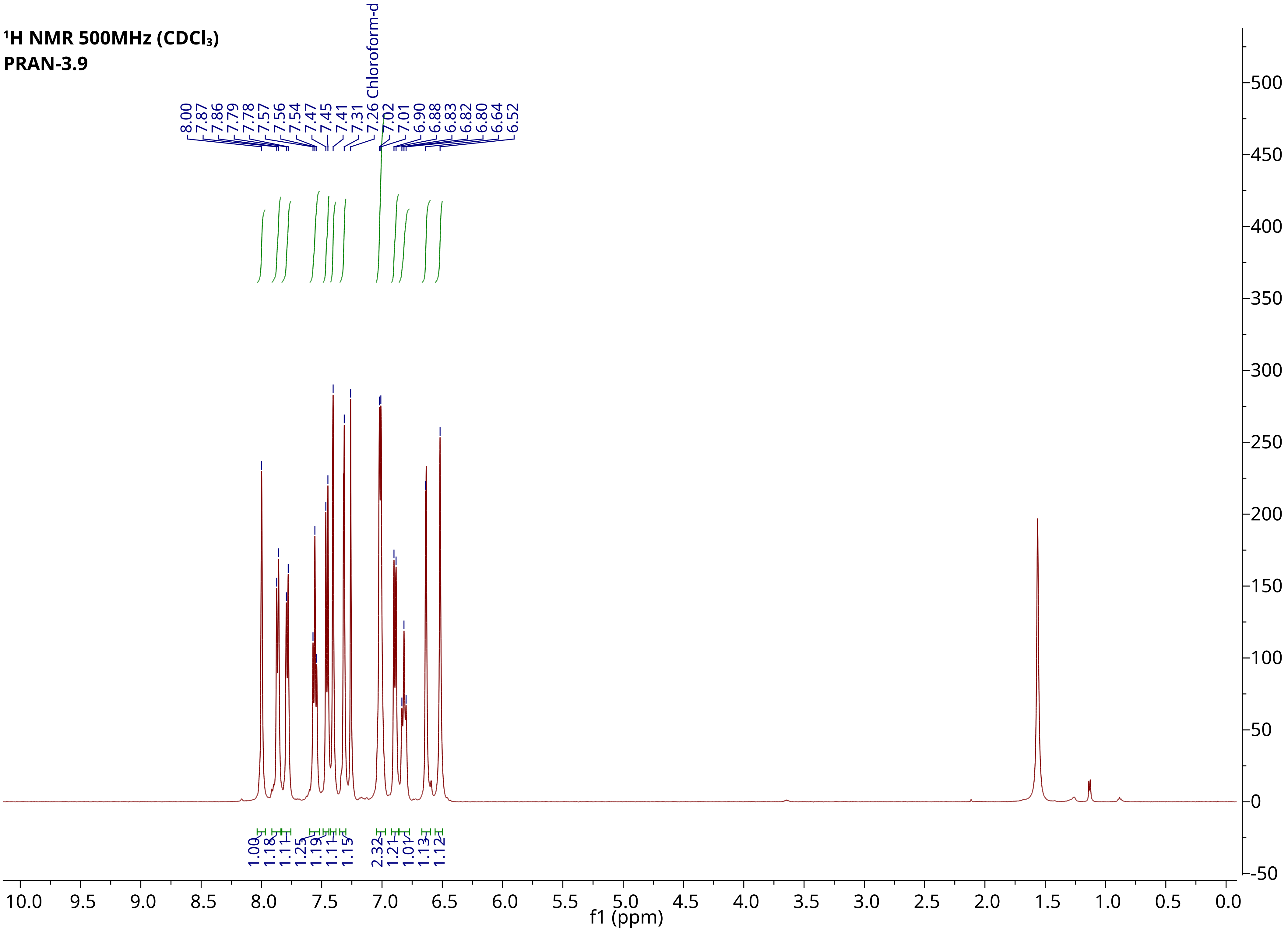

¹H NMR 500MHz (CDCl₃)
PRAN-3.9
8.00
7.87
7.86
7.79
7.78
7.57
7.56
7.54
7.47
7.45
7.41
7.31
7.26 Chloroform-d
7.02
7.01
6.90
6.88
6.83
6.82
6.80
6.64
6.52
1.00
1.18
1.11
1.25
1.19
1.11
1.11
1.15
2.32
1.21
1.01
1.13
1.12
f1 (ppm)

**$^{13}$C NMR**
**125.5MHz ($CDCl_3$)**
**PRAN-3.9**

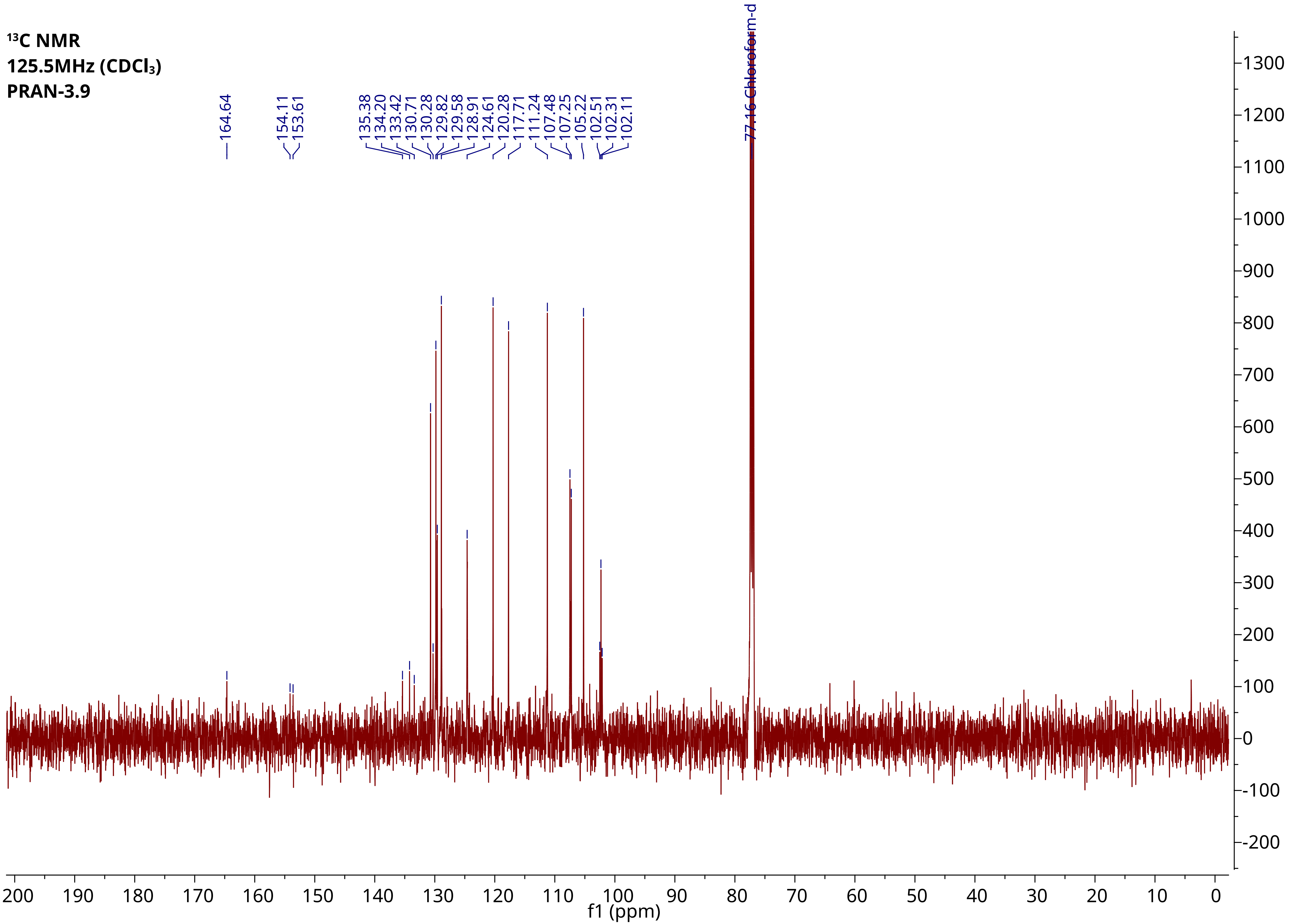

# ==== Shimadzu LCMSsolution Analysis Report ====

**Sample Name : PRAN-3.10**

Method

Column: Purospher RP-8
Mobile Phase A: H2O + 0.9% acetic acid
Mobile Phase B: ACN
% Pump B Concetrate: 50.0
Flow (ml/min): 0.6000

Detector A:SPD-20A
UV_1.Wavelength: 216
UV_2.Wavelength: 264
LC Program

| Time | Unit | Command | Value |
|---|---|---|---|
| 0.01 | Pumps | B.Conc | 50 |
| 15.00 | Pumps | B.Conc | 90 |
| 30.00 | Pumps | B.Conc | 90 |
| 30.01 | Pumps | B.Conc | 50 |
| 40.00 | Controller | Stop | |

MS Chromatogram

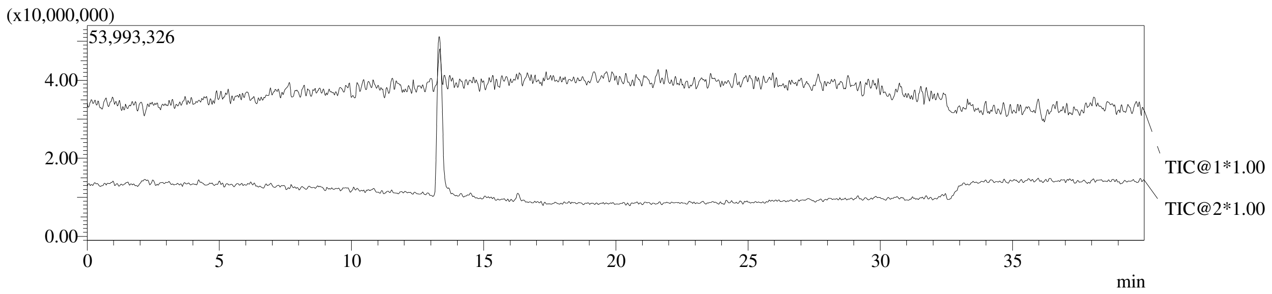


## <LC-UV Chromatogram>

Chromatogram

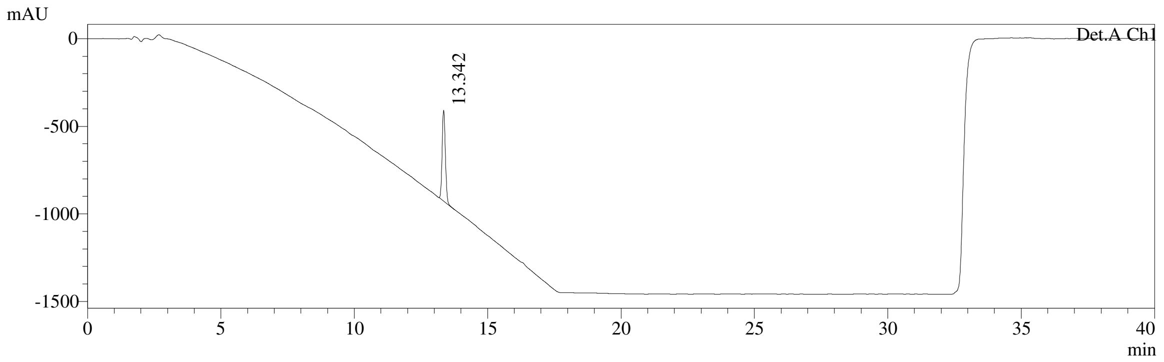

## Sample Name : PRAN-3.10

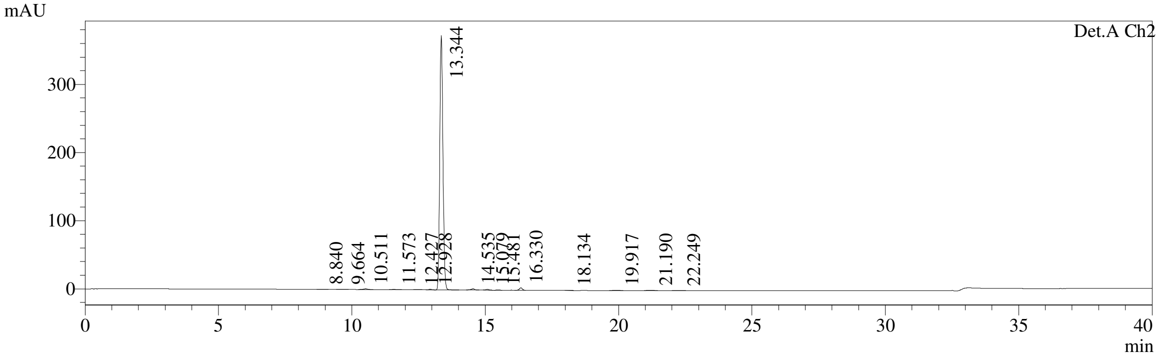


1 Det.A Ch1 / 216nm
2 Det.A Ch2 / 264nm

PeakTable

Detector A Ch2 264nm

| Peak# | Ret. Time | Area | Height | Area % | Height % |
|---|---|---|---|---|---|
| 1 | 8.840 | 1796 | 149 | 0.056 | 0.039 |
| 2 | 9.664 | 2271 | 178 | 0.071 | 0.046 |
| 3 | 10.511 | 12910 | 1131 | 0.404 | 0.295 |
| 4 | 11.573 | 3998 | 381 | 0.125 | 0.099 |
| 5 | 12.427 | 1298 | 121 | 0.041 | 0.031 |
| 6 | 12.928 | 8543 | 995 | 0.268 | 0.259 |
| 7 | 13.344 | 3091420 | 373346 | 96.796 | 97.215 |
| 8 | 14.535 | 18304 | 1814 | 0.573 | 0.472 |
| 9 | 15.079 | 9252 | 1096 | 0.290 | 0.285 |
| 10 | 15.481 | 2066 | 263 | 0.065 | 0.069 |
| 11 | 16.330 | 27734 | 3532 | 0.868 | 0.920 |
| 12 | 18.134 | 2220 | 250 | 0.070 | 0.065 |
| 13 | 19.917 | 5511 | 430 | 0.173 | 0.112 |
| 14 | 21.190 | 4164 | 224 | 0.130 | 0.058 |
| 15 | 22.249 | 2272 | 131 | 0.071 | 0.034 |
| Total | | 3193758 | 384042 | 100.000 | 100.000 |

MS Spectrum Graph

#:1 Ret.Time:Averaged 12.892-13.997(Scan#:1191-1293)
BG Mode:Averaged 22.317-26.395(2061-2437)
Mass Peaks:282 Base Peak:141.75(1326962) Polarity:Pos Segment1 - Event1

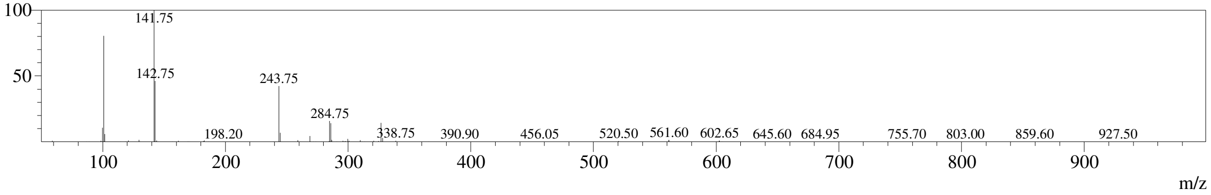


#:2 Ret.Time:Averaged 12.903-14.008(Scan#:1192-1294)
BG Mode:Averaged 22.328-26.395(2062-2438)
Mass Peaks:513 Base Peak:276.75(3008291) Polarity:Neg Segment1 - Event2

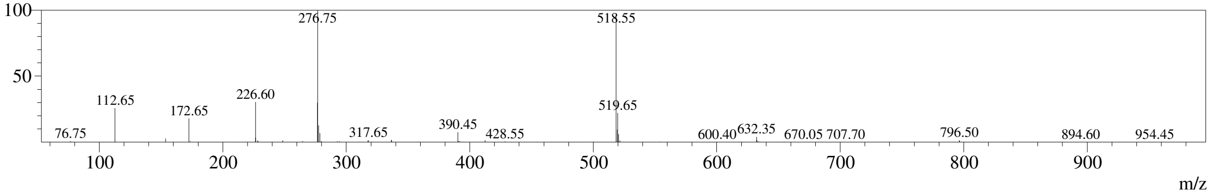

**$^1$H NMR 500MHz ($CDCl_3$)**
**PRAN-3.10**

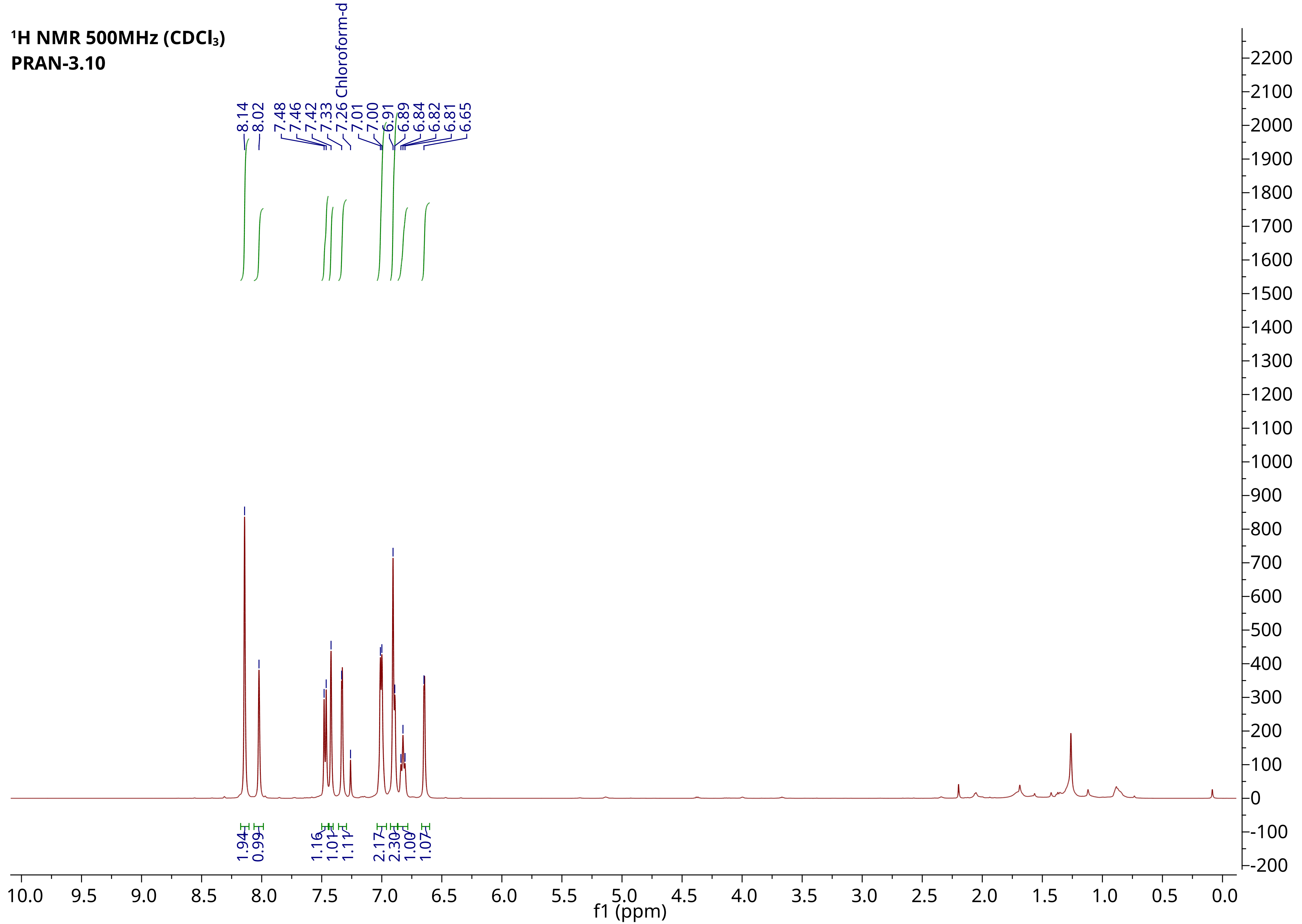

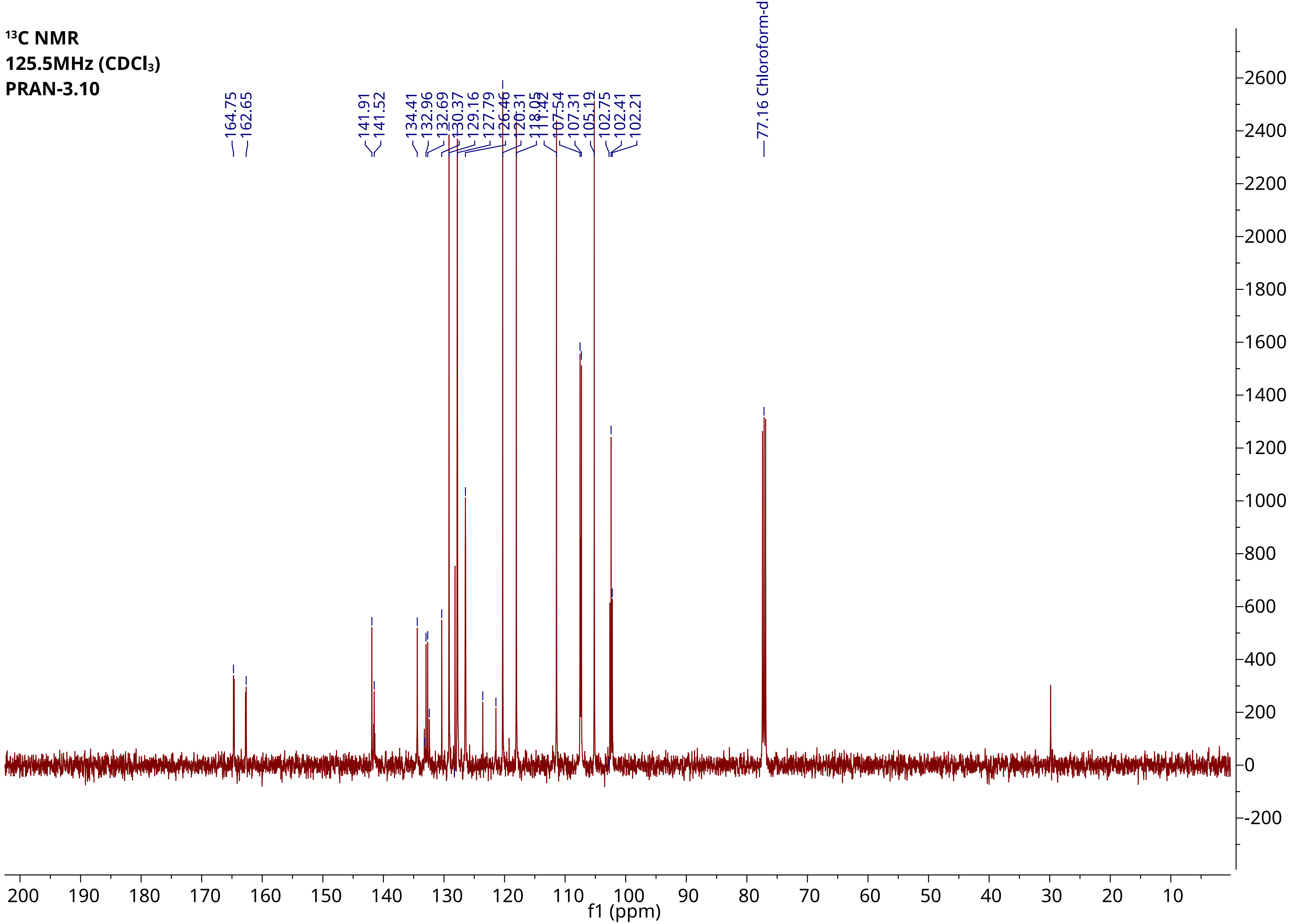

$^{13}$C NMR
125.5MHz (CDCl$_3$)
PRAN-3.10
164.75
162.65
141.91
141.52
134.41
132.96
132.69
130.37
129.16
127.79
126.46
120.31
118.05
111.42
107.54
107.31
105.19
102.75
102.41
102.21
77.16 Chloroform-d
f1 (ppm)
